# Polyketide Derivatives from the Macroalga-Associated Fungus *Penicillium sclerotiorum* Exhibiting Anti-Lymphangiogenic Effect

**DOI:** 10.1021/acs.jnatprod.5c01132

**Published:** 2025-11-11

**Authors:** Tzu-Yi Ke, Shih-Wei Wang, Zheng-Yu Lin, Govindarajan Ganesan, Cheng-Ta Lai, Juei-Yu Yen, Tian-Huei Chu, Yu-Chi Lin, Yuan-Bin Cheng

**Affiliations:** † Department of Marine Biotechnology and Resources, National Sun Yat-sen University, Kaohsiung 80424, Taiwan; ‡ Institute of Biomedical Sciences, MacKay Medical College, New Taipei City 25245, Taiwan; § Graduate Institute of Natural Products, College of Pharmacy, Kaohsiung Medical University, Kaohsiung 80708, Taiwan; ∥ Department of Medicine, 145474 MacKay Medical College, New Taipei City 25245, Taiwan; ⊥ Medical Laboratory, Medical Education and Research Center, Kaohsiung Armed Forces General Hospital, Kaohsiung 81304, Taiwan; # National Research Institute of Chinese Medicine, Ministry of Health and Welfare, Taipei 11221, Taiwan

## Abstract

Integrating genome mining with LC-MS/MS-based molecular networking analysis has revealed that the extract of the endophytic fungus *Penicillium sclerotiorum* represents a promising source for the discovery of new azaphilone analogues. Two rare polyketide derivatives (**1** and **2**), along with 11 new sclerotiorin-type azaphilones (**3**–**13**) and eight known compounds (**14**–**21**), were isolated from the macroalga-associated fungus *P. sclerotiorum*. The structural elucidation of these compounds was achieved using ECD, HR-ESI-MS, and NMR spectroscopic analyses. The absolute configurations of compounds **1** and **2** were determined through X-ray single-crystal diffraction. The antilymphangiogenic potential of these isolates was assessed *in vitro* using human lymphatic endothelial cells (LECs). Compounds **4** and **14**, both nitrogenated azaphilones, exhibited significant inhibitory activity, displaying IC_50_ values of 5.7 ± 0.2 and 5.8 ± 0.2 μg/mL, respectively.

## Graphical abstract

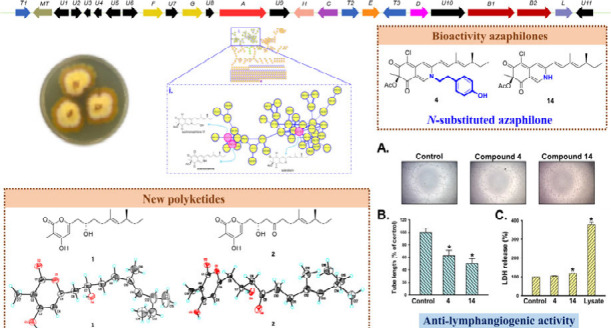

Marine microorganisms are an important source for the production of bioactive secondary metabolites, such as polyketides, alkaloids, peptides, and terpenes.^1,2^ Azaphilones are a larger group of fungal polyketide metabolites produced through the collaborative action of HR-PKSs and NR-PKSs.^3^ The skeleton is characterized by an isochromane scaffold featuring a pyrone–quinone bicyclic core and a quaternary carbon center that can be subdivided into 13 types based on structural patterns (citrinin-types, austdiols, deflectin-types, bulgarialactone-types, hydrogenated spiro-azaphilones, *O*-containing Monascus pigments, angular lactone-types, hydrogenated ones, chaetoviridins and chaephilones, pulvilloric acid-types, sclerotiorins, cohaerins and nitrogenated ones).^4^ These compounds are typically produced by genera such as *Penicillium*,^5^ *Monascus*,^6^ *Chaetomium*,^7^ and *Talaromyces*.^8^ Beyond their role as pigments,^6,9^ azaphilones have demonstrated a wide range of biological activities, including antimicrobial,^10^ cytotoxic,^11^ antiviral,^12^ and anti-inflammatory,^13^ emphasizing their promising potential for drug development.

In our previous study, extracts were obtained from cultures of the endophytic fungus *Penicillium sclerotiorum*, isolated from the macroalga *Grateloupia* sp. The extracts were analyzed using LC-MS/MS, and a molecular networking was constructed using the GNPS platform, resulting in 311 nodes and 15 molecular clusters (≥4 nodes) visualized in Cytoscape. In the molecular networking diagram (Figure 1), clusters A–C contained sclerotioramine, isochromophilone VI, sclerotiorin, geumsanol B, and geumsanol F, respectively. These compounds belong to the nitrogenated and sclerotiorin-type azaphilones,^4^ with their C-5 positions typically substituted by a chlorine atom and C-3 linking a branched C7 side chain. The unknown nodes in clusters A–C suggested the possibility of additional azaphilone analogues yet to be explored and discovered. The genome of *P. sclerotiorum* was sequenced and analyzed to identify genes involved in azaphilone biosynthesis. Using antiSMASH fungal version 8.0, a gene cluster associated with azaphilone production, designated as *Azp*, was identified and compared with a previously reported azaphilone biosynthetic gene cluster (*Aza* BGC) of *P. sclerotiorum* SNB-CN111 ^14^ (Figure 2). This comparison revealed that although the biosynthetic genes are conserved, their physical arrangements differ, which may lead to the production of additional, previously unknown metabolites.

**Figure 1:**
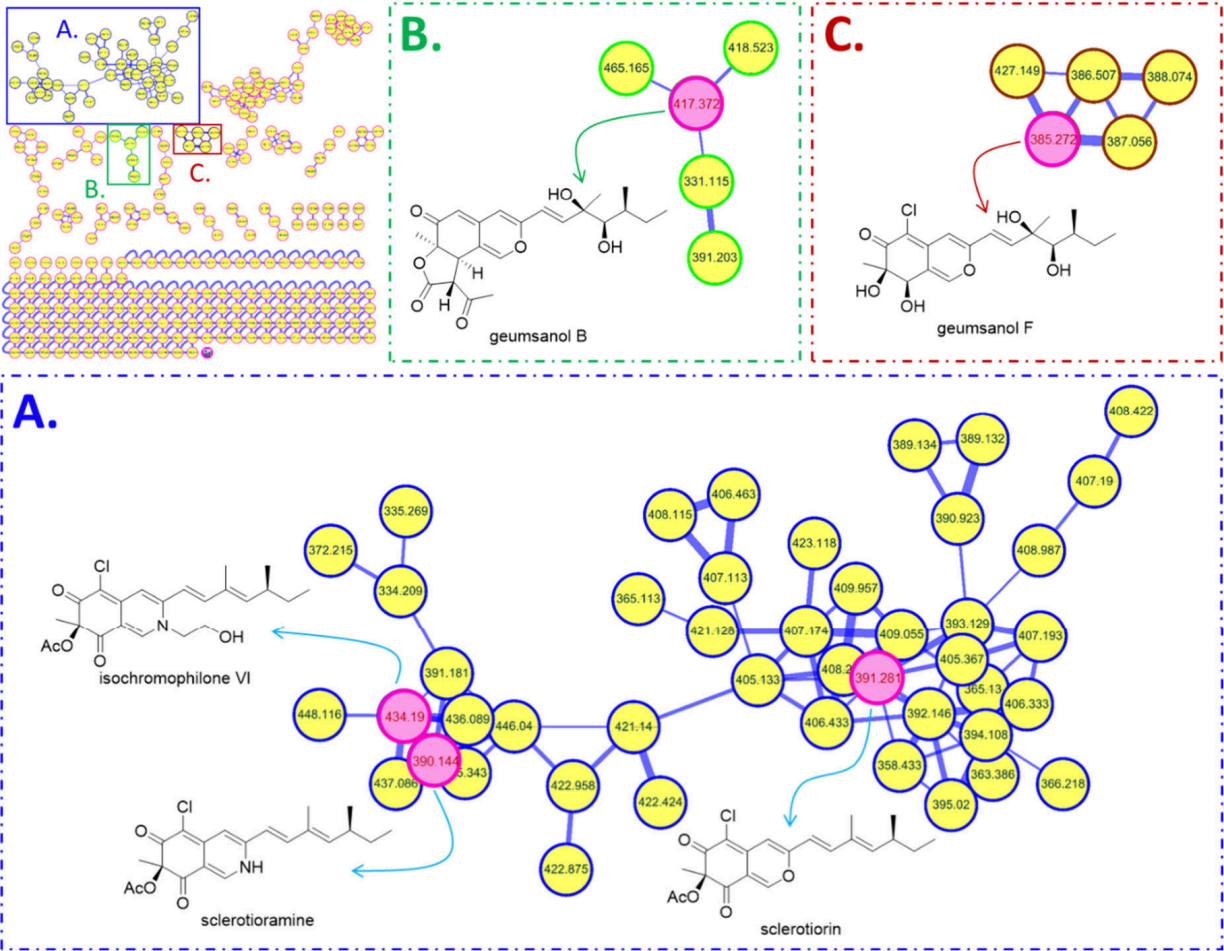
Molecular networking diagram of the extract from *P. sclerotiorum*. Pink nodes represent the known compounds identified through GNPS.

**Figure 2:**
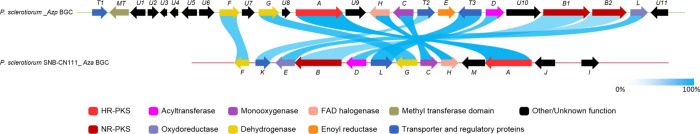
Comparison of the *Azp* gene cluster with the known *Aza* gene cluster (*P. sclerotiorum* SNB-CN111).

Therefore, a larger-scale fermentation of *P. sclerotiorum* was conducted, followed by chemical investigation of the growth media. Two rare polyketide derivatives (**1** and **2**), two new nitrogenated azaphilones (**3** and **4**), and nine new sclerotiorin-type azaphilones (**5**–**13**) were identified. Compounds **1**–**18** were evaluated for their antilymphangiogenic activity by their inhibitory effects on human lymphatic endothelial cells (LECs) growth. The bioactive compounds (**4** and **14**) were further subjected to tube formation experiments. Herein, the isolation, structural elucidation, and antilymphangiogenic activity of these metabolites are described.

## Results and Discussion

The fungal strain was cultivated in liquid medium which was extracted with EtOAc. The extract was subjected to fractionation via repeated column chromatography, leading to the isolation of new **1**–**13**. Known compounds included sclerotioramine (**14**),^15^ isochromophilone VI (**15**),^16^ (*R*)-5-chloro-3-((*S*,1*E*,3*E*)-3,5-dimethylhepta-1,3-dien-1-yl)-7-methyl-6,8-dioxo-2-phenethyl-2,6,7,8-tetrahydroisoquinolin-7-yl acetate (**16**),^17^ *N*-isoamylsclerotiorinamine (**17**),^18^ sclerazaphilone E (**18**),^13^ geumsanol C (**19**),^19^ *epi*-geumsanol D (**20**),^20^ and (7*R*,8*R*,8a*S*)-5-chloro-3-[(1*E*,3*R*,4*R*,5*S*)-3,4-dihydroxy-3,5-dimethyl-1-hepten-1-yl]-1,7,8,8a-tetrahydro-7,8-dihydroxy-7-methyl-6*H*-2-benzopyran-6-one (**21**),^21^ with structures confirmed by comparing their spectroscopic data with those reported in the literature.
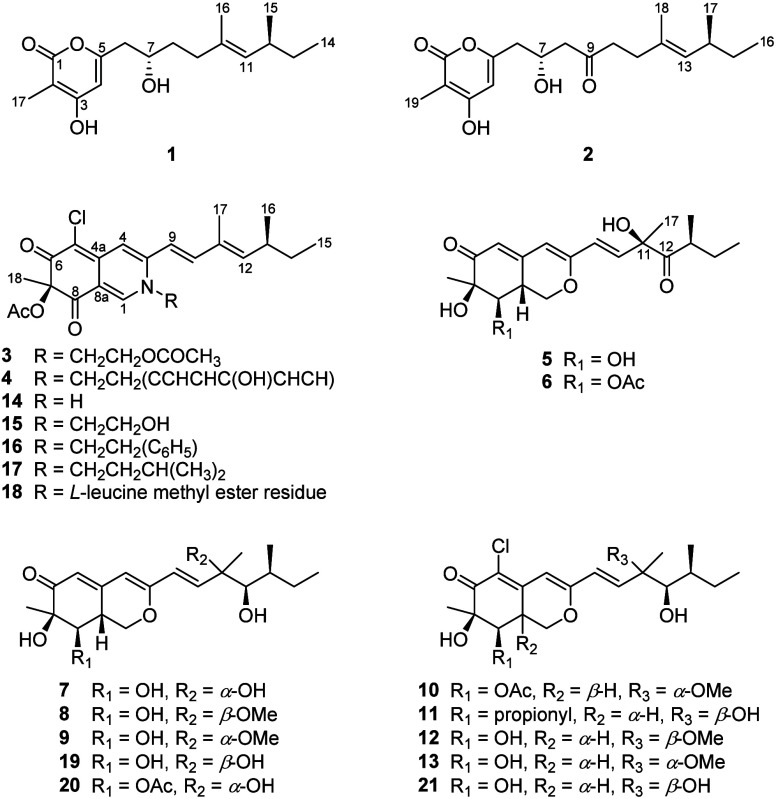


Sclerolactone A (**1**) was isolated as colorless crystals (mp 149–151 °C). Its molecular formula was determined as C_17_H_26_O_4_ by analysis of HRESIMS data *m*/*z* 317.1722 [M + Na]^+^, indicating five indices of hydrogen deficiency. The IR spectrum displayed absorptions characteristic of hydroxy (3431 cm^–1^) and carbonyl (1676 cm^–1^) functionalities. The UV spectrum showed maximum absorption bands at λ_max_ 200 and 290 (shoulder) nm. The ^1^H NMR spectrum of **1** (Table 1) exhibited two olefinic protons at δ_H_ 6.04 (s, H-4) and δ_H_ 4.93 (d, *J* = 9.5 Hz, H-11) and one oxymethine at δ_H_ 3.90 (m, H-7); four methylenes at δ_H_ 2.64 (dd, *J* = 14.5, 4.4 Hz, H-6a) and 2.50 (dd, *J* = 14.5, 4.4 Hz, H-6b), δ_H_ 1.59 (m, H_2_-8), δ_H_ 2.16 (m, H-9a) and 2.06 (m, H-9b), and δ_H_ 1.34 (m, H-13a) and 1.19 (m, H-13b); one methine at δ_H_ 2.25 (m, H-12); and four methyl groups at δ_H_ 0.84 (t, *J* = 7.4 Hz, H_3_-14), δ_H_ 0.91 (d, *J* = 6.6 Hz, H_3_-15), δ_H_ 1.62 (d, *J* = 1.3 Hz, H_3_-16) and δ_H_ 1.85 (s, H_3_-17). In addition, one ester carbonyl group at δ_C_ 169.2 (C-1); six olefinic carbons at δ_C_ 99.0 (C-2), δ_C_ 168.6 (C-3), δ_C_ 103.4 (C-4), δ_C_ 162.1 (C-5), δ_C_ 134.4 (C-10) and δ_C_ 132.8 (C-11); one oxymethine at δ_C_ 69.9 (C-7); four methylenes at δ_C_ 42.5 (C-6), δ_C_ 36.8 (C-8), δ_C_ 36.8 (C-9), and δ_C_ 31.6 (C-13); one methine at δ_C_ 35.3 (C-12) and four methyl groups at δ_C_ 12.4 (C-14), δ_C_ 21.4 (C-15), δ_C_ 16.4 (C-16), and δ_C_ 8.3 (C-17) were observed in the ^13^C NMR and DEPT spectra (Table 1).

**Table 1:** ^1^H (400 MHz) and ^13^C (100 MHz) NMR Data of **1** and **2**

**1** ^a^			**2** ^b^		
no.	δ_H_ (mult, *J* in Hz)	δ_C_ (type)	no.	δ_H_ (mult, *J* in Hz)	δ_C_ (type)
1		169.2, C	1		168.0, C
2		99.0, C	2		99.2, C
3		168.6, C	3		167.0, C
4	6.04, s	103.4, CH	4	6.16, s	103.4, CH
5		162.1, C	5		159.1, C
6a	2.64, dd (14.5, 4.4)	42.5, CH_2_	6	2.59, m	40.7, CH_2_
6b	2.50, dd (14.5, 8.4)		7	4.49, m	65.6, CH
7	3.90, m	69.6, CH	8	2.69, d (4.6)	48.5, CH_2_
8	1.59, m	36.8, CH_2_	9		211.5, C
9a	2.16, m	36.8, CH_2_	10	2.56, m	42.6, CH_2_
9b	2.06, m		11	2.20, t (7.6)	33.4, CH_2_
10		134.4, C	12		131.8, C
11	4.93, d (9.5)	132.8, CH	13	4.86, d (9.3)	132.3, C
12	2.25, m	35.3, CH	14	2.18, m	34.1, CH
13a	1.34, m	31.6, CH_2_	15a	1.28, m	30.5, CH_2_
13b	1.19, m		15b	1.14, m	
14	0.84, t (7.4)	12.4, CH_3_	16	0.78, t (7.4)	12.1, CH_3_
15	0.91, d (6.6)	21.4, CH_3_	17	0.86, d (6.6)	21.0, CH_3_
16	1.62, d (1.3)	16.4, CH_3_	18	1.57, s	16.4, CH_3_
17	1.85, s	8.3, CH_3_	19	1.84, s	8.3, CH_3_

a Data were measured in CD_3_OD. Chemical shifts are in ppm; *J* values in Hz are in parentheses.

b Data were measured in CDCl_3_. Chemical shifts are in ppm; *J* values in Hz are in parentheses.

The ^1^H–^1^H COSY cross-peaks observed between H_3_-14/H_2_-13, H_3_-15/H-12/H-11 and H_2_-6/H-7/H_2_-8/H_2_-9, along with HMBC correlations from H_3_-14 and H_2_-13 to C-12 and from H_3_-16 to C-9, C-10, and C-11, confirmed the side chain structure from C-6 to C-16 (Figure 3). Additional HMBC correlations from H_2_-6 to C-5 and C-4, from H-4 to C-2, C-3, and C-5, and from H_3_-17 to C-1, C-2, and C-3, combined with an analysis of the remaining degrees of unsaturation, indicated that C-1 is connected to a deshielded oxygenated carbon, C-5, via an ester bond, forming a δ-lactone ring. Furthermore, the deshielded chemical shifts of C-3 and C-7 suggested the presence of hydroxy groups at these positions; accordingly, the gross structure of compound **1** was determined.

**Figure 3:**
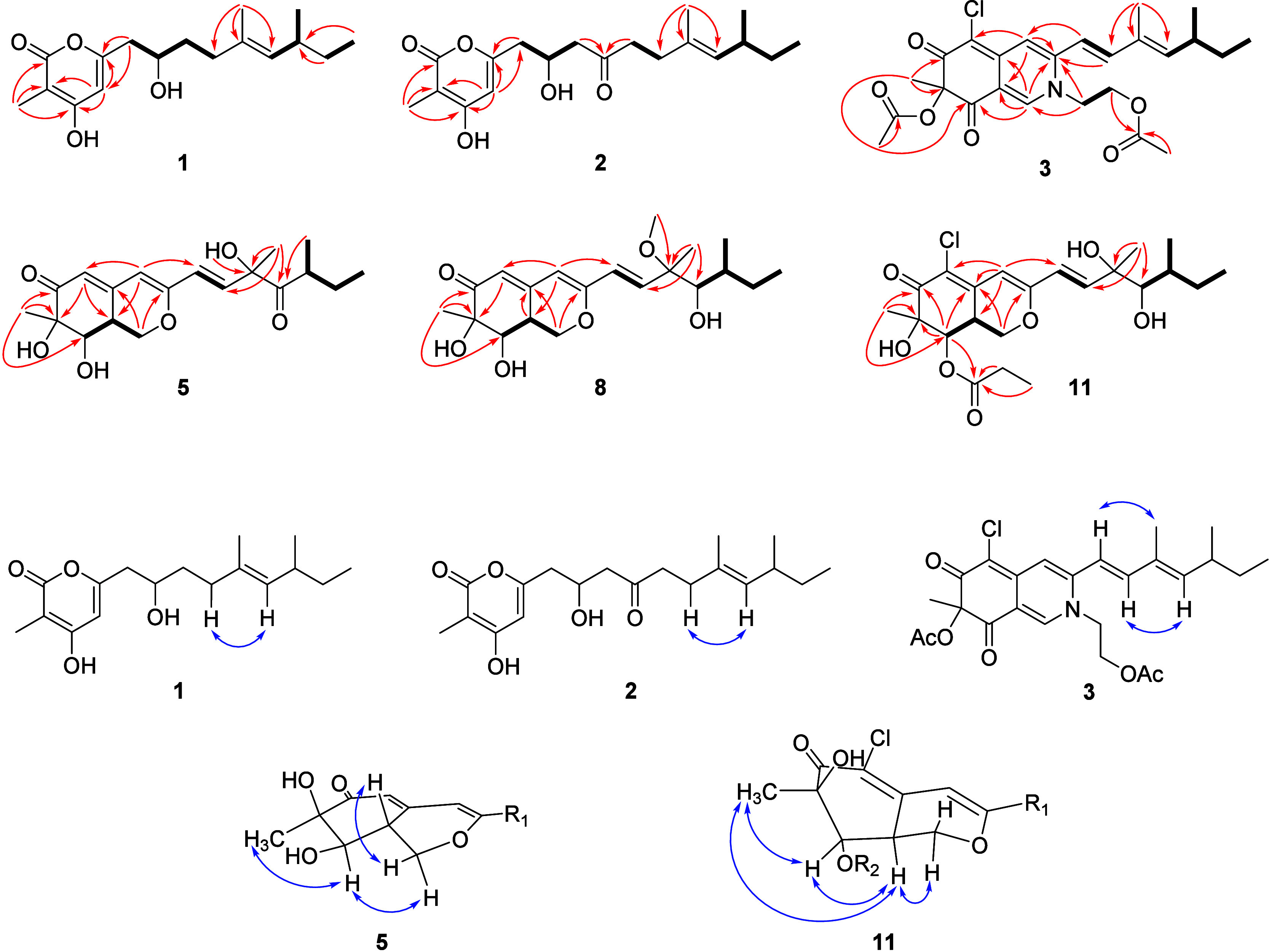
Key COSY (bold lines), HMBC (red arrows), and NOESY (blue double arrow) correlations of new compounds.

The geometry of the double bond Δ^10^ was determined as *E*-configuration by the NOESY correlations of H-9/H_2_-11 (Figure 3); however, due to the lack of the key NOESY correlations, the relative stereochemistry of the C-7 and C-12 in **1** could not be unambiguously defined. Suitable crystals were obtained from a mixture of MeOH and H_2_O by slow evaporation at room temperature afterward. The absolute configuration of **1** was then confirmed to be 7*S* and 12*S* by single-crystal X-ray diffraction analysis (Figure 4) using Cu Kα radiation with the Flack parameters of – 0.1(2) (CCDC 2405663).

**Figure 4:**
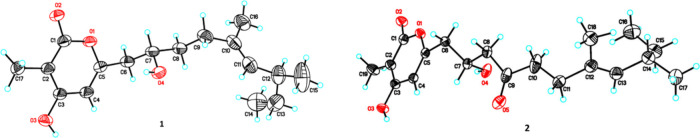
ORTEP diagrams for the crystal structures of **1** and **2** with 50% probability displacement ellipsoids.

Sclerolactone B (**2**) was obtained as colorless crystals with a mp of 109–111 °C. It presented the molecular formula C_19_H_28_O_5_, corresponding to six degrees of unsaturation by HRESIMS data. The IR spectrum of **2**, showing absorption bands at 3330, 1714, and 1680 cm^–1^, and the UV spectrum with λ_max_ at 200 and 288 (shoulder) nm, closely resembled those of **1**. The NMR data (Table 1) of **2** are similar to those of **1**, the main difference being that **2** bears one additional ketone carbonyl (δ_C_ 211.5, C-9) and one extra methylene (δ_H_ 2.56 and δ_C_ 42.6, C-10), as supported by the ^1^H–^1^H COSY correlations (Figure 3) of H_2_-11 (δ_H_ 2.20)/H_2_-10 (δ_H_ 2.56), as well as by HMBC correlations from both H_2_-10 and H_2_-8 (δ_H_ 2.69) to C-9. The geometry of the double bond Δ^12^ was deduced by the NOESY correlation of H-11/H-13 (δ_H_ 4.86) (Figure 3). The absolute configuration of **2** was determined to be 7*R*,14*S* based on the single-crystal X-ray diffraction data [Flack parameter: – 0.1(2)] (CCDC 2405665), as shown in Figure 4.

Peniphilone A (**3**) was purified as a red oil. Its molecular formula was identified as C_25_H_30_NO_6_Cl, requiring 11 degrees of unsaturation in line with its HRESIMS data *m*/*z* 476.1832 [M + H]^+^ (calcd for C_25_H_31_NO_6_Cl, 476.1834). Detailed analyses of 1D and 2D NMR data showed structurally close similarities to isochromophilone VI (**15**).^16^ The HMBC correlations from H_2_-2′ (δ_H_ 4.33) to C-1″ (δ_C_ 170.4) and from H_3_-2″ (δ_H_ 2.07) to C-1″, together with chemical shift values of C-2′ (δ_C_ 61.8) and C-1″, indicate the presence of an acetyl group, which should be connected to C-2′. The *E* geometries of double bonds Δ^9^ and Δ^11^ were deduced based on the coupling constants of H-9 and H-10 (*J* = 15.4 Hz) and the NOESY correlations of H-9 (δ_H_ 6.18)/H_3_-17 (δ_H_ 1.87) and H-10 (δ_H_ 6.97)/H-12 (δ_H_ 5.72) (Figure 3). The absolute configuration of 13*S* was confirmed through biosynthetic considerations cited in the literature^3,22^ and by comparing its NMR data with that of compound **15**. Additionally, the C-7 configuration was determined to be *R* based on the positive Cotton effect observed at 382 nm in the ECD spectrum,^23,24^ which closely resembles the ECD curves of **15** (Figure 5A). Based on the above analysis, the structure of **3** was unambiguously elucidated.

**Figure 5:**
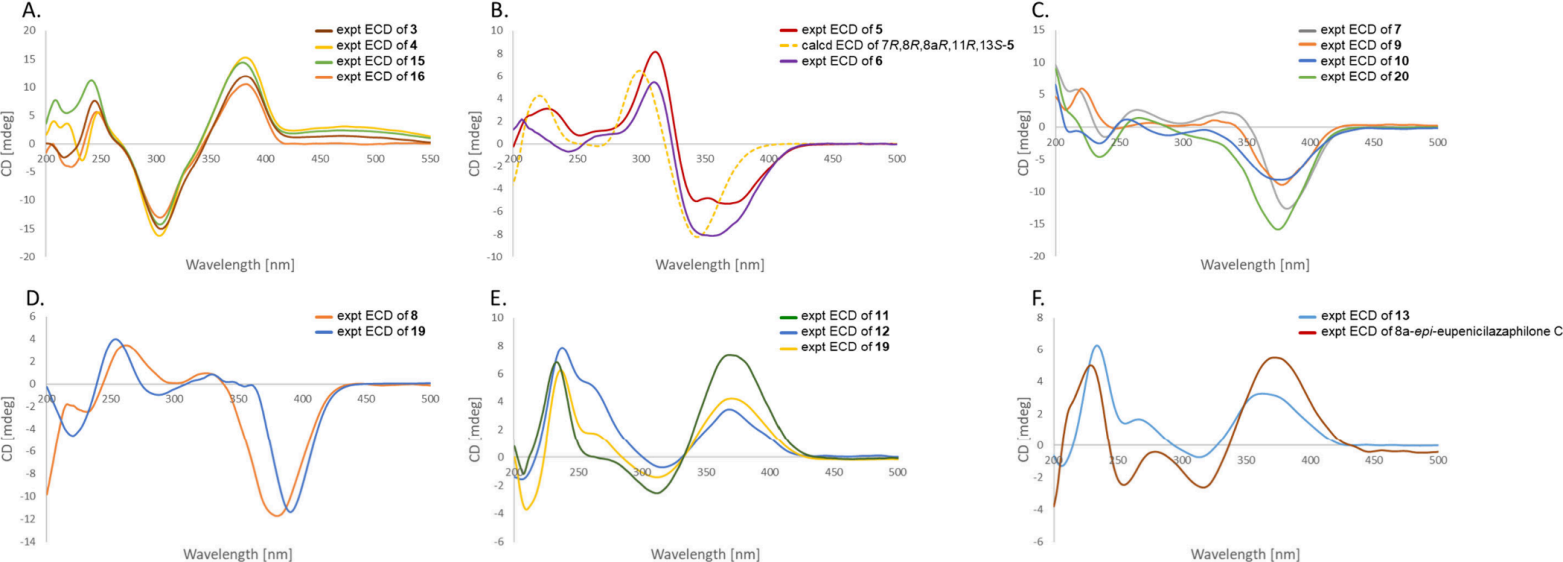
(A) ECD spectra of **3**, **4, 15**, and **16**. (B) ECD spectra of **5**, **6**, and 7*R*,8*R*,8a*R*,11*R*,13*S*-**5**. (C) ECD spectra of **7**, **9**, **10**, and **20**. (D) ECD spectra of **8** and **19**. (E) ECD spectra of **11**, **12**, and **19**. (F) ECD spectra of **13** and 8a-*epi*-eupenicilazaphilone C.

Peniphilone B (**4**) was obtained as a red oil with the molecular formula of C_29_H_32_NO_5_Cl based on its HRESIMS analysis *m*/*z* 532.1857 ([M + Na]^+^, calcd 532.1861). Comparison of the 1D and 2D NMR data (Table 2) with those of **16** ^17^ implied that they possessed a similar structure except for the presence of the phenol group in **4** instead of a phenyl group in **16**. The assignment of a hydroxyl group at C-6′ was determined by the HMBC correlations observed from H-4′ (δ_H_ 6.91) and H-5′ (δ_H_ 6.79) to C-6′ (δ_C_ 155.7), as well as by the downfield chemical shift of C-6′. In addition, the ECD spectrum of **4** showed good agreement with the experimental curve of **16**, supporting that **4** and **16** share the same absolute configurations.

**Table 2:** ^1^H (600 MHz) and ^13^C (150 MHz) NMR Data of **3** and **4** ^a^

**3**			**4**		
no.	δ_H_ (mult, *J* in Hz)	δ_C_ (type)	no.	δ_H_ (mult, *J* in Hz)	δ_C_ (type)
1	7.72, s	141.0, C	1	7.44, s	141.2, C
3		147.7, C	3		148.1, C
4	7.01, s	111.7, CH	4	6.99, s	111.8, CH
4a		144.0, C	4a		144.7, C
5		103.2, C	5		102.4, C
6		184.6, C	6		184.5, C
7		84.7, C	7		85.0, C
8		193.7, C	8		193.8, C
8a		114.9, C	8a		114.7, C
9	6.18, d (15.4)	114.4, CH	9	6.00, d (15.5)	114.7, CH
10	6.97, d (15.4)	145.4, CH	10	6.92, d (15.5)	145.2, CH
11		131.6, C	11		131.7, C
12	5.72, d (10.0)	148.4, CH	12	5.71, d (9.7)	148.3, CH
13	2.48, m	35.1, CH	13	2.48, m	35.2, CH
14a	1.45, m	30.0, CH_2_	14a	1.43, m	30.2, CH_2_
14b	1.36, m		14b	1.34, m	
15	0.89, t (7.4)	12.0, CH_3_	15	0.89, t (7.4)	12.1, CH_3_
16	1.03, d (6.7)	20.2, CH_3_	16	1.03, d (6.7)	20.4, CH_3_
17	1.87, s	12.5, CH_3_	17	1.87, s	12.8, CH_3_
18	1.56, s	23.1, CH_3_	18	1.52, s	23.4, CH_3_
19		170.1, C	19		170.4, C
20	2.18, s	20.3, CH_3_	20	2.16, s	20.5, CH_3_
1′	4.10, t (5.7)	51.9, CH_2_	1′	4.02, m	56.0, CH_2_
2′	4.33, t (5.7)	61.8, CH_2_	2′	2.95, m	35.7, CH_2_
1″		170.4, C	3′		127.4, C
2″	2.07, s	20.6, CH_3_	4′	6.91, d (8.6)	130.0, CH
			5′	6.79, d (8.6)	116.5, CH
			6′		155.7, C
			7′	6.79, d (8.6)	116.5, CH
			8′	6.91, d (8.6)	130.0, CH

a Data were measured in CDCl_3_. Chemical shifts are in ppm; *J* values in Hz are in parentheses.

Peniphilone C (**5**) was isolated as a pale-yellow oil. Its molecular formula was elucidated as C_19_H_26_O_6_ with seven degrees of unsaturation by a combination of HRESIMS (*m*/*z* 373.1621 [M + Na]^+^) and 1D NMR data. Analysis of the 1D and 2D NMR spectra suggested that **5** has a close structural relationship to geumsanol C (**19**).^19^ HMBC correlations from H_3_-16 (δ_H_ 1.06) and H_3_-17 (δ_H_ 1.49) to C-12 (δ_C_ 214.6), along with the deshielded chemical shift of C-12, indicate the presence of a carbonyl group at C-12 (Figure 3). The relative configurations at C-7, C-8, and C-8a of compounds **5** and **19** are identical, as it could be verified by the large coupling constant (^3^ *J*_H‑8/H‑8a_ = 9.4 Hz)^19,20^ and NOESY correlations between H_3_-18­(δ_H_ 1.48)/H-8 (δ_H_ 3.45)/H-1α (δ_H_ 3.78) and H-1β (δ_H_ 4.82)/H-8a (δ_H_ 3.01). The *E*-geometry of the Δ^9^ double bond was confirmed by measuring the coupling constant between H-9 and H-10 (*J* = 15.4 Hz). The *S* configuration was suggested for C-13 by biosynthetic considerations.^3,22^ To verify the absolute configuration of C-11, a theoretical ECD spectrum of **5** was calculated (Figure 5B). All conformers were optimized in Gaussian 16 at the B3LYP/6- 31G­(d) level. The ECD calculations of optimized conformers were carried out by the TDDFT method at the CAM-B3LYP/6–311++G­(d,p) level of theory with an IEFPCM solvent model in MeOH, with the spectra Boltzmann-averaged to obtain the ECD spectrum of the 11*R* and 11*S* isomers. Among all calculated stereoisomers, 7*R*,8*R*,8a*R*,11*R*,13*S*-**5** displayed the best overall agreement with the experimental data. Therefore, the results allowed the assignment of the absolute configuration of **5** to be 7*R*,8*R*,8a*R*,11*R*,13*S*.

Peniphilone D (**6**) was obtained as a pale-yellow oil. Its molecular formula was determined as C_21_H_28_O_7_ by HRESIMS, with a molecular ion peak at *m*/*z* 415.1728 [M + Na]^+^, indicating eight degrees of unsaturation. Structural similarities between **6** and **5** were apparent based on their almost identical 1D NMR data. The main difference between **6** and **5** at C-8 was the presence of an acetoxy group (δ_C_ 170.4 at C-19; δ_H_ 2.22 and δ_C_ 20.6 at C-20) in **6** (Table 3) instead of a hydroxy group in **5** confirmed by HMBC correlations observed from H-8 (δ_H_ 4.98) to C-19 and from H_3_-20 to C-19 as well as the deshielded chemical shift of H-8. Based on the NOESY correlations and the similar chemical shifts of their side chains, the relative configuration of **6** was suggested to be the same as that of **5**. Comparing the experimental ECD curves (Figure 5B) with those of **5**, the ECD spectrum of **6** showed a positive Cotton effect at 311 and a negative Cotton effect at 356 nm, which was in close agreement with the experimental curve for **5**, further indicating that **6** and **5** have the same absolute configurations.

**Table 3:** ^1^H and ^13^C NMR Data of **5**–**9**

	**5** ^a^		**6** ^a^		**7** ^b^		**8** ^a^		**9** ^a^	
no.	δ_H_ (mult, *J* in Hz)	δ_C_ (type)	δ_H_ (mult, *J* in Hz)	δ_C_ (type)	δ_H_ (mult, *J* in Hz)	δ_C_ (type)	δ_H_ (mult, *J* in Hz)	δ_C_ (type)	δ_H_ (mult, *J* in Hz)	δ_C_ (type)
1β	4.82, dd (10.9, 5.5)	69.0, CH_2_	4.36, dd (10.8, 5.3)	70.0, CH_2_	4.82, dd (11.2, 5.5)	69.0, CH_2_	4.83, dd (10.9, 5.4)	69.0, CH_2_	4.84, dd (10.9, 5.5)	69.0, CH_2_
1α	3.78, dd (13.6, 10.9)		3.80, dd (13.6, 10.8)		3.80, dd (12.9, 11.2)		3.80, dd (13.6, 10.9)		3.81, dd (13.6, 10.9)	
3		159.6, C		159.0, C		160.4, C		160.0, C		160.0, C
4	5.67, s	105.8, CH	5.70, s	105.5, CH	5.64, s	104.9, CH	5.66, s	105.1, CH	5.69, s	104.9, CH
4a		151.1, C		150.4, C		152.0, C		151.5, C		151.5, C
5	5.78, s	117.0, CH	5.82, s	117.2, CH	5.74, s	116.4, CH	5.77, s	116.6, CH	5.78, s	116.5, CH
6		196.4, C		194.2, C		196.8, C		196.5, C		196.4, C
7		74.4, C		74.4, C		74.4, C		74.4, C		74.4, C
8	3.45, t (9.8)	74.4, CH	4.98, d (10.0)	74.4, CH	3.44, t (9.8)	74.5, CH	3.46, t (9.7)	74.4, CH	3.46, d (9.4)	74.5, CH
8a	3.01, m	36.8, CH	3.35, m	34.7, CH	3.01, m	36.7, CH	3.02, m	36.8, CH	3.03, m	36.8, CH
9	6.30, d (15.4)	124.8, CH	6.31, d (15.3)	124.3, CH	6.20, d (15.6)	122.5, CH	6.01, d (16.0)	125.2, CH	5.99, d (16.1)	125.3, CH
10	6.52, d (15.4)	136.2, CH	6.49, d (15.3)	136.1, CH	6.47, d (15.6)	140.0, CH	6.37, d (16.0)	139.4, CH	6.46, d (16.1)	138.3, CH
11		79.5, C		79.3, C		76.2, C		80.5, C		80.7, C
12		214.6, C		214.3, C	3.50, d (7.0)	79.4, CH	3.41, dd (3.9, 2.7)	79.6, CH	3.47, d (2.2)	80.3, CH
13	2.88, m	42.2, CH	2.85, m	42.1, CH	1.56, m	36.4, CH	1.55, m	35.5, CH	1.50, m	35.8, CH
14a	1.73, m	27.1, CH_2_	1.73, m	26.9, CH_2_	1.42, m	28.9, CH_2_	1.40, m	29.0, CH_2_	1.43, m	29.3, CH_2_
14b	1.45, m		1.45, m		1.32, m		1.27, m		1.32, m	
15	0.89, t (7.6)	12.3, CH_3_	0.89, t (7.4)	12.1, CH_3_	0.91, t (7.5)	12.0, CH_3_	0.87, t (7.4)	11.9, CH_3_	0.89, t (7.4)	12.0, CH_3_
16	1.06, d (6.8)	18.5, CH_3_	1.04, d (6.8)	18.3, CH_3_	0.87, d (6.7)	13.4, CH_3_	0.92, d (6.8)	14.0, CH_3_	0.86, d (6.8)	13.9, CH_3_
17	1.49, s	25.0, CH_3_	1.49, s	24.8, CH_3_	1.37, s	26.8, CH_3_	1.31, s	16.5, CH_3_	1.33, s	16.8, CH_3_
18	1.48, s	20.8, CH_3_	1.36, s	19.9, CH_3_	1.48, s	20.6, CH_3_	1.49, s	20.7, CH_3_	1.49, s	20.8, CH_3_
19				170.4, C						
20			2.22, s	20.6, CH_3_						
11-OH	4.21, s		4.21, s							
11-OMe							3.17, s	50.4, CH_3_	3.19, s	50.6, CH_3_

a Data were measured at 600 MHz in CDCl_3_. Data were measured at 150 MHz in CDCl_3_. Chemical shifts are in ppm; *J* values in Hz are in parentheses.

b Data were measured at 400 MHz in CDCl_3_. Data were measured at 100 MHz in CDCl_3_. Chemical shifts are in ppm; *J* values in Hz are in parentheses.

Peniphilone E (**7**) was obtained as a yellow oil. Its molecular formula (C_19_H_28_O_6_) was deduced by analysis of HRESIMS (*m*/*z* 375.1777 [M + Na]^+^) and NMR data. Comparison of the NMR spectra of **7** and **20** ^20^ indicated that **7** lacks an acetoxy group at C-8, as observed by the shielded chemical shift of H-8 (δ_H_ 3.44). The NOESY correlations of H_3_-18 (δ_H_ 1.48)/H-8 (δ_H_ 3.44)/H-1α (δ_H_ 3.80) revealed that H_3_-18 and H-8 were α-oriented, and the NOESY correlation of H-1β (δ_H_ 4.82)/H-8a (δ_H_ 3.01) suggested that H-8a was β-oriented. The stereochemistry at C-11 and C-12 in the 3,5-dimethylhept-1-ene-3,4-diol moiety (C-9 to C-17) could be inferred from the observed chemical shift changes^19,20^ (Table S5). The *S* configuration of C-13 was suggested based on biosynthetic considerations.^3,22^ Considering the biogenetic origin and the similar chemical shifts, the relative configuration of **7** was proposed to be the same as that of **20**. Analysis of the ECD spectrum of **7** showed an identical curve to that of **20** (Figure 5C). Therefore, the absolute configuration of **7** was determined to be the same as **20**.

Peniphilone F (**8**) was isolated as a pale-yellow oil with the molecular formula C_20_H_30_O_6_, as evidenced by HRESIMS peak at *m*/*z* 389.1933 [M + Na]^+^. The NMR data of **8** were very similar to those of **19**,^19^ except for the appearance of a methoxy group (δ_C_ 50.4 and δ_H_ 3.17) in **8**. The HMBC correlations observed for this methoxy group indicated that it was attached at C-11. The relative configuration of **8** was deduced to be the same as that of **19** based on the NOESY correlations of H_3_-18 (δ_H_ 1.49)/H-8 (δ_H_ 3.46)/H-1α (δ_H_ 3.80) and H-1β (δ_H_ 4.83)/H-8a (δ_H_ 3.02) and similar ^1^H NMR data (Table S5). The absolute configuration of **8** was determined as 7*R*,8*R*,8a*R*,11*R*,12*R*,13*S* by comparing the experimental ECD spectra with that of **19** (Figure 5D).

Peniphilone G (**9**) presented same molecular formula as **8**, deduced from the HRESIMS peak at *m*/*z* 389.1933 [M + Na]^+^. The similar NMR data of **8** and **9** suggested that both compounds were a pair of C-11 epimers sharing the same planar structure. The relative configurations of C-7, C-8, and C-8a were assigned to be *R* configuration by observing NOESY correlations of H_3_-18 (δ_H_ 1.48)/H-8 (δ_H_ 3.44)/H-1α (δ_H_ 3.80), and H-1β (δ_H_ 4.82)/H-8a (δ_H_ 3.01) together with the large coupling constant of H-8/H-8a (^3^ *J* = 9.8 Hz). The ECD curves (Figure 5C) between **9** and **20** ^20^ were identical, indicating the absolute configuration of **9** as 7*R*,8*R*,8a*R*,11*S*,12*R*,13*S*.

Peniphilone H (**10**) was isolated as a pale-yellow oil. Its molecular formula was established as C_22_H_31_ClO_7_ by HRESIMS analysis at *m*/*z* 465.1650 [M + Na]^+^, indicating seven degrees of unsaturation. Although the ^1^H and ^13^C NMR spectra (Table 4) of **10** were similar to those of **9**, differences were observed at C-8 and C-5 in the isochromane core. A chlorine atom and an acetoxy group were observed and positioned at C-5 and C-8, respectively, based on the HRESIMS peak in a rough ratio of 3:1 and the HMBC correlations from H-8 (δ_H_ 5.00) and H_3_-20 (δ_H_ 2.22) to C-19 (δ_C_ 170.3). The absolute configuration of **10** was confirmed by its CD curve (Figure 5C), which showed the same characteristics as that of compound **9**.

**Table 4:** ^1^H (600 MHz) and ^13^C (150 MHz) NMR Data of **10**–**13** ^a^

	**10**		**11**		**12**		**13**	
no.	δ_H_ (mult, *J* in Hz)	δ_C_ (type)	δ_H_ (mult, *J* in Hz)	δ_C_ (type)	δ_H_ (mult, *J* in Hz)	δ_C_ (type)	δ_H_ (mult, *J* in Hz)	δ_C_ (type)
1β	4.39, dd (10.8, 5.0)	67.8, CH_2_	3.80, dd (13.0, 10.9)	67.3, CH_2_	4.25, dd (13.1, 11.2)	68.2, CH_2_	4.27, dd (13.1, 11.1)	68.3, CH_2_
1α	3.85, dd (13.7, 10.8)		4.48, dd (10.9, 4.8)		4.50, dd (11.2, 4.9)		4.53, dd (11.1, 4.9)	
3		161.2, C		160.8, C		160.8, C		160.8, C
4	6.13, s	102.1, CH	6.11, s	102.4, CH	6.09, s	102.9, CH	6.11, s	102.8, CH
4a		145.1, C		144.1, C		145.0, C		145.0, C
5		119.8, CH		117.2, CH		116.7, CH		116.7, CH
6		187.2, C		192.4, C		193.1, C		193.1, C
7		74.9, C		76.1, C		77.2, C		77.5, C
8	5.00, d (10.0)	73.1, CH	5.54, d (3.0)	73.9, CH	4.11, d (3.0)	73.6, CH	4.13, d (3.0)	73.6, CH
8a	3.47, m	35.6, CH	3.23, m	36.7, CH	3.05, m	36.7, CH	3.09, m	36.8, CH
9	6.09, d (16.0)	125.0, CH	6.30, d (15.5)	122.4, CH	6.09, d (16.0)	125.1, CH	6.10, d (16.0)	125.0, CH
10	6.51, d (16.0)	139.6, CH	6.55, d (15.5)	144.4, CH	6.42, d (16.0)	140.8, CH	6.51, d (16.0)	139.4, CH
11		80.5, C		75.8, C		80.4, C		80.5, C
12	3.48, brs	80.1, CH	3.50, brs	78.2, CH	3.40, brs	79.3, CH	3.50, d (2.7)	80.1, CH
13	1.47, m	35.5, CH	1.70, m	35.3, CH	1.56, m	35.3, CH	1.49, m	35.6, CH
14a	1.40, m	29.1, CH_2_	1.44, m	28.6, CH_2_	1.40, m	28.8, CH_2_	1.43, m	29.1, CH_2_
14b	1.28, m		1.35, m		1.23, m		1.31, m	
15	0.88, t (7.4)	11.9, CH_3_	0.92, t (7.4)	11.9, CH_3_	0.85, t (7.4)	11.7, CH_3_	0.89, t (7.4)	11.9, CH_3_
16	0.84, d (6.7)	13.7, CH_3_	0.97, d (6.8)	13.3, CH_3_	0.90, d (6.8)	13.8, CH_3_	0.84, d (6.6)	13.7, CH_3_
17	1.34, s	18.3, CH_3_	1.33, s	23.6, CH_3_	1.30, s	16.2, CH_3_	1.34, s	18.5, CH_3_
18	1.31, s	20.5, CH_3_	1.45, s	24.4, CH_3_	1.36, s	23.3, CH_3_	1.38, s	23.3, CH_3_
19		170.3, C		174.0, C				
20	2.22, s	20.6, CH_3_	2.29, m	27.3, CH_2_				
21			1.09, t (7.5)	9.1, CH_3_				
11-OMe	3.19, s	50.5, CH_3_			3.16, s	50.3, CH_3_	3.20, s	50.5, CH_3_

a Data were measured in CDCl_3_. Chemical shifts are in ppm; *J* values in Hz are in parentheses.

Peniphilone I (**11**) has the molecular formula C_22_H_31_ClO_7_, with seven degrees of unsaturation according to the HRESIMS data. Compound **11** is similar to **21** ^21^ based on a comparison of their NMR data (Table 4). The main difference between them is that the hydroxy group at C-8 in **21** is substituted by a propionyl group in **11**, as confirmed by the COSY correlations of H_2_-20 (δ_H_ 2.29)/H_3_-21 (δ_H_ 1.09) together with the HMBC correlations from H-8, H_2_-20, and H_3_-21 to C-19 (δ_C_ 174.0). The relative configuration of **11** was the same as that of **21**, as deduced from the NOESY correlations (Figure 3) of H_3_-18 (δ_H_ 1.45)/H-8a (δ_H_ 3.23) and H_3_-18/H-8 (δ_H_ 5.54)/H-8a/H-1α (δ_H_ 4.48), as well as by comparing the ^1^H NMR data of their side chain (Table S5). The positive Cotton effect at 368 nm in the experimental ECD spectrum of **11** (Figure 5E) suggested the *S* configuration at C-8a;^21^ moreover, the ECD spectra of **11** and **21** coincided well, so the absolute configuration of **11** was identified as 7*R*,8*R*,8a*S*,11*R*,12*R*,13*S*.

Peniphilone J (**12**) presented the molecular formula C_20_H_29_ClO_6_ as evidenced by the HRESIMS peak at *m*/*z* 423.1546 [M + Na]^+^. The NMR spectra of **12** were reminiscent of those of **21**,^21^ the main difference between these two compounds was the presence of a methoxy group at C-11 in **12** instead of a hydroxyl group in **21**, as shown by the HMBC correlation from the methoxy group (δ_H_ 3.16) to C-11 (δ_C_ 80.4). The ECD spectrum (Figure 5E) for **12** had a good match with **11** and **21**; accordingly, the absolute configuration of **12** was assigned as 7*R*,8*R*,8a*S*,11*R*,12*R*,13*S*.

The molecular formula of peniphilone K (**13**) was identical to that of **12** exhibited by the HRESIMS peak at *m*/*z* 423.1547 [M + Na]^+^, indicating that they share the same planar structure. The NMR signals assigned to **13** were very similar to those for **12**. The obvious differences between them were a small change in the chemical shifts of H_3_-15 (δ_H_ 0.89 in **13**; δ_H_ 0.85 in **12**), H_3_-16 (δ_H_ 0.84 in **13**; δ_H_ 0.90 in **12**), and H_3_-17 (δ_H_ 1.34 in **13**; δ_H_ 1.30 in **12**), implying that they are C-11 epimers (Table S5). Therefore, the absolute configuration of **13** was determined as 7*R*,8*R*,8a*S*,11*S*,12*R*,13*S* based on comparing the ECD spectra to 8a-*epi*-eupenicilazaphilone C (Figure 5F).^25^

Azaphilone biosynthesis begins with *AzpA* (Figure 6), which catalyzes the formation of the precursor moiety.^26,27^ The resulting precursor is subsequently transferred to the SAT domain of *AzpB*, where it undergoes chain extension to form three branching intermediates, as depicted in (i), (ii), and (iii). Compounds **1** and **2** are formed through the downstream reductase *AzpE* and spontaneous C1–C5 *O*-lactonization.^28^ The common intermediate of the azaphilone compounds is generated via C4a–C8a aldol condensation, followed by reductive release.^26^ This intermediate then undergoes hydroxylation catalyzed by *AzpC*,^27^ or alternatively, chlorination by *AzpH* introduces a halogen atom at C-5,^29^ after which *AzpC* catalyzes oxidation. These reactions lead to cyclization and dehydration, forming the characteristic azaphilone core structure. Subsequently, *AzpE* reduces the double bonds at C-8 and C-8a and introduces a hydrogen at the C-8a position,^30^ resulting in the formation of C-8a-enantioselective products. The C-11-enantioselective metabolites are produced through both nonenzymatic (introducing H_2_O) and enzymatic (*AzpC* and *AzpE*) transformations. In later stages, these compounds undergo methylation (*AzpMT*) and acetylation (*AzpD*), yielding compounds **5**–**13**. In addition, two nitrogenated azaphilones (**3** and **4**) are generated through the incorporation of endogenous amino acid derivatives.^31^

**Figure 6:**
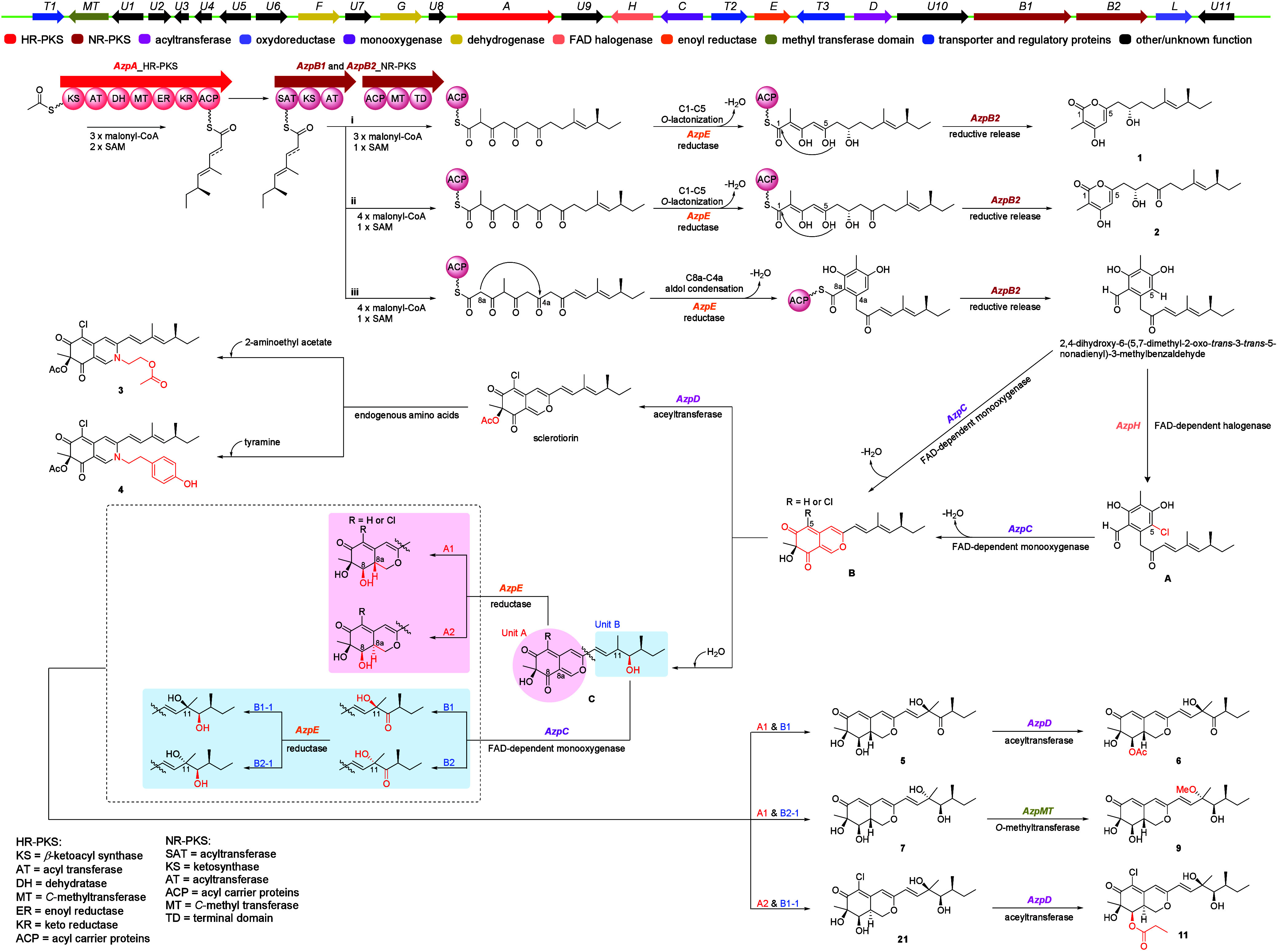
Proposed biosynthetic pathway for compounds **1**–**13**.

The isolated compounds were evaluated *in vitro* for their antilymphangiogenetic effect against human lymphatic endothelial cells (LECs). The results (Table 5) showed that **4** and **14** displayed significant inhibition activities with IC_50_ values of 5.7 ± 0.2 and 5.8 ± 0.2 μg/mL, respectively. Compounds **11**, **15**–**17** exhibited moderate to mild inhibition, with IC_50_ values ranging from 8.7 ± 0.4 to 20.9 ± 0.1 μg/mL. This finding suggested that nitrogenated azaphilones are significant inhibiters of lymphangiogenesis. Compounds **4** and **14** were further subjected to tube formation assay to validate the antilymphangiogeneic effect in human LECs. The results demonstrated that these compounds significantly inhibited tube formation in LECs (Figures 7A and 7B). Furthermore, compound **4** did not induce lactate dehydrogenase (LDH) release in LECs, suggesting that its inhibitory effect on lymphangiogenesis is not due to cytotoxicity. In contrast, compound **14** caused a slight increase in LDH release, implying that its antilymphangiogenesis activity may be associated with a certain degree of cytotoxicity (Figure 7C).

**Table 5:** Antilymphangiogenic Effects of Compounds **1**–**18**

LECs		LECs	
Compounds	IC_50_ (μg/mL)^a^	Compounds	IC_50_ (μg/mL)^a^
**1**	>25	**10**	>25
**2**	>25	**11**	20.9 ± 0.1
**3**	>25	**12**	>25
**4**	5.8 ± 0.2	**13**	>25
**5**	>25	**14**	5.7 ± 0.2
**6**	>25	**15**	18.7 ± 1.1
**7**	>25	**16**	9.2 ± 0.5
**8**	>25	**17**	8.7 ± 0.4
**9**	>25	**18**	>25

a Human LECs were treated with isolated compounds for 48 h, and antilymphangiogeneic activity was evaluated using a cell growth assay. Data are expressed as the mean ± SEM of five independent experiments.

**Figure 7:**
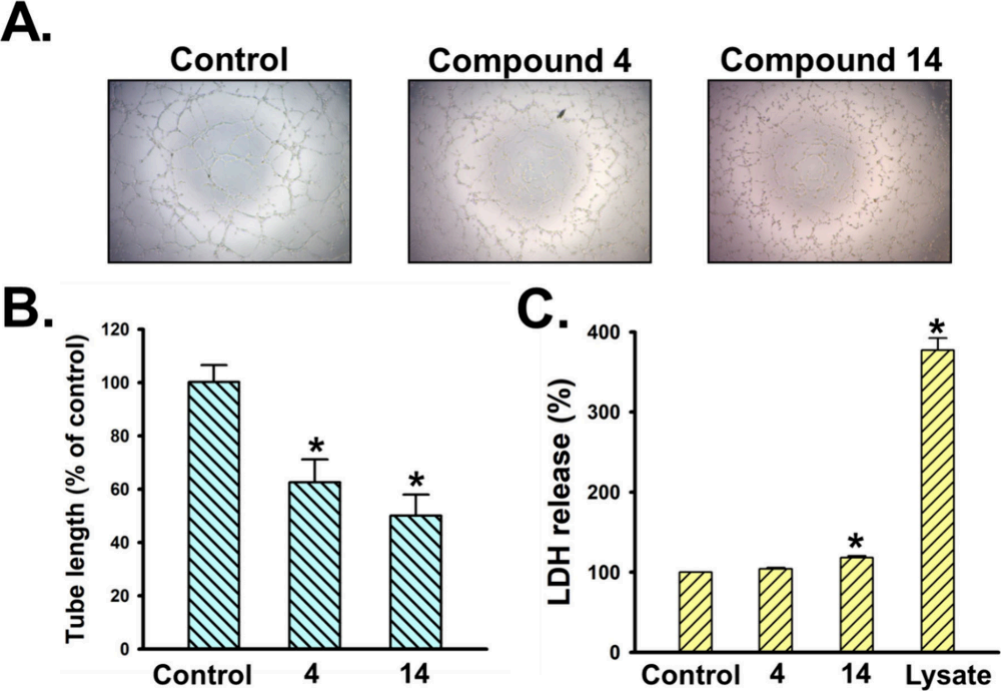
Effect of compounds **4** and **14** on tube formation and cytotoxicity of human LECs. LECs were treated with the indicated compounds at a concentration of 20 μg/mL for **8** hours. Capillary-like structure formation (A) and LDH release (C) were assessed using tube formation and cytotoxicity assays, respectively. Tubular morphogenesis was recorded using an inverted phase-contrast microscope. The extent of LEC tube formation was quantified by measuring tube length in ImageJ (B). Data represent the mean ± SEM of five independent experiments. *p < 0.05 compared with the control group.

In conclusion, the present study reports the discovery of two rare polyketides (**1** and **2**) and 11 azaphilone analogues (**3**–**13**) from the macroalga-associated fungus *P. sclerotiorum*. Among these, sclerolactones A (**1**) and B (**2**) represent uncommon polyketide scaffolds, while peniphilones A (**3**) and B (**4**) are classified as nitrogen-containing azaphilones. Notably, bioactivity assays revealed that the nitrogenated azaphilones exhibited significant antilymphangiogenic activity, with peniphilone B (**4**) showing the most potent effect. These findings expand the chemical diversity of *P. sclerotiorum*, particularly azaphilone derivatives, and indicate that azaphilones possess potential as antilymphangiogenic agents.

## Experimental Section

### General Experimental Procedures

Melting points were recorded on an MPA100-melting point apparatus. Optical rotations were measured on a Jasco P-2000 digital polarimeter. UV spectra were detected through a Jasco V-650 UV/vis spectrophotometer. A Jasco FTIR 4100 spectrometer was used for IR spectrum measurement. CD spectra were obtained on a Jasco J-815 CD spectrometer. NMR spectra were measured on either a Varian MR-400 MHz or Varian VNMRS 600 MHz NMR spectrometer with the residual CDCl_3_ (δ_H_ 7.26, δ_C_ 77.0) or CD_3_OD (δ_H_ 3.31, δ_C_ 49.0) as an internal standard. HRESIMS data collection was conducted on a Bruker 7T solariX spectrometer. Semipreparative HPLC was carried out using Shimadzu series HPLC instruments with a photodiode array detector (SPD-20A). Using various Phenomenex columns (10 mm × 250 mm, 5 μm), including Luna C_18_, Luna CN, Luna phenyl-hexyl, Kinetex F_5_, and Kinetex Biphenyl, to separate the target compounds.

### Fungal Material

The fungus *Penicillium sclerotiorum* was isolated from the inner tissue of the macroalgae *Grateloupia* sp. collected from the littoral area of Yilan, Taiwan, in May 2019, and was identified by its rDNA amplification and sequence analysis of the ITS region (GenBank accession number: KM265451.1).

### Genome Sequencing, Assembly, and antiSMASH Analysis

The genome of *P. sclerotiorum* was sequenced and assembled by GENOMICS, located in New Taipei, Taiwan. The FLO-PRO114 M R10.4.1 Flow cell (Nanopore, UK) was used for sequencing on a PromethION 2 platform with MinKNOW v24.06.16. A total of 100 ng of purified gDNA was used for sequencing. The gDNA was subjected to end-repair and A-tailing using the KAPA HyperPrep Kit (KAPA, USA). Barcode ligation and library construction were conducted in accordance with the manufacturer’s instructions using the SQK-NBD114.24 Native Barcoding Kit (Nanopore, UK). Base calling and sequence data processing, including demultiplexing, adapter trimming, and quality control (minimum acceptable Q-score of 7), were conducted using Dorado v7.4.14. Sequence read quality was assessed and visualized using NanoPlot v1.43.0. To investigate azaphilone biosynthesis, the genome of *P. sclerotiorum* was analyzed using antiSMASH fungal version 8.0, and the resulting gene clusters were annotated and functionally characterized using the NCBI BLASTP platform.^14^ The genome sequence was deposited in Genbank under accession No PV762938.

### Fermentation, Extraction, and Isolation

A fresh culture of *P. sclerotiorum* was transferred from an PDA agar culture to 500 mL Erlenmeyer flasks containing 300 mL potato dextrose broth (PDB) at 25 °C and shaken at 150 rpm for cultivation. After 14 days of incubation, 149.3 L of whole broth was extracted with EtOAc, which was filtered through cheesecloth to separate the supernatant from the mycelia. The EtOAc solutions were further separated and concentrated under reduced pressure to give the EtOAc extract (94.8 g). The extract was subjected to the silica gel chromatography column using a gradient elution of from hexanes/EtOAc (10:1 to 0:1, v/v) to EtOAc/MeOH (20:1 to 0:1, v/v) stepwise to give 11 fractions (Fr.1–Fr.11).

Fraction 7 was subjected to silica gel column chromatography (CC) eluted with hexanes/acetone (10:1 to 1:3, v/v) to obtain seven fractions (Fr. 7-A–Fr. 7-G). Fraction 7-E was subjected to silica gel column chromatography with a hexanes/EtOAc/MeOH gradient (from 4:1:0 to 0:0:1, v/v) to yield seven subfractions (Fr. 7-E-1–Fr. 7-E-7), and Fr. 7-E-4 was separated by ODS column eluted with MeOH/H_2_O 75:25 to MeOH/H_2_O 100:0 to obtain Fr.7-E-4-2, which was further purified by semipreparative HPLC (F_5_ column, MeCN/H_2_O 30:70, 2.5 mL/min) to afford **10** (10.2 mg, *t*_R_ = 78.8 min) and subfraction 7-E-4-2-a. Subfraction 7-E-4-2-a was further purified by semipreparative HPLC eluted with MeCN/H_2_O 40:60 (Biphenyl column, 2.0 mL/min) to yield **13** (1.3 mg, *t*_R_ = 13.3 min). Fraction 7-E-5 was separated by ODS column, eluting with MeOH/H_2_O gradient system (from MeOH/H_2_O 65:35 to MeOH/H_2_O 100:0) to give seven subfractions (Fr. 7-E-5-1–Fr. 7-E-5-7). Subfraction 7-E-5-7 was then purified by semipreparative HPLC (F_5_ column, 2.5 mL/min) eluted with MeCN/H_2_O 50:50 to yield **17** (2.3 mg, *t*_R_ = 40.1 min) and **18** (0.9 mg, *t*_R_ = 37.4 min). Fraction 7-F was separated by ODS column (from MeOH/H_2_O 50:50 to MeOH/H_2_O 100:0) to afford five fractions (Fr. 7-F-1–Fr. 7-F-5), fraction 7-F-4 followed by semipreparative HPLC (phenyl-hexyl column, MeOH/H_2_O 40:60, 2.5 mL/min) to afford **11** (1.4 mg, *t*_R_ = 36.8 min) and fraction 7-F-4-a. Fraction 7-F-4-a was further purified by semipreparative HPLC eluted with MeCN/H_2_O 30:70 (F_5_ column, 2.0 mL/min) to obtain **6** (0.6 mg, *t*_R_ = 51.4 min). Fraction 7-F-5 was subjected to a semipreparative HPLC with MeCN/H_2_O 30:70 (Biphenyl column, 2.0 mL/min) to obtain fraction 7-F-5-a, which was further purified by semipreparative HPLC using MeCN/H_2_O 35:65 (F_5_ column, 2.0 mL/min) to yield **12** (3.7 mg, *t*_R_ = 27.6 min).

Fraction 8 was further purified by silica gel column chromatography using an increasing gradient of from hexanes/acetone (10:1 to 0:1, v/v) to MeOH eluted to yield 15 fractions (Fr. 8-A–Fr. 8-O). Fraction 8-I was separated by semipreparative HPLC [C_18_ column, MeCN/H_2_O 65:35, 3.0 mL/min] to obtain **16** (1.4 mg, *t*_R_ = 37.2 min). Fraction 8-J was subjected to ODS column eluted with MeOH/H_2_O 30:70 to MeOH/H_2_O 100:0 to result in 14 subfractions, Fr. 8-J-1–Fr. 8-J-14, with Fraction 8-J-9 followed by semipreparative HPLC (C_18_ column, MeCN/H_2_O 55:45, 3.0 mL/min) to afford **3** (0.7 mg, *t*_R_ = 31.3 min). Fraction 8-K was subjected to ODS column eluted with MeOH/H_2_O 20:80 to MeOH/H_2_O 100:0 to yield 15 subfractions Fr. 8-K-1–Fr. 8-K-15. Fraction 8-K-5 was further separated through semipreparative HPLC using MeCN/H_2_O 20:80 (F_5_ column, 3.0 mL/min) to give **5** (3.6 mg, *t*_R_ = 49.7 min); Fraction 8-K-7 was further purified by semipreparative HPLC using MeCN/H_2_O 20:80 (F_5_ column, 3.0 mL/min) to yield **8** (1.6 mg, *t*_R_ = 54.2 min), **9** (0.9 mg, *t*_R_ = 57.0 min), **20** (8.9 mg, *t*_R_ = 72.2 min), and **21** (71.3 mg, *t*_R_ = 40.4 min); while Fraction 8-K-10 yielded **2** (45.5 mg) and Fraction 8-K-11 was subjected to semipreparative HPLC using MeCN/H_2_O 49:51 (C_18_ column, 3.0 mL/min) to afford **1** (6.9 mg, *t*_R_ = 20.3 min) and **14** (2.8 mg, *t*_R_ = 39.5 min). Fraction 8-L was fractionated by ODS column chromatography stepwise with MeOH/H_2_O 10:90 to MeOH/H_2_O 100:0 to give 15 subfractions Fr. 8-L-1–Fr. 8-L-15. Fraction 8-L-5 was further purified by semipreparative HPLC using MeCN/H_2_O 19:81 (C_18_ column, 3.0 mL/min) to yield **7** (15.1 mg, *t*_R_ = 61.7 min) and **19** (36.9 mg, *t*_R_ = 65.9 min); Fraction 8-L-11 was further separated through semipreparative HPLC using MeCN/H_2_O 35:75 (CN column, 2.0 mL/min), to give **15** (15.1 mg, *t*_R_ = 34.9 min); while Fraction 8-L-13 was fractionated by semipreparative HPLC using MeCN/H_2_O 45:55 (CN column, 2.0 mL/min), to give **4** (2.2 mg, *t*_R_ = 36.0 min).

#### Sclerolactone A (**1**)

colorless crystals; mp 149–151 °C; [α]_D_^23^ +22 (*c* 0.05, MeOH); UV (MeCN) λ_max_ (log *ε*) 200 (4.40), 290 (3.79) nm; ECD (MeOH) λ_max_ (Δ*ε*) 207 (+2.16), 283 (+2.53); IR (neat) *v*_max_ 3431, 2957, 2925, 2858, 2688, 1676, 1658, 1593,1506, 1415, 1384, 1256, 1180,1123, 1084 cm^–1^; ^1^H NMR (CD_3_OD, 400 MHz) and ^13^C NMR (CD_3_OD, 100 MHz) data, see Table 1; HRESIMS *m*/*z* 317.1722 [M + Na]^+^ (calcd for C_17_H_26_O_4_Na, 317.1723).

#### Sclerolactone B (**2**)

colorless crystals; mp 109–111 °C; [α]_D_^23^ +43 (*c* 0.05, MeOH); UV (MeCN) λ_max_ (log *ε*) 200 (4.46), 288 (3.78) nm; ECD (MeOH) λ_max_ (Δ*ε*) 206 (+1.91), 292 (+1.90); IR (neat) *v*_max_ 3331, 2958, 2921, 2870, 2700, 1714, 1681, 1592, 1413, 1380, 1256, 1178, 1123, 1084, 1002 cm^–1^; ^1^H NMR (CDCl_3_, 400 MHz) and ^13^C NMR (CDCl_3_, 100 MHz) data, see Table 1; HRESIMS *m*/*z* 359.1827 [M + Na]^+^ (calcd for C_19_H_28_O_5_Na, 359.1829).

#### Peniphilone A (**3**)

red oil; [α]_D_^23^ + 223 (*c* 0.03, MeOH); UV (MeOH) λ_max_ (log *ε*) 233 (4.08), 371 (4.19) nm; ECD (MeOH) λ_max_ (Δ*ε*) 217 (−0.88), 244 (+2.85), 305 (−5.63), 382 (+4.49); IR (neat) *v*_max_ 2958, 2919, 2851, 1743, 1706, 1592, 1500, 1465, 1371, 1313, 1236, 1195, 1145,1085, 1054, 1004 cm^–1^; ^1^H NMR (CDCl_3_, 600 MHz) and ^13^C NMR (CDCl_3_, 150 MHz) data, see Table 2; HRESIMS *m*/*z* 476.1832 [M + H]^+^ (calcd for C_25_H_31_NO_6_Cl, 476.1834).

#### Peniphilone B (**4**)

red oil; [α]_D_^23^ + 181 (*c* 0.05, MeOH); UV (MeOH) λ_max_ (log *ε*) 219 (4.17), 367 (4.32) nm; ECD (MeOH) λ_max_ (Δ*ε*) 209 (+3.72), 247 (+2.79), 303 (−7.06), 385 (+6.14); IR (neat) *v*_max_ 3357, 2981, 2947, 2890, 1745, 1712, 1586, 1506, 1381, 1242, 1156 cm^–1^; ^1^H NMR (CDCl_3_, 600 MHz) and ^13^C NMR (CDCl_3_, 150 MHz) data, see Table 2; HRESIMS *m*/*z* 532.1857 [M + Na]^+^ (calcd for C_29_H_32_NO_5_ClNa, 532.1861).

#### Peniphilone C (**5**)

pale-yellow oil; [α]_D_^23^ −179 (*c* 0.05, MeOH); UV (MeOH) λ_max_ (log *ε*) 352 (4.54), 238 (3.71) nm; ECD (MeOH) λ_max_ (Δ*ε*) 258 (+0.54), 308 (−3.97), 389 (−1.45); IR (neat) *v*_max_ 3444, 2989, 2951, 2895, 1720, 1659, 1596, 1468, 1396, 1300, 1278 cm^–1^; ^1^H NMR (CDCl_3_, 600 MHz) and ^13^C NMR (CDCl_3_, 150 MHz) data, see Table 3; HRESIMS *m*/*z* 373.1621 [M + Na]^+^ (calcd for C_19_H_26_O_6_Na, 373.1622).

#### Peniphilone D (**6**)

pale-yellow oil; [α]_D_^24^ −51 (*c* 0.05, MeOH); UV (MeOH) λ_max_ (log *ε*) 352 (3.60), 239 (3.00) nm; ECD (MeOH) λ_max_ (Δ*ε*) 211 (+0.05), 310 (−1.53), 380 (−1.10); IR (neat) *v*_max_ 3426, 2965, 2928, 2667, 1714, 1658, 1586, 1456, 1375, 1228, 1133, 1207 cm^–1^; ^1^H NMR (CDCl_3_, 600 MHz) and ^13^C NMR (CDCl_3_, 150 MHz) data, see Table 3; HRESIMS *m*/*z* 415.1728 ([M + Na]^+^, calcd for C_21_H_28_O_7_Na, 415.1727).

#### Peniphilone E (**7**)

yellow oil; [α]_D_^23^ −17 (*c* 0.05, MeOH); UV (MeOH) λ_max_ (log *ε*) 351 (4.56), 243 (3.67) nm; ECD (MeOH) λ_max_ (Δ*ε*) 216 (+0.93), 264 (+0.42), 331 (+0.38), 382 (−2.02); IR (neat) *v*_max_ 3388, 2983, 2948, 2893, 1662, 1596, 1468, 1464, 1396, 1267, 1183 cm^–1^; ^1^H NMR (CDCl_3_, 600 MHz) and ^13^C NMR (CDCl_3_, 150 MHz) data, see Table 4; HRESIMS *m*/*z* 375.1777 [M + Na]^+^ (calcd for C_19_H_28_O_6_Na, 375.1778).

#### Peniphilone F (**8**)

yellow oil; [α]_D_^23^ −54 (*c* 0.05, MeOH); UV (MeOH) λ_max_ (log *ε*) 350 (4.25), 241 (3.53) nm; ECD (MeOH) λ_max_ (Δ*ε*) 262 (+0.28), 325 (+0.08), 380 (−0.97); IR (neat) *v*_max_ 3415, 2981, 2950, 2893, 1663, 1596, 1466, 1396, 1347, 1304, 1282, 1232, 1130, 1070 cm^–1^; ^1^H NMR (CDCl_3_, 600 MHz) and ^13^C NMR (CDCl_3_, 150 MHz) data, see Table 4; HRESIMS *m*/*z* 389.1933 [M + Na]^+^ (calcd for C_20_H_30_O_6_Na, 389.1935).

#### Peniphilone G (**9**)

yellow oil; [α]_D_^24^ −108 (*c* 0.04, MeOH); UV (MeOH) λ_max_ (log *ε*) 350 (4.53), 238 (3.78) nm; ECD (MeOH) λ_max_ (Δ*ε*) 221 (+0.97), 274 (+0.10), 324 (+0.17), 378 (−1.49); IR (neat) *v*_max_ 3443, 2981, 2949, 2893, 1738, 1663, 1597, 1468, 1396, 1347, 1300, 1278, 1232, 1145, 1071 cm^–1^; ^1^H NMR (CDCl_3_, 600 MHz) and ^13^C NMR (CDCl_3_, 150 MHz) data, see Table 4; HRESIMS *m*/*z* 389.1933 [M + Na]^+^ (calcd for C_20_H_30_O_6_Na, 389.1935).

#### Peniphilone H (**10**)

yellow oil; [α]_D_^24^ −92 (*c* 0.05, MeOH); UV (MeOH) λ_max_ (log *ε*) 363 (4.24), 235 (3.29) nm; ECD (MeOH) λ_max_ (Δ*ε*) 217 (−0.12), 256 (+0.23), 317 (−0.09), 376 (−1.64); IR (neat) *v*_max_ 3341, 2963, 2935, 2881, 1738, 1675, 1565, 1456, 1373, 1230 cm^–1^; ^1^H NMR (CDCl_3_, 600 MHz) and ^13^C NMR (CDCl_3_, 150 MHz) data, see Table 5; HRESIMS *m*/*z* 465.1650 [M + Na]^+^ (calcd for C_22_H_31_ClO_7_Na, 465.1651).

#### Peniphilone I (**11**)

yellow oil; [α]_D_^24^ +113 (*c* 0.05, MeOH); UV (MeOH) λ_max_ (log *ε*) 358 (3.74), 232 (2.92) nm; ECD (MeOH) λ_max_ (Δ*ε*) 234 (+1.70), 312 (−0.64), 369 (+1.82); IR (neat) *v*_max_ 3434, 2960, 2925, 2863, 1734, 1701, 1594, 1499, 1371, 1245, 1213 cm^–1^; ^1^H NMR (CDCl_3_, 600 MHz) and ^13^C NMR (CDCl_3_, 150 MHz) data, see Table 5; HRESIMS *m*/*z* 465.1650 [M + Na]^+^ (calcd for C_22_H_31_ClO_7_Na, 465.1651).

#### Peniphilone J (**12**)

yellow oil; [α]_D_^24^ +66 (*c* 0.05, MeOH); UV (MeOH) λ_max_ (log *ε*) 361 (3.93), 234 (2.94) nm; ECD (MeOH) λ_max_ (Δ*ε*) 238 (+1.42), 316 (−0.13), 369 (+0.62); IR (neat) *v*_max_ 3430, 2978, 2928, 1669, 1567, 1459, 1402, 1370, 1264 cm^–1^; ^1^H NMR (CDCl_3_, 600 MHz) and ^13^C NMR (CDCl_3_, 150 MHz) data, see Table 5; HRESIMS *m*/*z* 423.1546 [M + Na]^+^ (calcd for C_20_H_29_ClO_6_Na, 423.1545).

#### Peniphilone K (**13**)

yellow oil;[α]_D_^24^ +288 (*c* 0.05, MeOH); UV (MeOH) λ_max_ (log *ε*) 362 (4.38), 234 (3.30) nm; ECD (MeOH) λ_max_ (Δ*ε*) 234 (+2.28), 267 (+0.60), 314 (−0.27), 363 (+1.18); IR (neat) *v*_max_ 3462, 2963, 2930, 2879, 1669, 1560, 1457, 1369, 1257, 1192, 1145 cm^–1^; ^1^H NMR (CDCl_3_, 600 MHz) and ^13^C NMR (CDCl_3_, 150 MHz) data, see Table 5; HRESIMS *m*/*z* 423.1547 [M + Na]^+^ (calcd for C_20_H_29_ClO_6_Na, 423.1545).

### X-ray Crystallographic Analyses of **1** and **2**

Compounds **1** and **2** were crystallized by slow evaporation from MeOH and H_2_O. Intensity data for crystal were collected on a Bruker APEX DUO single-crystal diffractometer with Cu Kα radiation (λ = 1.54178 Å) at 100 K. Crystallographic data for **1** (deposition no. CCDC 2405663) and **2** (deposition no. CCDC 2405665) have been deposited at the Cambridge Crystallographic Data Centre.

#### Crystal Data of **1**

monoclinic (0.150 × 0.100 × 0.010 mm^3^); space group *P*2_1_; unit cell dimensions *a* = 14.8068(10) Å, *b* = 6.6748(5) Å, *c* = 17.6717(13) Å, α = γ = 90°, β = 101.424(3)°,*V* = 1711.9(2) Å^3^; *Z* = 4; *d* = 1.142 Mg/m^3^; μ (CuKα) = 0.645 mm^–1^; *T* = 100(2) K; The total number of independent reflections measured was 6310, of which 5435 were observed [R­(int) = 0.0643]. Completeness to θ = 67.679°: 99.8%; Absorption correction: semiempirical from equivalents; Max. and min transmission: 1.00000 and 0.8303; goodness of fit on *F* ^2^ was 1.085; and the final refinement gave *R1* = 0.0582 and *wR2* = 0.1532 [*I > 2σ­(I)*] with absolute structure parameter = −0.1 (2).

#### Crystal Data of **2**

orthorhombic (0.150 × 0.020 × 0.015 mm^3^); space group *P*2_1_2_1_2_1_; unit cell dimensions *a* = 5.5157(2) Å, *b* = 15.6768(6) Å, *c* = 43.0946(19) Å, α = β = γ = 90°, *V* = 3726.3(3) Å^3^; *Z* = 8; *d* = 1.199 Mg/m^3^; μ (CuKα) = 0.697 mm^–1^; *T* = 100(2) K; The total number of independent reflections measured was 7089, of which 5498 were observed [R­(int) = 0.1421]. Completeness to θ = 67.679°: 99.9%; Absorption correction: semiempirical from equivalents; Max. and min transmission: 1.00000 and 0.7539; goodness of fit on *F* ^2^ was 1.023; with the final refinement giving *R1* = 0.0614 and *wR2* = 0.1486 [*I > 2σ­(I)*] and absolute structure parameter = −0.1 (2).

### ECD Calculation

A conformational distribution was generated using Spartan 20 under the MMFF force field, focusing on populations exceeding 1% for subsequent reoptimization. All identified minima were initially preoptimized using DFT at the B3LYP/6–31G­(d,p) level. Following this, TDDFT calculations for the reoptimized geometries were carried out at the CAM-B3LYP/6-311++G­(d,p) level, employing the IEF-PCM solvent model for MeOH, with Gaussian 16 software. The final ECD spectra were obtained by performing a Boltzmann averaging of all calculated spectra at 298 K.^32^

### Antilymphangiogenesis Assays

The protocols for cell culture, cell growth and the measurement of tube formation and cytotoxicity in human lymphatic endothelial cells have been previously described in detail.^33,34^

## Supplementary Material



## Notes

ECD calculation details of **5**, ^1^H NMR spectra of known compounds, crystallographic data of **1** and **2**, annotation of the *Azp* BGC, 1D and 2D NMR, HRESIMS, and UV spectra of **1**–**13** (PDF)
