# Supplementary material for: Polyketide Derivatives from the Macroalga-Associated Fungus Penicillium sclerotiorum Exhibiting Anti-Lymphangiogenic Effect
Source: J Nat Prod. 2025 Nov 11;88(11):2757–67. doi: 10.1021/acs.jnatprod.5c01132 (PMC12670496; doi:10.1021/acs.jnatprod.5c01132)
Supplement: Supplementary file 1 [file np5c01132_si_001.pdf]

Supporting information

Polyketide Derivatives from the Macroalga-Associated Fungus *Penicillium sclerotiorum* Exhibiting Anti-Lymphangiogenic Effect

Tzu-Yi Ke[a], Shih-Wei Wang[b,c], Zheng-Yu Lin[a], Govindarajan Ganesan[a], Cheng-Ta Lai[d], Juei-Yu Yen[b], Tian-Huei Chu[e], Yu-Chi Lin[f], and Yuan-Bin Cheng[a,]*

[a]Department of Marine Biotechnology and Resources, National Sun Yat-sen University, Kaohsiung 80424, Taiwan

[b]Institute of Biomedical Sciences, MacKay Medical College, New Taipei City 25245, Taiwan

[c]Graduate Institute of Natural Products, College of Pharmacy, Kaohsiung Medical University, Kaohsiung 80708, Taiwan

[d]Department of Medicine, Mackay Medical College, New Taipei City, Taipei 25245, Taiwan

[e]Medical Laboratory, Medical Education and Research Center, Kaohsiung Armed Forces General Hospital, Kaohsiung, Taiwan

[f]National Research Institute of Chinese Medicine, Ministry of Health and Welfare, Taipei 11221, Taiwan

*Corresponding authors,

E-mail address: jmb@mail.nsysu.edu.tw; orcid.org/0000-0001-6581-1320

Contents

Table S1. Energy analyses of 11*R*-**5** (seven conformers). S1
Table S2. Cartesian coordinates of the low-energy conformers of 11*R*-**5** re-optimized at B3LYP/6-31G(d,p) level. S2
Table S3. Energy analyses of 11*S*-**5** (six conformers). S9
Table S4. Cartesian coordinates of the low-energy conformers of 11*S*-**5** re-optimized at B3LYP/6-31G(d,p) level. S10
Table S5. ^{1}H NMR data of the 3,5-dimethylhept-1-ene-3,4-diol moiety of know compounds in $CDCl_3$. S16
Table S6. Crystal data and experimental details for **1**. S17
Table S7. Bond lengths [Å] and angles [°] for **1**. S18
Table S8. Crystal data and experimental details for **2**. S21
Table S9. Bond lengths [Å] and angles [°] for **2**. S22
Table S10 General genome sequencing data for *P. sclerotiorum* S26
Table S11 Deduced gene functions in *Azp* cluster. S27
Figure S1. HRESIMS spectrum of sclerolactone A (**1**) S28
Figure S2. UV spectrum of sclerolactone A (**1**) S28
Figure S3. ^{1}H NMR spectrum (400 MHz, CD_3OD) of sclerolactone A (**1**) S29
Figure S4. ^{13}C NMR spectrum (100 MHz, CD_3OD) of sclerolactone A (**1**) S29
Figure S5. ^{1}H-^{13}C HSQC spectrum of sclerolactone A (**1**) S30
Figure S6. COSY spectrum of sclerolactone A (**1**) S30
Figure S7. HMBC spectrum of sclerolactone A (**1**) S31
Figure S8. NOESY spectrum of sclerolactone A (**1**) S31
Figure S9. HRESIMS spectrum of sclerolactone B (**2**) S32
Figure S10. UV spectrum of sclerolactone B (**2**) S32
Figure S11. ^{1}H NMR spectrum (400 MHz, $CDCl_3$) of sclerolactone B (**2**) S33
Figure S12. ^{13}C NMR spectrum (100 MHz, $CDCl_3$) of sclerolactone B (**2**) S33
Figure S13. ^{1}H-^{13}C HSQC spectrum of sclerolactone B (**2**) S34
Figure S14. COSY spectrum of sclerolactone B (**2**) S34
Figure S15. HMBC spectrum of sclerolactone B (**2**) S35
Figure S16. NOESY spectrum of sclerolactone B (**2**) S35
Figure S17. HRESIMS spectrum of peniphilone A (**3**) S36
Figure S18. UV spectrum of peniphilone A (**3**) S36
Figure S19. ^{1}H NMR spectrum (600 MHz, $CDCl_3$) of peniphilone A (**3**) S37
Figure S20. ^{13}C NMR spectrum (150 MHz, $CDCl_3$) of peniphilone A (**3**) S37
Figure S21. ^{1}H-^{13}C HSQC spectrum of peniphilone A (**3**) S38
Figure S22. COSY spectrum of peniphilone A (**3**) S38
Figure S23. HMBC spectrum of peniphilone A (**3**) S39
Figure S24. NOESY spectrum of peniphilone A (**3**) S39
Figure S25. HRESIMS spectrum of peniphilone B (**4**) S40
Figure S26. UV spectrum of peniphilone B (**4**) S40
Figure S27. ^{1}H NMR spectrum (600 MHz, $CDCl_3$) of peniphilone B (**4**) S41

Figure S28. ^{13}C NMR spectrum (150 MHz, $CDCl_3$) of peniphilone B (**4**)....S41
Figure S29. ^{1}H-^{13}C HSQC spectrum of peniphilone B (**4**)....S42
Figure S30. COSY spectrum of peniphilone B (**4**)....S42
Figure S31. HMBC spectrum of peniphilone B (**4**)....S43
Figure S32. NOESY spectrum of peniphilone B (**4**)....S43
Figure S33. HRESIMS spectrum of peniphilone C (**5**)....S44
Figure S34. UV spectrum of peniphilone C (**5**)....S44
Figure S35. ^{1}H NMR spectrum (600 MHz, $CDCl_3$) of peniphilone C (**5**)....S45
Figure S36. ^{13}C NMR spectrum (150 MHz, $CDCl_3$) of peniphilone C (**5**)....S45
Figure S37. ^{1}H-^{13}C HSQC spectrum of peniphilone C (**5**)....S46
Figure S38. COSY spectrum of peniphilone C (**5**)....S46
Figure S39. HMBC spectrum of peniphilone C (**5**)....S47
Figure S40. NOESY spectrum of peniphilone C (**5**)....S47
Figure S41. HRESIMS spectrum of peniphilone D (**6**)....S48
Figure S42. UV spectrum of peniphilone D (**6**)....S48
Figure S43. ^{1}H NMR spectrum (600 MHz, $CDCl_3$) of peniphilone D (**6**)....S49
Figure S44. ^{13}C NMR spectrum (150 MHz, $CDCl_3$) of peniphilone D (**6**)....S49
Figure S45. ^{1}H-^{13}C HSQC spectrum of peniphilone D (**6**)....S50
Figure S46. COSY spectrum of peniphilone D (**6**)....S50
Figure S47. HMBC spectrum of peniphilone D (**6**)....S51
Figure S48. NOESY spectrum of peniphilone D (**6**)....S51
Figure S49. HRESIMS spectrum of peniphilone E (**7**)....S52
Figure S50. UV spectrum of peniphilone E (**7**)....S52
Figure S51. ^{1}H NMR spectrum (400 MHz, $CDCl_3$) of peniphilone E (**7**)....S53
Figure S52. ^{13}C NMR spectrum (100 MHz, $CDCl_3$) of peniphilone E (**7**)....S53
Figure S53. ^{1}H-^{13}C HSQC spectrum of peniphilone E (**7**)....S54
Figure S54. COSY spectrum of peniphilone E (**7**)....S54
Figure S55. HMBC spectrum of peniphilone E (**7**)....S55
Figure S56. NOESY spectrum of peniphilone E (**7**)....S55
Figure S57. HRESIMS spectrum of peniphilone F (**8**)....S56
Figure S58. UV spectrum of peniphilone F (**8**)....S56
Figure S59. ^{1}H NMR spectrum (600 MHz, $CDCl_3$) of peniphilone F (**8**)....S57
Figure S60. ^{13}C NMR spectrum (150 MHz, $CDCl_3$) of peniphilone F (**8**)....S57
Figure S61. ^{1}H-^{13}C HSQC spectrum of peniphilone F (**8**)....S58
Figure S62. COSY spectrum of peniphilone F (**8**)....S58
Figure S63. HMBC spectrum of peniphilone F (**8**)....S59
Figure S64. NOESY spectrum of peniphilone F (**8**)....S59
Figure S65. HRESIMS spectrum of peniphilone G (**9**)....S60
Figure S66. UV spectrum of peniphilone G (**9**)....S60
Figure S67. ^{1}H NMR spectrum (600 MHz, $CDCl_3$) of peniphilone G (**9**)....S61
Figure S68. ^{13}C NMR spectrum (150 MHz, $CDCl_3$) of peniphilone G (**9**)....S61
Figure S69. ^{1}H-^{13}C HSQC spectrum of peniphilone G (**9**)....S62
Figure S70. COSY spectrum of peniphilone G (**9**)....S62
Figure S71. HMBC spectrum of peniphilone G (**9**)....S63

Figure S72. NOESY spectrum of peniphilone G (**9**) S63
Figure S73. HRESIMS spectrum of peniphilone H (**10**) S64
Figure S74. UV spectrum of peniphilone H (**10**)...S64
Figure S75. ^{1}H NMR spectrum (600 MHz, $CDCl_3$) of peniphilone H (**10**)..........................S65
Figure S76. ^{13}C NMR spectrum (150 MHz, $CDCl_3$) of peniphilone H (**10**)........................S65
Figure S77. ^{1}H-^{13}C HSQC spectrum of peniphilone H (**10**) S66
Figure S78. COSY spectrum of peniphilone H (**10**) S66
Figure S79. HMBC spectrum of peniphilone H (**10**)..S67
Figure S80. NOESY spectrum of peniphilone H (**10**) S67
Figure S81. HRESIMS spectrum of peniphilone I (**11**) S68
Figure S82. UV spectrum of peniphilone I (**11**)...S68
Figure S83. ^{1}H NMR spectrum (600 MHz, $CDCl_3$) of peniphilone I (**11**) S69
Figure S84. ^{13}C NMR spectrum (150 MHz, $CDCl_3$) of peniphilone I (**11**) S69
Figure S85. ^{1}H-^{13}C HSQC spectrum of peniphilone I (**11**)...S70
Figure S86. COSY spectrum of peniphilone I (**11**)..S70
Figure S87. HMBC spectrum of peniphilone I (**11**)...S71
Figure S88. NOESY spectrum of peniphilone I (**11**) S71
Figure S89. HRESIMS spectrum of peniphilone J (**12**)...S72
Figure S90. UV spectrum of peniphilone J (**12**) S72
Figure S91. ^{1}H NMR spectrum (600 MHz, $CDCl_3$) of peniphilone J (**12**)..........................S73
Figure S92. ^{13}C NMR spectrum (150 MHz, $CDCl_3$) of peniphilone J (**12**)..........................S73
Figure S93. ^{1}H-^{13}C HSQC spectrum of peniphilone J (**12**) S74
Figure S94. COSY spectrum of peniphilone J (**12**) S74
Figure S95. HMBC spectrum of peniphilone J (**12**) S75
Figure S96. NOESY spectrum of peniphilone J (**12**)...S75
Figure S97. HRESIMS spectrum of peniphilone K (**13**) S76
Figure S98. UV spectrum of peniphilone K (**13**)..S76
Figure S99. ^{1}H NMR spectrum (600 MHz, $CDCl_3$) of peniphilone K (**13**)..........................S77
Figure S100. ^{13}C NMR spectrum (150 MHz, $CDCl_3$) of peniphilone K (**13**)......................S77
Figure S101. ^{1}H-^{13}C HSQC spectrum of peniphilone K (**13**) S78
Figure S102. COSY spectrum of peniphilone K (**13**)..S78
Figure S103. HMBC spectrum of peniphilone K (**13**)...S79
Figure S104. NOESY spectrum of peniphilone K (**13**) S79
Figure S105. Key COSY (bold lines), HMBC (red arrows), and NOESY (blue double arrow) correlations of new compounds. S80

Table S1. Energy analyses of 11*R*-**5** (seven conformers).

No.	3D conformers B3LYP/6-31G(d,p)	E (Hartree)	ΔE (kJ/mol)	Boltzmann distribution
11*R*-**5** (conformer 1)	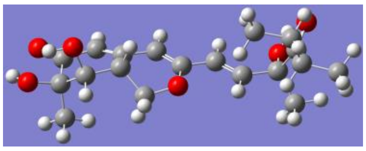	-1190.602247	0	75.03%
11*R*-**5** (conformer 2)	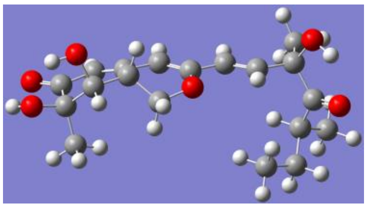	-1190.599092	8.283453131	2.65%
11*R*-**5** (conformer 3)	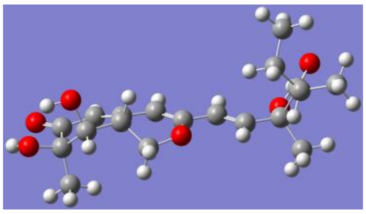	-1190.600916	3.494540766	18.32%
11*R*-**5** (conformer 4)	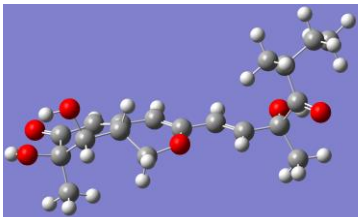	-1190.598540	9.732729241	1.48%
11*R*-**5** (conformer 5)	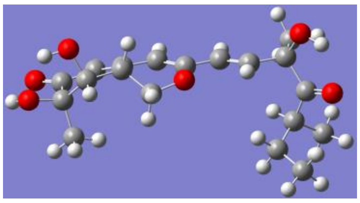	-1190.597815	11.63621689	0.69%
11*R*-**5** (conformer 6)	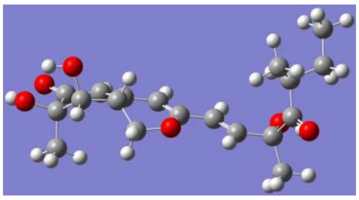	-1190.598216	10.58339131	1.05%
11*R*-**5** (conformer 7)	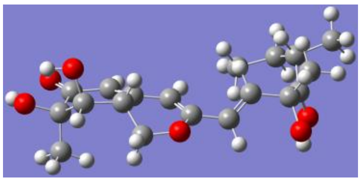	-1190.597769	11.7569899	0.65%

Table S2. Cartesian coordinates of the low-energy conformers of 11*R*-**5** re-optimized at B3LYP/6-31G(d,p) level.

11*R*-**5** (conformer 1)							
Atomic Type	Standard Orientation (Å)			Atomic Type	Standard Orientation (Å)		
	X	Y	Z		X	Y	Z
C	-4.852178	-0.71491	-0.801703	H	-2.012475	1.401459	-0.576418
C	-4.9644	0.177669	0.451865	H	-3.483609	-2.109488	-1.763289
C	-3.840811	1.237712	0.456075	H	-1.391982	1.560133	1.813334
C	-2.442982	0.669841	0.121584	H	-1.784826	-0.165071	2.033411
C	-2.413888	-0.66705	-0.598799	H	-0.940657	-2.088891	-1.372107
C	-3.537248	-1.23263	-1.122787	H	-4.000192	-1.237614	1.833194
C	-1.47793	0.597724	1.30424	H	-5.115045	-0.071616	2.594101
O	-0.144022	0.284266	0.860784	H	-5.754253	-1.438087	1.661952
C	-0.028226	-0.72522	-0.041893	H	-6.713544	0.366693	-0.291523
C	-1.098568	-1.23021	-0.727503	H	-5.101024	2.32081	-0.478926
O	-5.889657	-1.00886	-1.396951	H	1.464107	-2.002359	-0.962736
C	-4.947074	-0.70052	1.713463	H	2.24814	-0.02544	1.241302
O	-6.203407	0.87376	0.378602	H	4.222285	1.214069	1.190898
O	-4.130635	2.216751	-0.52605	H	4.879328	3.24884	-0.121001
C	1.324622	-1.22607	-0.216895	H	5.219517	2.227329	-1.515606
C	2.385259	-0.78934	0.480989	H	2.965663	3.318887	-1.702933
C	3.791603	-1.31383	0.27358	H	2.792197	1.561415	-1.641125
C	4.643351	-0.18742	-0.385812	H	2.439852	2.534192	-0.206246
C	4.879342	1.145451	0.31538	H	5.444534	-2.074647	1.469964
C	4.576588	2.326366	-0.633188	H	3.835194	-2.570985	2.025875
C	3.111711	2.43848	-1.067724	H	4.409515	-0.923107	2.357089
C	4.415207	-1.73551	1.623121	H	4.327848	-2.140785	-1.366598
O	3.793532	-2.424	-0.595014	H	6.543983	2.146305	1.297376
O	5.135741	-0.42022	-1.478708	H	6.57618	0.387069	1.500592
C	6.34692	1.193035	0.795523	H	7.026678	1.103573	-0.057822
H	-3.817616	1.702293	1.458843				

11*R*-**5** (conformer 2)							
Atomic Type	Standard Orientation (Å)			Atomic Type	Standard Orientation (Å)		
	X	Y	Z		X	Y	Z
C	-4.66398	0.214447	1.260094	H	-2.52522	-1.75853	-0.7794
C	-4.81296	0.635783	-0.21658	H	-3.13935	-0.07886	2.787143
C	-4.04519	-0.34391	-1.13124	H	-1.59192	-0.27519	-2.52327
C	-2.61774	-0.6725	-0.63903	H	-1.4565	1.04666	-1.33461
C	-2.33748	-0.41269	0.830892	H	-0.67734	-0.59528	2.244769
C	-3.32841	-0.11539	1.717069	H	-3.27982	2.213362	-0.16261
C	-1.49266	-0.04441	-1.46064	H	-4.53138	2.411161	-1.4178
O	-0.21214	-0.58171	-1.07686	H	-4.91348	2.742834	0.281748
C	0.026666	-0.69191	0.253355	H	-6.64398	0.510416	0.314237
C	-0.95741	-0.57532	1.196262	H	-5.69147	-1.30471	-1.19262
O	-5.66012	0.271514	1.981955	H	1.611937	-1.06633	1.658618
C	-4.3445	2.089906	-0.38772	H	2.184382	-0.91212	-1.35999
O	-6.1912	0.541602	-0.5576	H	3.611107	1.072823	1.232412
O	-4.7515	-1.57084	-1.18241	H	4.325508	3.349558	0.446265
C	1.420352	-0.93413	0.597269	H	5.211579	2.599236	-0.87896
C	2.413943	-1.02488	-0.30364	H	2.975418	3.471698	-1.62938
C	3.873674	-1.31719	-0.02353	H	3.063734	1.721502	-1.84787
C	4.687437	-0.00925	-0.27452	H	2.157708	2.396576	-0.48403
C	4.504614	1.215248	0.613047	H	5.220198	-2.06866	1.493091
C	4.328402	2.491561	-0.23837	H	3.600687	-2.78351	1.533283
C	3.0593	2.518596	-1.09581	H	3.861922	-1.14696	2.178627
C	4.153225	-1.85003	1.392175	H	4.984	-1.8284	-1.50358
O	4.304185	-2.28384	-0.96351	H	5.632477	2.223037	2.178204
O	5.491382	-0.0129	-1.19353	H	5.86931	0.468019	2.176038
C	5.739336	1.344568	1.53288	H	6.647303	1.464617	0.933043
H	-3.9914	0.105338	-2.13966				

11*R*-**5** (conformer 3)							
Atomic Type	Standard Orientation (Å)			Atomic Type	Standard Orientation (Å)		
	X	Y	Z		X	Y	Z
C	-4.86835	-0.57743	-0.91947	H	-2.01049	1.461291	-0.40285
C	-4.99646	0.170348	0.424209	H	-3.49637	-1.87367	-2.00545
C	-3.86095	1.207331	0.569558	H	-1.43684	1.342319	2.001012
C	-2.4632	0.661697	0.20006	H	-1.85239	-0.39153	2.017572
C	-2.43426	-0.58605	-0.66564	H	-0.96129	-1.93068	-1.56687
C	-3.55312	-1.07383	-1.27124	H	-4.07872	-1.40481	1.65612
C	-1.52304	0.444589	1.385013	H	-5.19519	-0.31572	2.521443
O	-0.18394	0.166267	0.933396	H	-5.831	-1.55954	1.429028
C	-0.06094	-0.7372	-0.07475	H	-6.72734	0.466109	-0.32722
C	-1.12283	-1.14805	-0.83259	H	-5.08907	2.405535	-0.2621
O	-5.89658	-0.78839	-1.56396	H	1.43883	-1.91161	-1.11235
C	-5.01628	-0.84406	1.57904	H	2.192461	-0.23211	1.336187
O	-6.22541	0.887706	0.405453	H	4.325323	0.890815	1.539715
O	-4.1191	2.2945	-0.30163	H	2.847998	1.811094	-0.18496
C	1.290114	-1.23097	-0.28005	H	3.882515	3.018994	0.557022
C	2.339298	-0.89621	0.48863	H	3.651125	3.379229	-1.90487
C	3.742247	-1.40847	0.251873	H	5.340814	3.073776	-1.48847
C	4.653743	-0.2425	-0.24073	H	4.373642	1.794932	-2.23183
C	4.75405	1.056857	0.544225	H	5.370438	-2.31041	1.384976
C	3.885832	2.162342	-0.13014	H	3.749	-2.86373	1.846321
C	4.343173	2.623168	-1.51778	H	4.321615	-1.26966	2.38363
C	4.338399	-1.98857	1.557964	H	4.398652	-2.10677	-1.40385
O	3.747051	-2.4118	-0.73824	H	6.287354	2.447674	1.208884
O	5.280388	-0.42165	-1.27388	H	6.777533	0.747852	1.311568
C	6.224856	1.47564	0.707268	H	6.72161	1.549556	-0.26305
H	-3.85322	1.556055	1.618459				

11*R*-**5** (conformer 4)							
Atomic Type	Standard Orientation (Å)			Atomic Type	Standard Orientation (Å)		
	X	Y	Z		X	Y	Z
C	4.827466	0.06903	1.284747	H	2.165702	1.454051	-0.61371
C	5.112858	-0.10616	-0.22144	H	3.286843	-0.33624	2.769399
C	4.06731	0.66533	-1.05658	H	1.779501	-0.03805	-2.54813
C	2.610002	0.449135	-0.59018	H	2.078606	-1.46077	-1.51598
C	2.426323	-0.05917	0.829316	H	0.796252	-0.60836	2.182572
C	3.457032	-0.11467	1.718682	H	4.187853	-2.09427	-0.40361
C	1.756093	-0.41027	-1.52174	H	5.440787	-1.7239	-1.61773
O	0.369253	-0.36462	-1.13536	H	5.90231	-2.10637	0.051201
C	0.104071	-0.50526	0.188829	H	6.796155	0.55202	0.396771
C	1.069902	-0.4047	1.152264	H	5.304533	2.112124	-0.94592
O	5.784293	0.260495	2.036405	H	-1.54237	-0.84429	1.552358
C	5.152883	-1.60331	-0.56808	H	-1.99707	-0.88576	-1.48523
O	6.383491	0.474716	-0.49209	H	-3.86583	1.125539	1.095569
O	4.329251	2.05351	-0.9487	H	-5.84909	2.661076	1.142193
C	-1.28972	-0.77782	0.499109	H	-6.49794	1.85156	-0.28212
C	-2.24656	-0.94685	-0.42919	H	-7.57687	1.033248	1.84928
C	-3.69566	-1.21372	-0.12093	H	-6.76007	-0.28006	1.006107
C	-4.49764	0.027859	-0.63895	H	-6.00336	0.469038	2.431347
C	-4.46605	1.307905	0.198909	H	-5.24015	-2.65785	-0.62914
C	-5.90002	1.694762	0.624071	H	-3.60355	-3.34768	-0.45335
C	-6.59381	0.671002	1.529564	H	-4.05181	-2.41375	-1.90704
C	-4.17393	-2.49158	-0.82461	H	-4.75042	-1.58787	1.482894
O	-3.83549	-1.32567	1.295859	H	-3.80867	3.367041	-0.06191
O	-5.10877	-0.03091	-1.68903	H	-2.78163	2.187756	-0.89466
C	-3.8168	2.431436	-0.63215	H	-4.37624	2.592746	-1.55921
H	4.167819	0.341228	-2.10852				

11*R*-**5** (conformer 5)							
Atomic Type	Standard Orientation (Å)			Atomic Type	Standard Orientation (Å)		
	X	Y	Z		X	Y	Z
C	-4.72903	0.045643	1.236332	H	-2.57223	-1.57379	-1.07693
C	-4.83911	0.723062	-0.14542	H	-3.24353	-0.53909	2.7174
C	-4.06393	-0.0926	-1.20361	H	-1.57922	0.177464	-2.5122
C	-2.65279	-0.5281	-0.74869	H	-1.45276	1.265999	-1.1058
C	-2.40232	-0.53664	0.749251	H	-0.7776	-0.9976	2.140733
C	-3.40879	-0.38278	1.654207	H	-3.28659	2.240432	0.215629
C	-1.50076	0.215126	-1.42373	H	-4.5082	2.677655	-1.00809
O	-0.2366	-0.40365	-1.11584	H	-4.92225	2.7105	0.715824
C	-0.03015	-0.75367	0.17813	H	-6.68307	0.5373	0.318563
C	-1.03344	-0.78652	1.107205	H	-5.72126	-0.99867	-1.46735
O	-5.74035	-0.00806	1.937053	H	1.518469	-1.39132	1.528151
C	-4.34775	2.176496	-0.04773	H	2.154982	-0.76461	-1.41594
O	-6.21044	0.714038	-0.52494	H	3.613266	0.687078	1.540974
O	-4.78542	-1.27857	-1.48594	H	2.771669	1.807105	-0.45923
C	1.350815	-1.08202	0.500167	H	3.325397	2.922661	0.775095
C	2.360735	-1.04712	-0.38668	H	3.962457	3.687806	-1.51863
C	3.805562	-1.40508	-0.12776	H	5.407778	3.406875	-0.54233
C	4.682062	-0.11142	-0.12105	H	4.930794	2.224039	-1.76513
C	4.355477	1.040061	0.815838	H	5.113921	-2.40636	1.275476
C	3.674259	2.198355	0.026823	H	3.480513	-3.08717	1.186657
C	4.547606	2.914657	-1.0082	H	3.7647	-1.57474	2.080532
C	4.052068	-2.15459	1.197482	H	5.05317	-1.78229	-1.53495
O	4.238382	-2.21567	-1.20386	H	5.389718	2.373605	2.187396
O	5.636963	-0.0794	-0.88182	H	5.988017	0.706429	2.231041
C	5.615303	1.496846	1.570166	H	6.417372	1.755679	0.874559
H	-3.98083	0.527106	-2.11506				

11*R*-**5** (conformer 6)							
Atomic Type	Standard Orientation (Å)			Atomic Type	Standard Orientation (Å)		
	X	Y	Z		X	Y	Z
C	4.826514	0.653861	1.134775	H	2.142862	1.077706	-1.1508
C	5.131523	-0.10132	-0.17574	H	3.284661	0.829737	2.662296
C	4.071314	0.237388	-1.2467	H	1.819694	-1.07553	-2.324
C	2.616787	0.180797	-0.72804	H	2.151417	-1.95564	-0.80927
C	2.434829	0.278347	0.776772	H	0.80876	0.267667	2.241548
C	3.458192	0.616545	1.610391	H	4.268526	-2.0237	0.457394
C	1.79782	-1.00595	-1.2344	H	5.521665	-2.1319	-0.80714
O	0.406595	-0.85274	-0.8941	H	5.978013	-1.79847	0.873696
C	0.132948	-0.45825	0.375623	H	6.787974	0.802173	0.12122
C	1.086277	0.049192	1.215323	H	5.263455	1.645223	-1.7294
O	5.76999	1.160503	1.743435	H	-1.51374	-0.27867	1.768893
C	5.219939	-1.60972	0.106928	H	-1.94919	-1.51871	-1.00823
O	6.386709	0.361857	-0.66074	H	-3.90872	1.305267	0.499581
O	4.290444	1.559987	-1.70526	H	-6.7898	0.960827	-0.46058
C	-1.2553	-0.62521	0.773488	H	-6.19536	0.499513	1.128874
C	-2.20046	-1.17137	-0.00972	H	-7.33054	2.67483	1.307152
C	-3.64619	-1.33014	0.384038	H	-5.60373	2.905637	1.624751
C	-4.4503	-0.43781	-0.62025	H	-6.29494	3.385304	0.066019
C	-4.63211	1.04018	-0.27871	H	-5.14397	-2.90044	0.531746
C	-6.05551	1.221482	0.312571	H	-3.48441	-3.41618	0.939798
C	-6.33348	2.627218	0.854923	H	-3.97063	-3.15602	-0.75856
C	-4.08408	-2.79728	0.266131	H	-4.65933	-1.16052	2.046919
O	-3.79974	-0.85266	1.721127	H	-4.52317	2.961139	-1.3098
O	-4.89353	-0.92527	-1.64287	H	-3.37865	1.757438	-1.91364
C	-4.3958	1.897259	-1.53145	H	-5.09364	1.616308	-2.32613
H	4.191631	-0.4778	-2.08079				

11*R*-**5** (conformer 7)							
Atomic Type	Standard Orientation (Å)			Atomic Type	Standard Orientation (Å)		
	X	Y	Z		X	Y	Z
C	-4.06627	1.52534	0.517904	H	-2.61776	-1.67584	1.080527
C	-4.82473	0.453874	-0.2932	H	-2.05171	2.271729	0.863069
C	-4.33684	-0.95769	0.099489	H	-2.63467	-2.86991	-1.08993
C	-2.79929	-1.09244	0.166978	H	-2.22786	-1.34984	-1.92909
C	-2.0171	0.199466	0.326739	H	0.057229	0.8678	0.46597
C	-2.62755	1.386254	0.605039	H	-3.62254	0.655932	-2.12481
C	-2.16814	-1.88953	-0.97353	H	-5.27305	0.024071	-2.36431
O	-0.78011	-2.16234	-0.70214	H	-5.0185	1.745496	-2.02263
C	-0.03731	-1.13263	-0.22047	H	-6.27588	1.422397	0.483216
C	-0.59462	0.036718	0.219537	H	-5.72966	-0.88549	1.399754
O	-4.71034	2.478015	0.960633	H	1.676688	-2.25651	-0.93044
C	-4.66414	0.73493	-1.79586	H	2.073758	-0.04616	1.157615
O	-6.20138	0.53921	0.057919	H	3.930741	1.402922	1.233849
O	-4.83128	-1.26942	1.39043	H	4.525975	3.468604	-0.095
C	1.39087	-1.43755	-0.27703	H	4.979047	2.433613	-1.44689
C	2.340578	-0.82391	0.444954	H	2.673899	3.352791	-1.7642
C	3.814037	-1.1817	0.355779	H	2.591942	1.594934	-1.60052
C	4.569437	0.020041	-0.28832	H	2.14801	2.636025	-0.23621
C	4.642787	1.379278	0.39942	H	5.446244	-1.71067	1.695224
C	4.30131	2.514841	-0.58937	H	3.86733	-2.37881	2.147725
C	2.848002	2.520807	-1.07342	H	4.219712	-0.66733	2.464302
C	4.376065	-1.49125	1.760554	H	4.517859	-1.97686	-1.23641
O	4.011201	-2.30843	-0.46565	H	6.15042	2.530532	1.466694
O	5.124631	-0.17333	-1.35839	H	6.324992	0.782896	1.694522
C	6.069073	1.559691	0.966618	H	6.804566	1.524271	0.156598
H	-4.73573	-1.67359	-0.64207				

Table S3. Energy analyses of 11*S*-**5** (six conformers).

No.	**3D conformers** B3LYP/6-31G(d,p)	**E (Hartree)**	**ΔE (kJ/mol)**	**Boltzmann distribution**
11*S*-**5** (conformer 1)	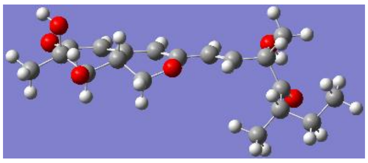	-1190.605318	0	80.65%
11*S*-**5** (conformer 2)	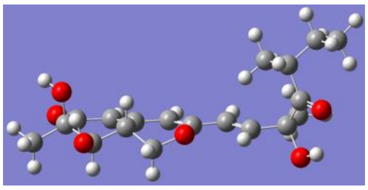	-1190.602291	7.947389105	3.27%
11*S*-**5** (conformer 3)	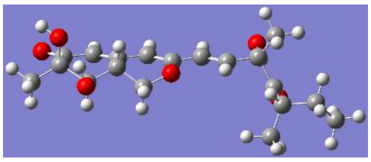	-1190.603371	5.111848889	10.26%
11*S*-**5** (conformer 4)	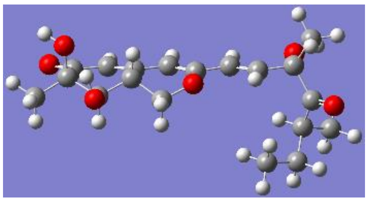	-1190.601713	9.464928221	1.77%
11*S*-**5** (conformer 5)	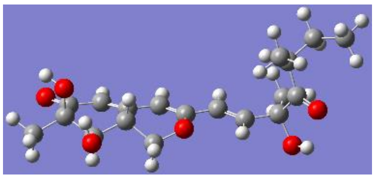	-1190.600630	12.30834494	0.56%
11*S*-**5** (conformer 6)	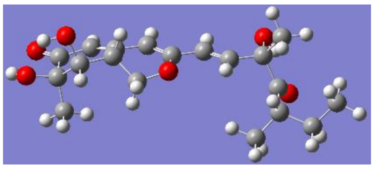	-1190.602274	7.992022609	3.21%

Table S4. Cartesian coordinates of the low-energy conformers of 11*S*-**5** re-optimized at B3LYP/6-31G(d,p) level.

11*S*-**5** (conformer 1)							
Atomic Type	Standard Orientation (Å)			Atomic Type	Standard Orientation (Å)		
	X	Y	Z		X	Y	Z
C	5.022538	-1.09197	-0.51191	H	2.84945	0.268739	1.470327
C	5.243301	0.352633	0.004324	H	3.562018	-2.55431	-1.1557
C	3.979808	1.226117	-0.08449	H	1.493395	2.213259	0.808801
C	2.7289	0.470566	0.396427	H	1.389823	1.611954	-0.86565
C	2.56899	-0.85824	-0.31469	H	1.047863	-2.32598	-0.86058
C	3.664094	-1.56463	-0.71685	H	6.235662	1.095228	-1.78339
C	1.475162	1.316443	0.190336	H	6.606834	1.999694	-0.29782
O	0.272241	0.612787	0.550013	H	7.322447	0.383456	-0.58613
C	0.152732	-0.64025	0.043729	H	6.274348	-0.33036	1.524132
C	1.224183	-1.34213	-0.43772	H	4.613614	2.179898	1.459397
O	6.009992	-1.80664	-0.68018	H	-1.31987	-2.19588	-0.29968
C	6.429464	1.002426	-0.70856	H	-2.1445	0.489984	0.921263
O	5.503348	0.25222	1.422445	H	-4.31264	1.542924	0.542231
O	4.157239	2.436941	0.637697	H	-6.19023	2.695559	-0.66849
C	-1.19564	-1.18134	0.065739	H	-6.56052	1.187453	-1.50077
C	-2.26607	-0.51704	0.530914	H	-8.10085	1.562637	0.447496
C	-3.6596	-1.10618	0.572117	H	-7.14454	0.098997	0.692595
C	-4.57097	-0.35095	-0.44224	H	-6.72158	1.59041	1.554687
C	-4.70261	1.167429	-0.41211	H	-5.28101	-1.36448	2.005542
C	-6.17469	1.603169	-0.56291	H	-3.65227	-1.58601	2.67415
C	-7.08236	1.189241	0.599569	H	-4.2596	0.053453	2.357919
C	-4.25663	-0.97893	1.994057	H	-4.26245	-2.5371	-0.54692
O	-3.64029	-2.46799	0.207044	H	-3.90131	2.840619	-1.55105
O	-5.15782	-1.02012	-1.27816	H	-2.78573	1.465026	-1.45601
C	-3.83858	1.747148	-1.55584	H	-4.2002	1.383351	-2.52317
H	3.832019	1.512773	-1.13344				

11*S*-**5** (conformer 2)							
Atomic Type	Standard Orientation (Å)			Atomic Type	Standard Orientation (Å)		
	X	Y	Z		X	Y	Z
C	-4.70214	1.295087	-0.74248	H	-2.48479	0.088853	1.290654
C	-5.10443	0.212106	0.290547	H	-3.14363	2.010348	-2.06387
C	-4.10917	-0.95957	0.356532	H	-1.75274	-2.25995	1.208501
C	-2.65073	-0.46907	0.358018	H	-1.89448	-2.27669	-0.5678
C	-2.37333	0.465841	-0.80231	H	-0.73769	1.195815	-2.04992
C	-3.35362	1.281939	-1.28418	H	-6.60815	-0.75372	-0.94925
C	-1.68306	-1.6487	0.3095	H	-6.81997	-0.99648	0.799514
O	-0.30907	-1.22474	0.226721	H	-7.22058	0.575365	0.040377
C	-0.04233	-0.28985	-0.71662	H	-5.60477	1.596401	1.58431
C	-1.01812	0.491489	-1.27301	H	-4.57034	-1.15893	2.212104
O	-5.52615	2.162739	-1.02856	H	1.596576	0.636878	-1.76132
C	-6.53094	-0.27314	0.032786	H	2.055801	-1.79414	0.06746
O	-5.01055	0.827684	1.594992	H	3.569088	1.505987	0.322703
O	-4.37578	-1.77885	1.485924	H	5.144911	2.626513	1.938955
C	1.363772	-0.16928	-1.07108	H	6.104226	1.150291	2.002999
C	2.327587	-0.97865	-0.59792	H	7.123021	2.837331	0.447468
C	3.804662	-0.90711	-0.90823	H	6.659353	1.384516	-0.44202
C	4.574122	-0.38955	0.349721	H	5.654079	2.842865	-0.53774
C	4.20649	0.933588	1.007655	H	5.239004	-0.05532	-2.28638
C	5.464812	1.756599	1.35104	H	3.653555	-0.39705	-2.9989
C	6.266872	2.22979	0.1347	H	3.873848	1.035969	-1.96656
C	4.160422	-0.01044	-2.10996	H	4.992432	-2.3714	-0.55227
O	4.247069	-2.22576	-1.17237	H	3.10646	1.560902	2.776464
O	5.467945	-1.09479	0.790096	H	2.468851	0.075024	2.05331
C	3.387209	0.624652	2.282139	H	3.982596	0.028863	2.981862
H	-4.263	-1.59308	-0.52615				

11*S*-**5** (conformer 3)							
Atomic Type	Standard Orientation (Å)			Atomic Type	Standard Orientation (Å)		
	X	Y	Z		X	Y	Z
C	5.045204	-1.05511	-0.6303	H	2.927607	0.213318	1.470548
C	5.280644	0.364662	-0.05515	H	3.566806	-2.48828	-1.29855
C	4.015632	1.240368	-0.06995	H	1.554858	2.18444	0.934423
C	2.77797	0.463089	0.410474	H	1.404879	1.658882	-0.76187
C	2.598117	-0.83263	-0.35487	H	1.061944	-2.27541	-0.92474
C	3.681352	-1.51934	-0.81842	H	6.224717	1.188762	-1.83323
C	1.519318	1.316321	0.276998	H	6.636521	2.024597	-0.3185
O	0.326706	0.595977	0.637796	H	7.343069	0.423684	-0.6996
C	0.192559	-0.63239	0.078221	H	6.352522	-0.3854	1.403685
C	1.250208	-1.31164	-0.46263	H	4.691344	2.123961	1.497793
O	6.027227	-1.76052	-0.85853	H	-1.28597	-2.17774	-0.28375
C	6.447458	1.047032	-0.76942	H	-2.08673	0.465752	1.03963
O	5.578782	0.199885	1.349426	H	-4.10413	1.515258	0.668481
O	4.213459	2.417546	0.700949	H	-6.39501	0.854345	1.256435
C	-1.15492	-1.17515	0.111427	H	-6.27914	2.474528	0.596958
C	-2.21552	-0.52964	0.622707	H	-8.30216	1.277801	-0.24189
C	-3.60948	-1.12738	0.669148	H	-7.23588	1.690529	-1.5887
C	-4.49761	-0.33061	-0.33329	H	-7.28778	0.029507	-0.9884
C	-4.75348	1.157208	-0.13872	H	-5.21088	-1.43897	2.110439
C	-6.21731	1.411679	0.32748	H	-3.5751	-1.67321	2.754487
C	-7.31934	1.07931	-0.68394	H	-4.18844	-0.02765	2.493666
C	-4.18832	-1.04853	2.098486	H	-4.12158	-2.49481	-0.56741
O	-3.59737	-2.47639	0.260719	H	-4.63743	2.987612	-1.30895
O	-4.96793	-0.94535	-1.2789	H	-3.32705	1.841423	-1.64528
C	-4.39666	1.924882	-1.42418	H	-4.94828	1.531113	-2.28145
H	3.839387	1.573965	-1.10047				

11*S*-**5** (conformer 4)							
Atomic Type	Standard Orientation (Å)			Atomic Type	Standard Orientation (Å)		
	X	Y	Z		X	Y	Z
C	-4.75137	-0.80646	1.164359	H	-2.85151	-0.51481	-1.44634
C	-5.11268	0.210401	0.052023	H	-3.14538	-1.75633	2.261566
C	-3.90885	1.0457	-0.41768	H	-1.57805	1.535202	-1.9319
C	-2.65429	0.177195	-0.61519	H	-1.29172	1.807898	-0.19437
C	-2.34507	-0.64861	0.617543	H	-0.68728	-1.64795	1.626799
C	-3.35146	-1.09832	1.420662	H	-6.00175	1.697331	1.367406
C	-1.44767	1.042917	-0.96882	H	-6.55636	1.772499	-0.32051
O	-0.23549	0.274456	-1.0795	H	-7.13274	0.476976	0.773591
C	0.011146	-0.58081	-0.05782	H	-6.22021	-1.13405	-0.84541
C	-0.9651	-0.98946	0.809976	H	-4.73132	1.134206	-2.15295
O	-5.66966	-1.37177	1.757224	H	1.599511	-1.76916	0.810446
C	-6.27734	1.097358	0.492495	H	2.160969	0.086855	-1.57292
O	-5.48185	-0.56178	-1.11272	H	4.183524	0.013363	1.669551
O	-4.22917	1.761869	-1.60216	H	5.249098	2.235237	2.283758
C	1.38651	-1.04219	0.033287	H	5.581686	2.51128	0.574554
C	2.367037	-0.64045	-0.79253	H	3.505231	3.718717	1.312813
C	3.793886	-1.12059	-0.71844	H	3.11515	2.516135	0.078257
C	4.644271	0.159229	-0.418	H	2.762635	2.181262	1.782319
C	4.93151	0.510291	1.042319	H	5.270671	-2.07966	-1.99733
C	4.881638	2.032347	1.269004	H	3.588106	-2.60193	-2.2852
C	3.486995	2.643902	1.100793	H	4.163171	-1.01273	-2.86147
C	4.226698	-1.74446	-2.05407	H	4.711374	-2.56944	0.219587
O	3.893375	-2.06551	0.347135	H	6.557912	0.173311	2.453206
O	5.043893	0.845183	-1.34012	H	6.356964	-1.14803	1.296339
C	6.320191	-0.05903	1.409421	H	7.09798	0.379658	0.774577
H	-3.69291	1.800732	0.348836				

11*S*-**5** (conformer 5)							
Atomic Type	Standard Orientation (Å)			Atomic Type	Standard Orientation (Å)		
	X	Y	Z		X	Y	Z
C	-4.76636	1.042563	-1.03502	H	-2.51517	0.471123	1.225894
C	-5.15169	0.310235	0.275295	H	-3.23015	1.332613	-2.53258
C	-4.15551	-0.79598	0.664846	H	-1.78484	-1.80278	1.821659
C	-2.6971	-0.33395	0.499686	H	-1.95819	-2.33744	0.130627
C	-2.43922	0.219684	-0.8875	H	-0.82516	0.544303	-2.32057
C	-3.42717	0.864772	-1.57086	H	-6.67561	-0.96809	-0.60505
C	-1.73079	-1.48153	0.782162	H	-6.85847	-0.68922	1.141622
O	-0.35828	-1.1085	0.556008	H	-7.27172	0.59475	-0.03656
C	-0.1075	-0.49075	-0.6226	H	-5.63077	2.014439	1.11578
C	-1.0924	0.099125	-1.36732	H	-4.58564	-0.44265	2.504919
O	-5.59474	1.793277	-1.54872	H	1.506298	0.061522	-1.93564
C	-6.58224	-0.22235	0.192754	H	2.017081	-1.67193	0.554317
O	-5.03667	1.279408	1.341395	H	3.436661	1.457548	-0.25015
O	-4.40344	-1.2485	1.988284	H	5.717603	1.659187	-1.16022
C	1.291588	-0.48789	-1.02302	H	5.219467	3.042369	-0.2051
C	2.269125	-1.11232	-0.34306	H	7.606172	2.43955	0.21256
C	3.74076	-1.16031	-0.70077	H	6.668778	2.290265	1.702615
C	4.502974	-0.2878	0.344862	H	7.125467	0.838667	0.804687
C	4.277715	1.214854	0.408916	H	5.140819	-0.79753	-2.30728
C	5.516067	1.987414	-0.13208	H	3.542175	-1.3405	-2.84027
C	6.800058	1.877149	0.696982	H	3.776278	0.343209	-2.31868
C	4.066407	-0.69481	-2.12956	H	4.813216	-2.47665	0.193061
O	4.176853	-2.49865	-0.55263	H	3.800279	2.717954	1.906931
O	5.267214	-0.86313	1.105118	H	2.942043	1.184603	2.137098
C	3.899094	1.628562	1.842493	H	4.657097	1.302293	2.559126
H	-4.3241	-1.6589	0.008244				

11*S*-**5** (conformer 6)							
Atomic Type	Standard Orientation (Å)			Atomic Type	Standard Orientation (Å)		
	X	Y	Z		X	Y	Z
C	4.987943	-0.72918	-0.77109	H	2.572421	-0.35803	1.80291
C	5.004003	0.721281	-0.24488	H	3.615252	-2.22219	-1.56409
C	4.104179	0.846905	1.004333	H	1.53023	1.868049	1.527213
C	2.722845	0.171173	0.851571	H	1.568663	1.671146	-0.2444
C	2.604272	-0.88023	-0.23814	H	1.088502	-2.22178	-1.07071
C	3.69295	-1.36725	-0.89697	H	3.554758	1.51212	-1.69918
C	1.546584	1.137025	0.715924	H	4.681851	2.714765	-1.01734
O	0.291285	0.437803	0.811899	H	5.246087	1.552526	-2.23205
C	0.183113	-0.71532	0.10085	H	6.871253	0.320821	-0.27508
C	1.262918	-1.34891	-0.44987	H	5.693489	0.432893	1.973116
O	6.055178	-1.22281	-1.13785	H	-1.2971	-2.14871	-0.57569
C	4.585096	1.682243	-1.36922	H	-2.13903	0.306648	1.051265
O	6.335051	1.020764	0.159717	H	-4.27007	1.458336	0.769869
O	4.748549	0.218107	2.098153	H	-6.071	2.84822	-0.30075
C	-1.17467	-1.2152	-0.03554	H	-6.42504	1.517014	-1.39929
C	-2.25615	-0.61467	0.486783	H	-8.04851	1.582161	0.515976
C	-3.6587	-1.16959	0.361523	H	-7.12639	0.076928	0.542849
C	-4.51049	-0.22766	-0.54256	H	-6.72214	1.38581	1.669704
C	-4.6204	1.26464	-0.25171	H	-5.34985	-1.638	1.654025
C	-6.07701	1.754069	-0.38742	H	-3.75827	-2.01079	2.344405
C	-7.04386	1.165046	0.64447	H	-4.3255	-0.32771	2.295699
C	-4.3205	-1.28004	1.756033	H	-4.23087	-2.36907	-1.01586
O	-3.64148	-2.44605	-0.23675	H	-3.74025	3.091891	-1.04245
O	-5.06838	-0.72681	-1.5071	H	-2.65236	1.694515	-1.14329
C	-3.6948	2.014991	-1.23706	H	-4.01661	1.835191	-2.26804
H	3.964884	1.923225	1.214358				

Table S5. ^{1}H NMR data of the 3,5-dimethylhept-1-ene-3,4-diol moiety of know compounds in $CDCl_3$.

	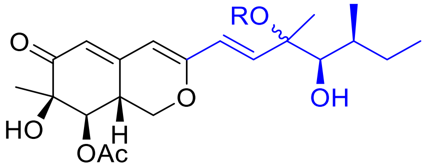			
	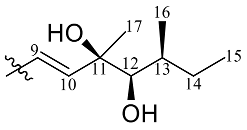	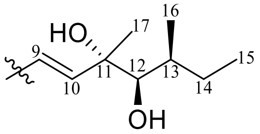	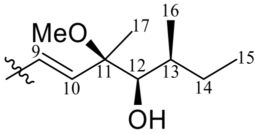	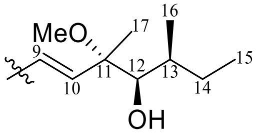
	**geumsanol D**[19]	***epi*-geumsanol D (20)**[19]	**penicilazaphilone I**[20]	**penicilazaphilone J**[20]
no.	δ_H (mult, J in Hz)	δ_H (mult, J in Hz)	δ_H (mult, J in Hz)	δ_H (mult, J in Hz)
9	6.16, d (15.4)	6.20, d (15.5)	6.00, d (16.0)	5.98, d (16.1)
10	6.44, d (15.4)	6.43, d (15.5)	6.31, d (16.0)	6.41, d (16.1)
12	3.47, brs	3.50, brs	3.34, brs	3.45, brs
13	1.68, m	1.63, m	1.52, m	1.47, m
14a	1.42, m	1.41, m	1.42, m	1.41, m
14b	1.31, m	1.31, m	1.24, m	1.25, m
15	0.91, t (7.4)	0.90, t (7.4)	0.85, t (7.4)	0.87, t (7.4)
16	0.98, d (6.7)	0.86, d (6.8)	0.90, d (6.7)	0.83, d (6.7)
17	1.30, s	1.36, s	1.29, s	1.31, s
11-OMe			3.15, s	3.17, s
Data were measured in $CDCl_3$. Chemical shifts are in ppm; J values in Hz are in parentheses.				

Table S6. Crystal data and experimental details for **1**.

	Crystal data	
Empirical formula	$C_{17} H_{26} O_4$	
Formula weight	294.38	
Crystal system	Monoclinic	
Space group	$P2_1$	
Unit cell dimensions	a = 14.8068(10) Å	α= 90°.
	b = 6.6748(5) Å	β= 101.424(3)°.
	c = 17.6717(13) Å	γ = 90°.
Volume	1711.9(2) Å ^{3}	
Z	4	
F(000)	640	
Density (calculated)	1.142 Mg/m^3	
Wavelength	1.54178 Å	
Cell parameters reflections used	9917	
Theta range for Cell parameters	2.55 to 69.66°.	
Absorption coefficient	0.645 mm^{-1}	
Temperature	100(2) K	
Crystal size	0.150 x 0.100 x 0.010 mm^3	
	Data collection	
Diffractometer	Bruker AXS D8 VENTURE, PhotonIII_C28	
Absorption correction	Semi-empirical from equivalents	
Max. and min. transmission	1.0000 and 0.8303	
No. of measured reflections	29571	
No. of independent reflections	6310 [R(int) = 0.0643]	
No. of observed [I>2_igma(I)]	5435	
Completeness to theta = 67.679°	99.8 %	
Theta range for data collection	2.551 to 70.110°.	
	Refinement	
Final R indices [I>2sigma(I)]	R1 = 0.0582, wR2 = 0.1532	
R indices (all data)	R1 = 0.0696, wR2 = 0.1663	
Goodness-of-fit on F^2	1.085	
No. of reflections	6310	
No. of parameters	403	
No. of restraints	1	
Absolute structure parameter	-0.1(2)	
Largest diff. peak and hole	0.208 and -0.291 e.Å $^{-3}$	

Table S7. Bond lengths [Å] and angles [°] for **1**.

O(1)-C(5)	1.372(4)
O(1)-C(1)	1.376(4)
O(2)-C(1)	1.237(4)
O(3)-C(3)	1.337(4)
O(4)-C(7)	1.429(5)
C(1)-C(2)	1.413(5)
C(2)-C(3)	1.380(4)
C(2)-C(17)	1.507(5)
C(3)-C(4)	1.435(5)
C(4)-C(5)	1.335(5)
C(5)-C(6)	1.491(5)
C(6)-C(7)	1.532(6)
C(7)-C(8)	1.529(5)
C(8)-C(9)	1.531(6)
C(9)-C(10)	1.508(6)
C(10)-C(11)	1.325(7)
C(10)-C(16)	1.514(6)
C(11)-C(12)	1.514(7)
C(12)-C(13)	1.508(8)
C(12)-C(15)	1.525(8)
C(13)-C(14)	1.491(10)
O(5)-C(22)	1.371(4)
O(5)-C(18)	1.389(4)
O(6)-C(18)	1.232(4)
O(7)-C(20)	1.346(4)
O(8)-C(24)	1.428(5)
C(18)-C(19)	1.421(5)
C(19)-C(20)	1.368(4)
C(19)-C(34)	1.502(4)
C(20)-C(21)	1.435(4)
C(21)-C(22)	1.335(5)
C(22)-C(23)	1.493(5)
C(23)-C(24)	1.534(6)
C(24)-C(25)	1.526(4)
C(25)-C(26)	1.533(6)
C(26)-C(27)	1.514(5)

C(27)-C(28)	1.329(6)
C(27)-C(33)	1.501(6)
C(28)-C(29)	1.513(5)
C(29)-C(32)	1.528(8)
C(29)-C(30)	1.534(7)
C(30)-C(31)	1.536(6)

C(5)-O(1)-C(1)	121.7(2)
O(2)-C(1)-O(1)	115.0(3)
O(2)-C(1)-C(2)	125.5(3)
O(1)-C(1)-C(2)	119.5(3)
C(3)-C(2)-C(1)	117.9(3)
C(3)-C(2)-C(17)	123.3(3)
C(1)-C(2)-C(17)	118.8(3)
O(3)-C(3)-C(2)	118.7(3)
O(3)-C(3)-C(4)	120.5(3)
C(2)-C(3)-C(4)	120.8(3)
C(5)-C(4)-C(3)	119.3(3)
C(4)-C(5)-O(1)	120.7(3)
C(4)-C(5)-C(6)	127.6(3)
O(1)-C(5)-C(6)	111.6(3)
C(5)-C(6)-C(7)	111.5(3)
O(4)-C(7)-C(8)	105.9(3)
O(4)-C(7)-C(6)	110.7(3)
C(8)-C(7)-C(6)	112.7(3)
C(7)-C(8)-C(9)	111.4(3)
C(10)-C(9)-C(8)	114.9(4)
C(11)-C(10)-C(9)	120.4(4)
C(11)-C(10)-C(16)	123.8(4)
C(9)-C(10)-C(16)	115.7(4)
C(10)-C(11)-C(12)	130.3(4)
C(13)-C(12)-C(11)	112.4(4)
C(13)-C(12)-C(15)	109.0(6)
C(11)-C(12)-C(15)	109.3(4)
C(14)-C(13)-C(12)	114.7(5)
C(22)-O(5)-C(18)	121.1(2)
O(6)-C(18)-O(5)	115.1(3)
O(6)-C(18)-C(19)	125.8(3)

O(5)-C(18)-C(19)	119.1(3)
C(20)-C(19)-C(18)	118.5(3)
C(20)-C(19)-C(34)	123.8(3)
C(18)-C(19)-C(34)	117.7(3)
O(7)-C(20)-C(19)	119.2(3)
O(7)-C(20)-C(21)	120.0(3)
C(19)-C(20)-C(21)	120.8(3)
C(22)-C(21)-C(20)	119.3(3)
C(21)-C(22)-O(5)	121.2(3)
C(21)-C(22)-C(23)	126.6(3)
O(5)-C(22)-C(23)	112.2(3)
C(22)-C(23)-C(24)	114.6(3)
O(8)-C(24)-C(25)	106.8(3)
O(8)-C(24)-C(23)	111.9(3)
C(25)-C(24)-C(23)	111.0(3)
C(24)-C(25)-C(26)	113.2(3)
C(27)-C(26)-C(25)	112.4(3)
C(28)-C(27)-C(33)	125.3(3)
C(28)-C(27)-C(26)	120.1(4)
C(33)-C(27)-C(26)	114.5(4)
C(27)-C(28)-C(29)	130.3(4)
C(28)-C(29)-C(32)	109.4(4)
C(28)-C(29)-C(30)	109.4(4)
C(32)-C(29)-C(30)	111.6(4)
C(29)-C(30)-C(31)	114.4(4)

Symmetry transformations used to generate equivalent atoms:

Table S8. Crystal data and experimental details for **2**.

Crystal data		
Empirical formula	$C_{19} H_{28} O_5$	
Formula weight	336.41	
Crystal system	Orthorhombic	
Space group	$P2_12_12_1$	
Unit cell dimensions	a = 5.5157(2) Å	α= 90°.
	b = 15.6768(6) Å	β= 90°.
	c = 43.0946(19) Å	γ = 90°.
Volume	3726.3(3) Å ^{3}	
Z	8	
F(000)	1456	
Density (calculated)	1.199 Mg/m^3	
Wavelength	1.54178 Å	
Cell parameters reflections used	9956	
Theta range for Cell parameters	3.00 to 70.00°.	
Absorption coefficient	0.697 mm^{-1}	
Temperature	100(2) K	
Crystal size	0.150 x 0.020 x 0.015 mm^3	
Data collection		
Diffractometer	Bruker AXS D8 VENTURE, PhotonIII_C28	
Absorption correction	Semi-empirical from equivalents	
Max. and min. transmission	1.0000 and 0.7539	
No. of measured reflections	49413	
No. of independent reflections	7089 [R(int) = 0.1421]	
No. of observed [I>2_igma(I)]	5498	
Completeness to theta = 67.679°	99.9 %	
Theta range for data collection	2.999 to 70.280°.	
Refinement		
Final R indices [I>2sigma(I)]	R1 = 0.0614, wR2 = 0.1486	
R indices (all data)	R1 = 0.0850, wR2 = 0.1652	
Goodness-of-fit on F^2	1.023	
No. of reflections	7089	
No. of parameters	587	
No. of restraints	824	
Absolute structure parameter	-0.1(2)	
Largest diff. peak and hole	0.213 and -0.317 e.Å $^{-3}$	

Table S9. Bond lengths [Å] and angles [°] for **2**.

O(1)-C(5)	1.372(5)
O(1)-C(1)	1.381(5)
O(2)-C(1)	1.230(5)
O(3)-C(3)	1.345(5)
O(4)-C(7)	1.437(5)
O(5)-C(9)	1.225(6)
C(1)-C(2)	1.423(6)
C(2)-C(3)	1.373(5)
C(2)-C(19)	1.493(6)
C(3)-C(4)	1.427(6)
C(4)-C(5)	1.327(6)
C(5)-C(6)	1.502(6)
C(6)-C(7)	1.525(5)
C(7)-C(8)	1.519(6)
C(8)-C(9)	1.513(6)
C(9)-C(10)	1.504(7)
C(10)-C(11)	1.397(11)
C(10)-C(11')	1.686(12)
C(11)-C(12)	1.524(14)
C(12)-C(13)	1.322(13)
C(12)-C(18)	1.517(13)
C(13)-C(14)	1.510(12)
C(14)-C(17)	1.511(14)
C(14)-C(15)	1.560(15)
C(15)-C(16)	1.493(17)
C(11')-C(12')	1.522(13)
C(12')-C(13')	1.318(13)
C(12')-C(18')	1.506(13)
C(13')-C(14')	1.515(13)
C(14')-C(15')	1.516(15)
C(14')-C(17')	1.550(15)
C(15')-C(16')	1.51(2)
O(6)-C(22)	1.341(5)
O(7)-C(20)	1.232(5)
O(8)-C(24)	1.368(5)
O(8)-C(20)	1.386(5)

O(9)-C(26)	1.434(5)
O(10)-C(28)	1.222(5)
C(20)-C(21)	1.421(6)
C(21)-C(22)	1.370(6)
C(21)-C(38)	1.496(6)
C(22)-C(23)	1.427(6)
C(23)-C(24)	1.338(6)
C(24)-C(25)	1.485(6)
C(25)-C(26)	1.520(5)
C(26)-C(27)	1.522(6)
C(27)-C(28)	1.520(5)
C(28)-C(29)	1.502(6)
C(29)-C(30)	1.517(6)
C(30)-C(31')	1.456(13)
C(30)-C(31)	1.587(13)
C(31)-C(32)	1.326(16)
C(31)-C(37)	1.536(14)
C(32)-C(33)	1.518(12)
C(33)-C(34)	1.488(14)
C(33)-C(36)	1.539(13)
C(34)-C(35)	1.536(15)
C(31')-C(32')	1.328(15)
C(31')-C(37')	1.514(14)
C(32')-C(33')	1.505(13)
C(33')-C(36')	1.501(16)
C(33')-C(34')	1.522(14)
C(34')-C(35')	1.541(15)

C(5)-O(1)-C(1)	121.2(3)
O(2)-C(1)-O(1)	115.1(4)
O(2)-C(1)-C(2)	125.7(4)
O(1)-C(1)-C(2)	119.2(4)
C(3)-C(2)-C(1)	118.0(4)
C(3)-C(2)-C(19)	123.6(4)
C(1)-C(2)-C(19)	118.4(4)
O(3)-C(3)-C(2)	117.7(4)
O(3)-C(3)-C(4)	121.3(3)
C(2)-C(3)-C(4)	121.0(4)

C(5)-C(4)-C(3)	119.4(3)
C(4)-C(5)-O(1)	121.2(3)
C(4)-C(5)-C(6)	127.5(3)
O(1)-C(5)-C(6)	111.3(3)
C(5)-C(6)-C(7)	113.8(3)
O(4)-C(7)-C(8)	106.1(3)
O(4)-C(7)-C(6)	110.3(3)
C(8)-C(7)-C(6)	111.9(3)
C(9)-C(8)-C(7)	115.2(3)
O(5)-C(9)-C(10)	122.2(4)
O(5)-C(9)-C(8)	121.7(4)
C(10)-C(9)-C(8)	116.1(4)
C(11)-C(10)-C(9)	126.8(6)
C(9)-C(10)-C(11')	105.6(5)
C(10)-C(11)-C(12)	118.2(9)
C(13)-C(12)-C(18)	123.4(8)
C(13)-C(12)-C(11)	121.5(8)
C(18)-C(12)-C(11)	114.8(8)
C(12)-C(13)-C(14)	129.5(9)
C(13)-C(14)-C(17)	113.4(8)
C(13)-C(14)-C(15)	108.7(8)
C(17)-C(14)-C(15)	111.5(9)
C(16)-C(15)-C(14)	115.8(11)
C(12')-C(11')-C(10)	108.3(7)
C(13')-C(12')-C(18')	124.2(8)
C(13')-C(12')-C(11')	121.1(8)
C(18')-C(12')-C(11')	114.8(8)
C(12')-C(13')-C(14')	128.7(9)
C(13')-C(14')-C(15')	112.1(9)
C(13')-C(14')-C(17')	110.5(8)
C(15')-C(14')-C(17')	109.0(9)
C(16')-C(15')-C(14')	112.6(11)
C(24)-O(8)-C(20)	120.9(3)
O(7)-C(20)-O(8)	114.2(3)
O(7)-C(20)-C(21)	126.2(4)
O(8)-C(20)-C(21)	119.6(3)
C(22)-C(21)-C(20)	118.0(4)
C(22)-C(21)-C(38)	123.8(4)

C(20)-C(21)-C(38)	118.2(4)
O(6)-C(22)-C(21)	119.6(4)
O(6)-C(22)-C(23)	119.7(4)
C(21)-C(22)-C(23)	120.7(4)
C(24)-C(23)-C(22)	119.9(4)
C(23)-C(24)-O(8)	120.7(3)
C(23)-C(24)-C(25)	126.4(4)
O(8)-C(24)-C(25)	112.9(3)
C(24)-C(25)-C(26)	115.2(3)
O(9)-C(26)-C(25)	108.0(3)
O(9)-C(26)-C(27)	111.2(3)
C(25)-C(26)-C(27)	109.0(3)
C(28)-C(27)-C(26)	117.2(3)
O(10)-C(28)-C(29)	122.3(4)
O(10)-C(28)-C(27)	121.9(4)
C(29)-C(28)-C(27)	115.7(3)
C(28)-C(29)-C(30)	115.0(4)
C(31')-C(30)-C(29)	117.8(6)
C(29)-C(30)-C(31)	111.3(5)
C(32)-C(31)-C(37)	123.1(10)
C(32)-C(31)-C(30)	121.3(9)
C(37)-C(31)-C(30)	115.5(9)
C(31)-C(32)-C(33)	130.8(9)
C(34)-C(33)-C(32)	110.3(9)
C(34)-C(33)-C(36)	109.8(9)
C(32)-C(33)-C(36)	110.5(8)
C(33)-C(34)-C(35)	115.1(11)
C(32')-C(31')-C(30)	124.7(9)
C(32')-C(31')-C(37')	124.2(10)
C(30)-C(31')-C(37')	111.1(9)
C(31')-C(32')-C(33')	127.9(9)
C(36')-C(33')-C(32')	111.3(9)
C(36')-C(33')-C(34')	112.1(11)
C(32')-C(33')-C(34')	110.4(8)
C(33')-C(34')-C(35')	115.0(9)

Symmetry transformations used to generate equivalent atoms:

Table S10 General genome sequencing data for *P. sclerotiorum*

General Features	
**Genome size**	22.6 Mb
**GC contents**	47.91%
**Number of contigs**	533
**Largest contig length**	173775
**N50**	60890
**N75**	39534
**L50**	126
**L75**	243
**Number of predicted genes**	7389

Table S11 Deduced gene functions in *Azp* cluster.

Gene	Predicted Function	Identity/Similarity (%)	Size: Gene/Protein	Blast Homologs and Origin
AzpT1	MFS	78/83	1605/534	KAJ6024617; *P. herquei*
AzpMT	Methyl transferase family protein	76/85	1632/543	KAB8225327; *Aspergillus novoparasiticus*
AzpU1	Other/Unknown function	81/89	1242/413	KAJ6024619; *P. herquei*
AzpU2	Other/Unknown function	72/83	936/311	XP_056941745; *P. malachiteum*
AzpU3	Other/Unknown function	81/88	381/126	KAJ5533582; *P. frequentans*
AzpU4	Other/Unknown function	49/49	492/163	NWI69292; *Todus mexicanus*
AzpU5	Other/Unknown function	26/36	1038/345	CAG8993149; *P. sclerotiorum*
AzpU6	Other/Unknown function	22/37	957/318	XP_040651148; *P. griseofulvum*
AzpF	Phytoene dehydrogenase-related protein	58/66	1659/552	WDW19272; *P. meliponae*
AzpU7	Other/Unknown function	56/60	831/276	WDW19273; *P. meliponae*
AzpG	Phytoene dehydrogenase-related protein	71/77	1299/432	WDW19274; *P. meliponae*
AzpU8	Other/Unknown function	84/91	654/217	OOQ88729; *P. brasilianum*
AzpA	HR polyketide synthase	91/95	7778/2585	WDW_19275; *P. meliponate*
AzpU9	Other/Unknown function	69/79	1557/518	WDW19276; *P. meliponae*
AzpH	FAD-dependent halogenase	92/95	1626/541	FixC-CAG8993148; *P. sclerotiorum*
AzpC	FAD-dependent monooxygenase	88/92	1377/458	WDW19278; *P. meliponae*
AzpT2	Putative Zn(II)$_2$Cys$_6$ transcription factor	55/56	1083/360	WDW19279; *P. meliponae*
AzpE	Enoyl reductase-like protein	92/97	1164/387	WDW19280; *P. meliponae*
AzpT3	MFS general substrate transporter	84/87	1899/632	WDW19281; *P. meliponae*
AzpD	3-*O*-acetyltransferase	70/74	1143/380	WDW19282; *P. meliponae*
AzpU10	Other/Unknown function	45/56	2844/947	XP_057071918; *P. verrucosum*
AzpB1	NR polyketide synthase	47/49	4176/1391	CAG8993142; *P. sclerotiorum*
AzpB2	NR polyketide synthase	39/41	3609/1202	CAG8993142;*P. sclerotiorum* (Psc_Aza_B)
AzpL	FAD-oxidoredutase	69/75	1212/403	GlcD-CAG8993145;*P. sclerotiorum*
AzpU11	Other/Unknown function	92/96	1236/411	WDW19286; *P. meliponae*

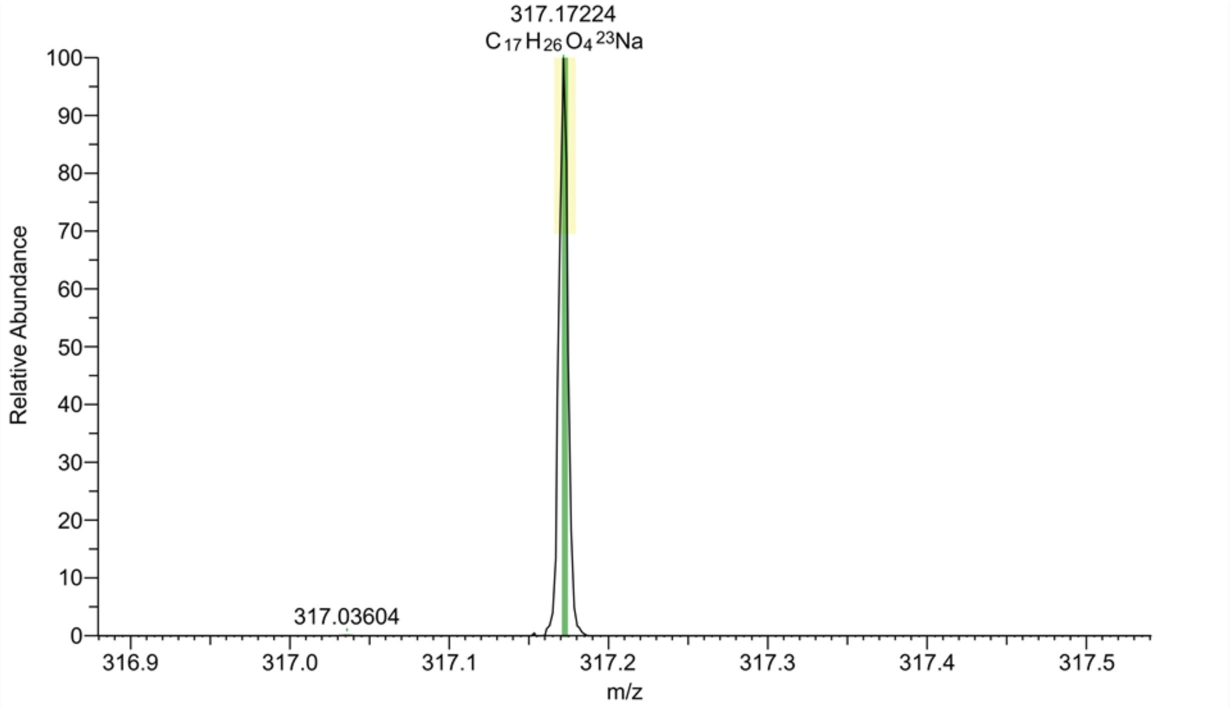

Peak Mass	Display Formula	S Fit	RDB	Delta [ppm]	Theo. mass	Pattern Cov. [%]	MSMS Matched...
317.17224	$C_{17}H_{26}O_4{}^{23}Na$	83.43723931006 22	4.50	-0.28	317.17233	100	(Collection)

Figure S1. HRESIMS spectrum of sclerolactone A (**1**)

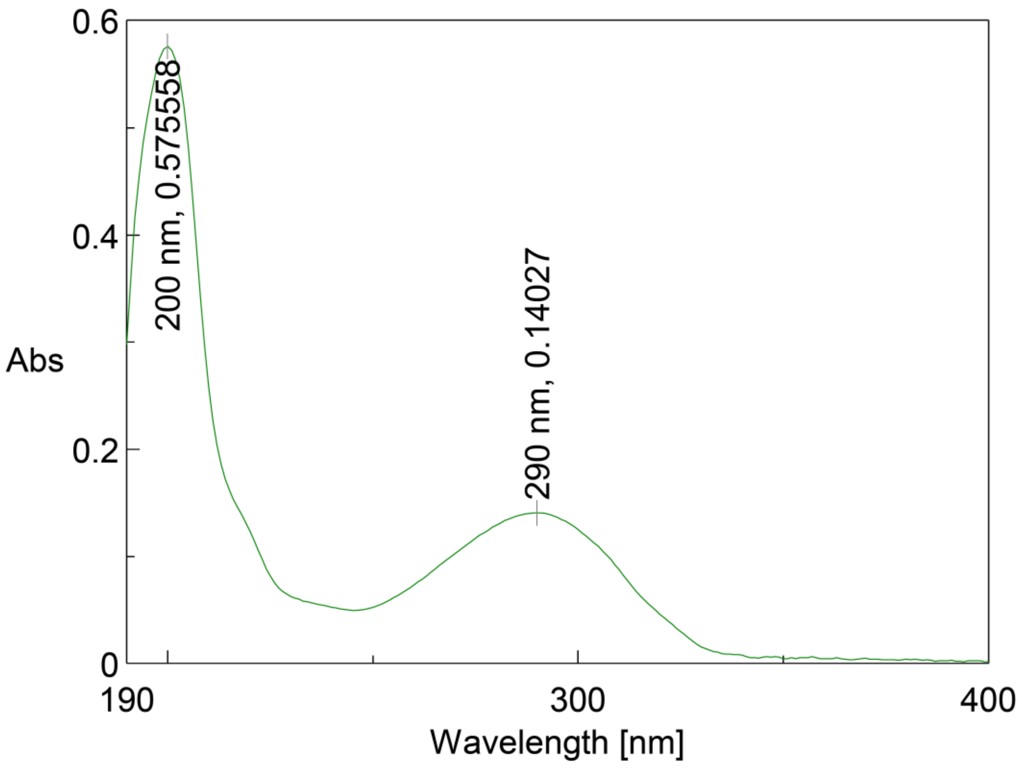

Figure S2. UV spectrum of sclerolactone A (**1**)

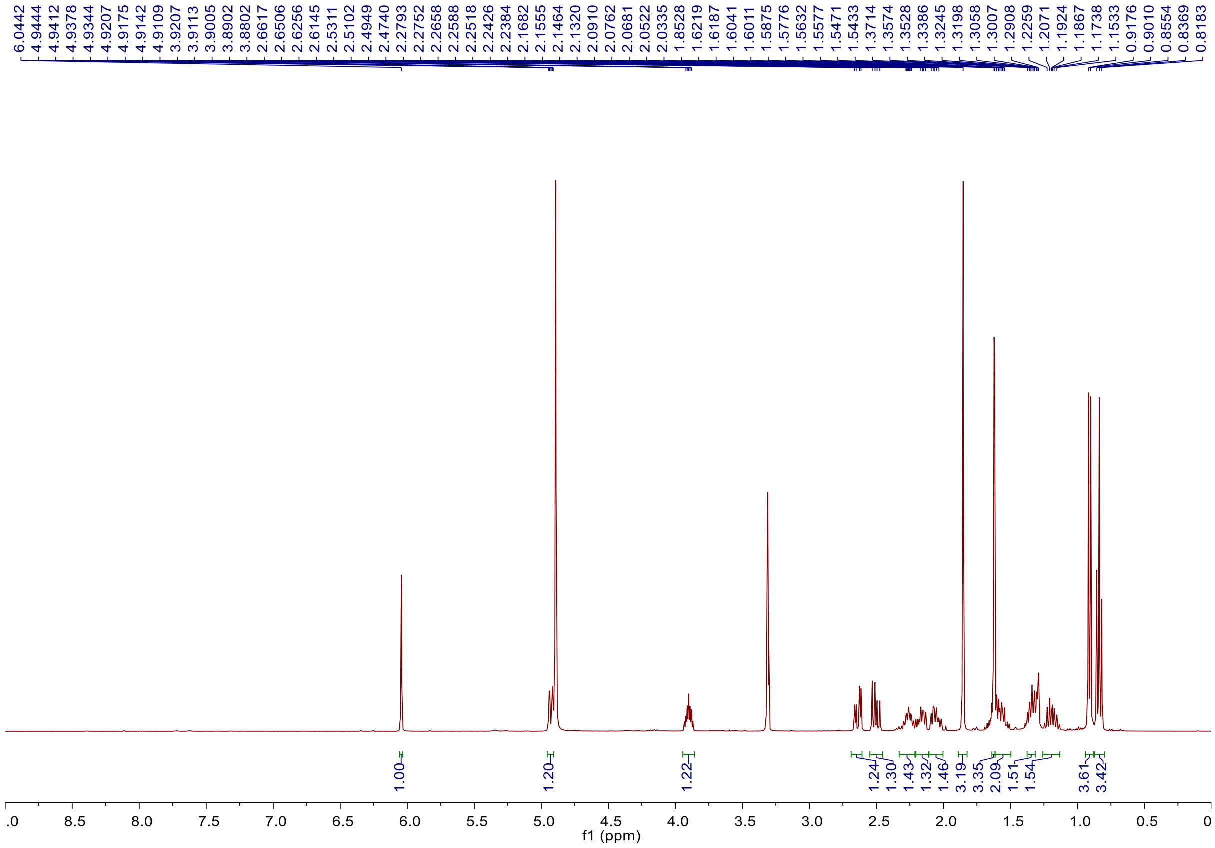

Figure S3. ^{1}H NMR spectrum (400 MHz, CD_3OD) of sclerolactone A (**1**)

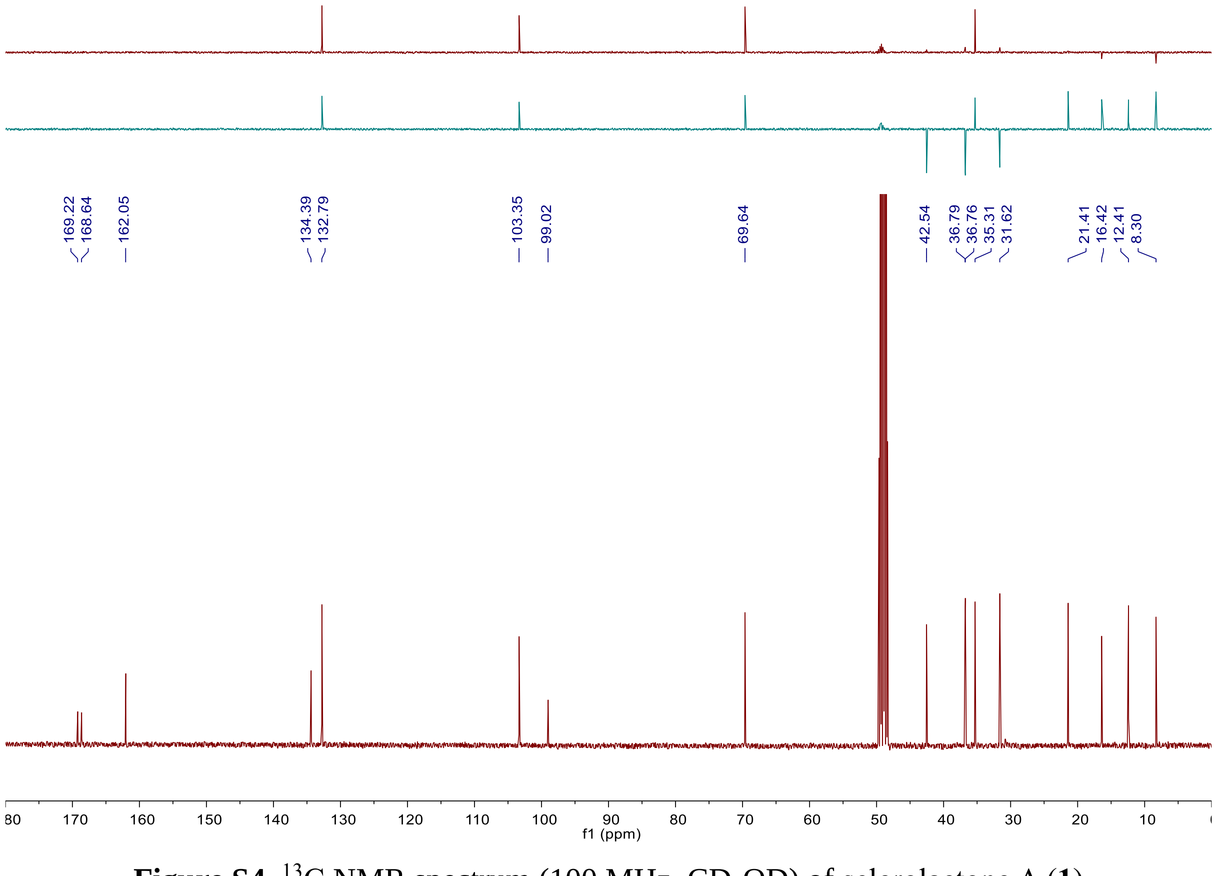

Figure S4. ^{13}C NMR spectrum (100 MHz, CD_3OD) of sclerolactone A (**1**)

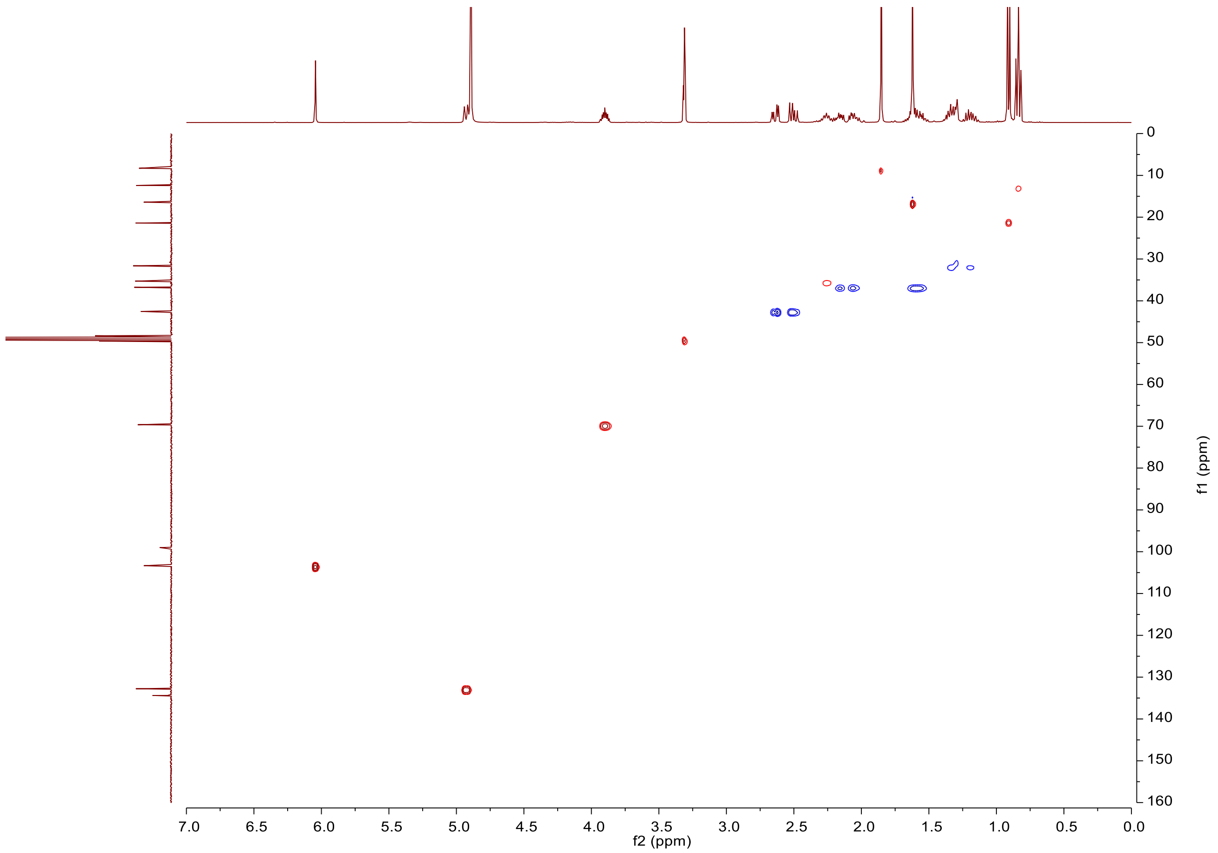

Figure S5. ^{1}H-^{13}C HSQC spectrum of sclerolactone A (**1**)

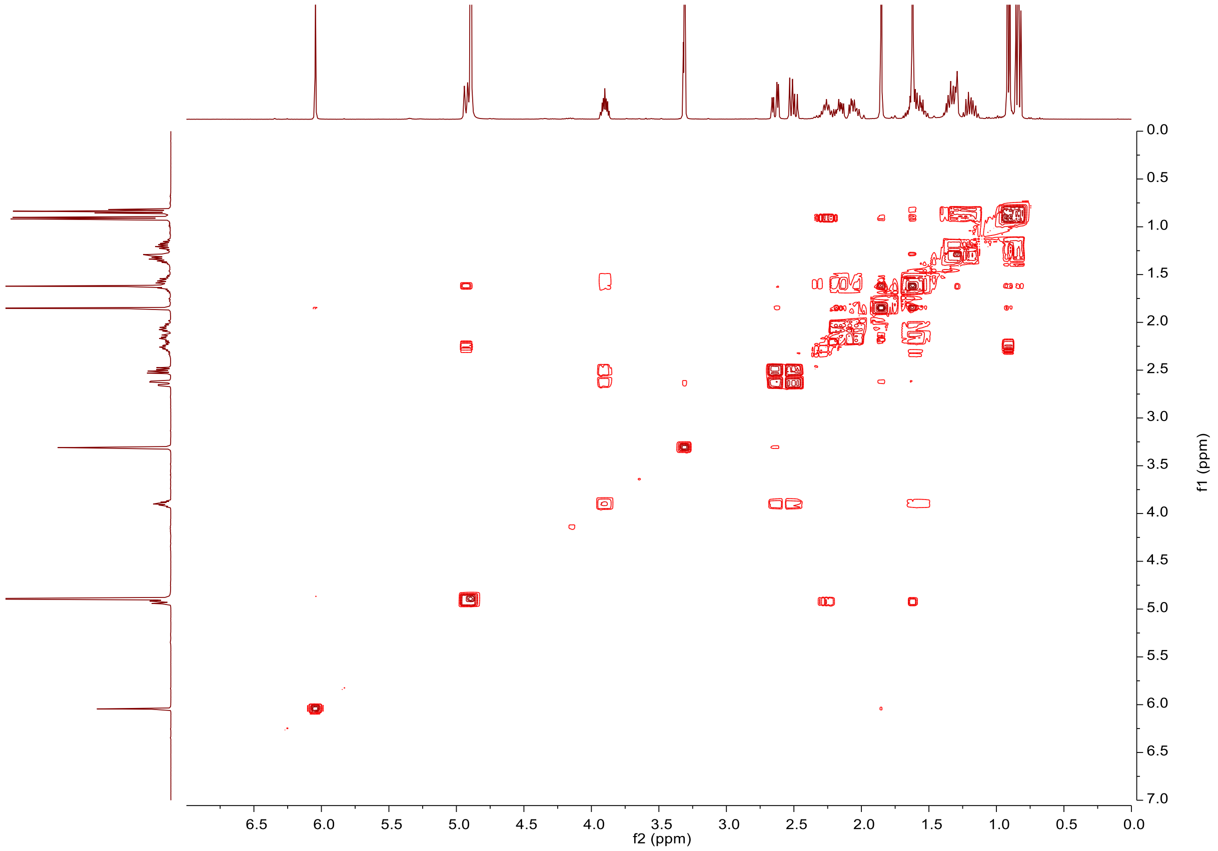

Figure S6. COSY spectrum of sclerolactone A (**1**)

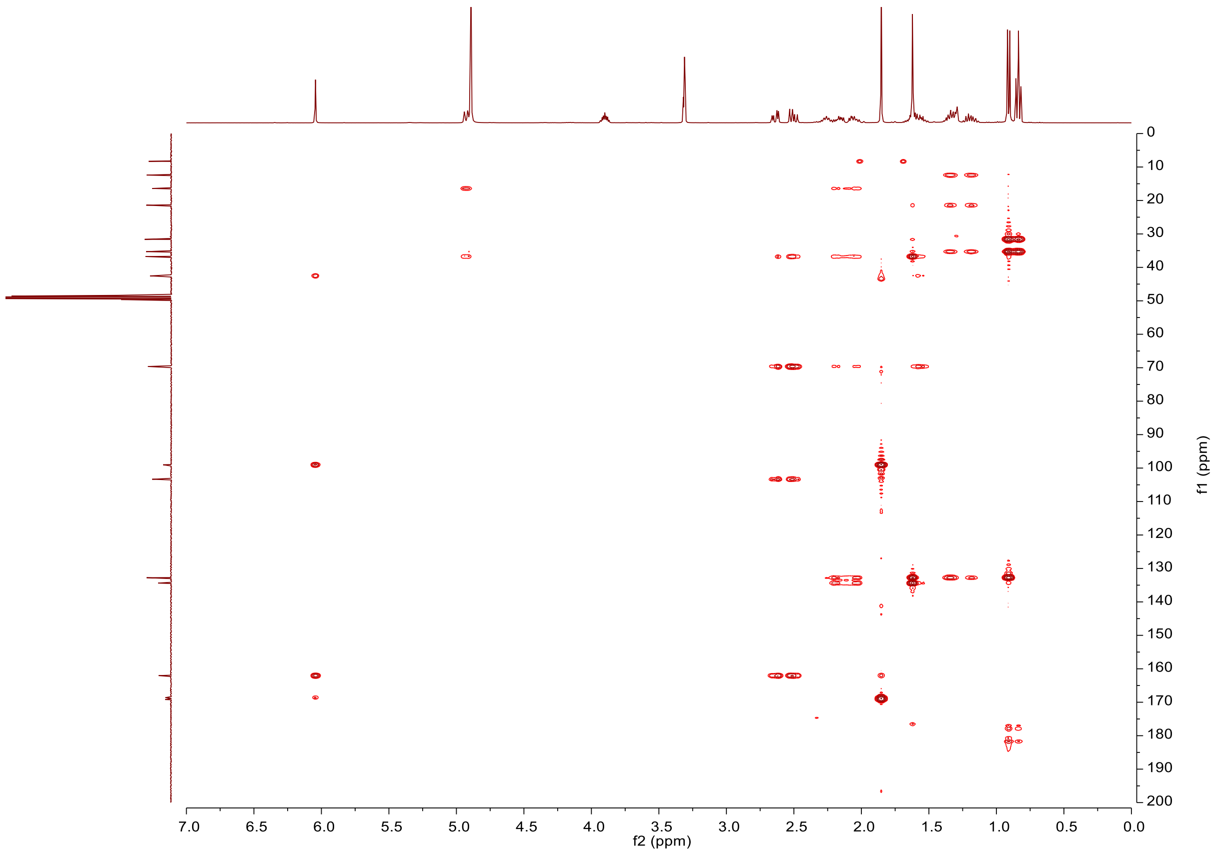

Figure S7. HMBC spectrum of sclerolactone A (**1**)

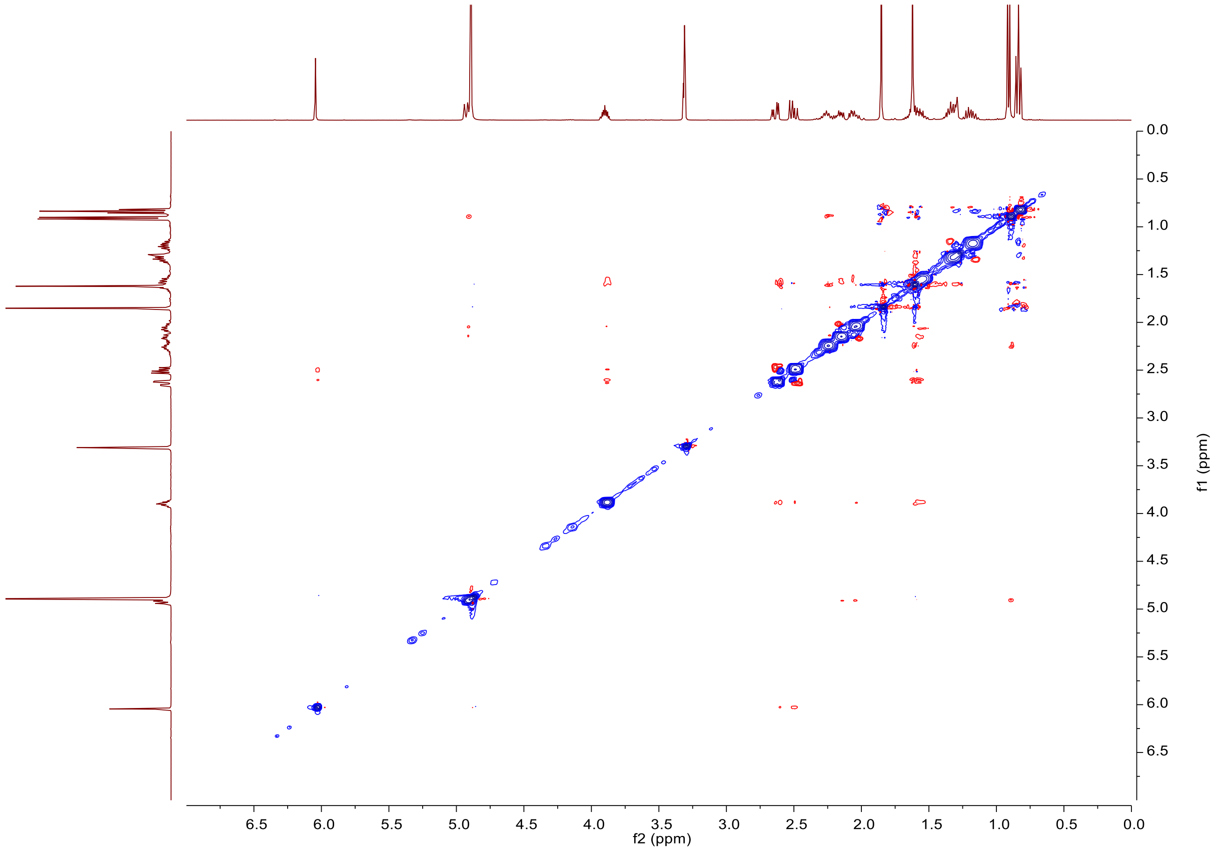

Figure S8. NOESY spectrum of sclerolactone A (**1**)

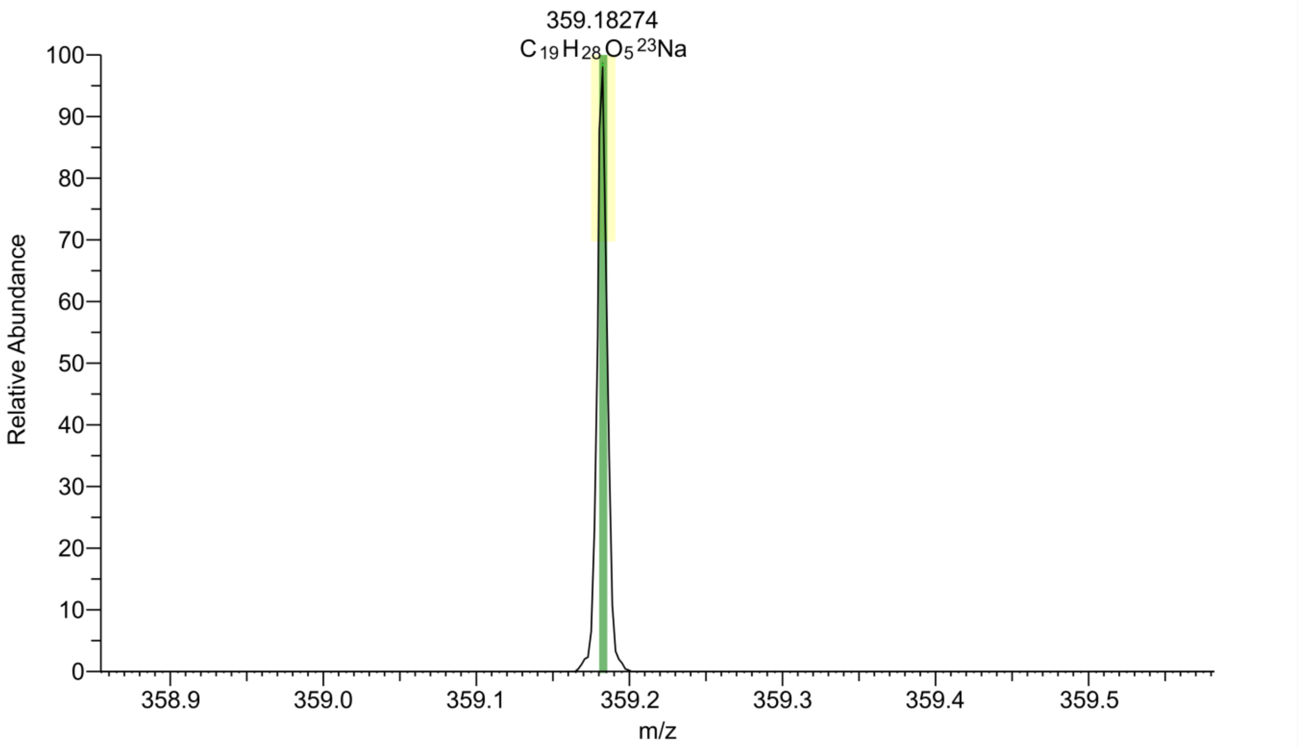

Peak Mass	Display Formula	S Fit	RDB	Delta [ppm]	Theo. mass	Pattern Cov. [%]	MSMS Matched...
359.18274	$C_{19}H_{28}O_5{}^{23}Na$	91.8658212005852	5.50	-0.43	359.18290	100	(Collection)

Figure S9. HRESIMS spectrum of sclerolactone B (**2**)

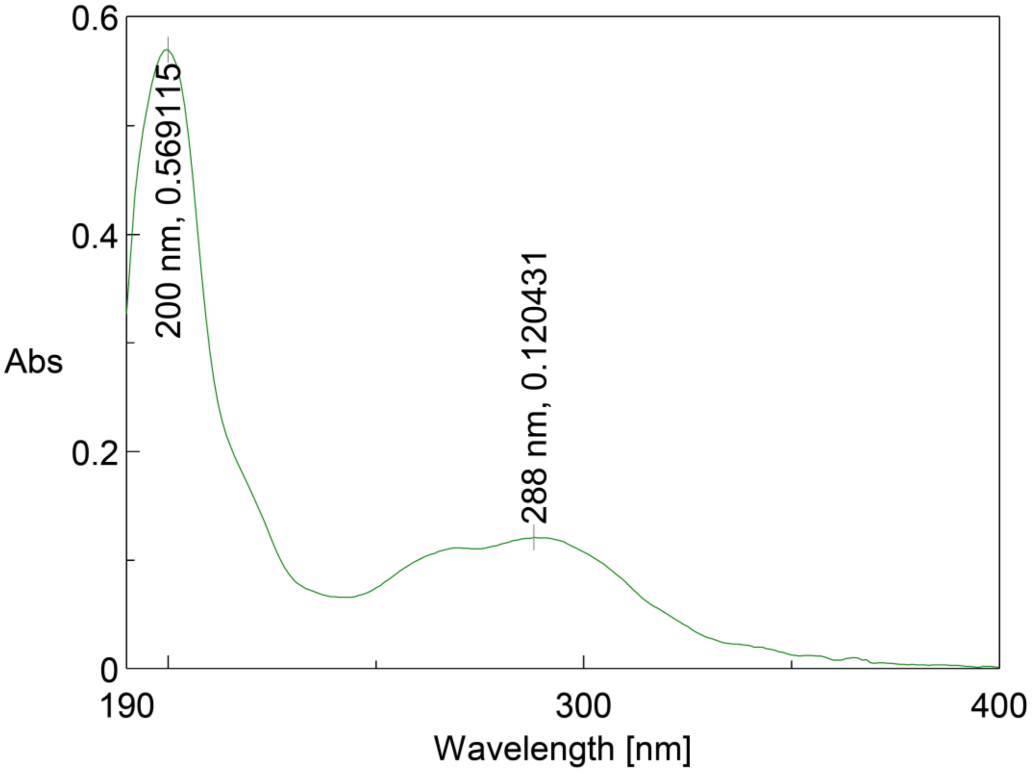

Figure S10. UV spectrum of sclerolactone B (**2**)

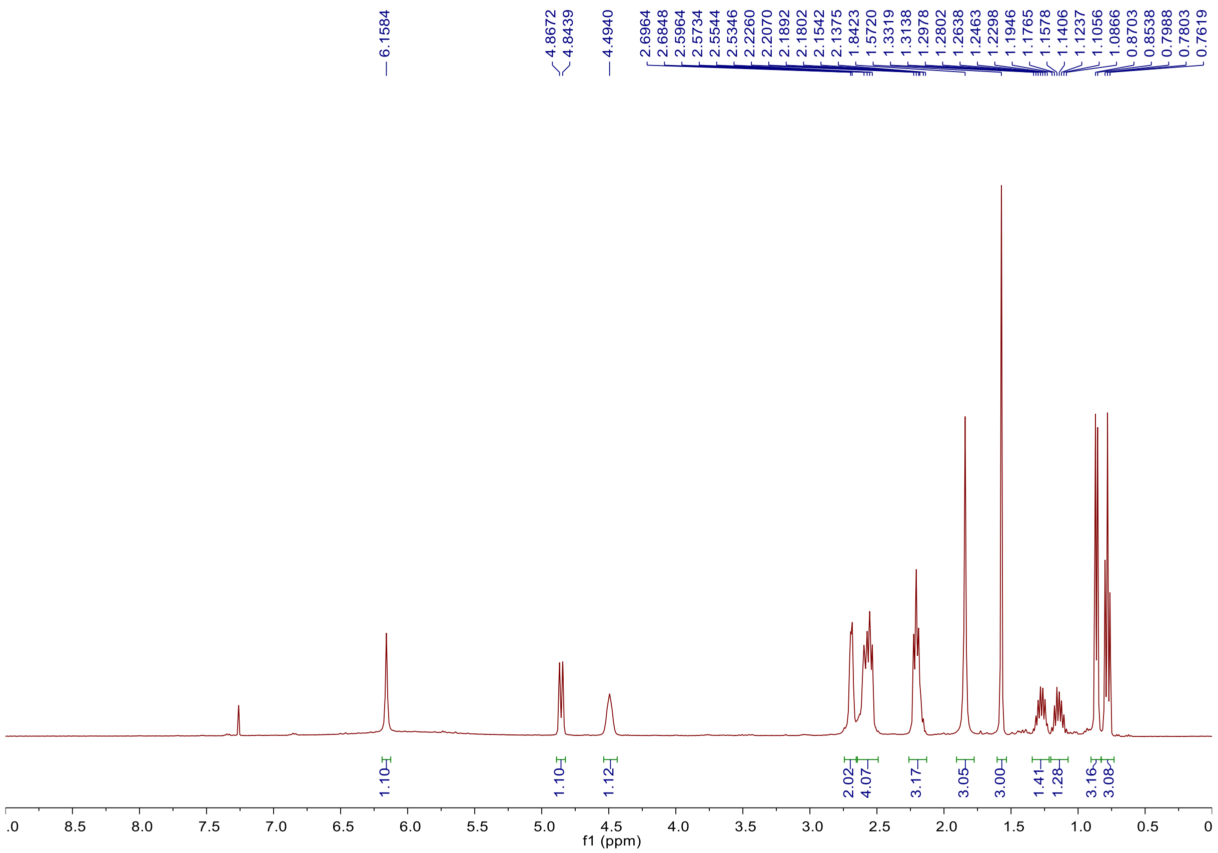

Figure S11. ^{1}H NMR spectrum (400 MHz, $CDCl_3$) of sclerolactone B (**2**)

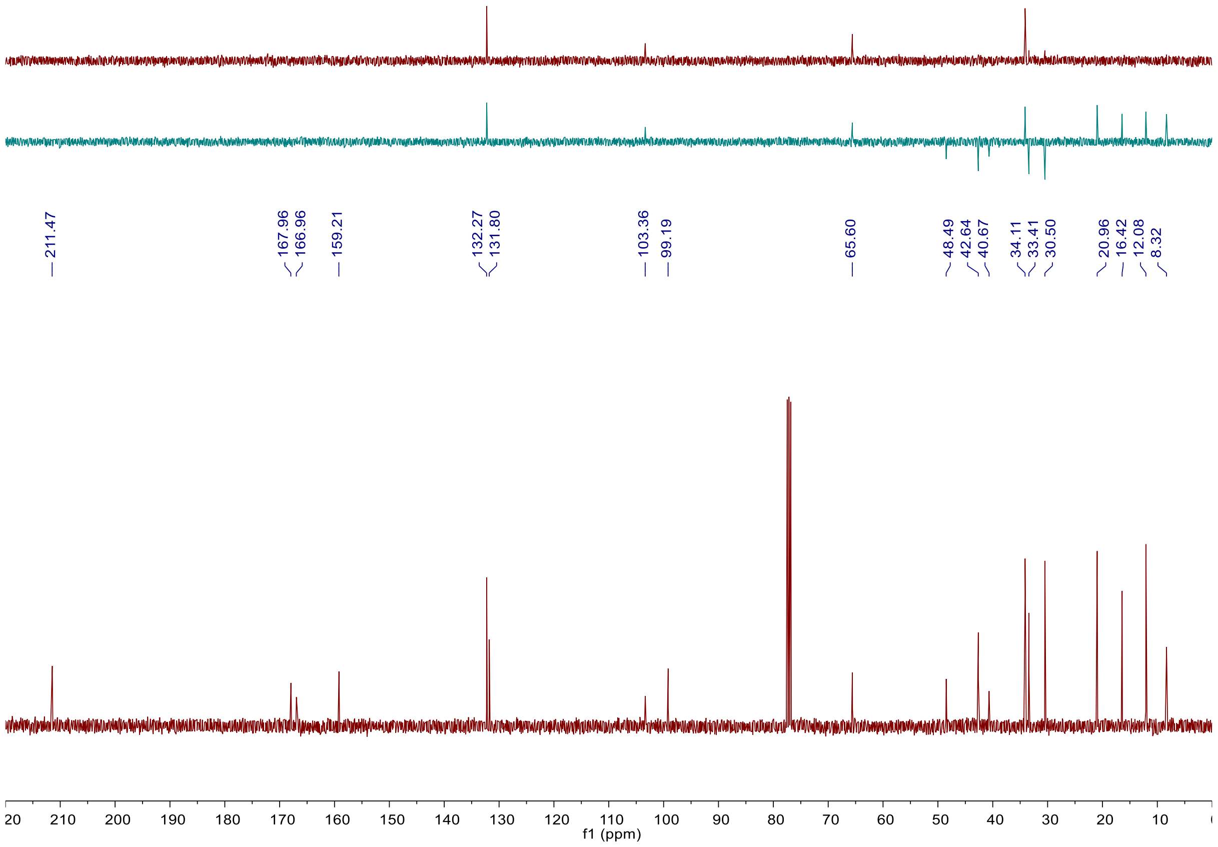

Figure S12. ^{13}C NMR spectrum (100 MHz, $CDCl_3$) of sclerolactone B (**2**)

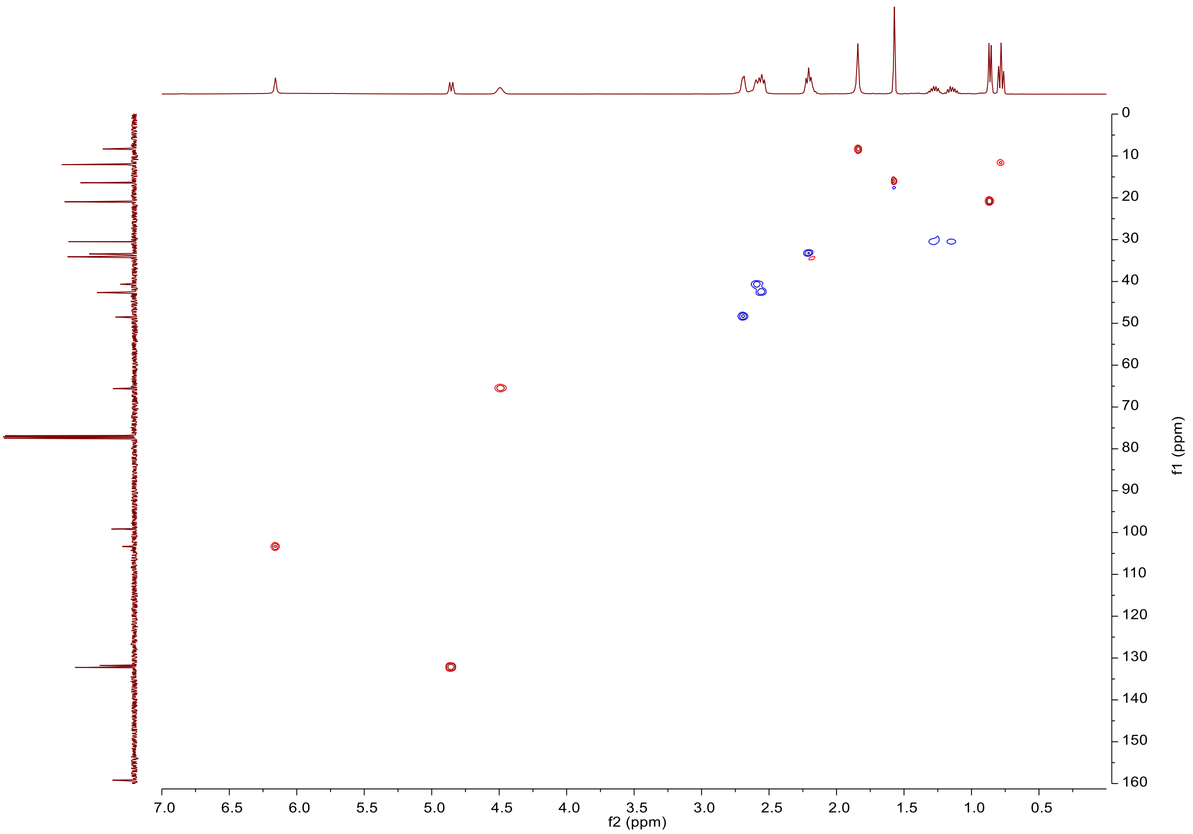

Figure S13. ^{1}H-^{13}C HSQC spectrum of sclerolactone B (**2**)

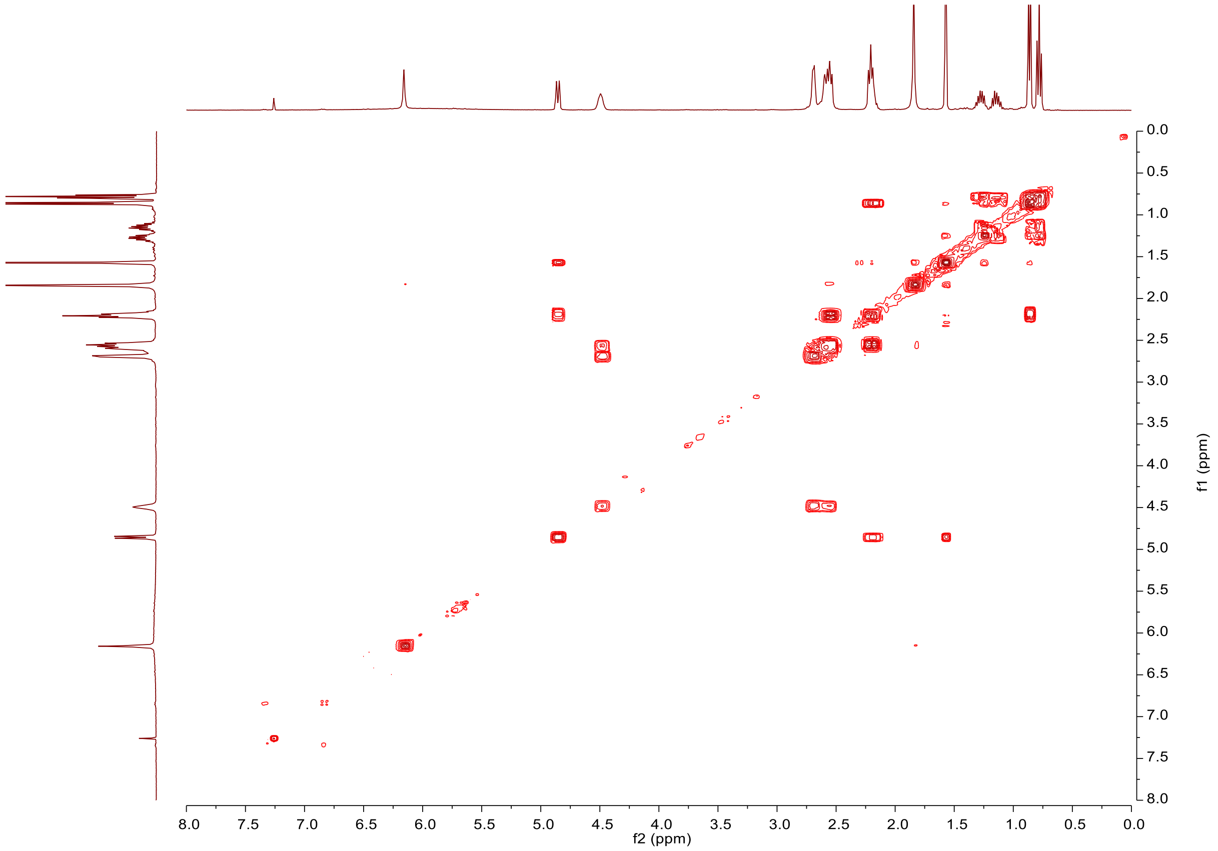

Figure S14. COSY spectrum of sclerolactone B (**2**)

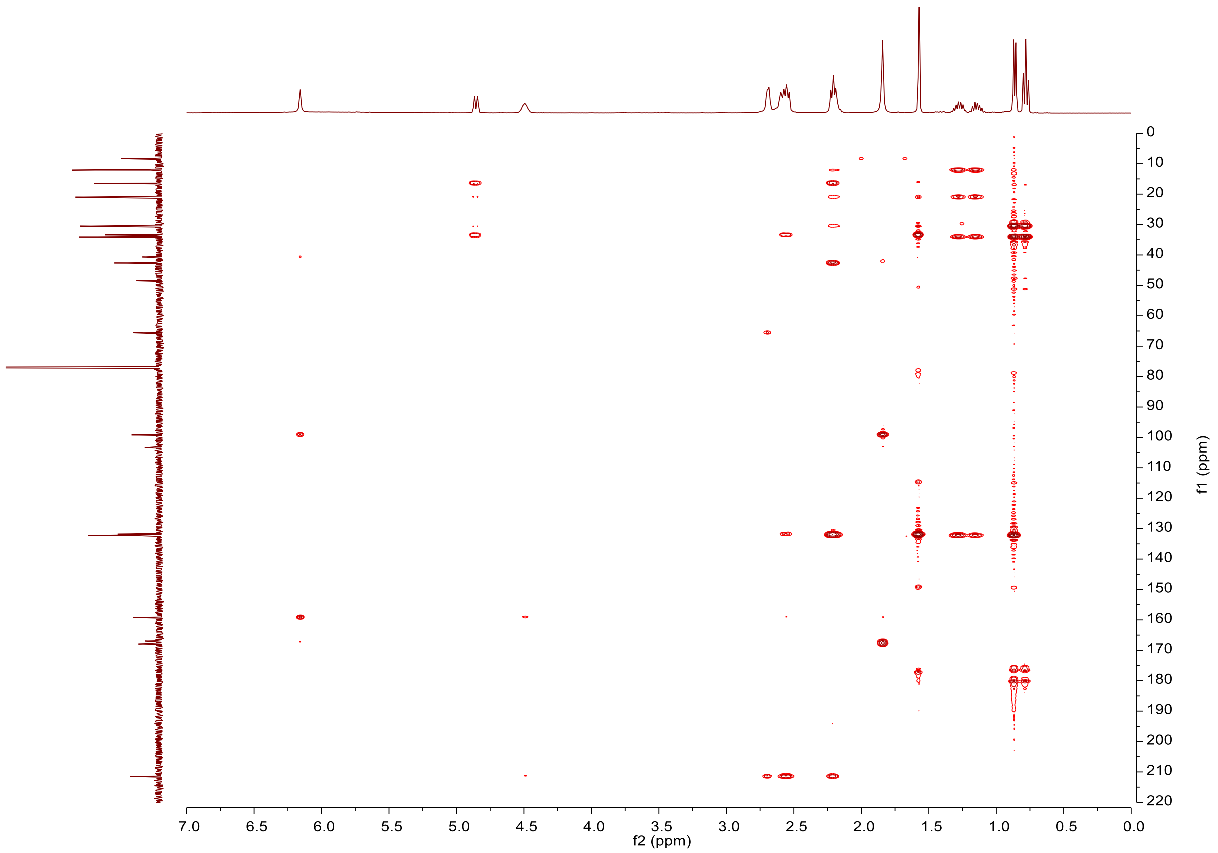

Figure S15. HMBC spectrum of sclerolactone B (**2**)

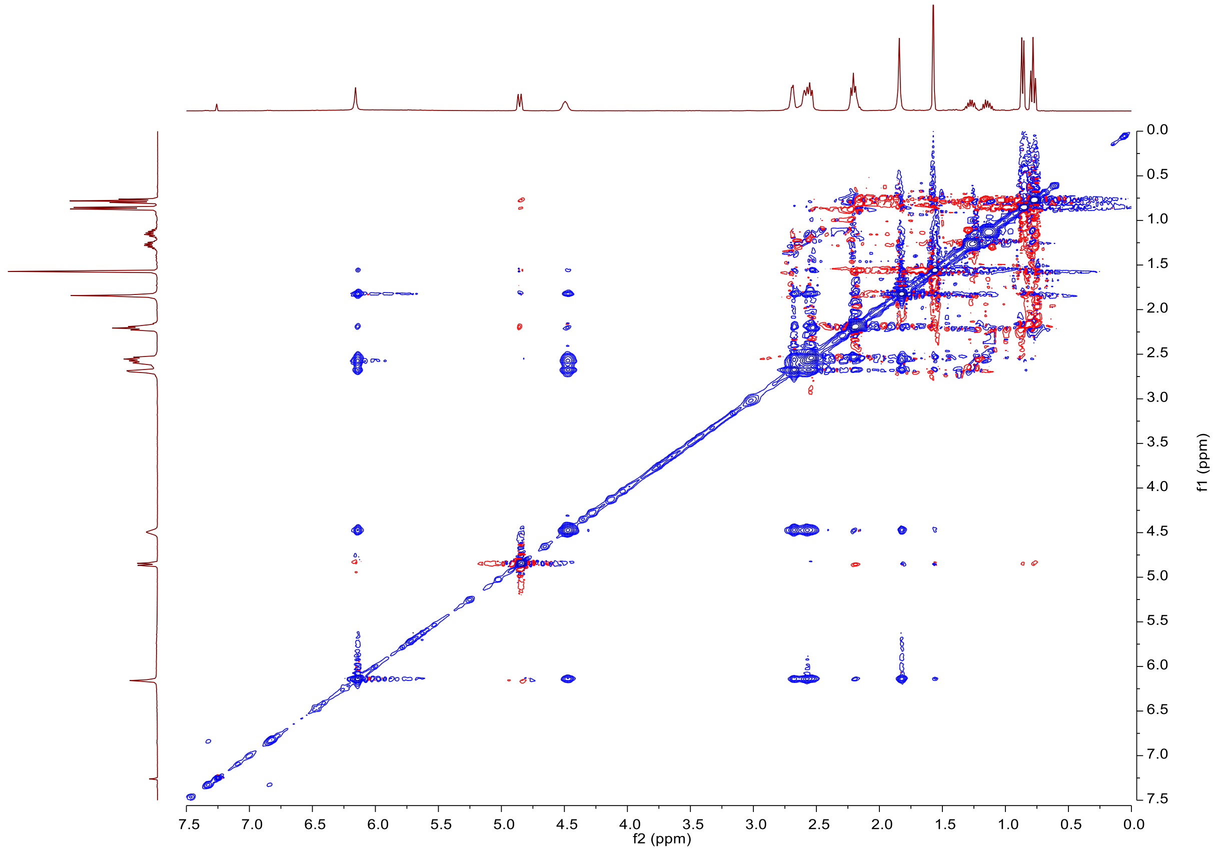

Figure S16. NOESY spectrum of sclerolactone B (**2**)

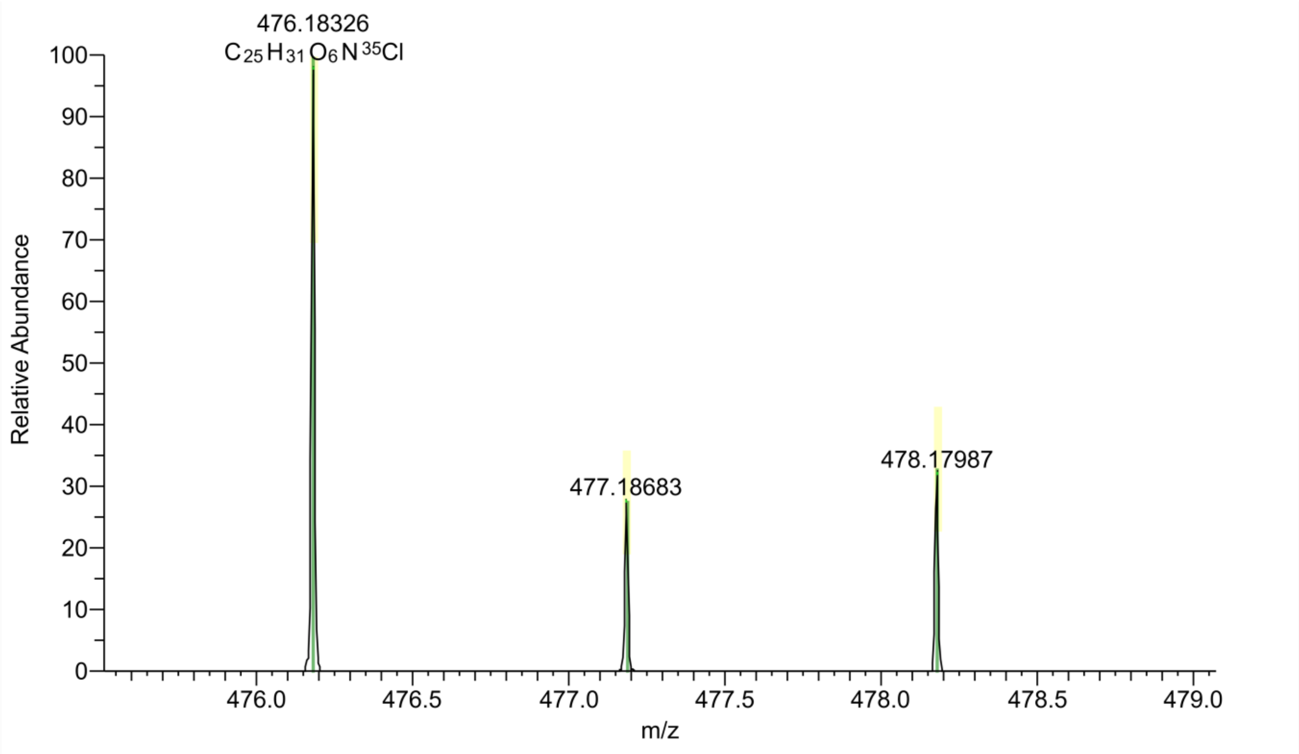

Peak Mass	Display Formula	S Fit	RDB	Delta [ppm]	Theo. mass	Pattern Cov. [%]	MSMS Matched...
476.18326	$C_{25}H_{31}O_6N^{35}Cl$	51.56268253839 5	10.50	-0.39	476.18344	99.89	(Collection)

Figure S17. HRESIMS spectrum of peniphilone A (**3**)

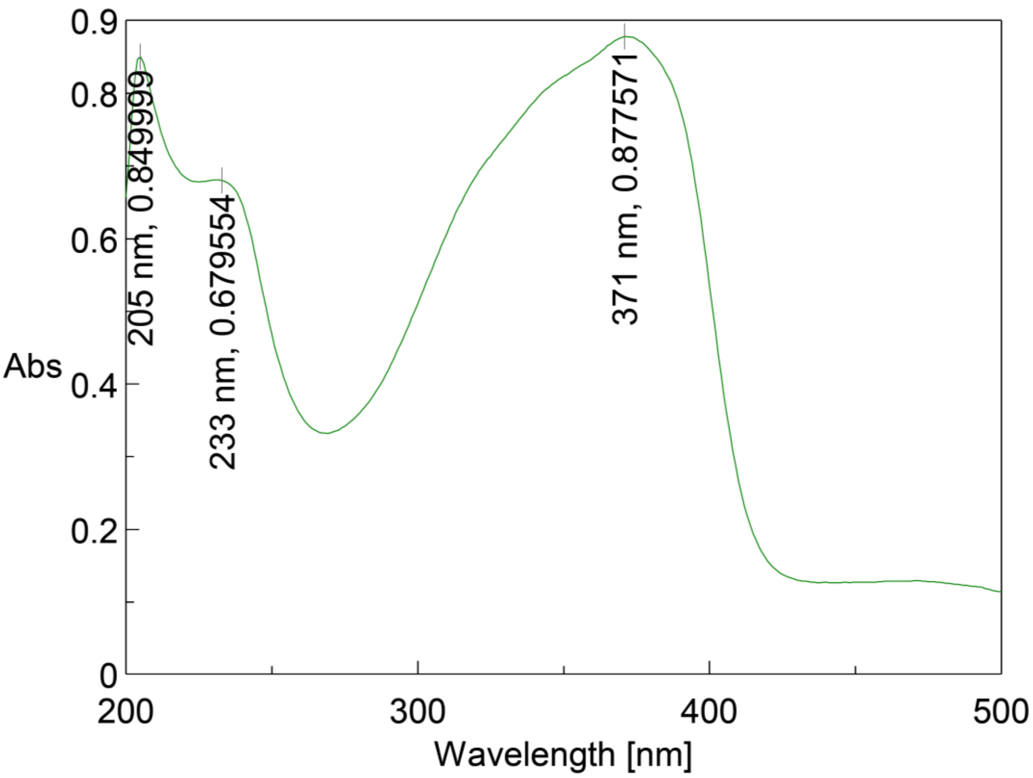

Figure S18. UV spectrum of peniphilone A (**3**)

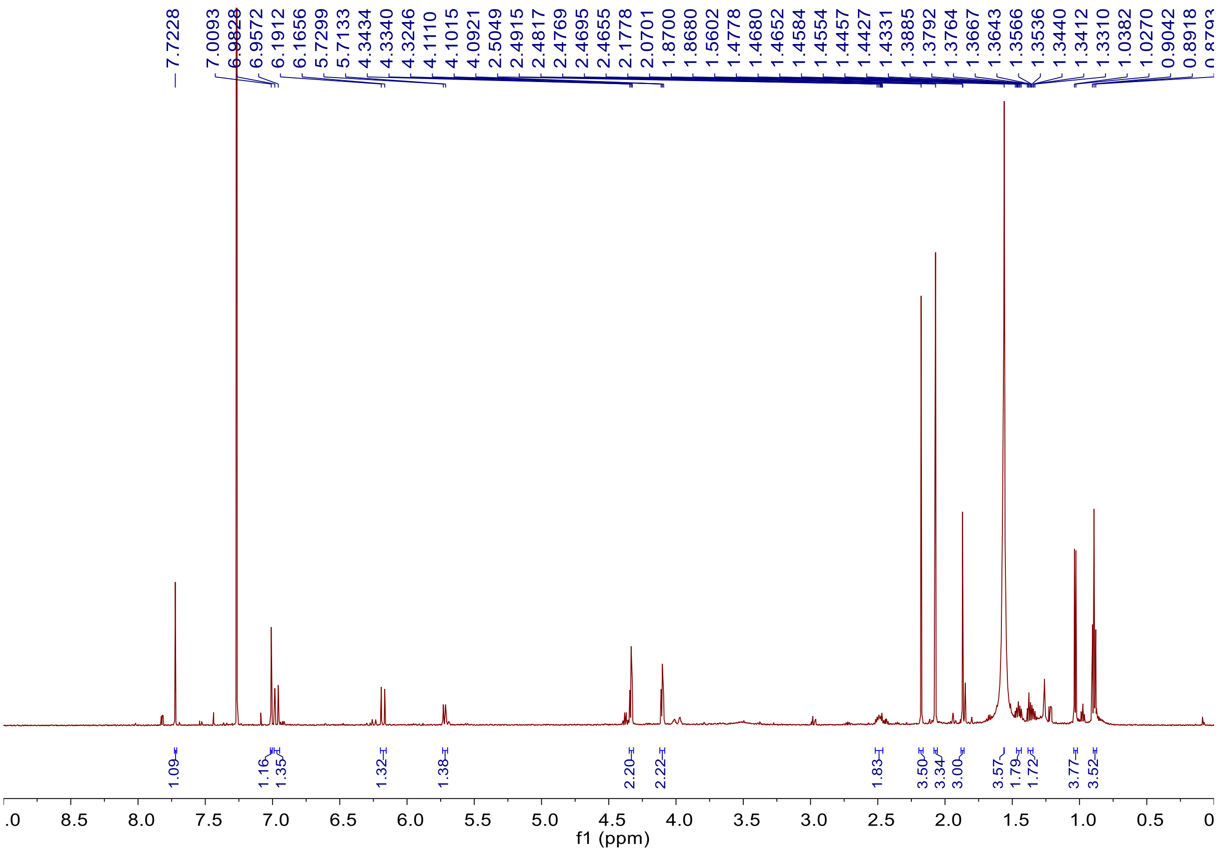

Figure S19. ^{1}H NMR spectrum (600 MHz, $CDCl_3$) of peniphilone A (**3**)

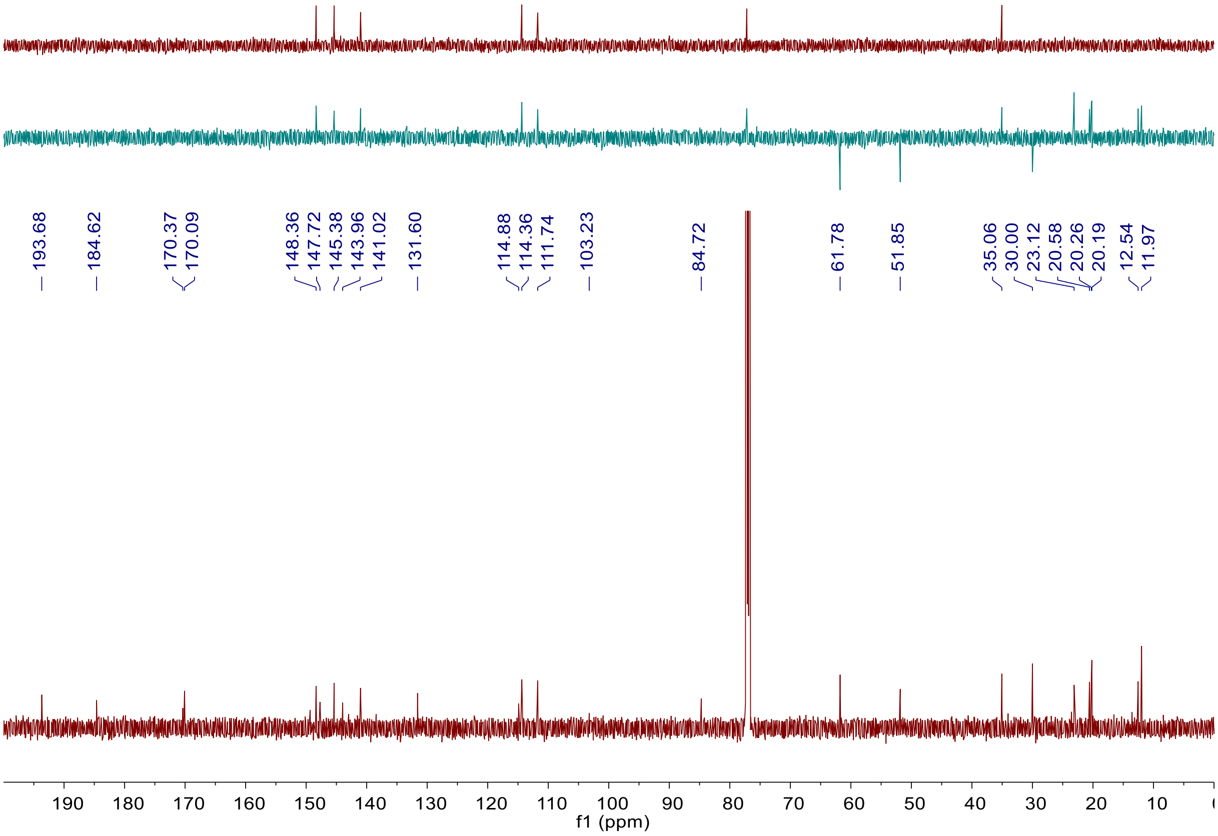

Figure S20. ^{13}C NMR spectrum (150 MHz, $CDCl_3$) of peniphilone A (**3**)

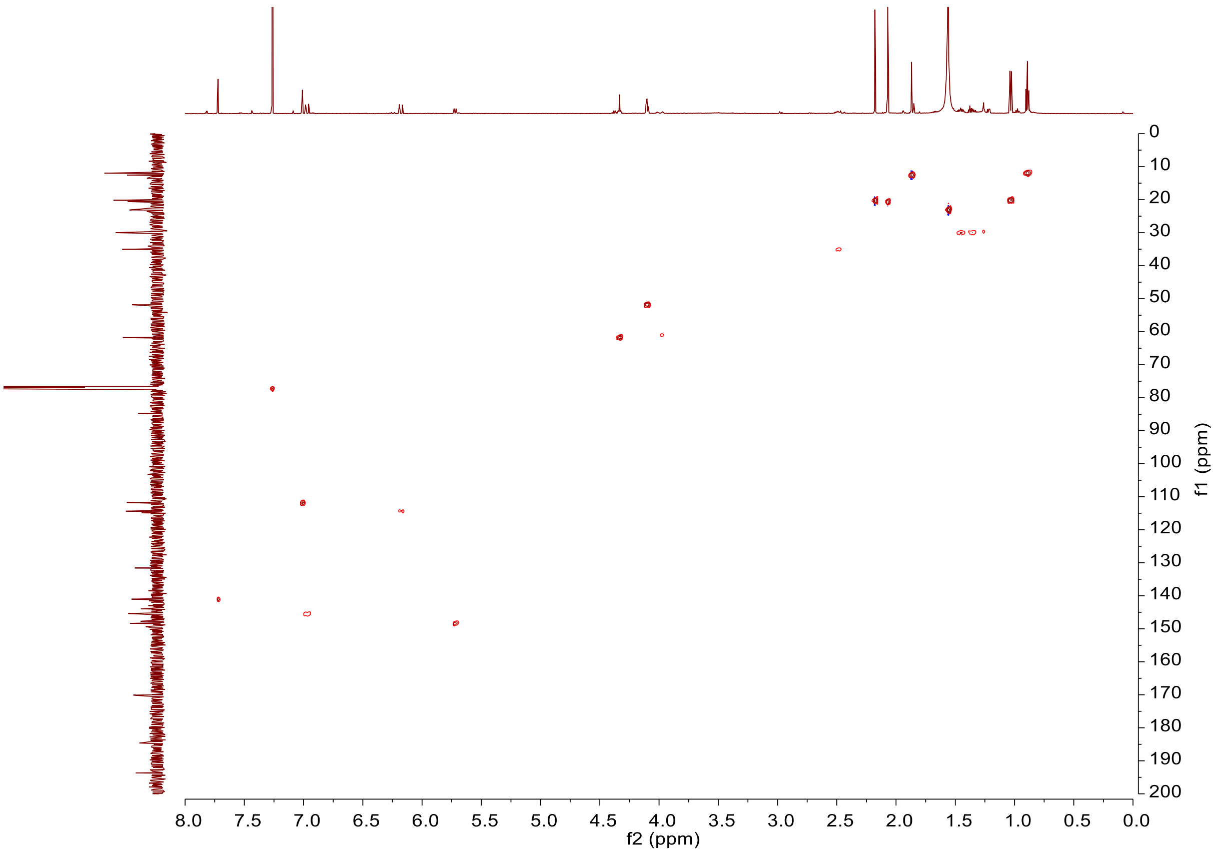

Figure S21. ^{1}H-^{13}C HSQC spectrum of peniphilone A (**3**)

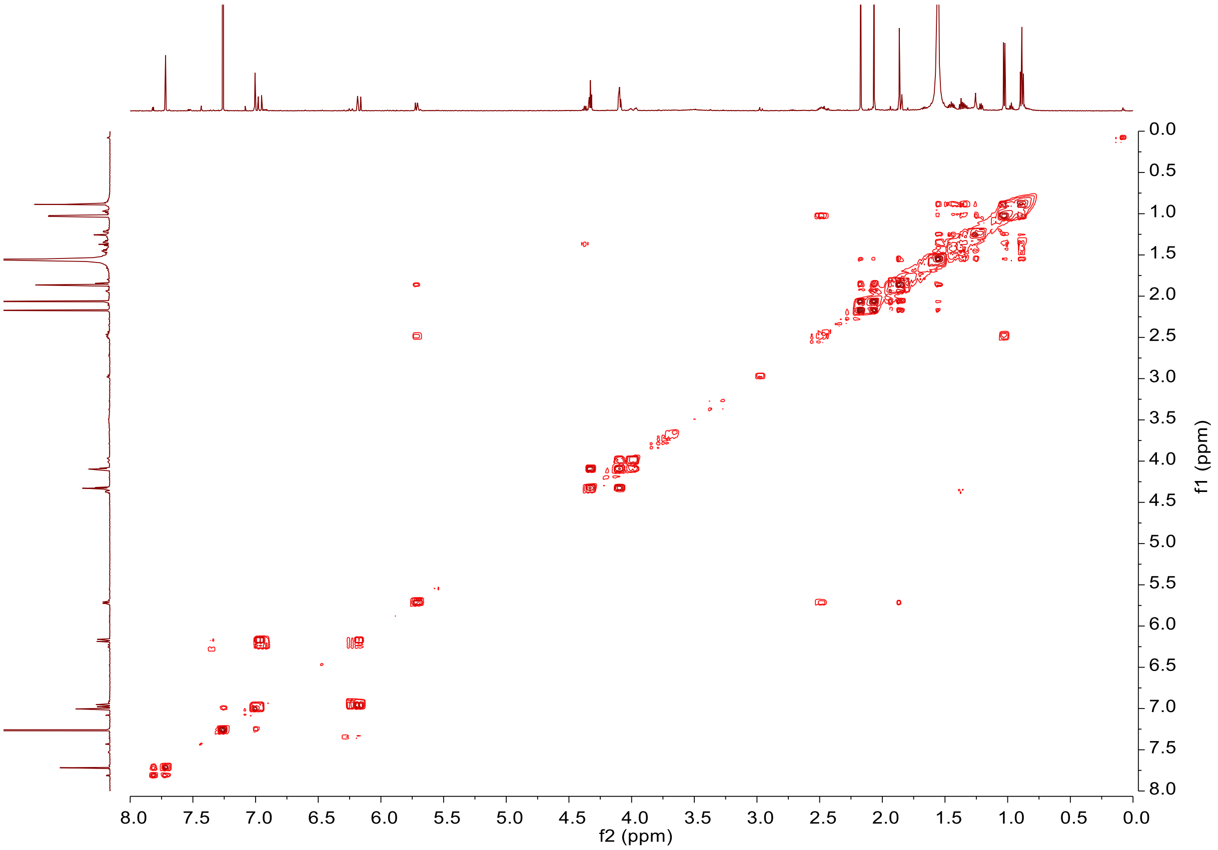

Figure S22. COSY spectrum of peniphilone A (**3**)

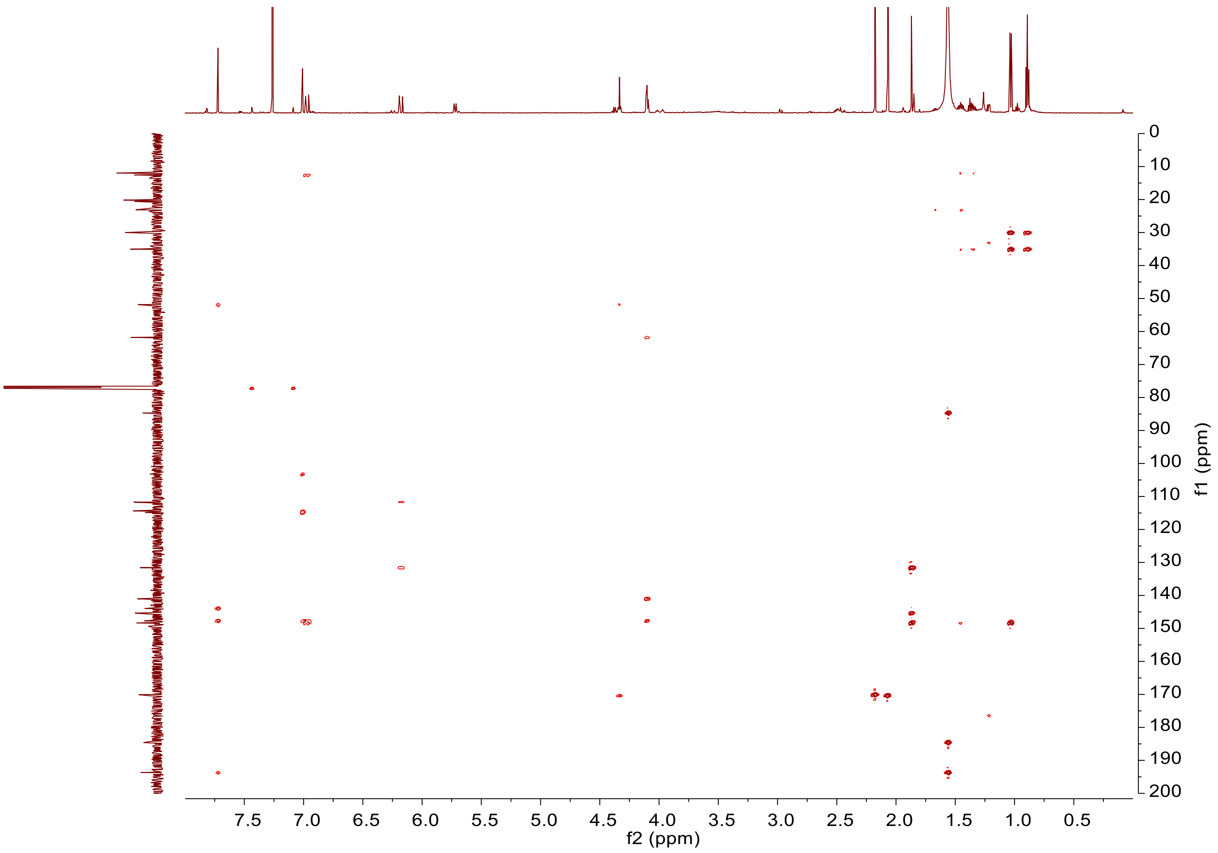

Figure S23. HMBC spectrum of peniphilone A (**3**)

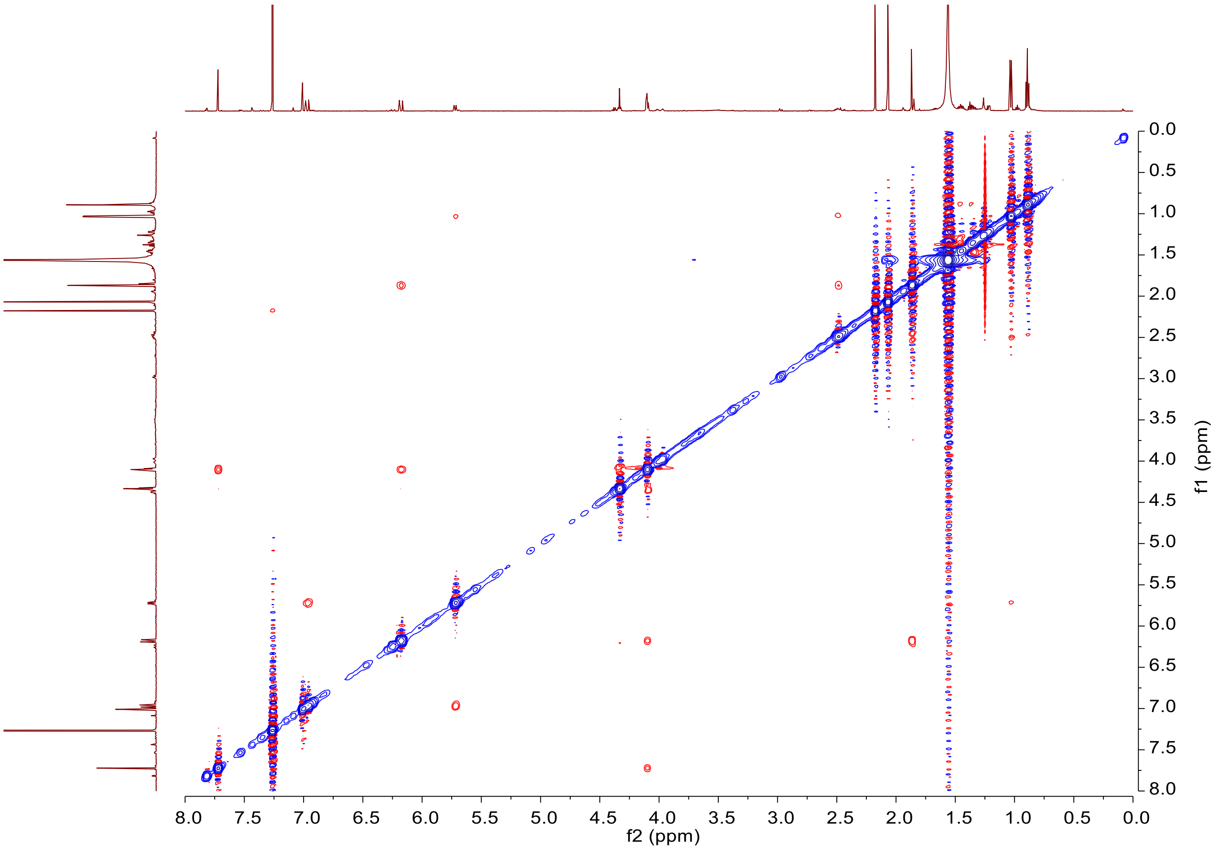

Figure S24. NOESY spectrum of peniphilone A (**3**)

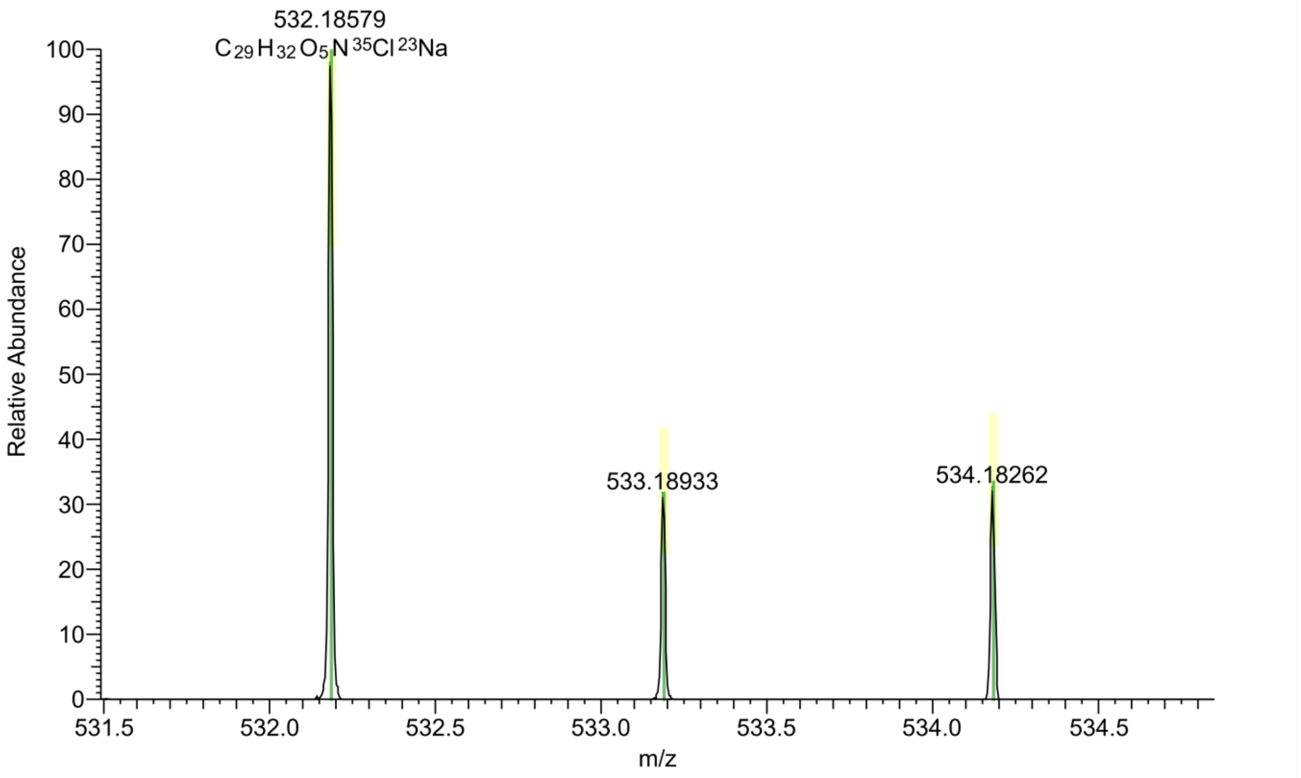

Peak Mass	Display Formula	S Fit	RDB	Delta [ppm]	Theo. mass	Pattern Cov. [%]	MSMS Matched...
532.18579	$C_{29}H_{32}O_5N^{35}Cl^{23}Na$	77.4295724814099	13.50	-0.62	532.18612	100	(Collection)

Figure S25. HRESIMS spectrum of peniphilone B (**4**)

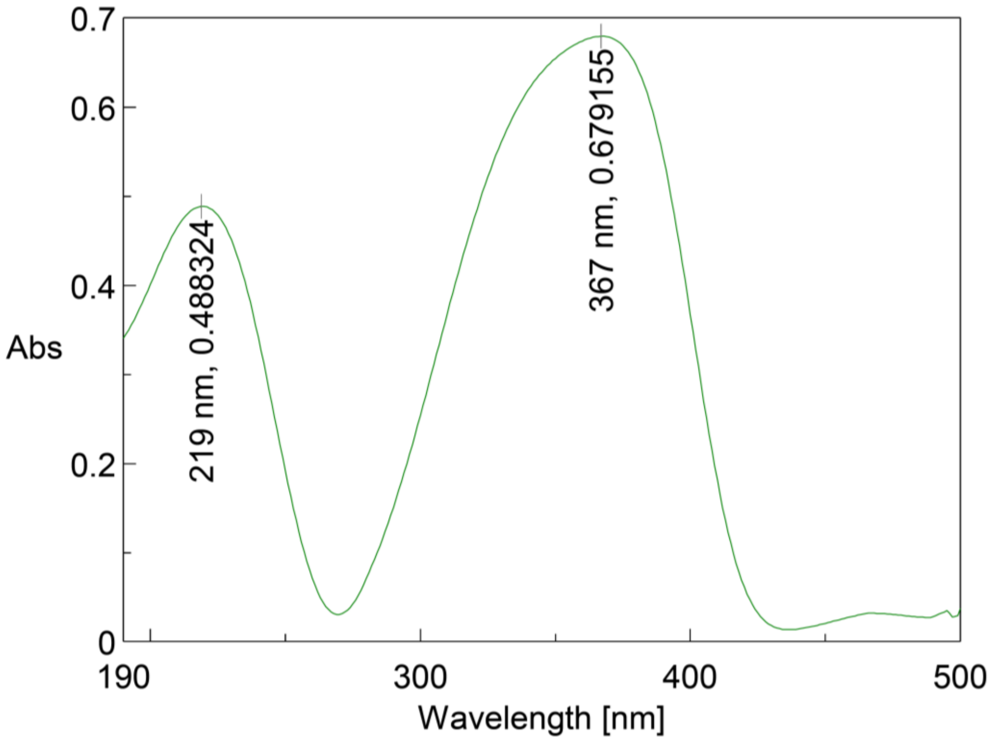

Figure S26. UV spectrum of peniphilone B (**4**)

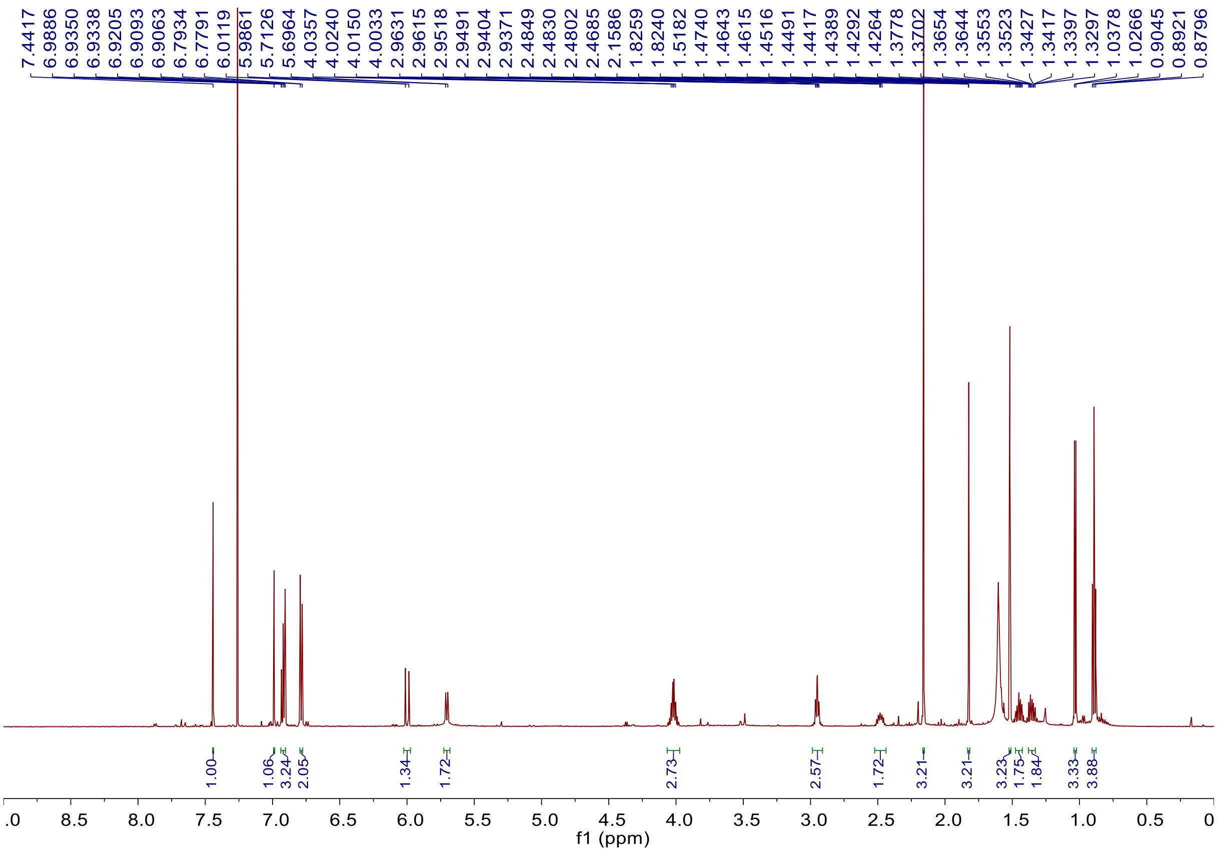

Figure S27. ^{1}H NMR spectrum (600 MHz, $CDCl_3$) of peniphilone B (**4**)

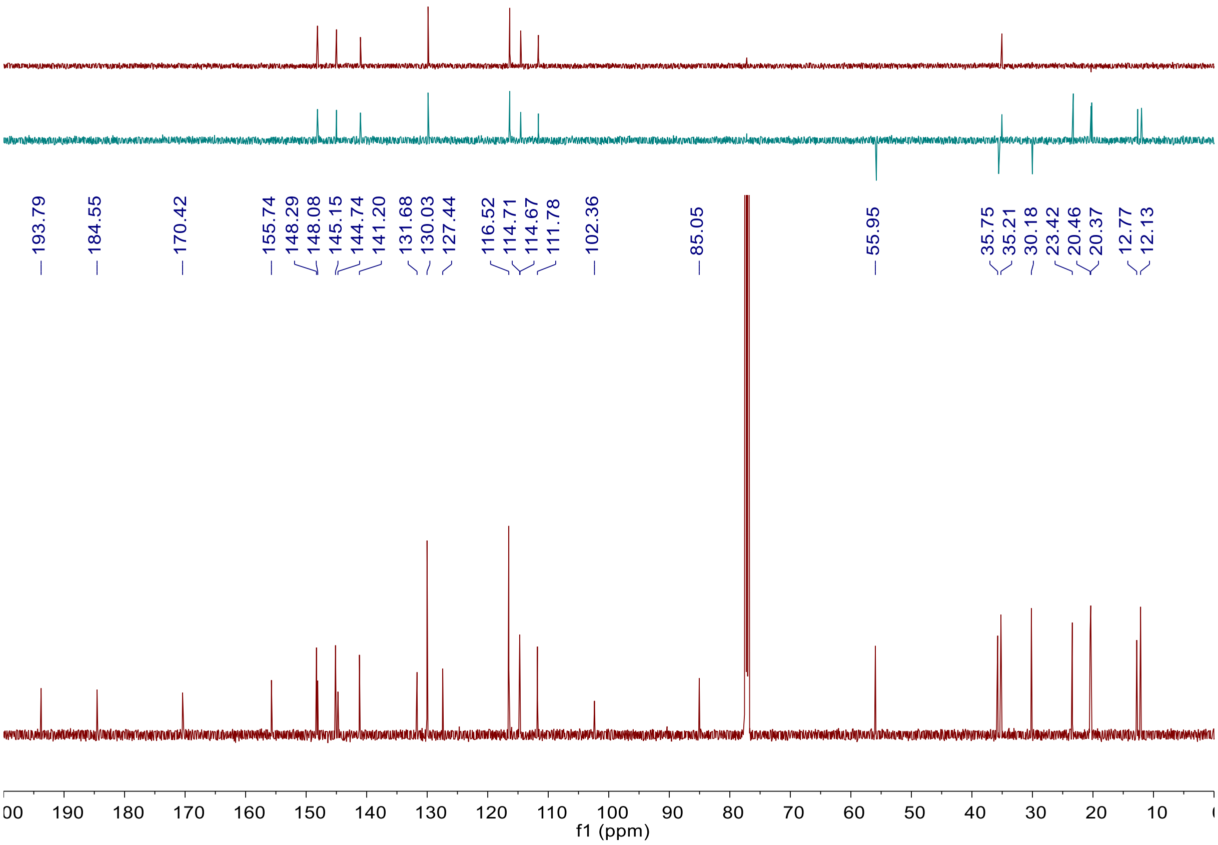

Figure S28. ^{13}C NMR spectrum (150 MHz, $CDCl_3$) of peniphilone B (**4**)

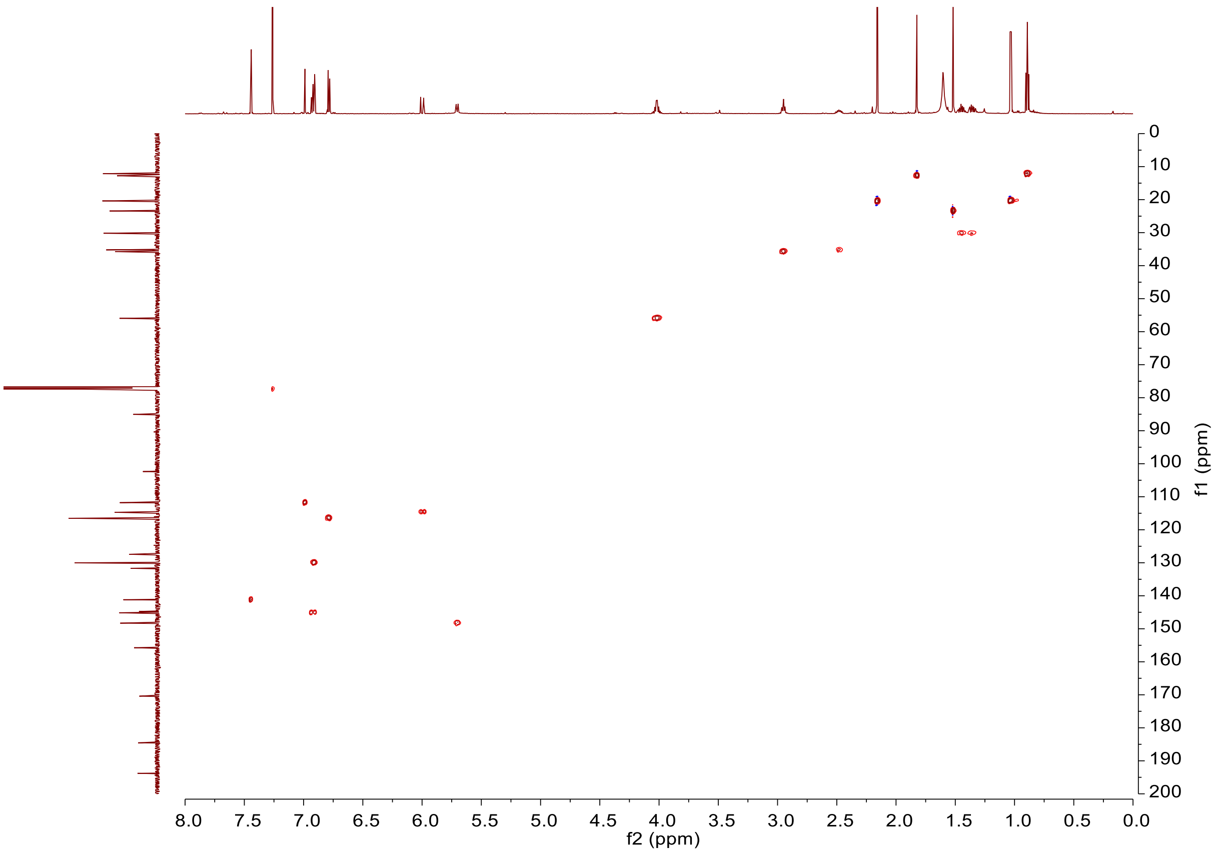

Figure S29. ^{1}H-^{13}C HSQC spectrum of peniphilone B (**4**)

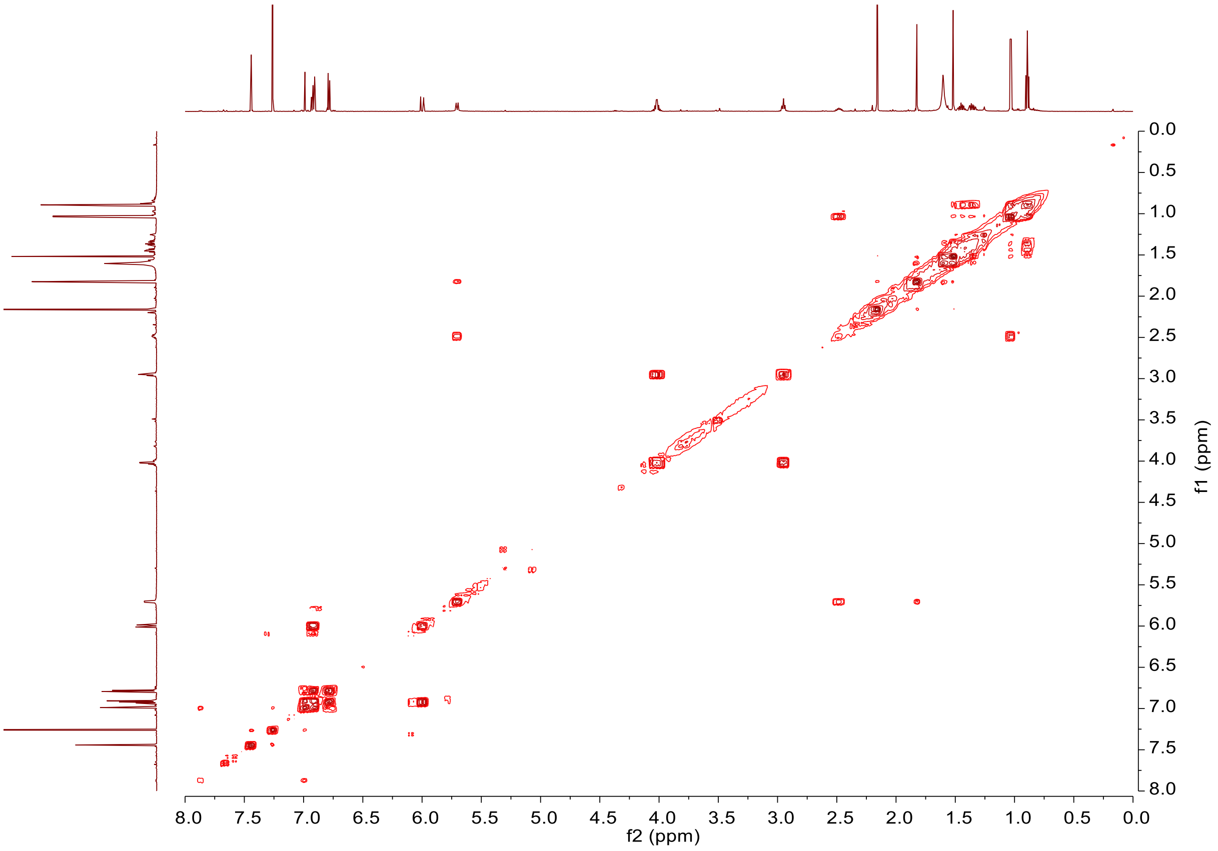

Figure S30. COSY spectrum of peniphilone B (**4**)

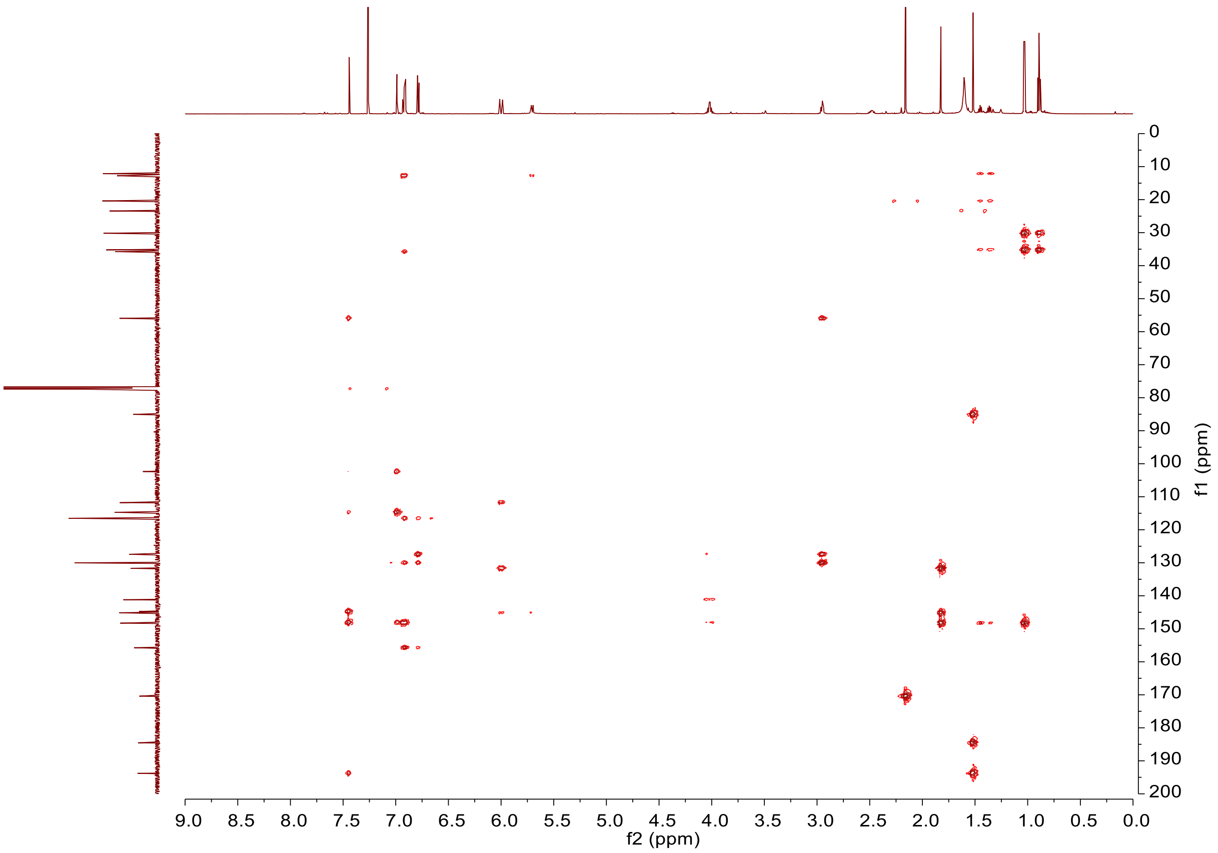

Figure S31. HMBC spectrum of peniphilone B (**4**)

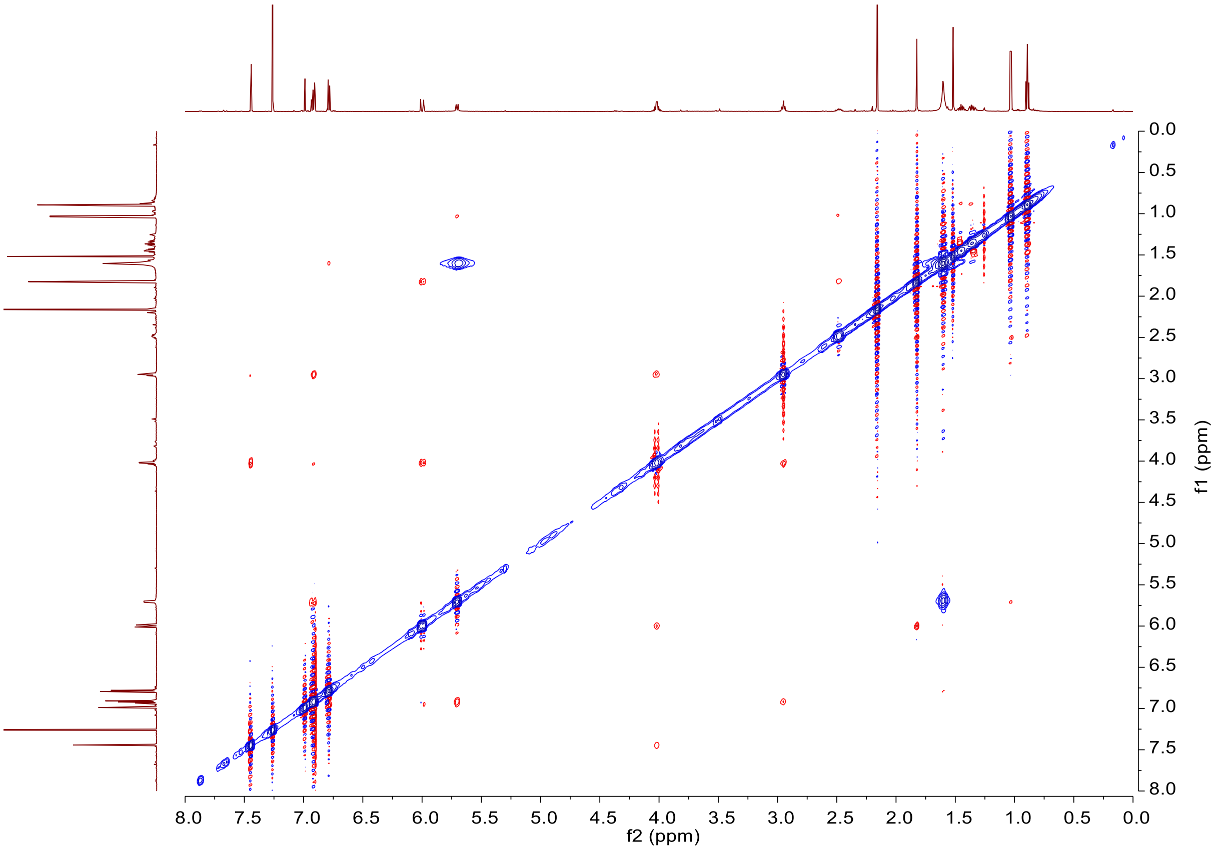

Figure S32. NOESY spectrum of peniphilone B (**4**)

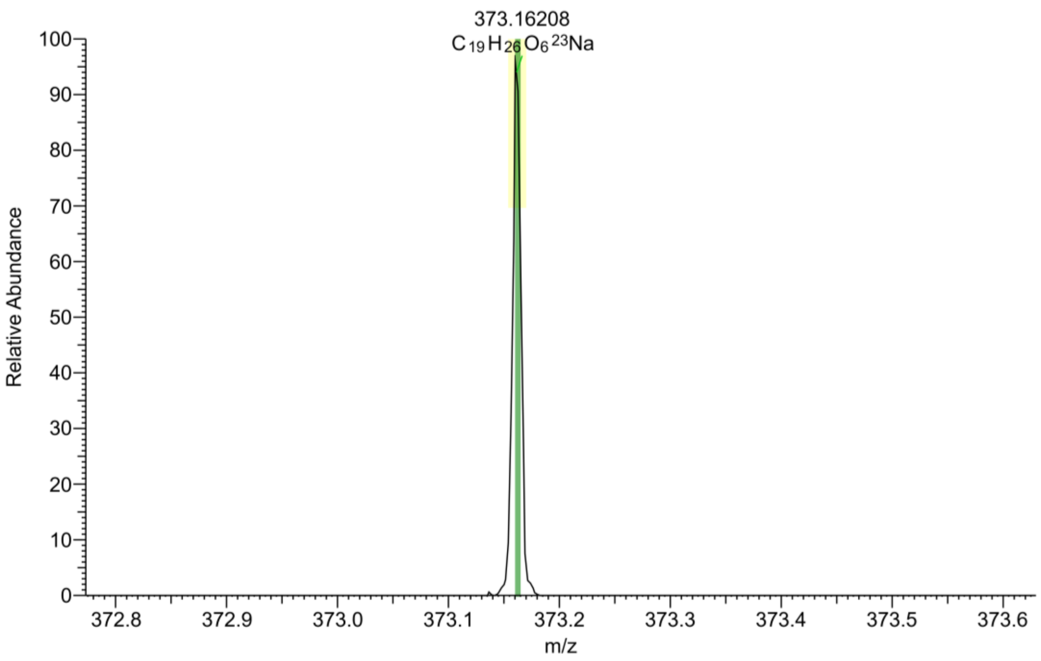

Peak Mass	Display Formula	S Fit	RDB	Delta [ppm]	Theo. mass	Pattern Cov. [%]	MSMS Matched...
373.16208	$C_{19}H_{26}O_6{}^{23}Na$	83.93463461351 24	6.50	-0.22	373.16216	100	(Collection)

Figure S33. HRESIMS spectrum of peniphilone C (**5**)

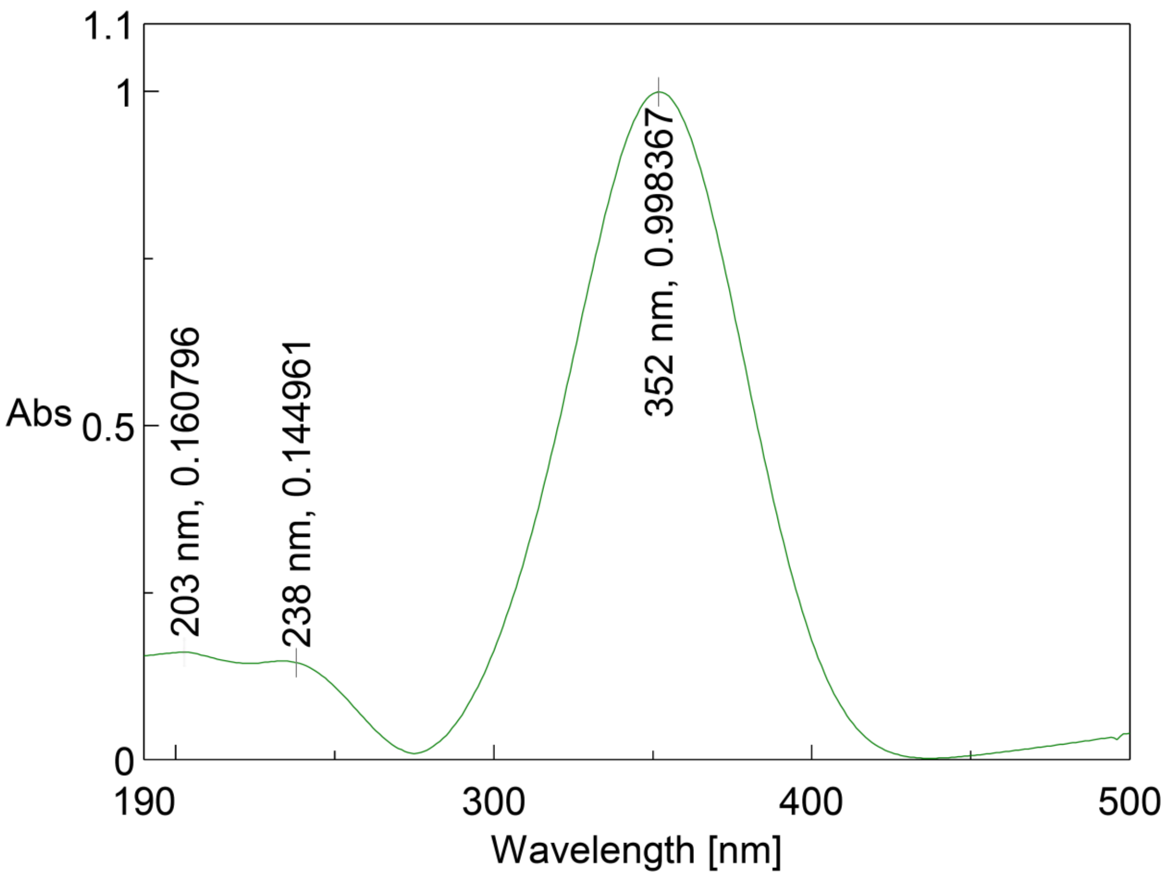

Figure S34. UV spectrum of peniphilone C (**5**)

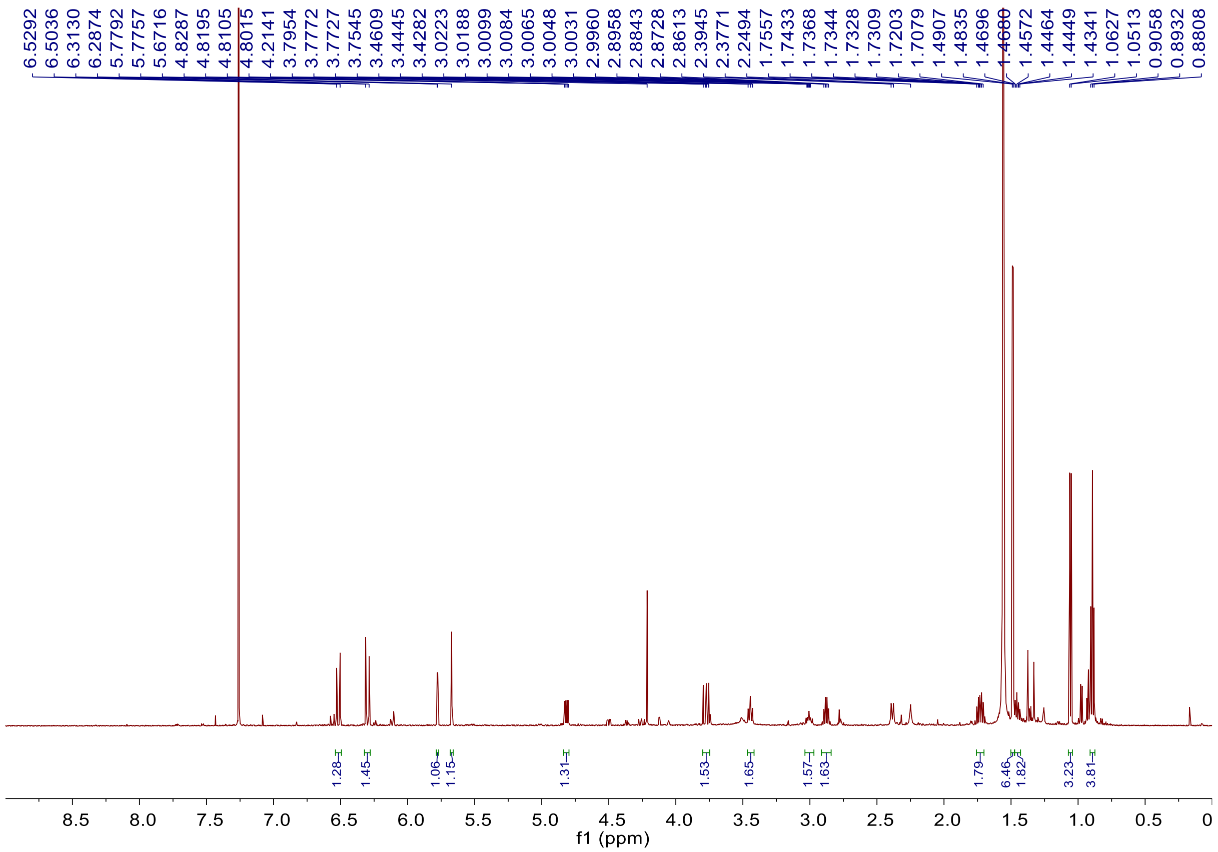

Figure S35. ^{1}H NMR spectrum (600 MHz, $CDCl_3$) of peniphilone C (**5**)

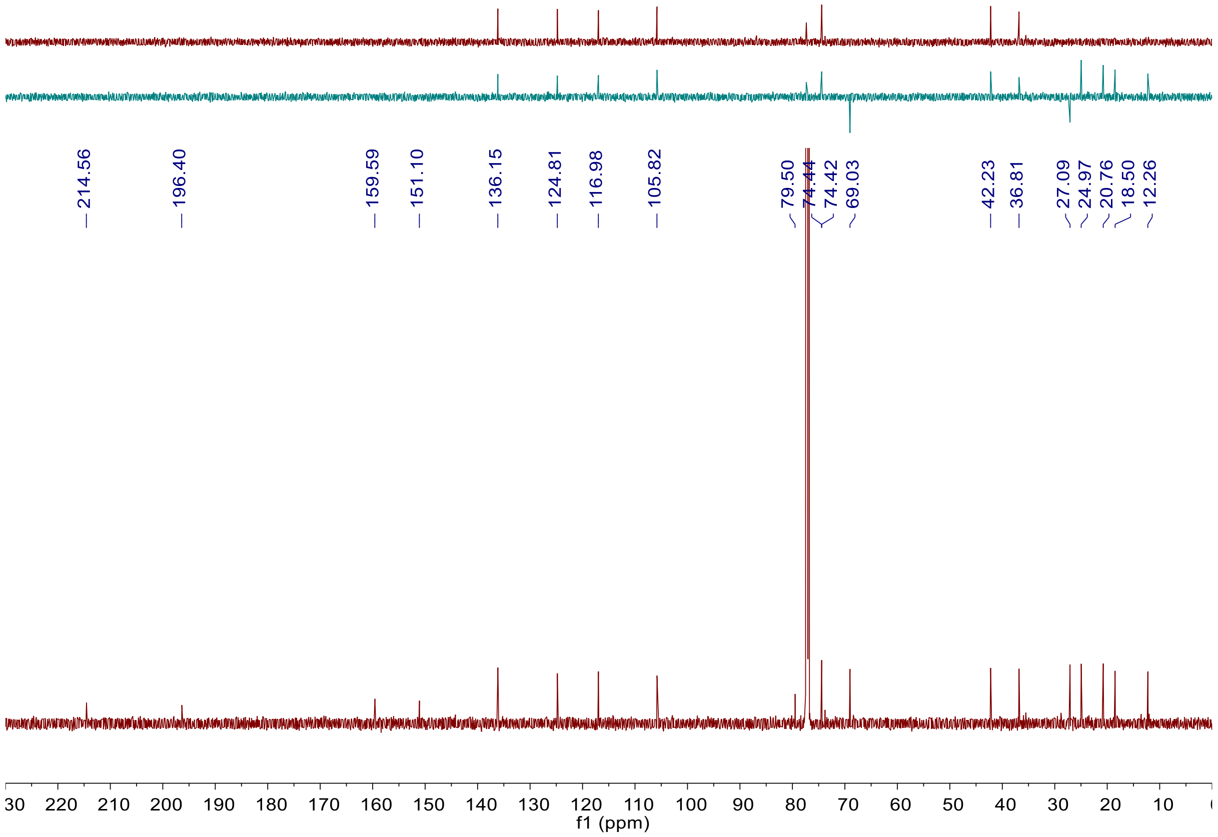

Figure S36. ^{13}C NMR spectrum (150 MHz, $CDCl_3$) of peniphilone C (**5**)

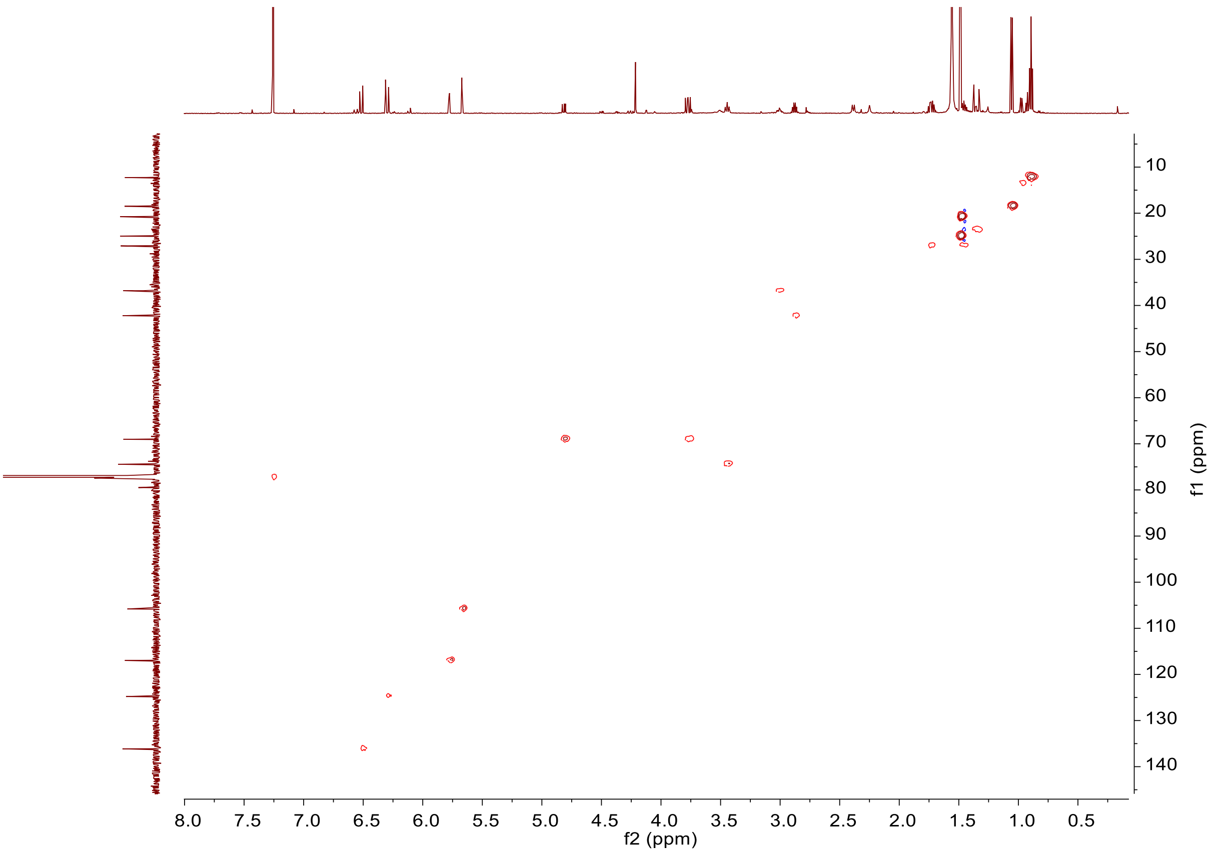

Figure S37. ^{1}H-^{13}C HSQC spectrum of peniphilone C (**5**)

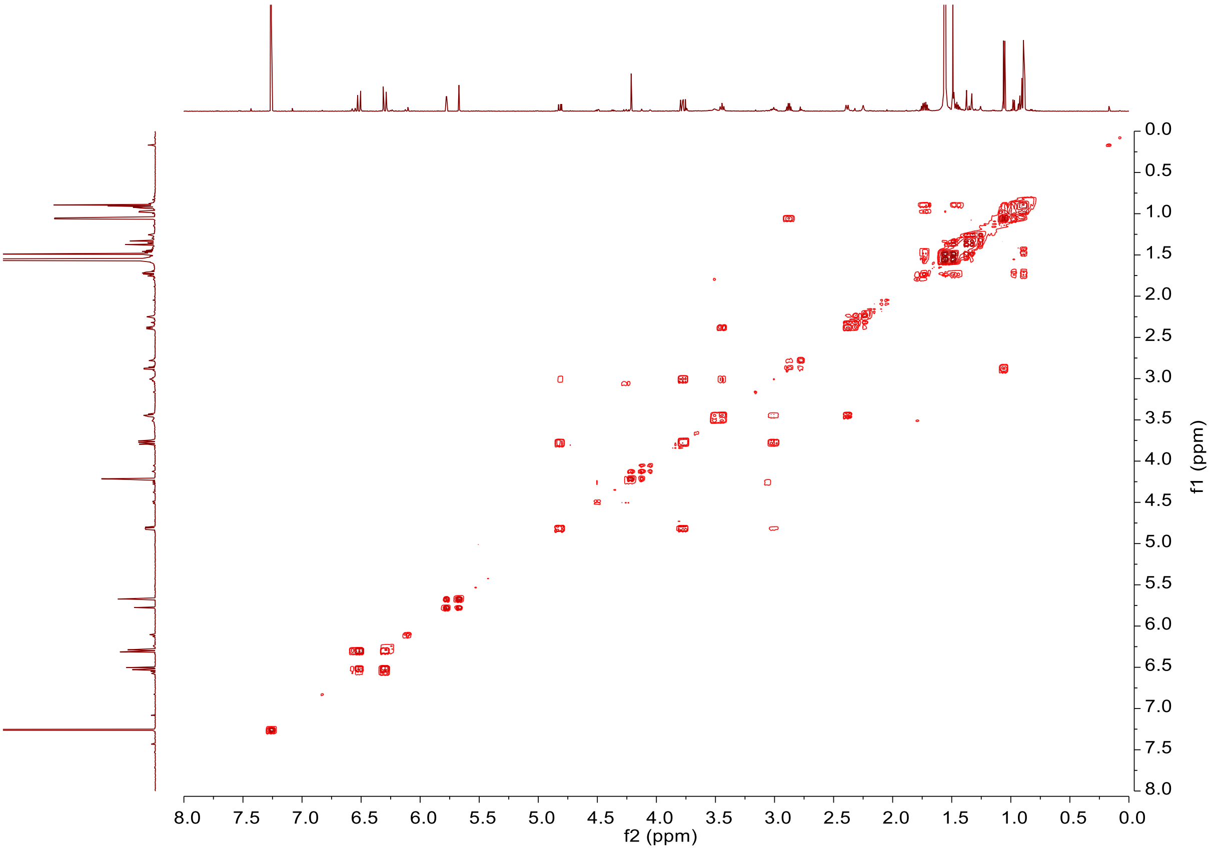

Figure S38. COSY spectrum of peniphilone C (**5**)

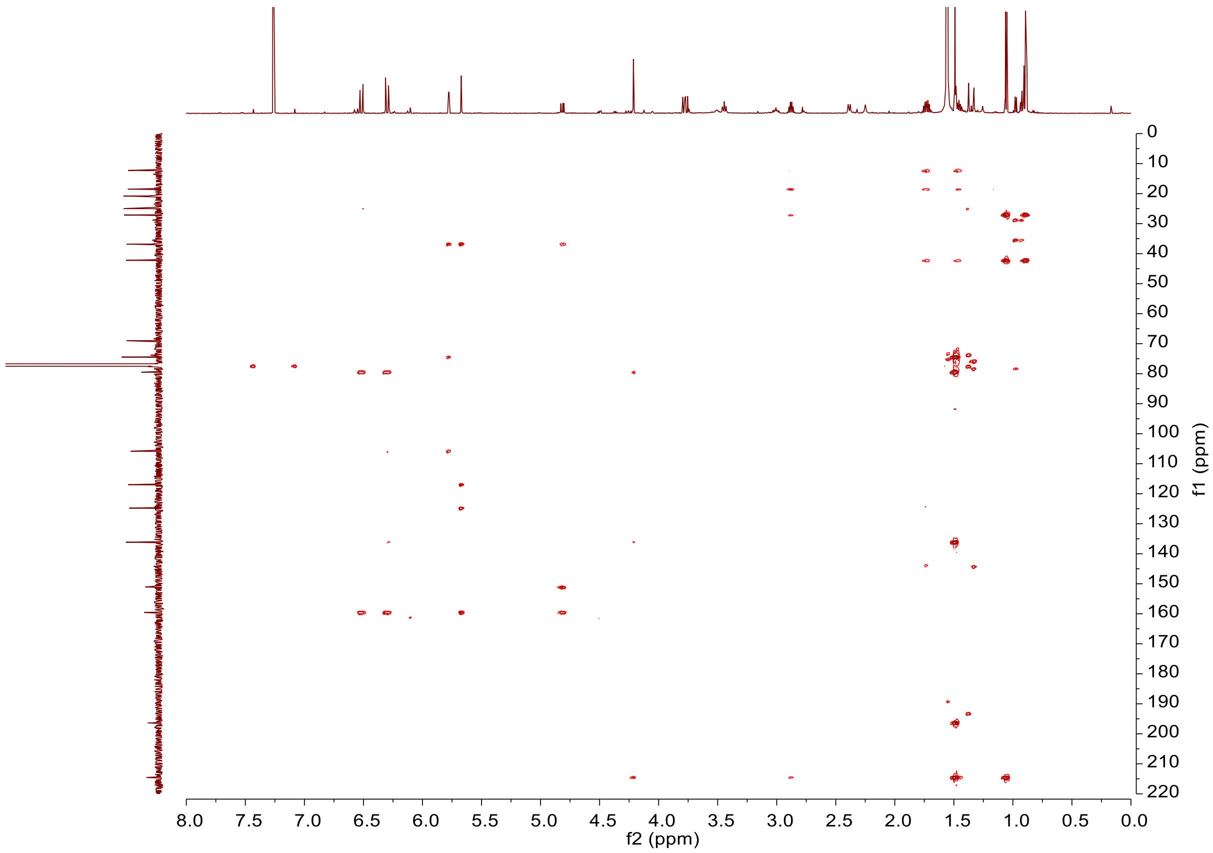

Figure S39. HMBC spectrum of peniphilone C (**5**)

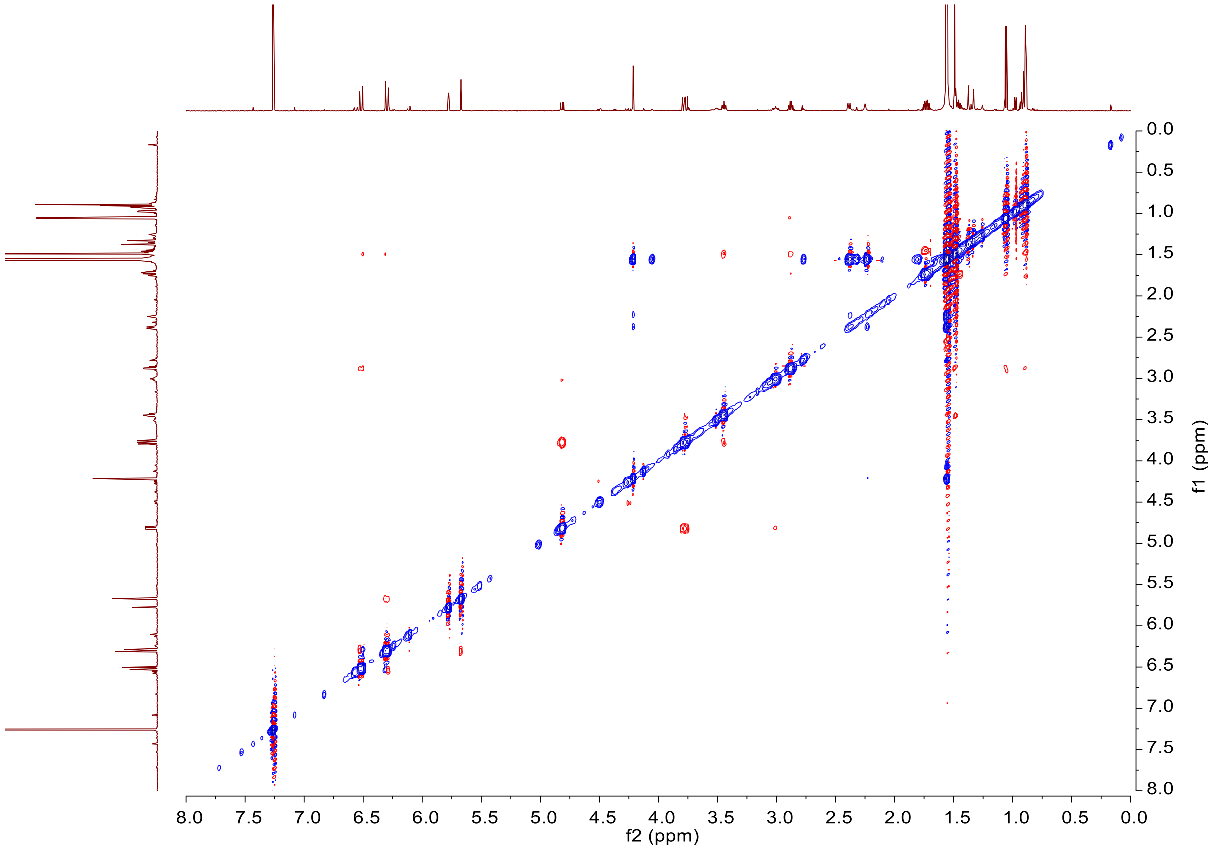

Figure S40. NOESY spectrum of peniphilone C (**5**)

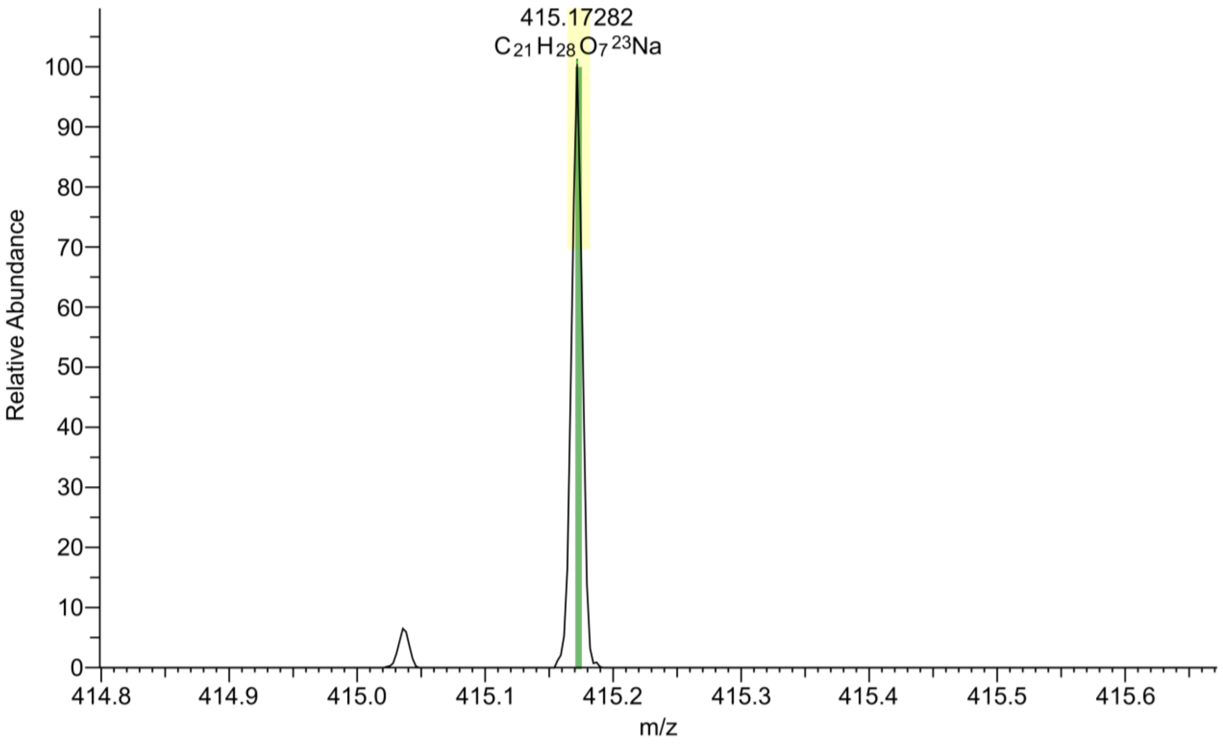

Peak Mass	Display Formula	S Fit	RDB	Delta [ppm]	Theo. mass	Pattern Cov. [%]	MSMS Matched...
415.17282	$C_{21}H_{28}O_7{}^{23}Na$	49.21923157738 48	7.50	0.23	415.17272	99.63	(Collection)

Figure S41. HRESIMS spectrum of peniphilone D (**6**)

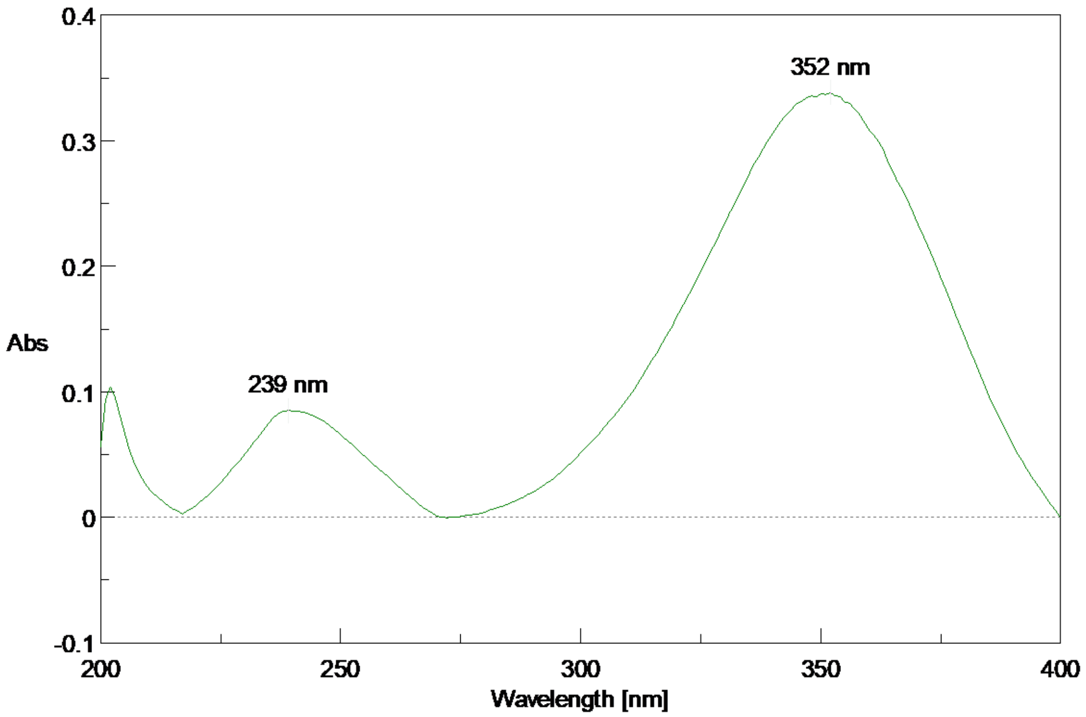

Figure S42. UV spectrum of peniphilone D (**6**)

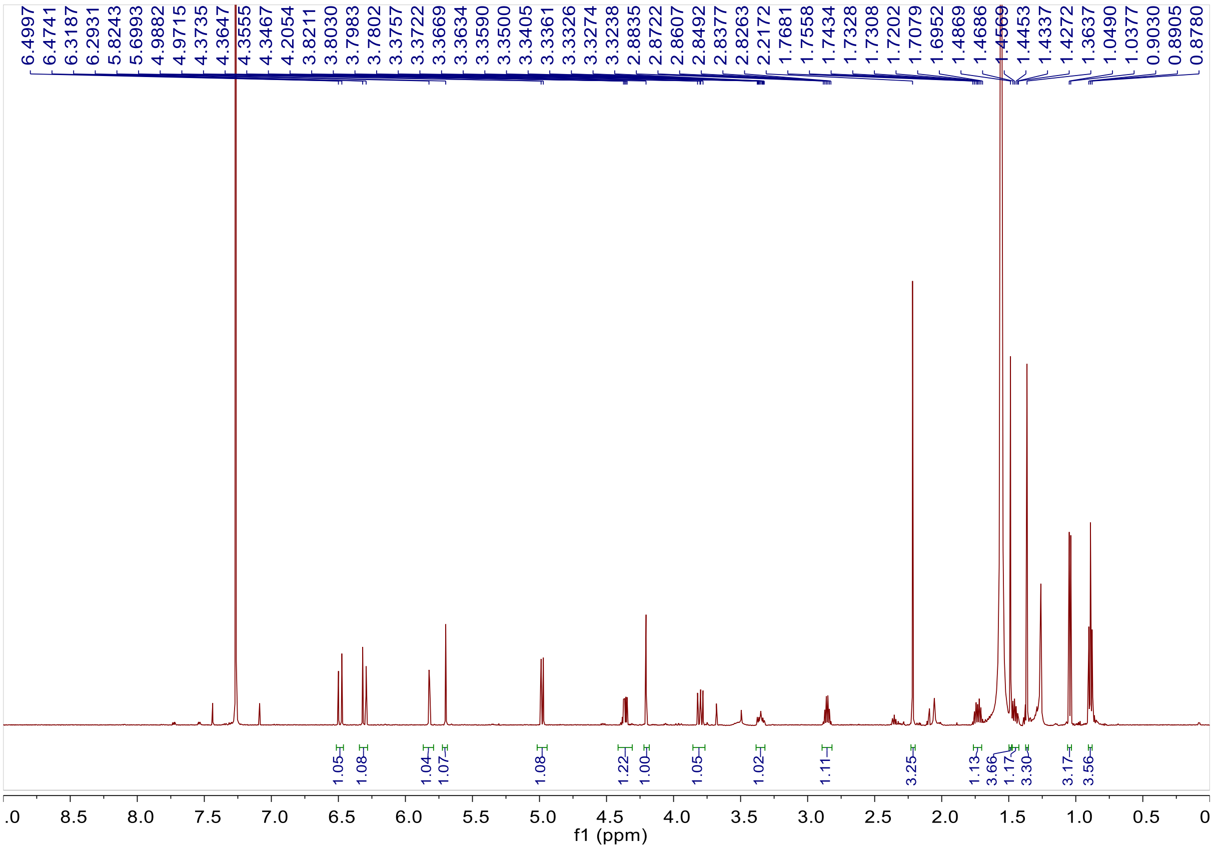

Figure S43. ^{1}H NMR spectrum (600 MHz, CDCl$_3$) of peniphilone D (**6**)

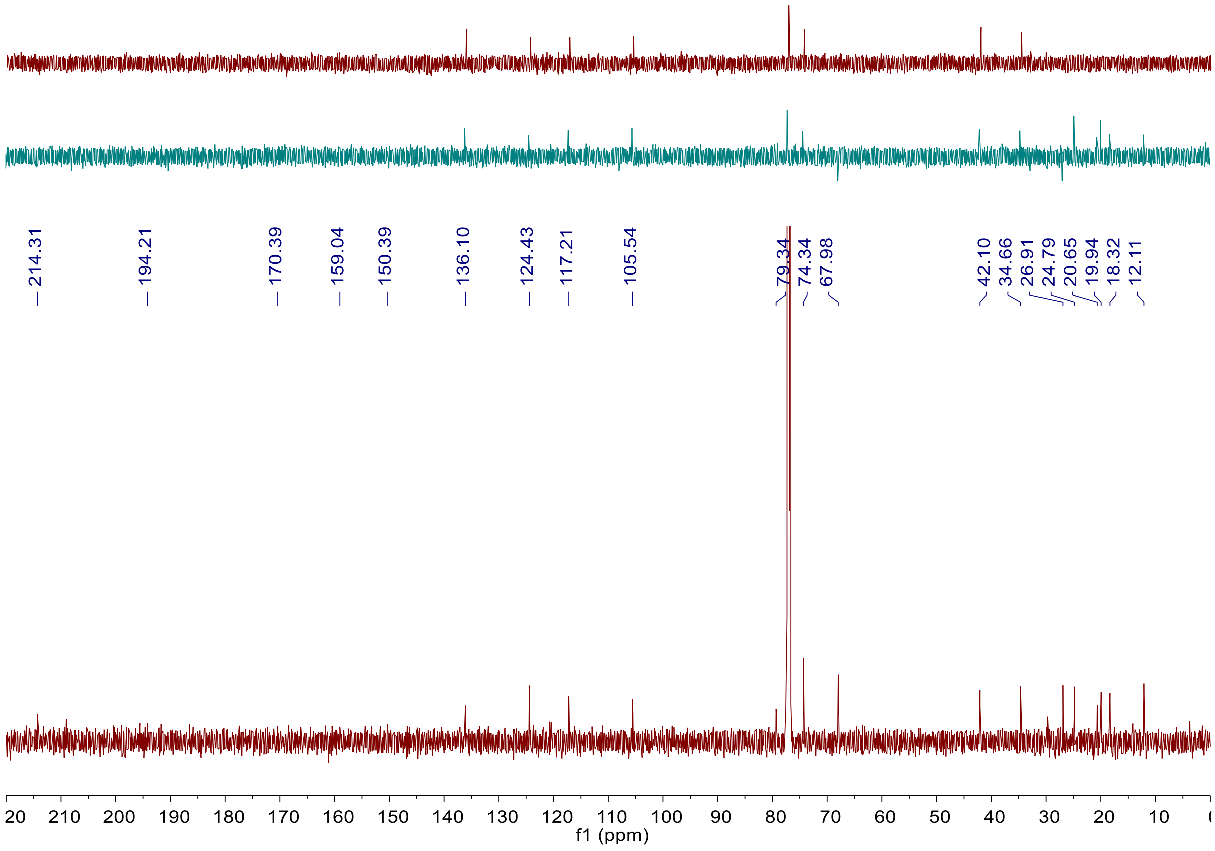

Figure S44. ^{13}C NMR spectrum (150 MHz, CDCl$_3$) of peniphilone D (**6**)

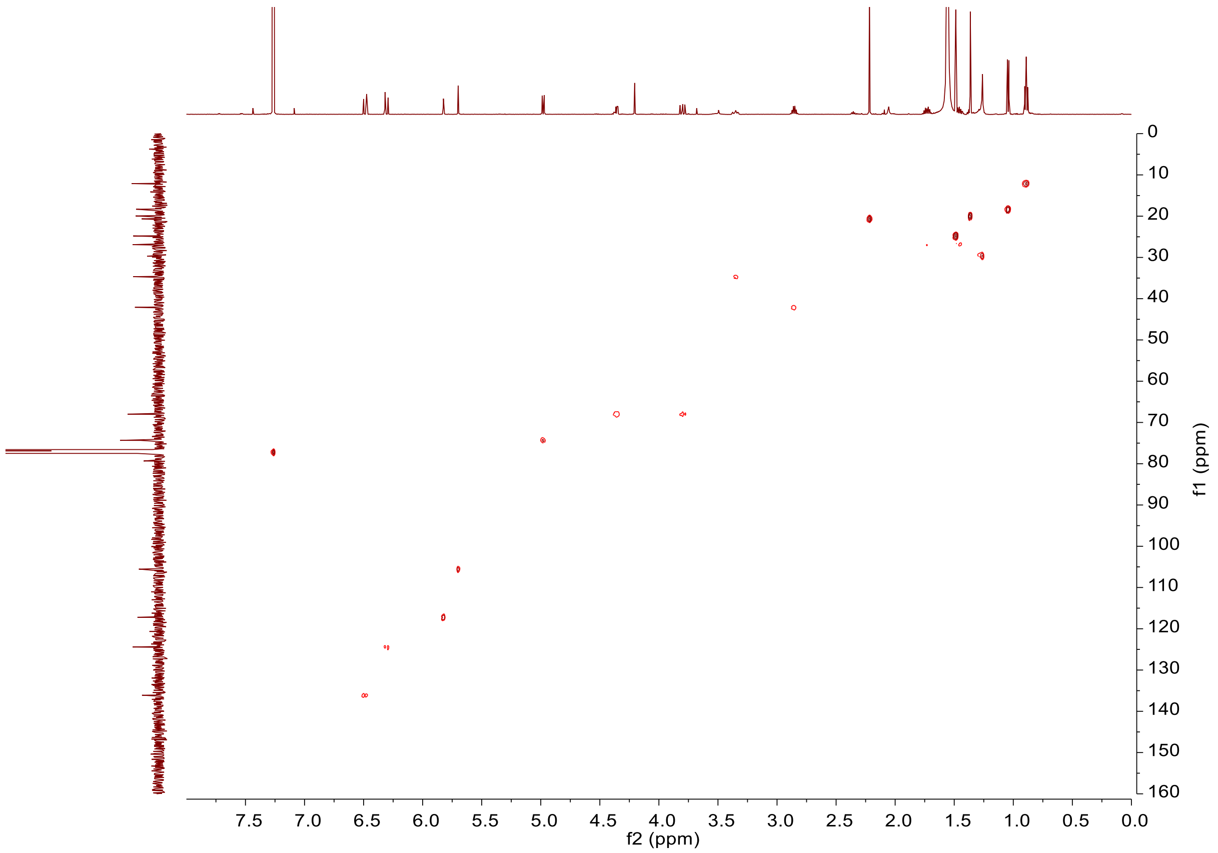

Figure S45. ^{1}H-^{13}C HSQC spectrum of peniphilone D (**6**)

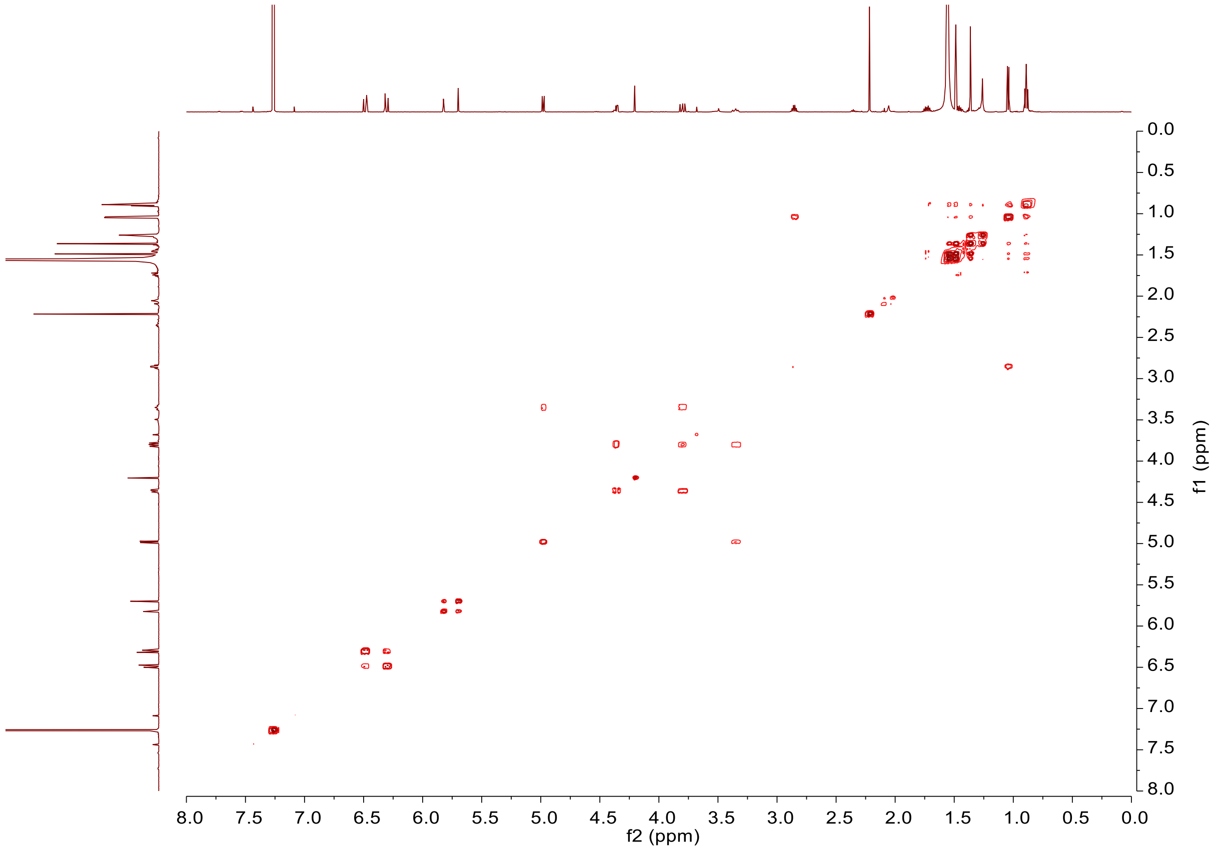

Figure S46. COSY spectrum of peniphilone D (**6**)

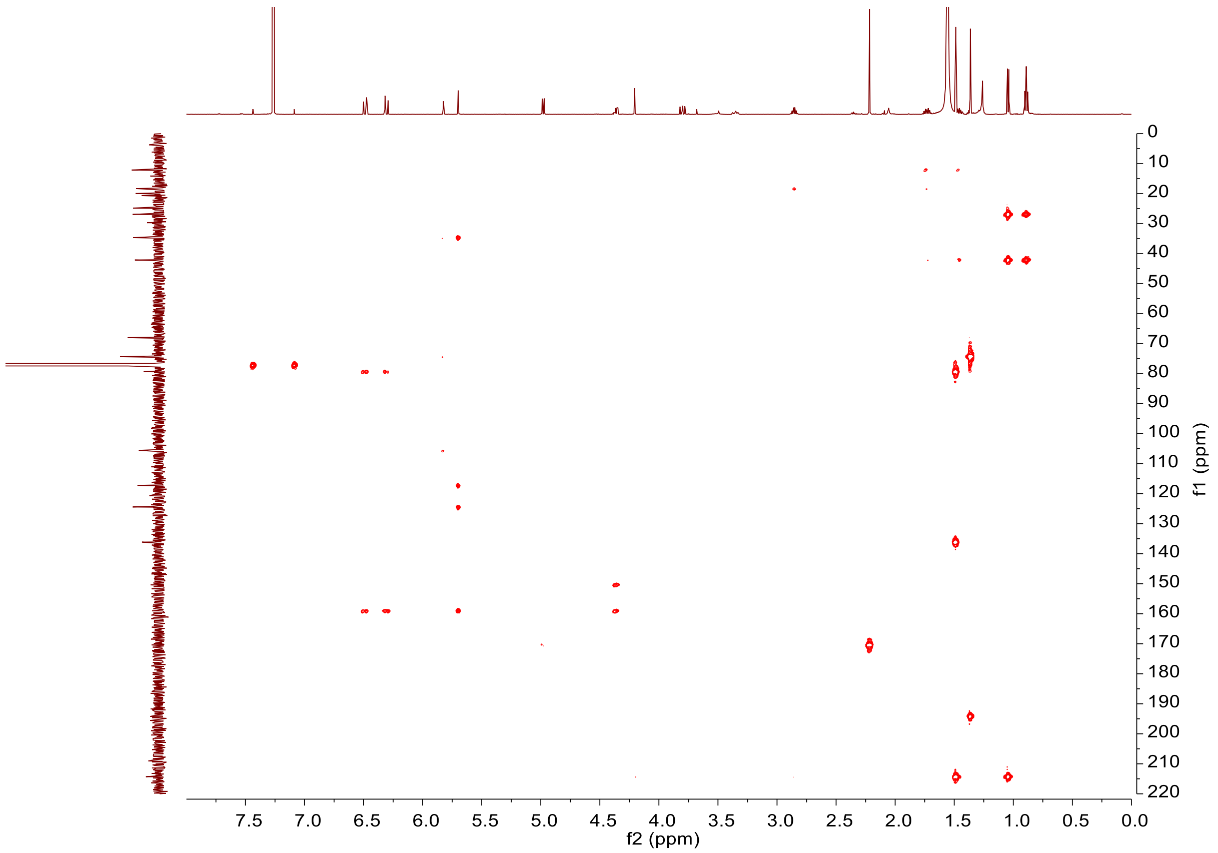

Figure S47. HMBC spectrum of peniphilone D (**6**)

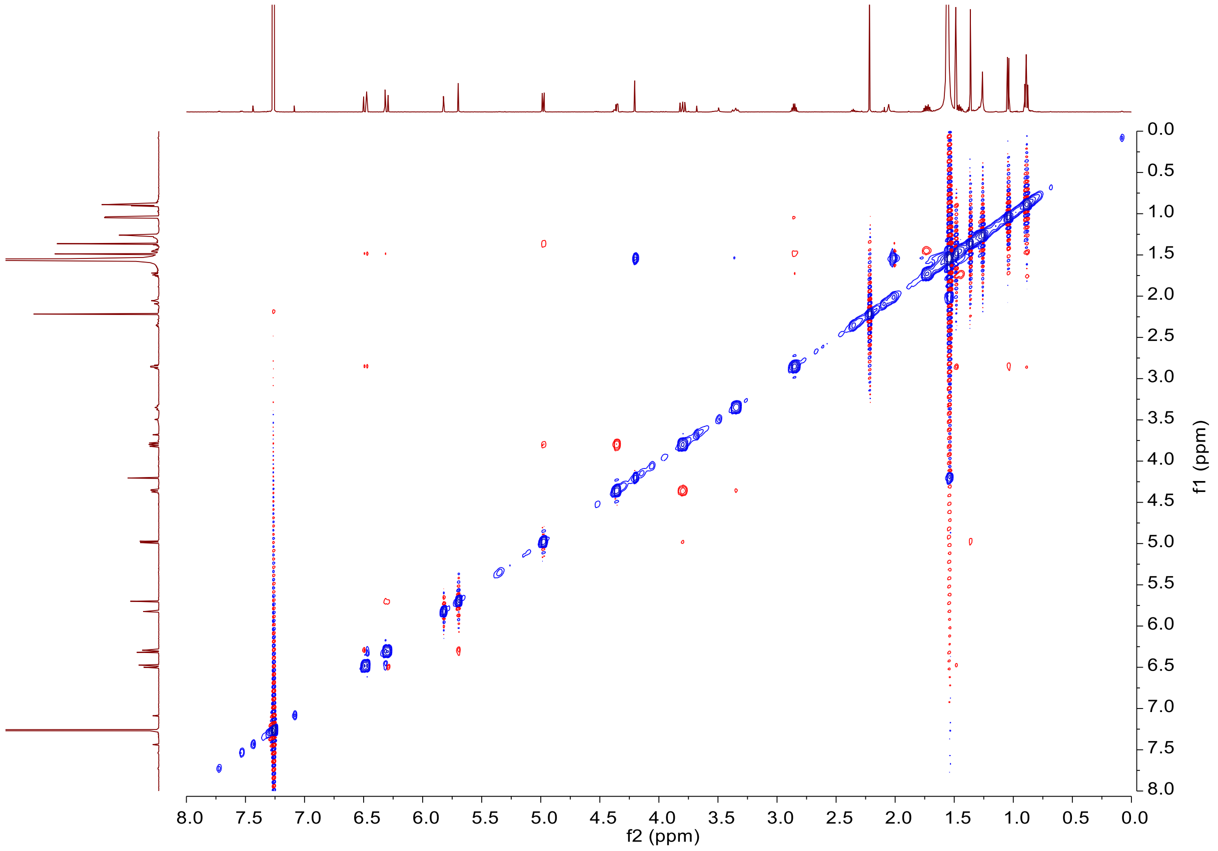

Figure S48. NOESY spectrum of peniphilone D (**6**)

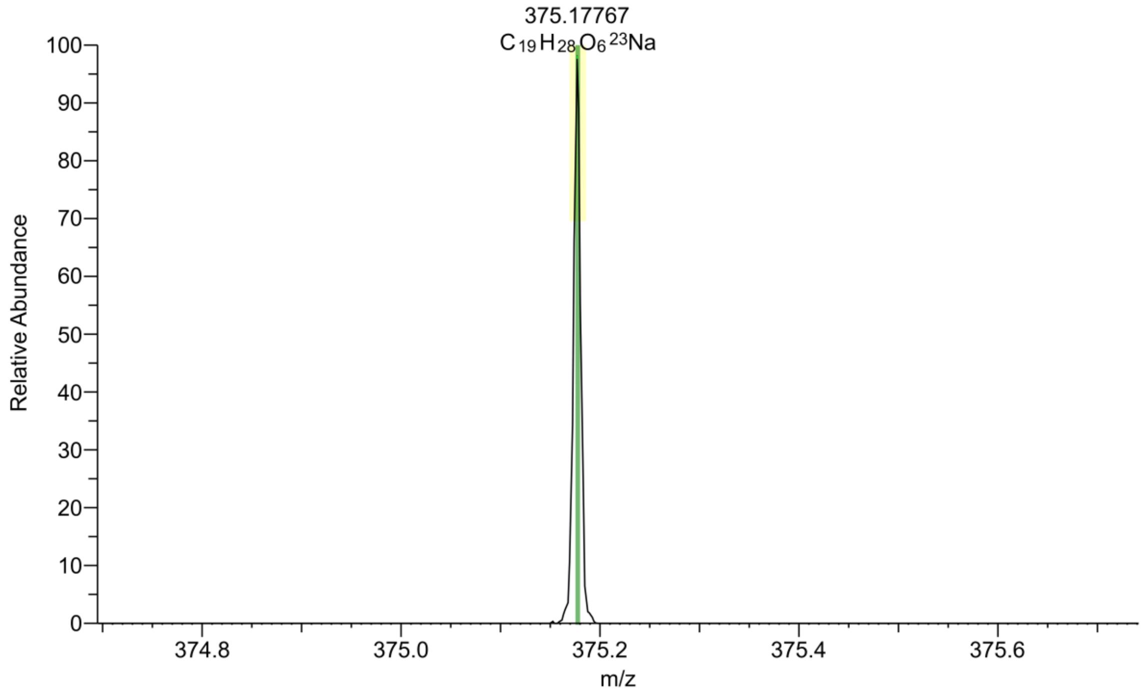

Peak Mass	Display Formula	S Fit	RDB	Delta [ppm]	Theo. mass	Pattern Cov. [%]	MSMS Matched...
375.17767	$C_{19}H_{28}O_6{}^{23}Na$	86.42293084635 33	5.50	-0.36	375.17781	100	(Collection)

Figure S49. HRESIMS spectrum of peniphilone E (**7**)

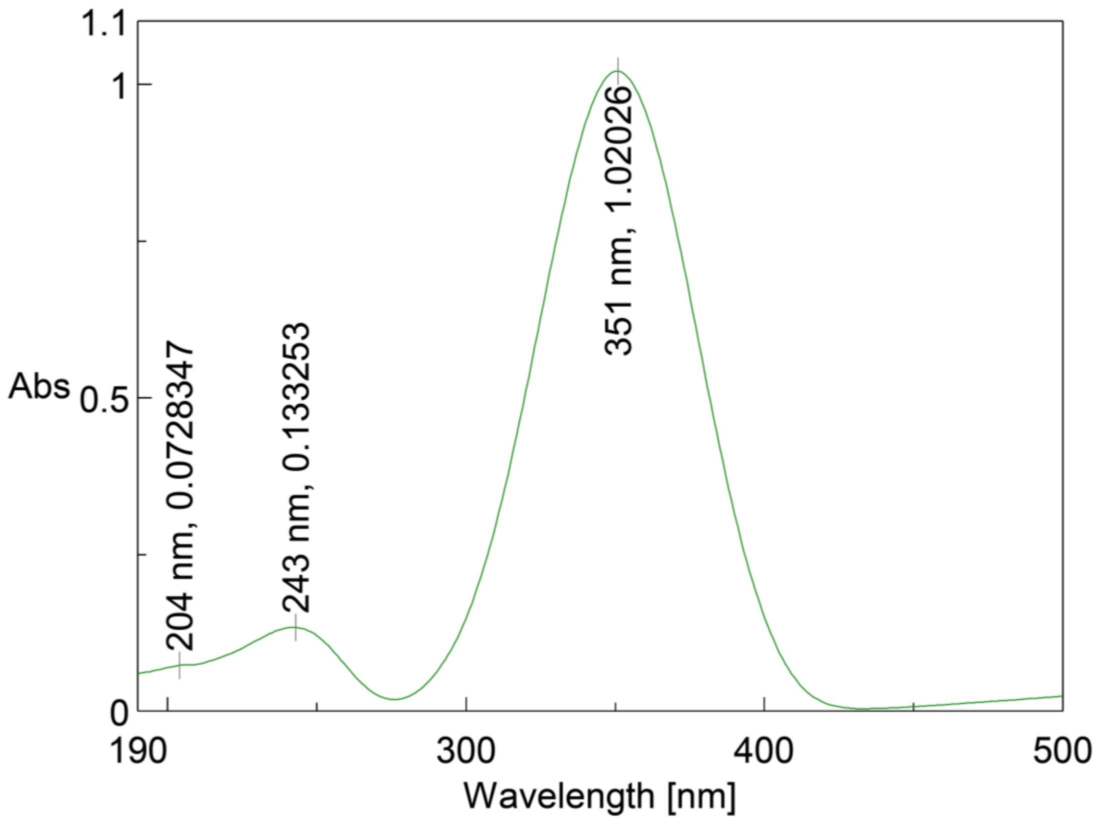

Figure S50. UV spectrum of peniphilone E (**7**)

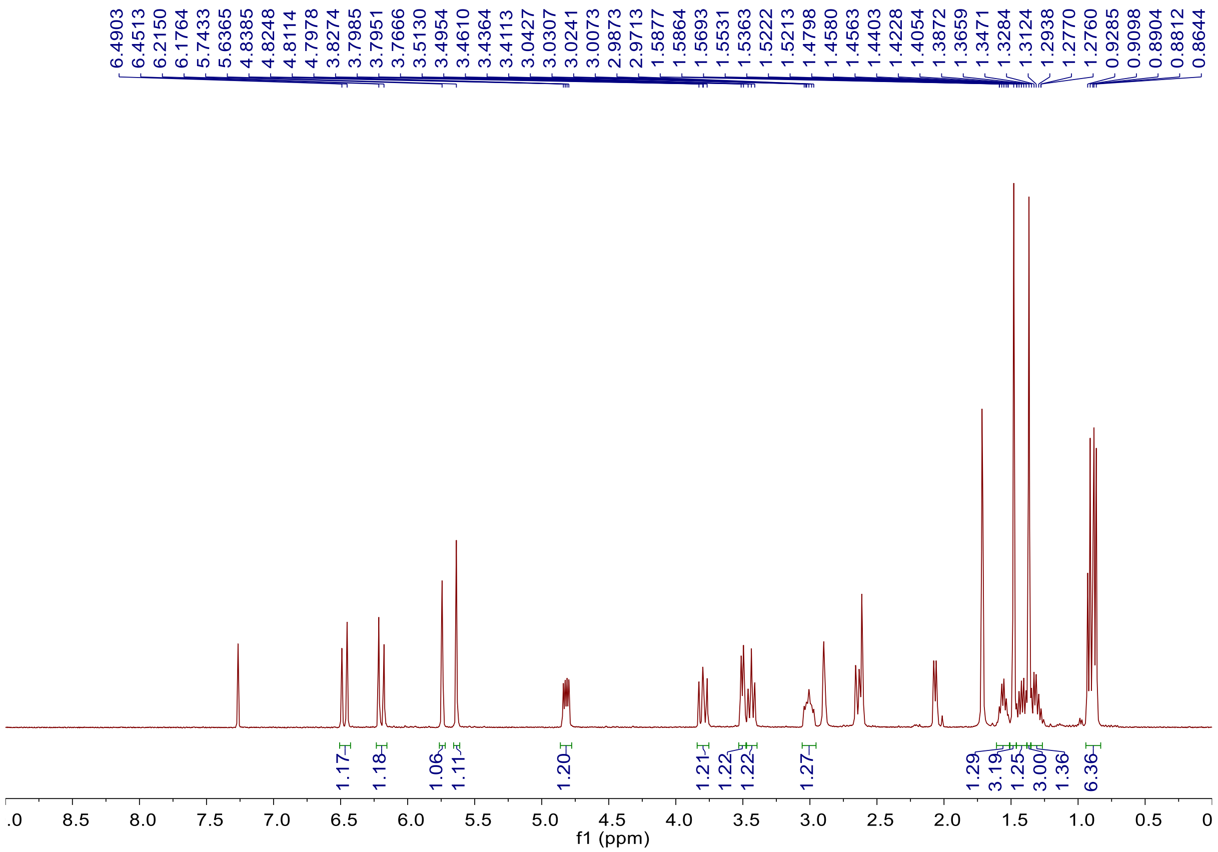

Figure S51. ^{1}H NMR spectrum (400 MHz, $CDCl_3$) of peniphilone E (**7**)

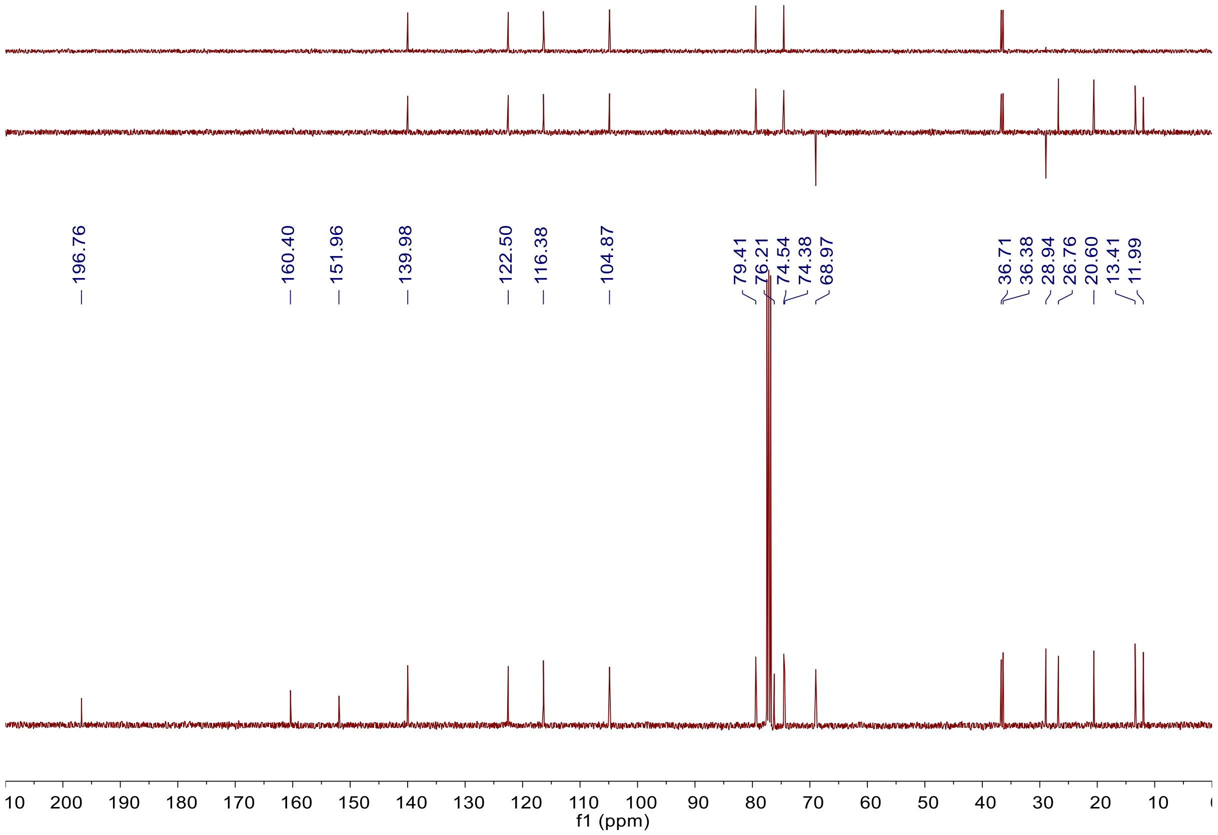

Figure S52. ^{13}C NMR spectrum (100 MHz, $CDCl_3$) of peniphilone E (**7**)

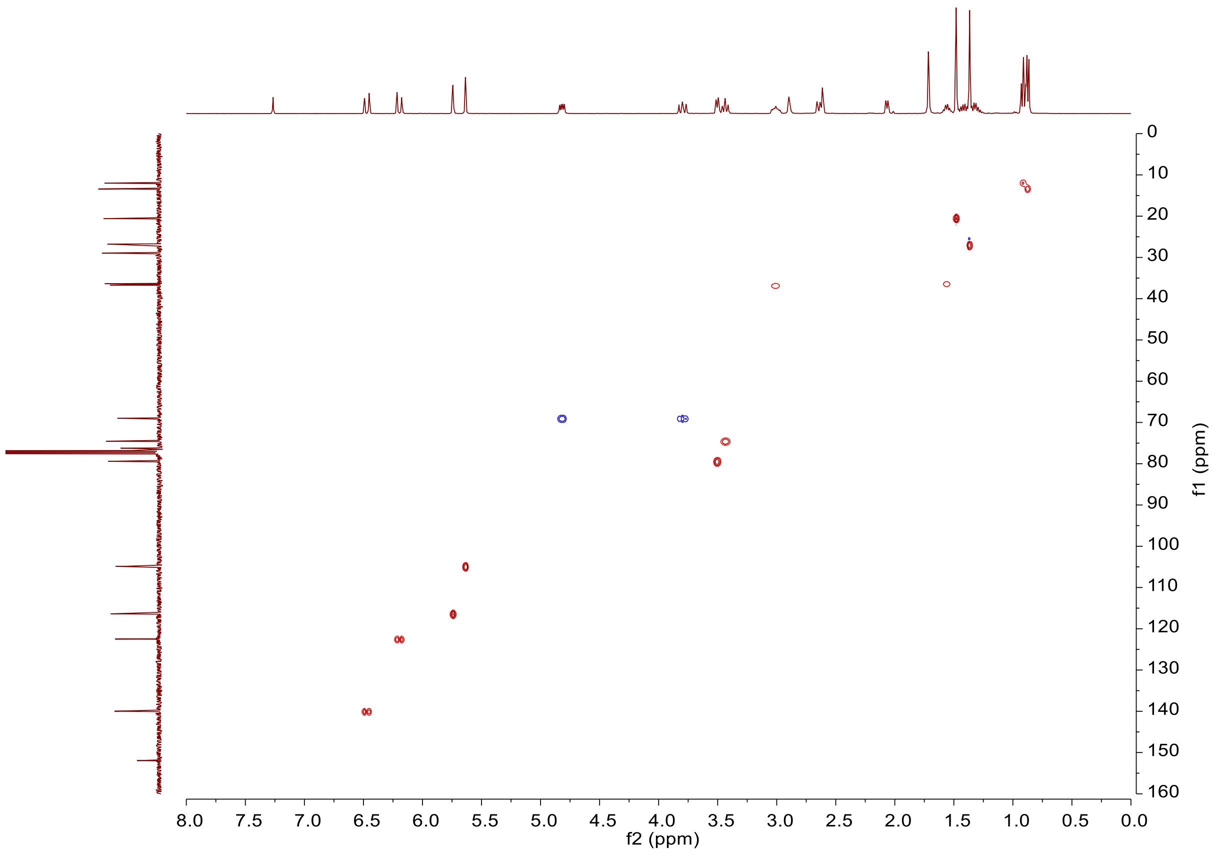

Figure S53. ^{1}H-^{13}C HSQC spectrum of peniphilone E (**7**)

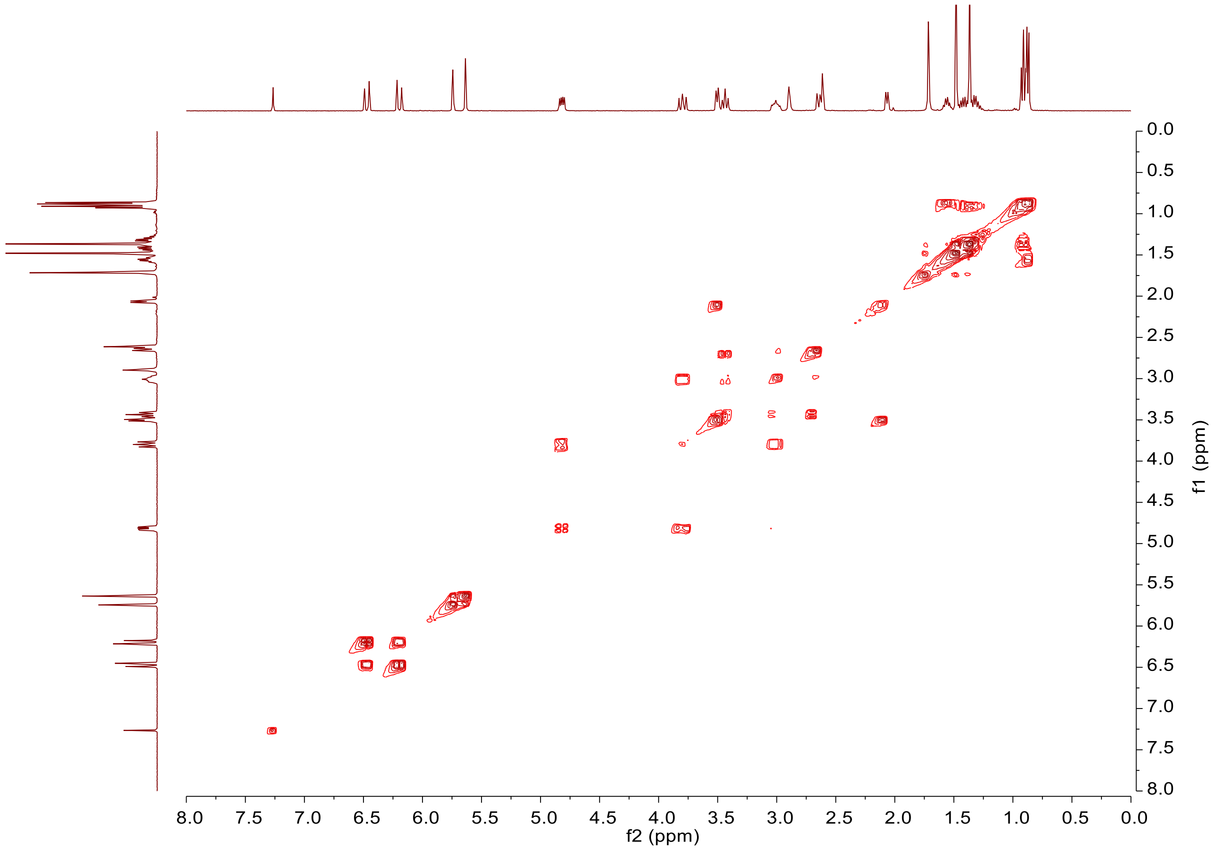

Figure S54. COSY spectrum of peniphilone E (**7**)

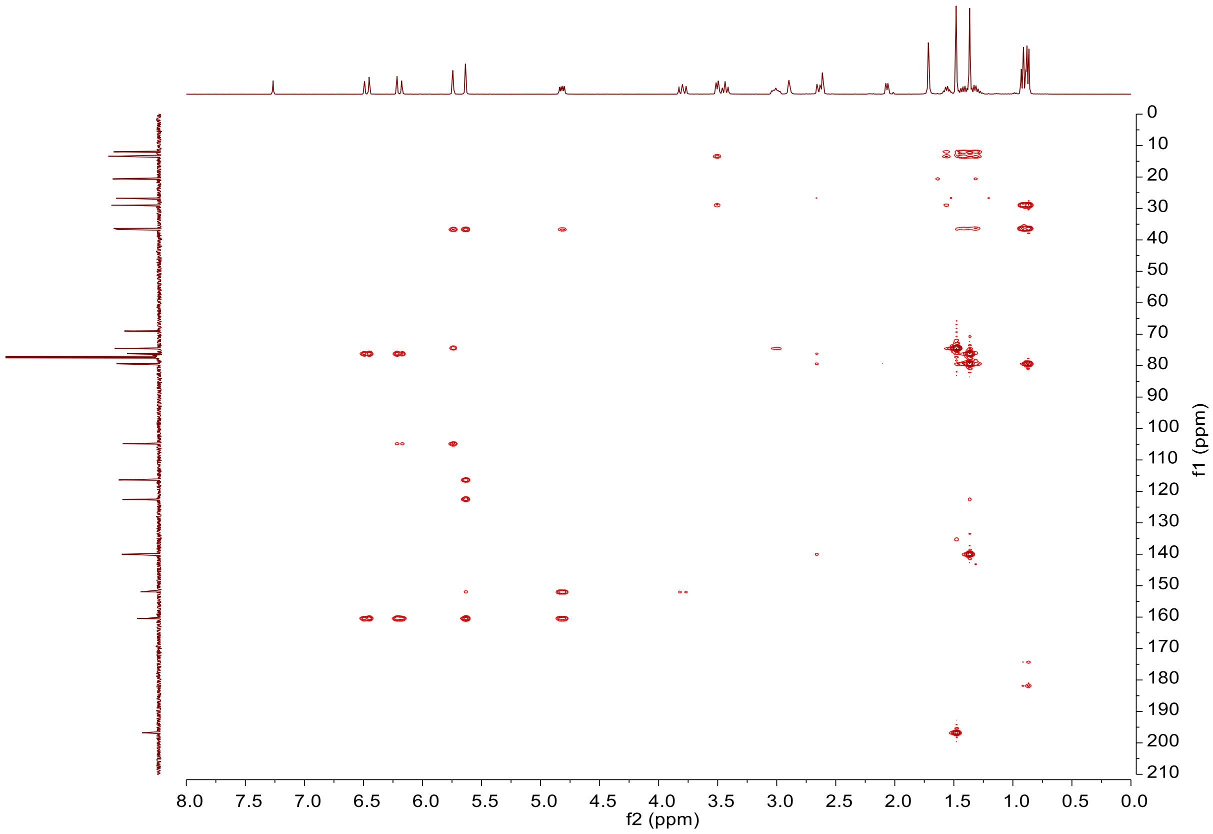

Figure S55. HMBC spectrum of peniphilone E (**7**)

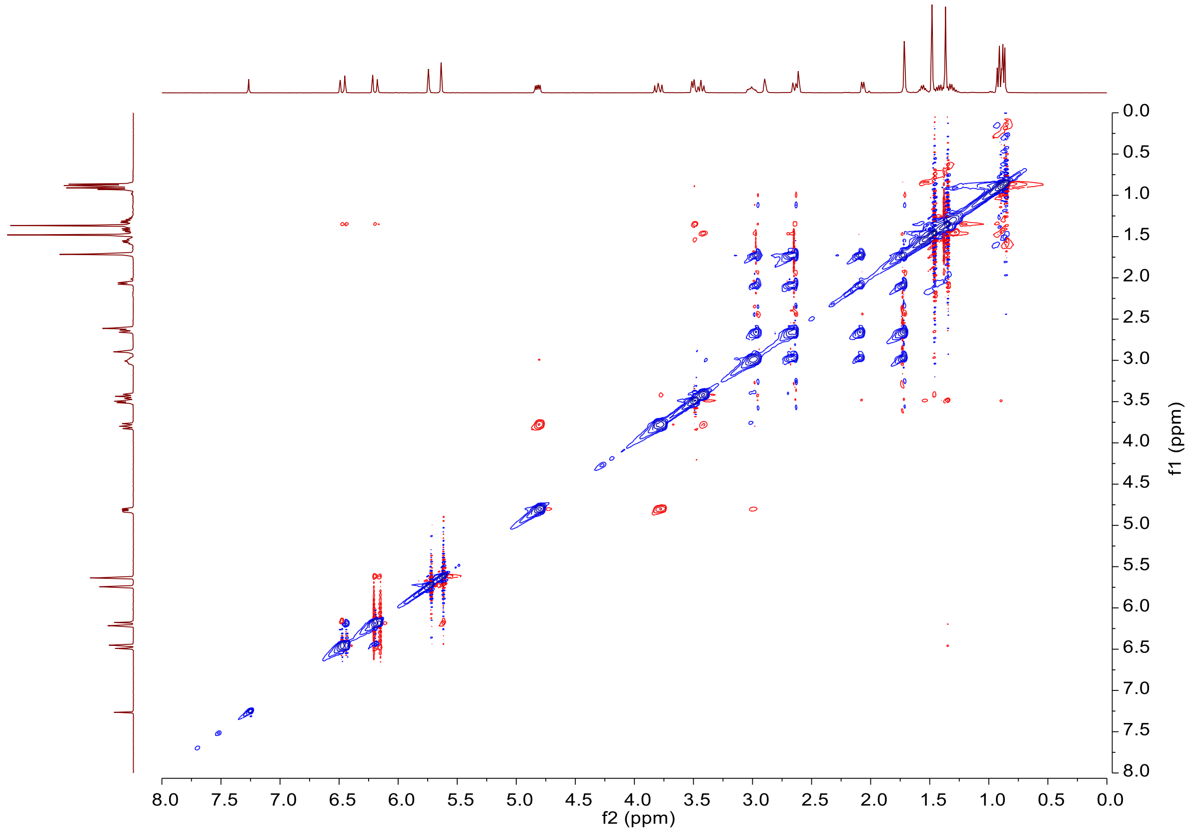

Figure S56. NOESY spectrum of peniphilone E (**7**)

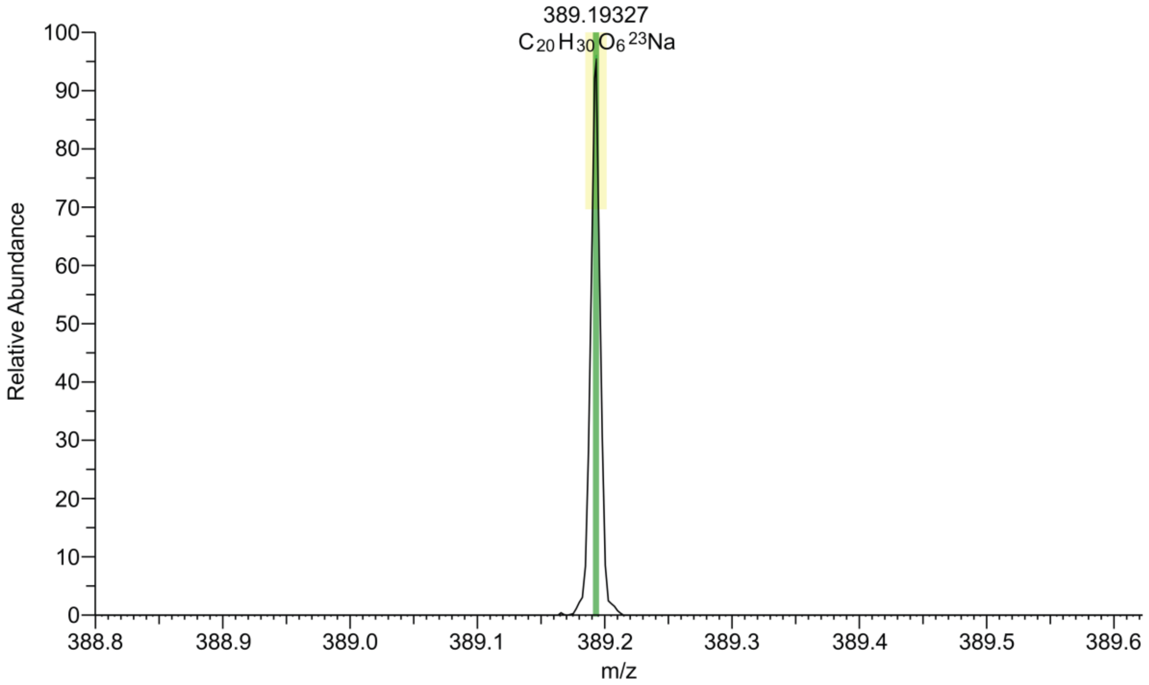

Peak Mass	Display Formula	S Fit	RDB	Delta [ppm]	Theo. mass	Pattern Cov. [%]	MSMS Matched...
389.19327	$C_{20}H_{30}O_6{}^{23}Na$	92.12058693473 98	5.50	-0.49	389.19346	100	(Collection)

Figure S57. HRESIMS spectrum of peniphilone F (**8**)

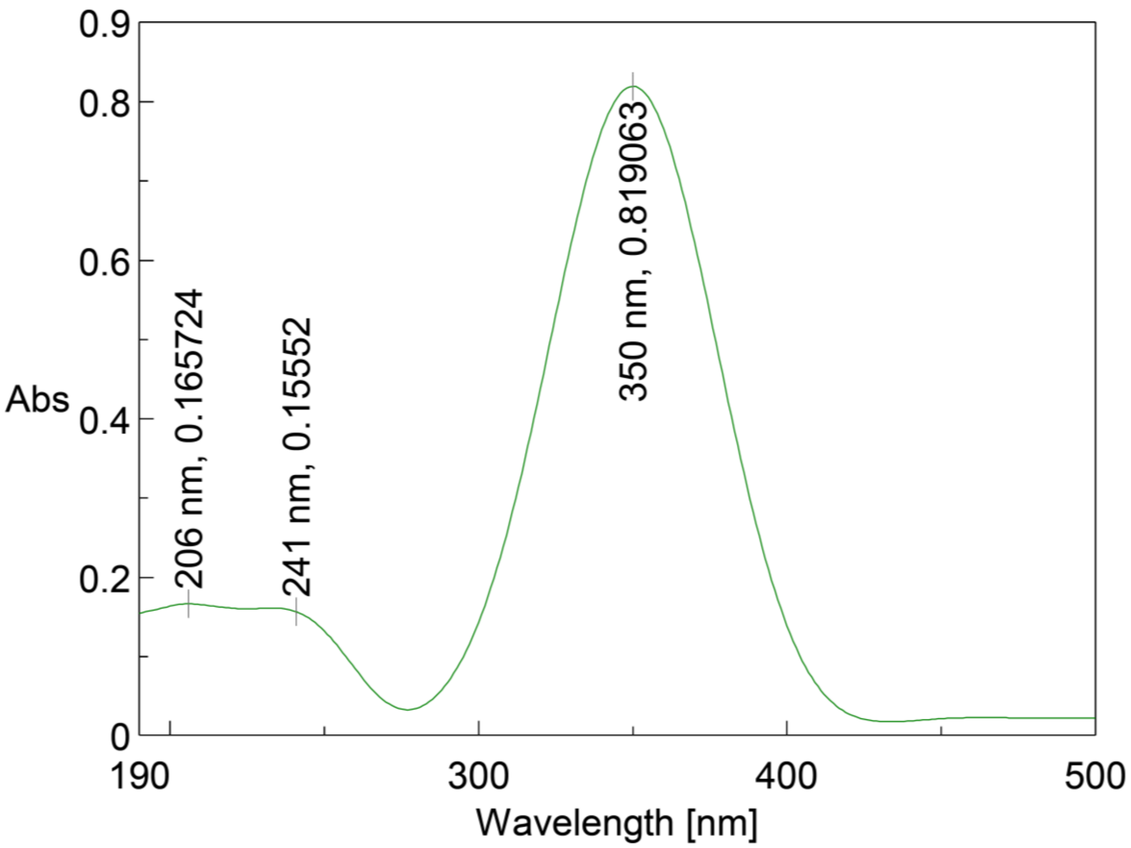

Figure S58. UV spectrum of peniphilone F (**8**)

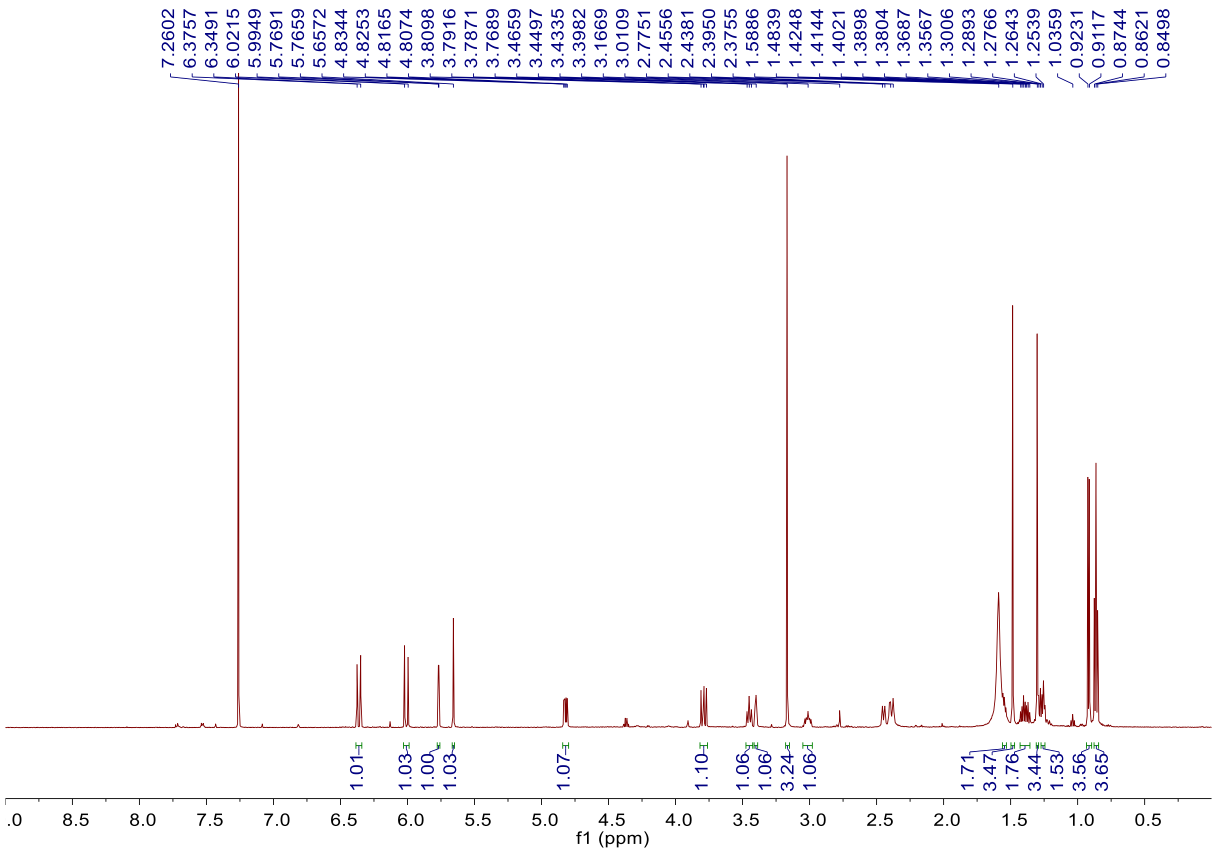

Figure S59. ^{1}H NMR spectrum (600 MHz, $CDCl_3$) of peniphilone F (**8**)

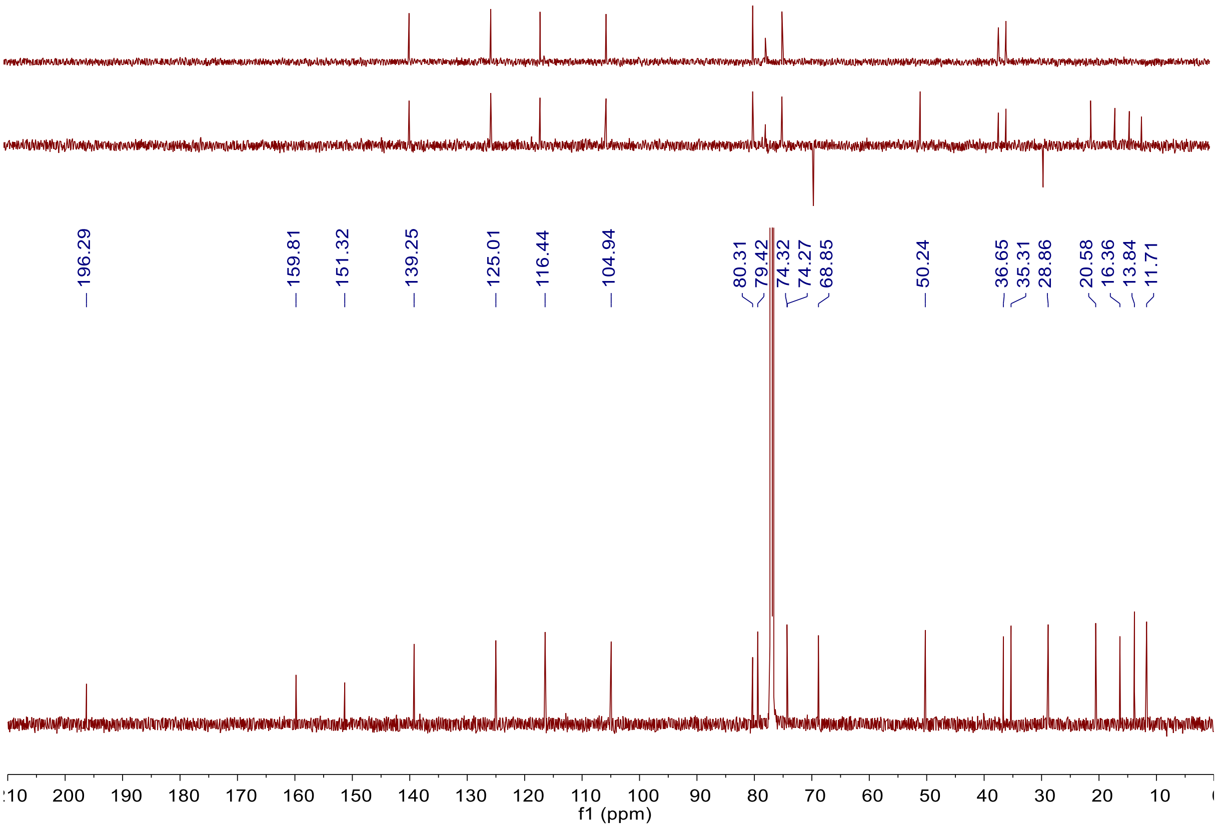

Figure S60. ^{13}C NMR spectrum (150 MHz, $CDCl_3$) of peniphilone F (**8**)

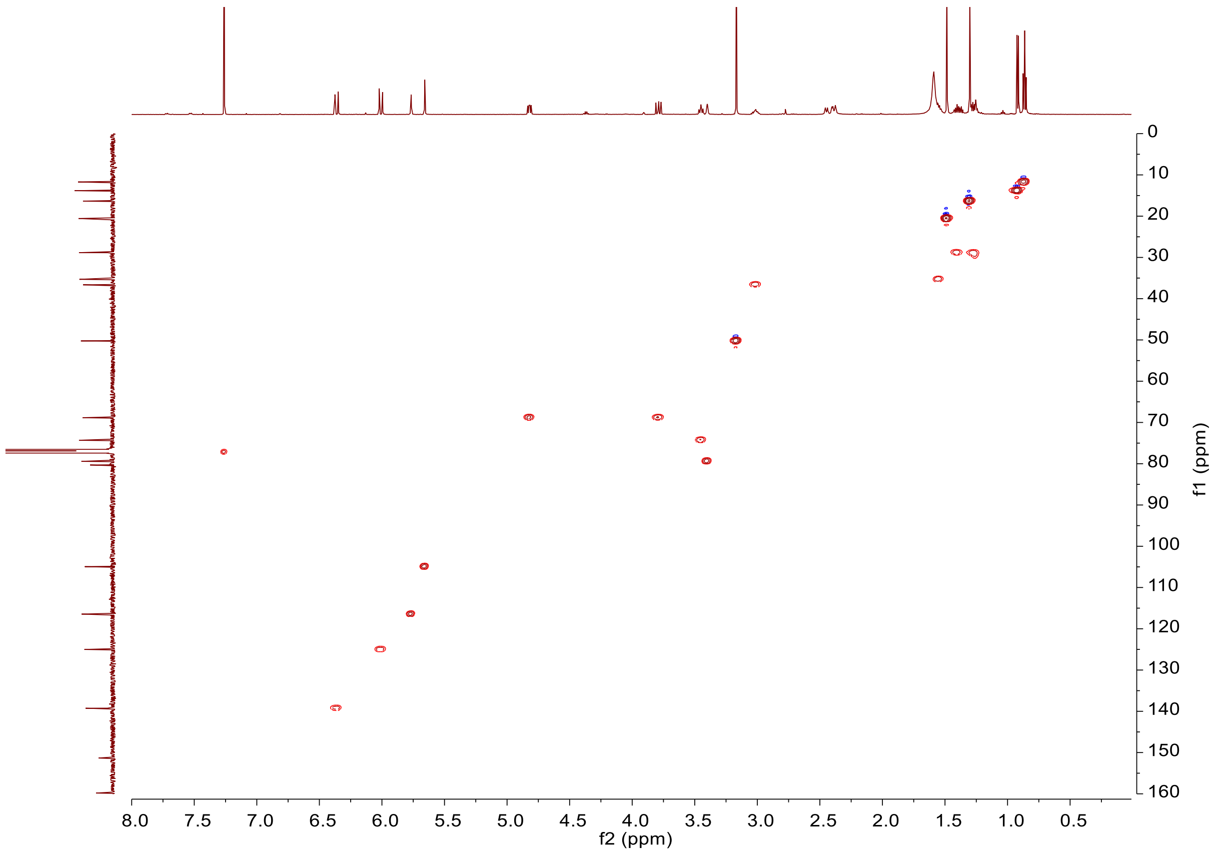

Figure S61. ^{1}H-^{13}C HSQC spectrum of peniphilone F (**8**)

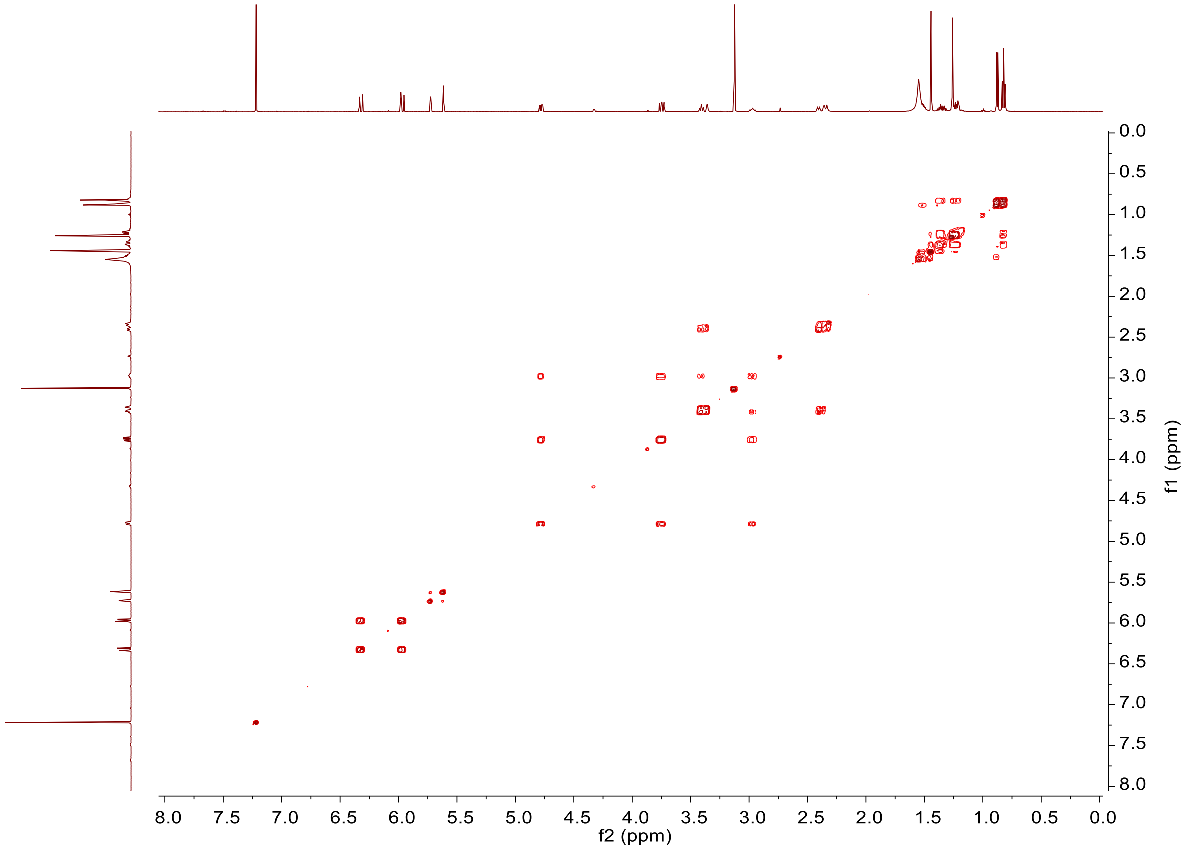

Figure S62. COSY spectrum of peniphilone F (**8**)

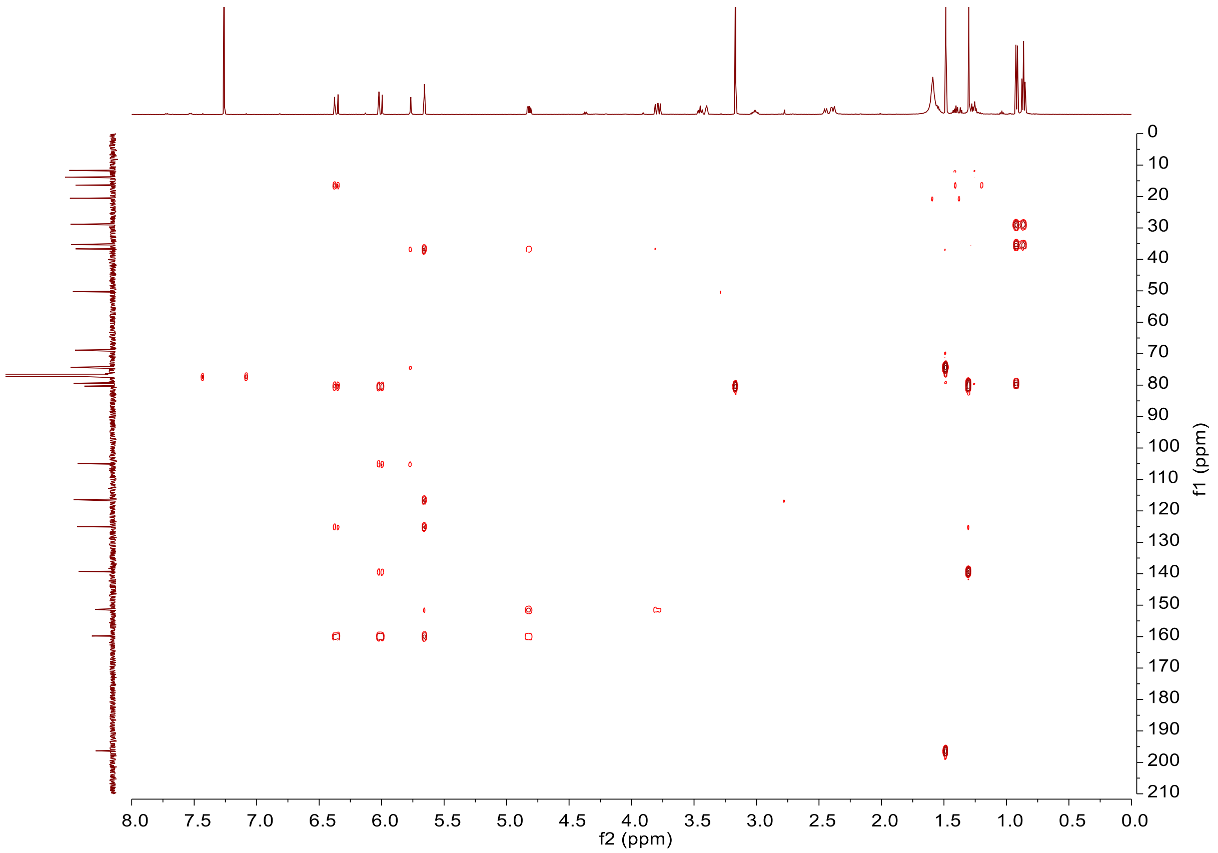

Figure S63. HMBC spectrum of peniphilone F (**8**)

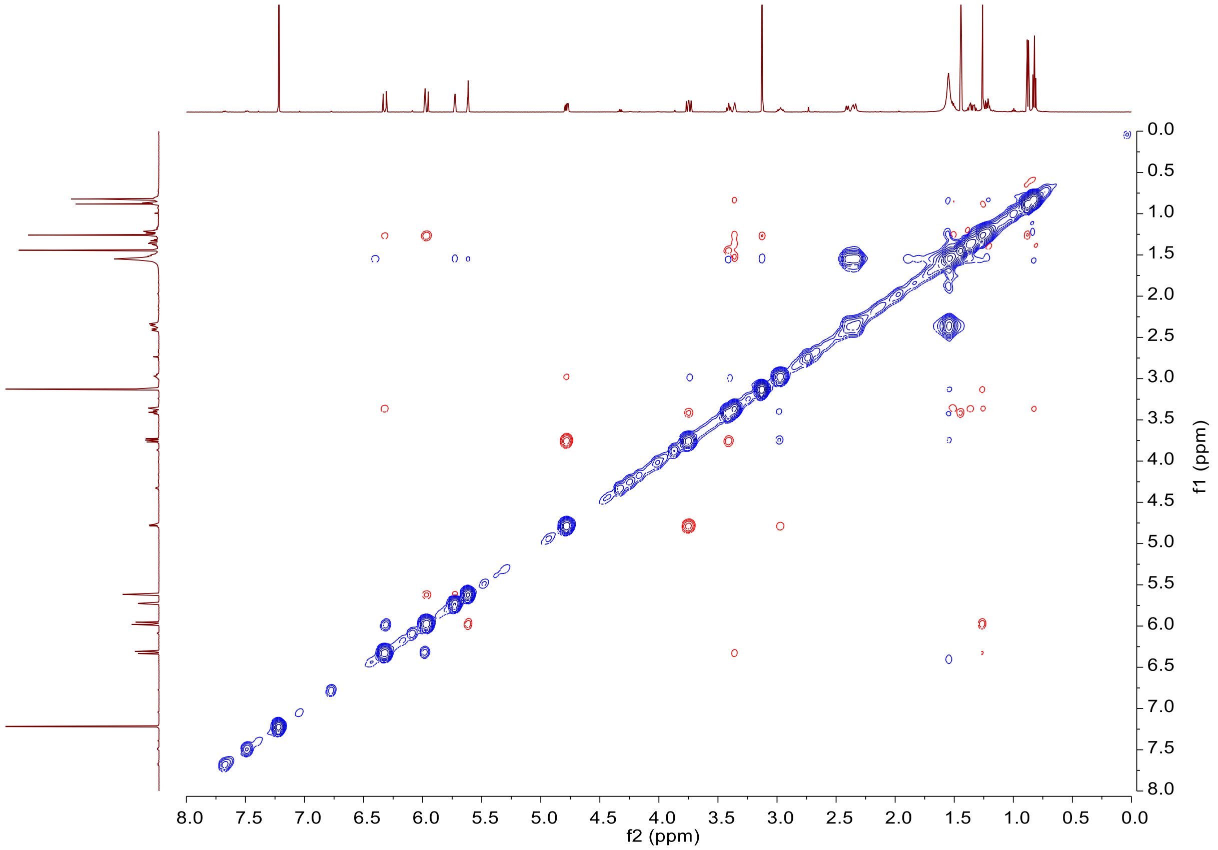

Figure S64. NOESY spectrum of peniphilone F (**8**)

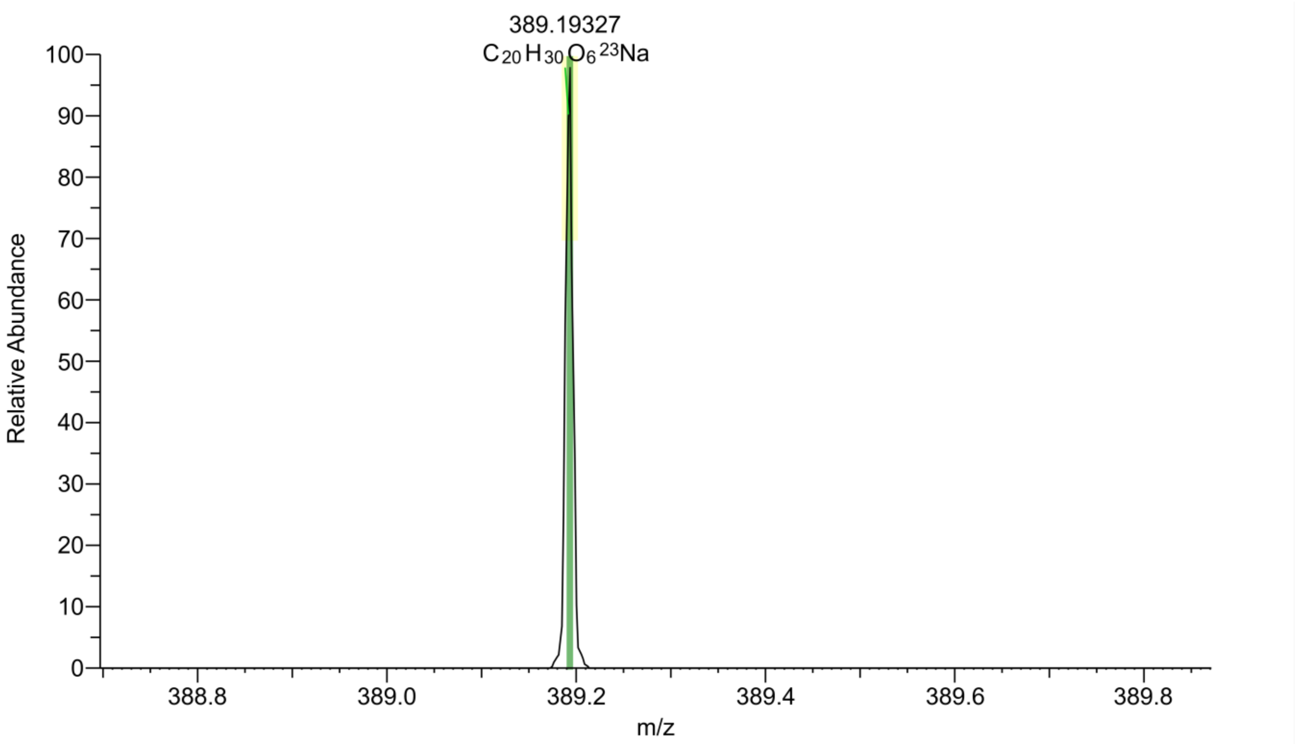

Peak Mass	Display Formula	S Fit	RDB	Delta [ppm]	Theo. mass	Pattern Cov. [%]	MSMS Matched...
389.19327	$C_{20}H_{30}O_6{}^{23}Na$	49.40814547901 15	5.50	-0.49	389.19346	99.69	(Collection)

Figure S65. HRESIMS spectrum of peniphilone G (**9**)

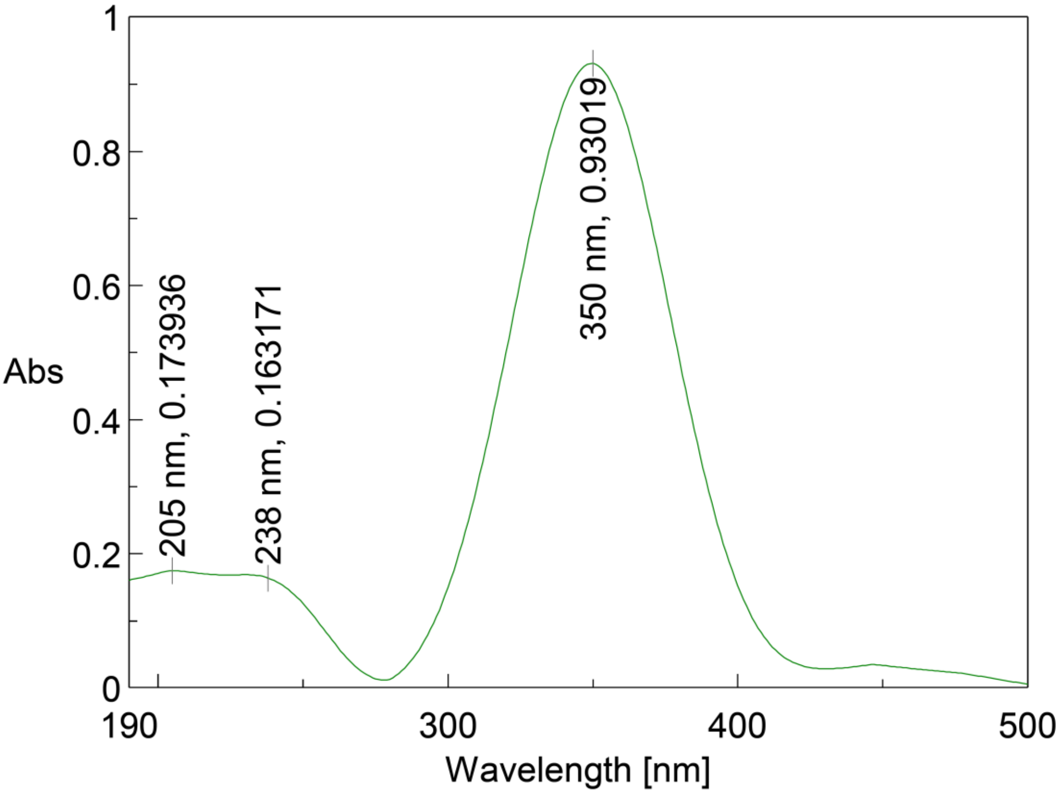

Figure S66. UV spectrum of peniphilone G (**9**)

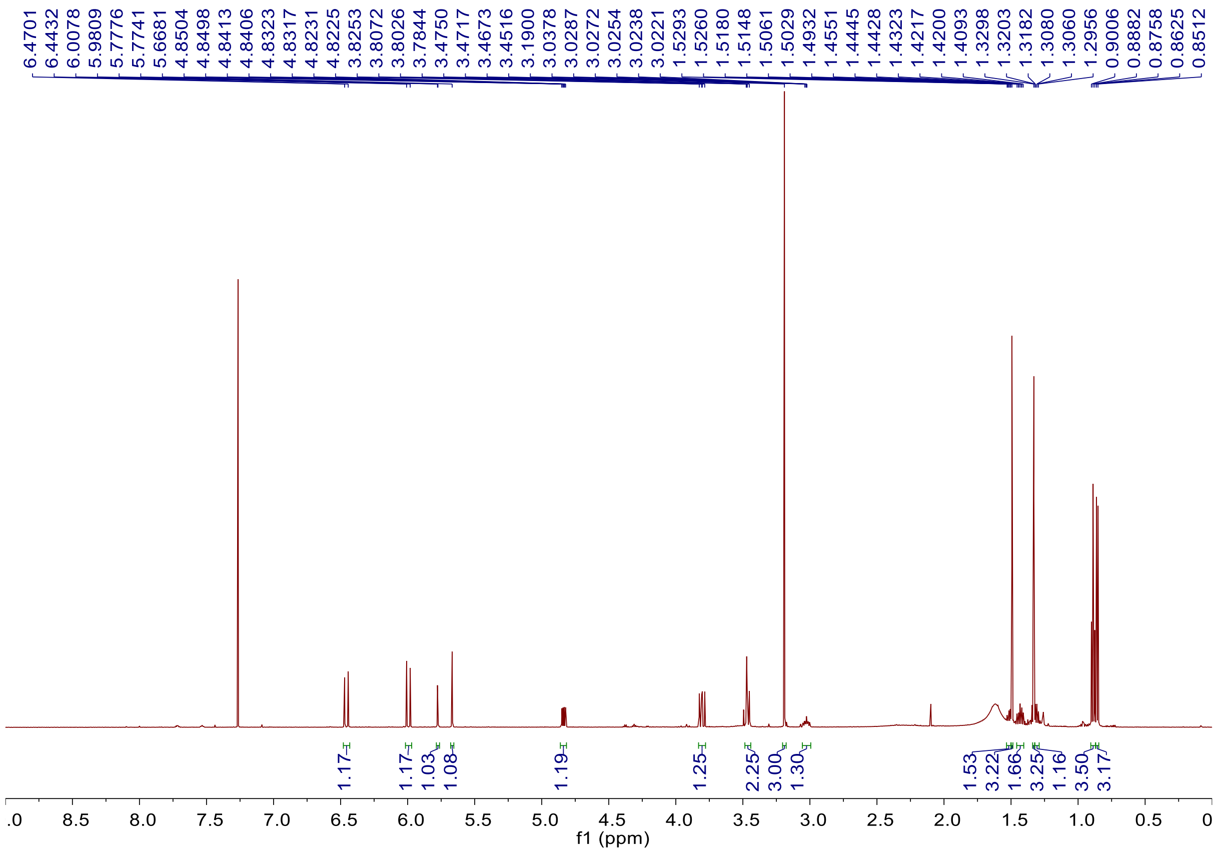

Figure S67. ^{1}H NMR spectrum (600 MHz, CDCl$_3$) of peniphilone G (**9**)

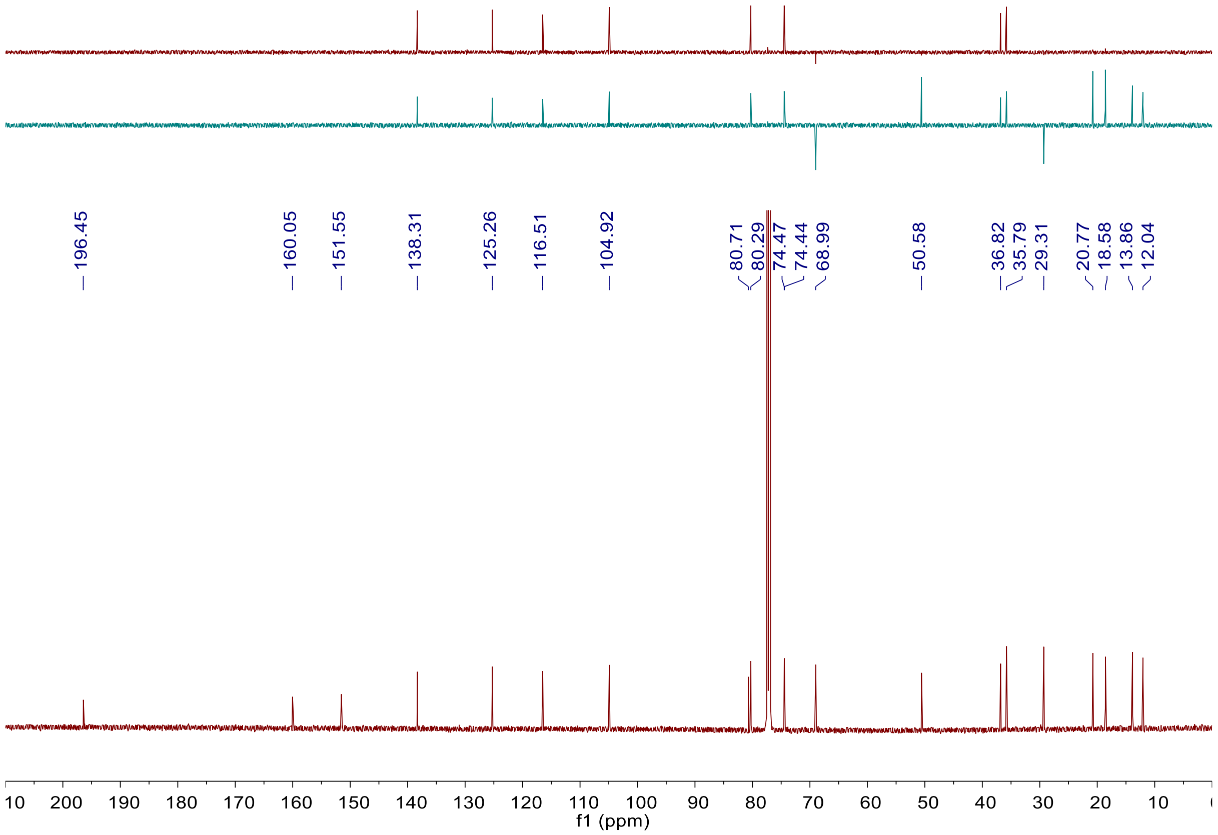

Figure S68. ^{13}C NMR spectrum (150 MHz, CDCl$_3$) of peniphilone G (**9**)

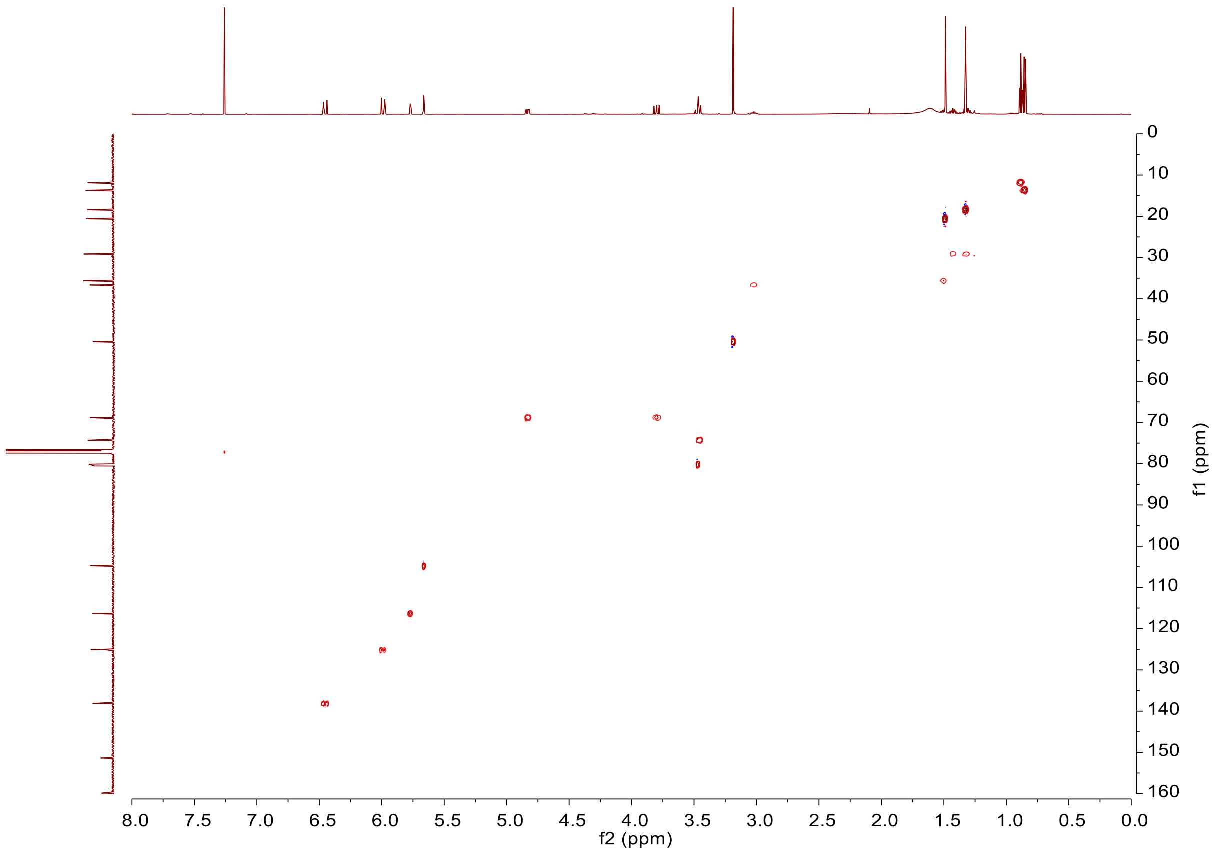

Figure S69. ^{1}H-^{13}C HSQC spectrum of peniphilone G (**9**)

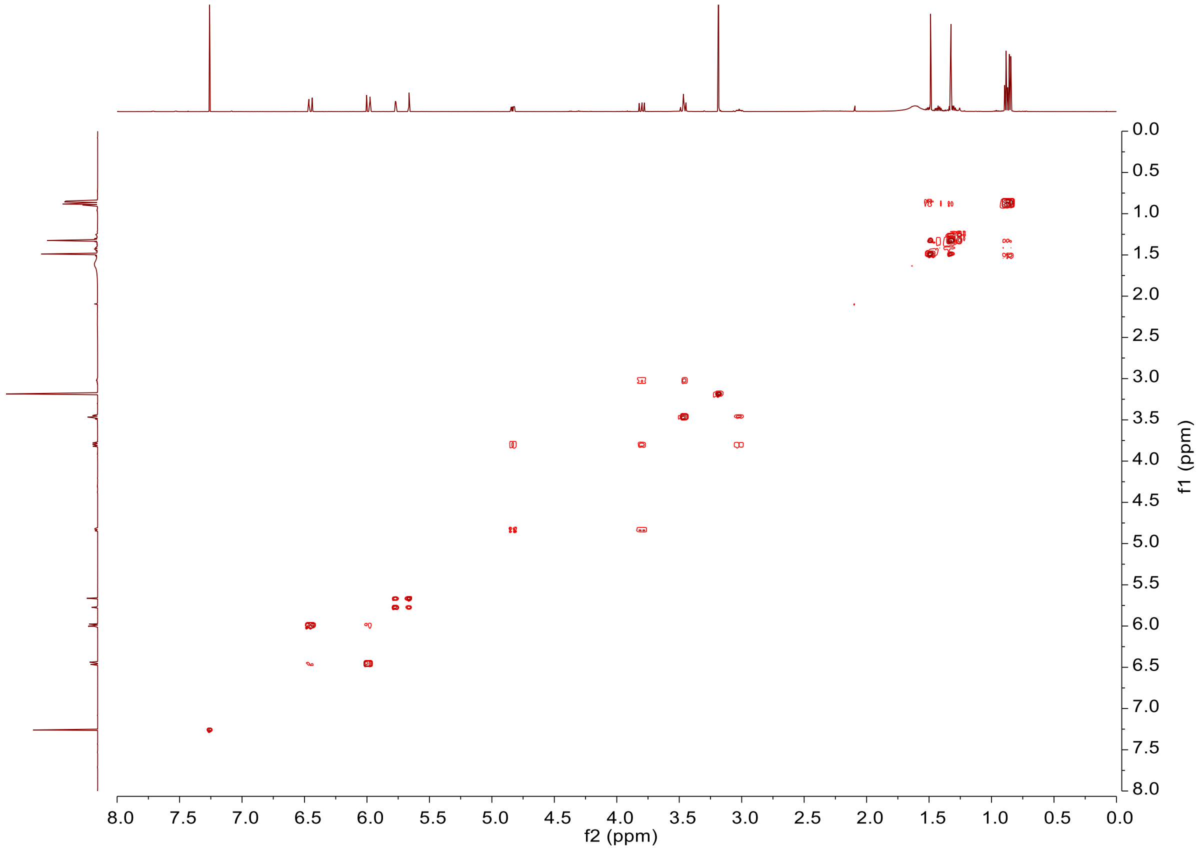

Figure S70. COSY spectrum of peniphilone G (**9**)

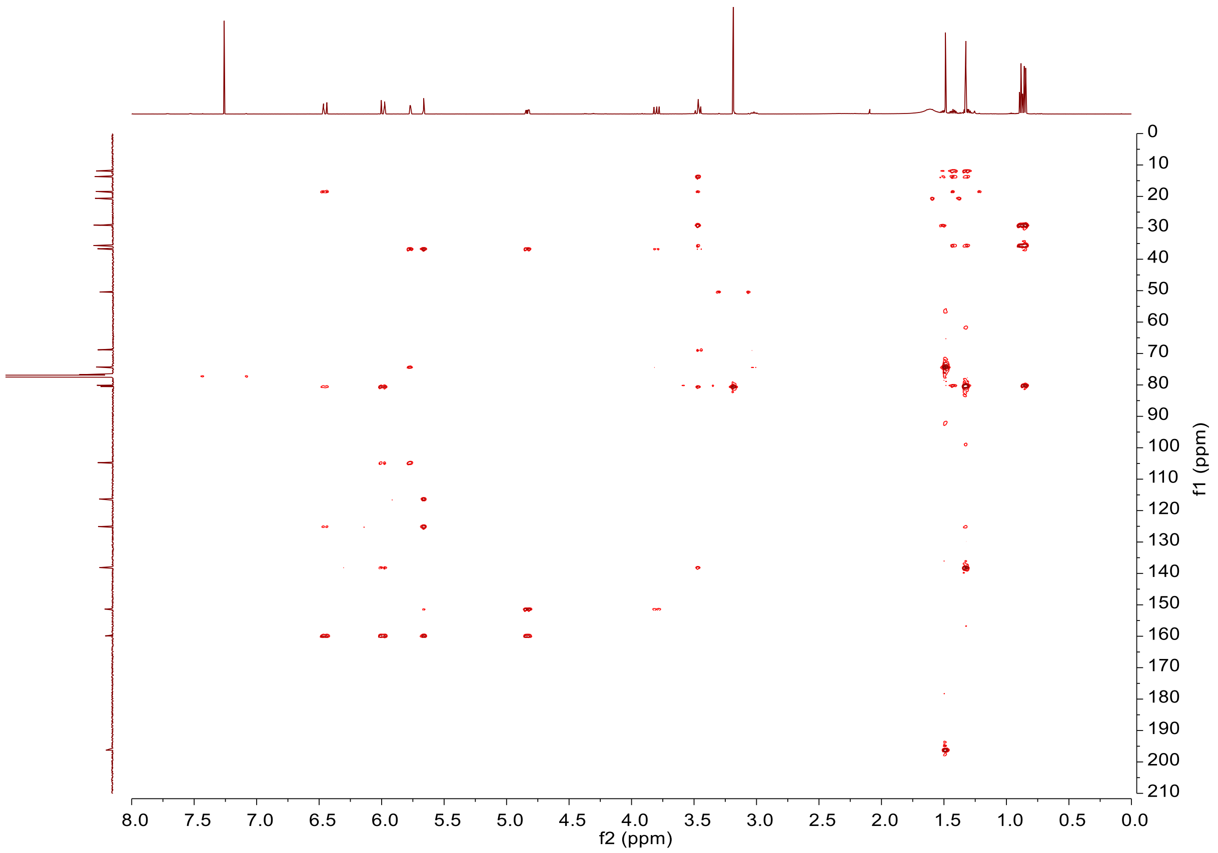

Figure S71. HMBC spectrum of peniphilone G (**9**)

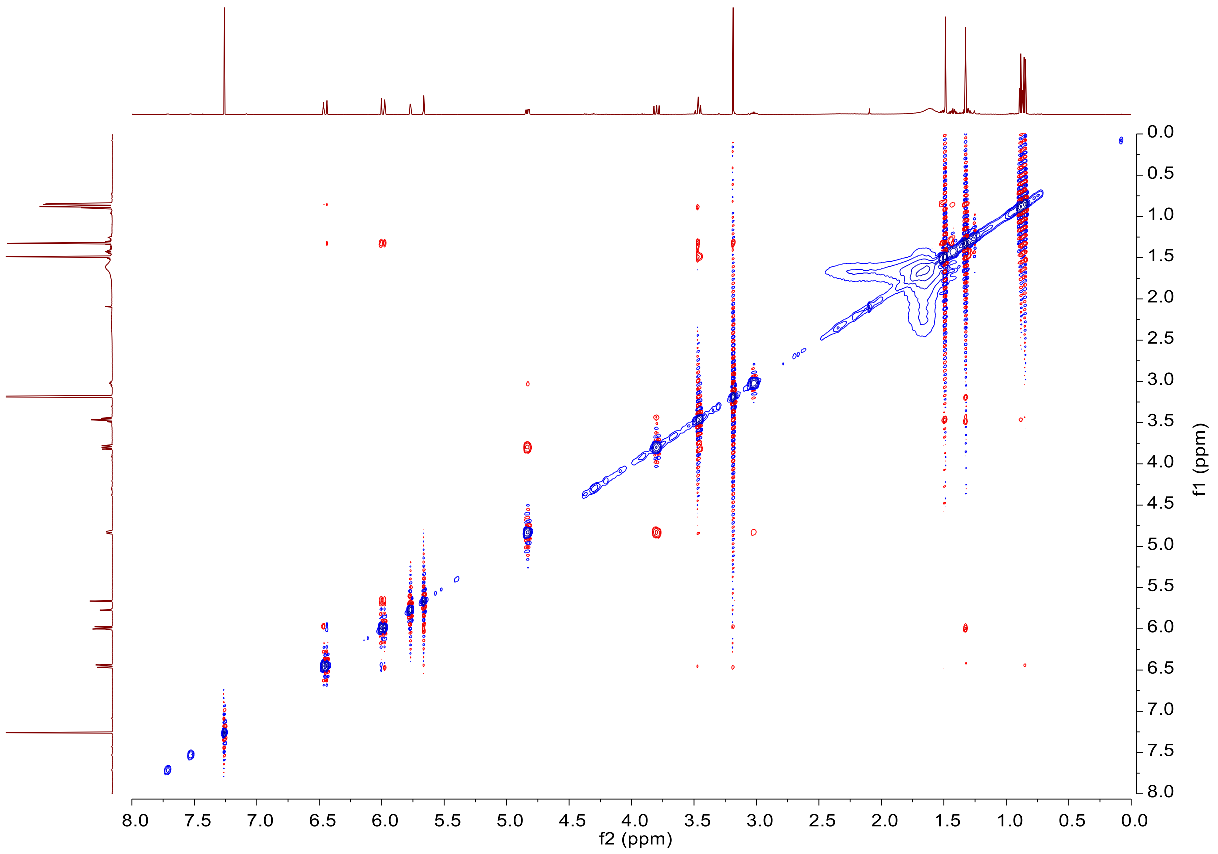

Figure S72. NOESY spectrum of peniphilone G (**9**)

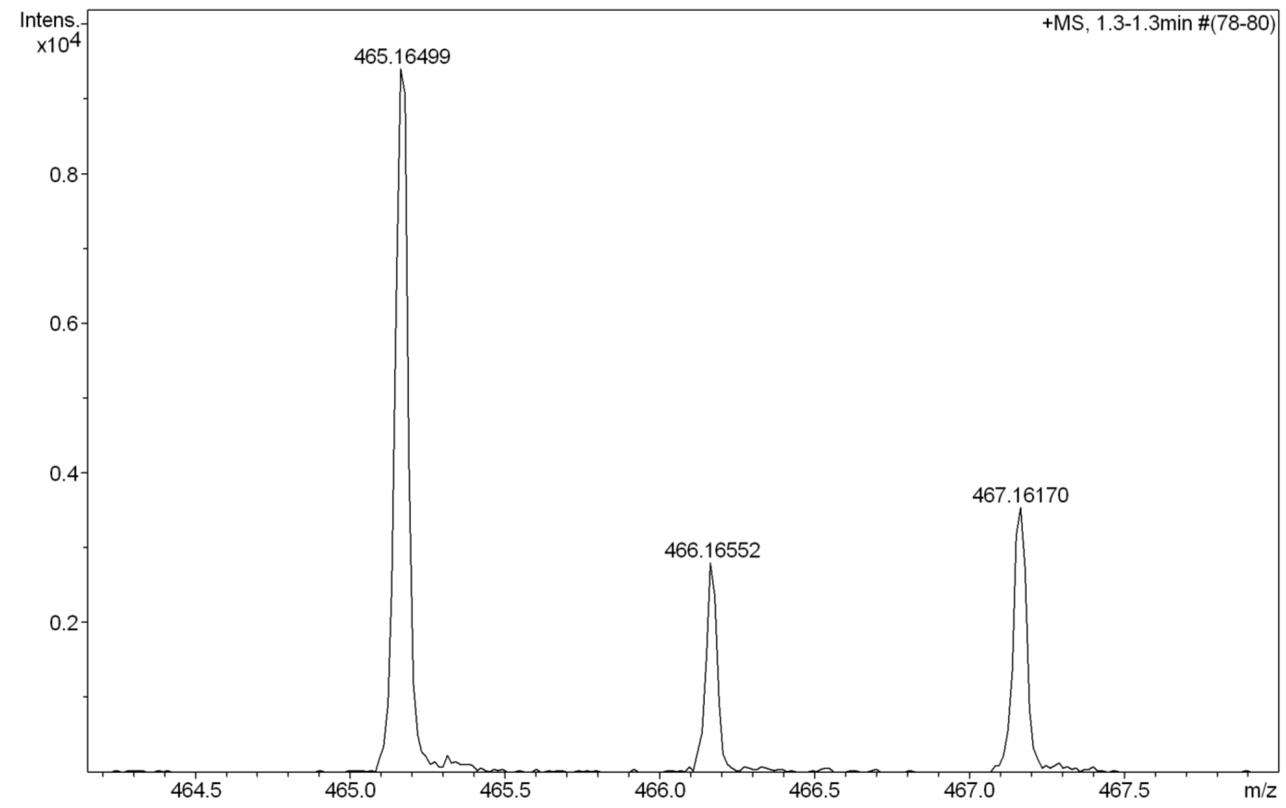

Meas. m/z	#	Formula	Score	m/z	err [mDa]	err [ppm]	mSigma	rdb	e⁻ Conf	N-Rule
465.16499	1	C 22 H 31 Cl Na O 7	100.00	465.16505	0.07	0.14	29.4	6.5	even	ok

Figure S73. HRESIMS spectrum of peniphilone H (**10**)

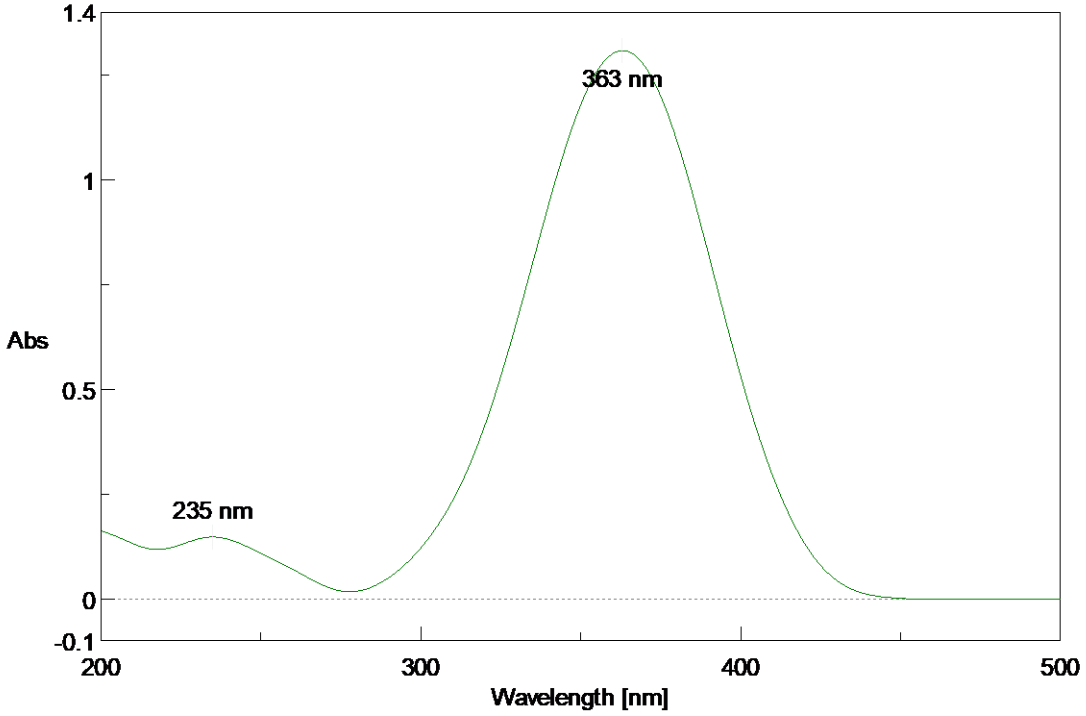

Figure S74. UV spectrum of peniphilone H (**10**)

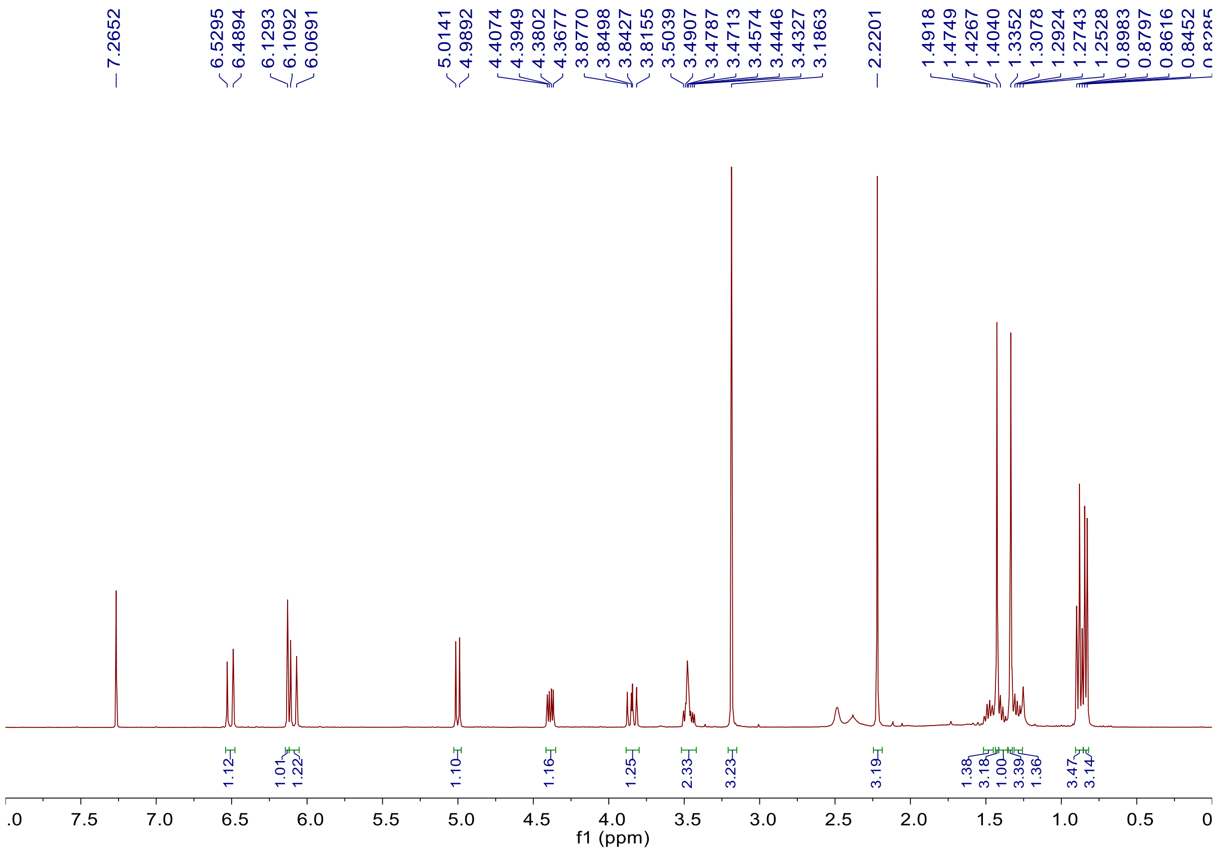

Figure S75. ^{1}H NMR spectrum (600 MHz, $CDCl_3$) of peniphilone H (**10**)

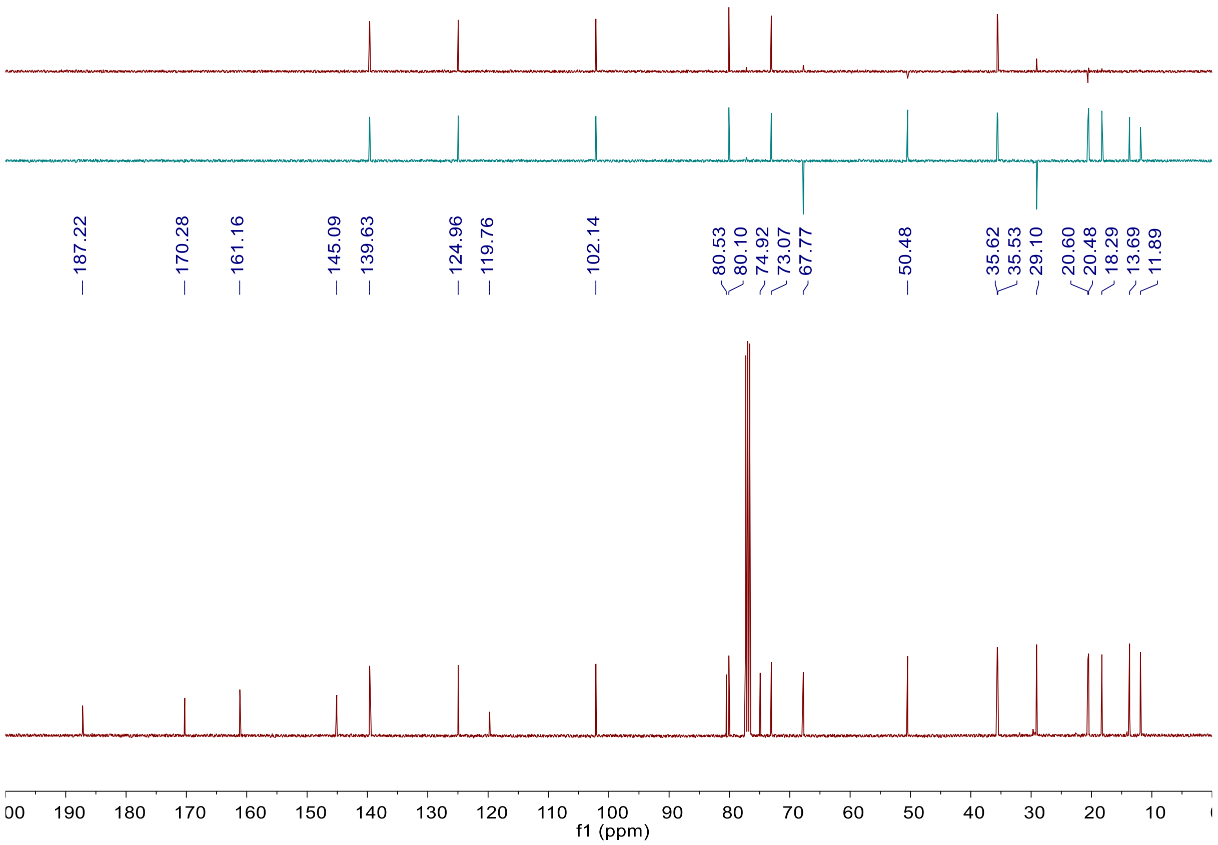

Figure S76. ^{13}C NMR spectrum (150 MHz, $CDCl_3$) of peniphilone H (**10**)

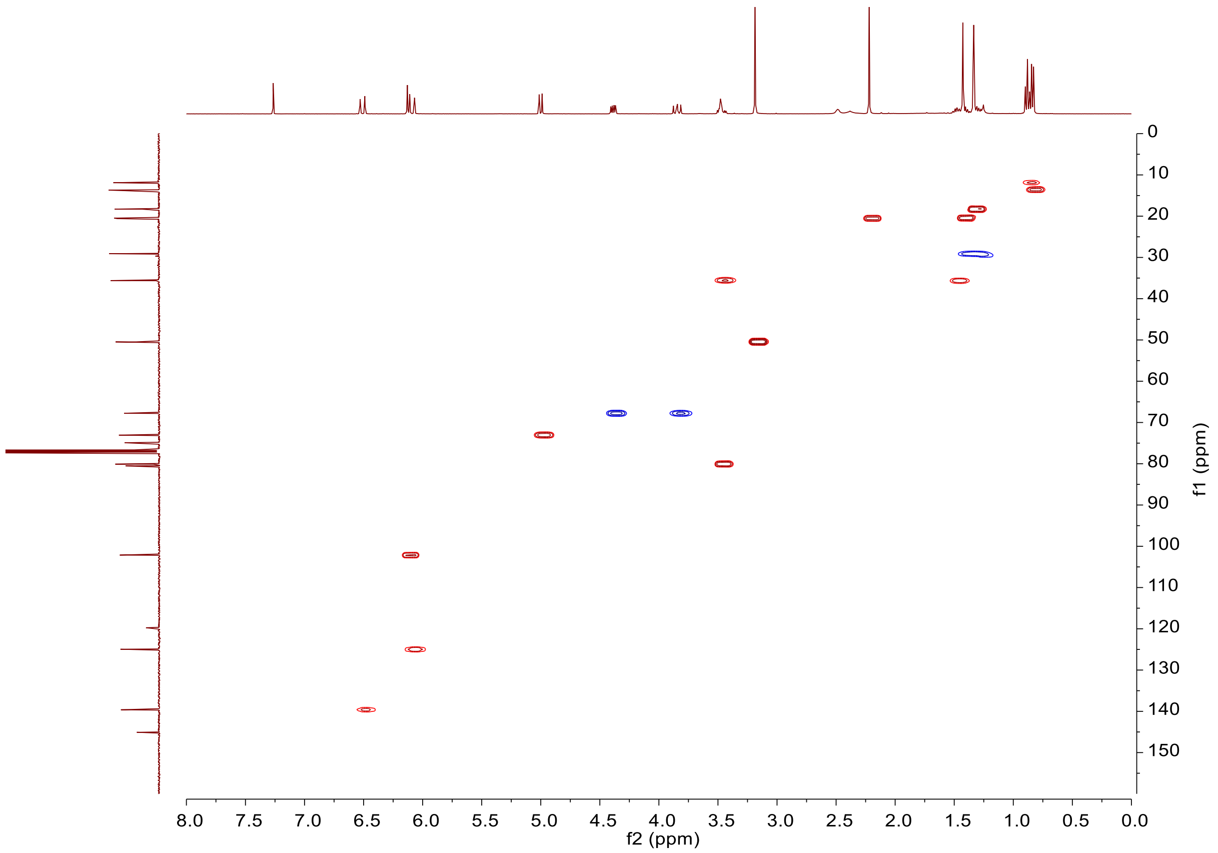

Figure S77. ^{1}H-^{13}C HSQC spectrum of peniphilone H (**10**)

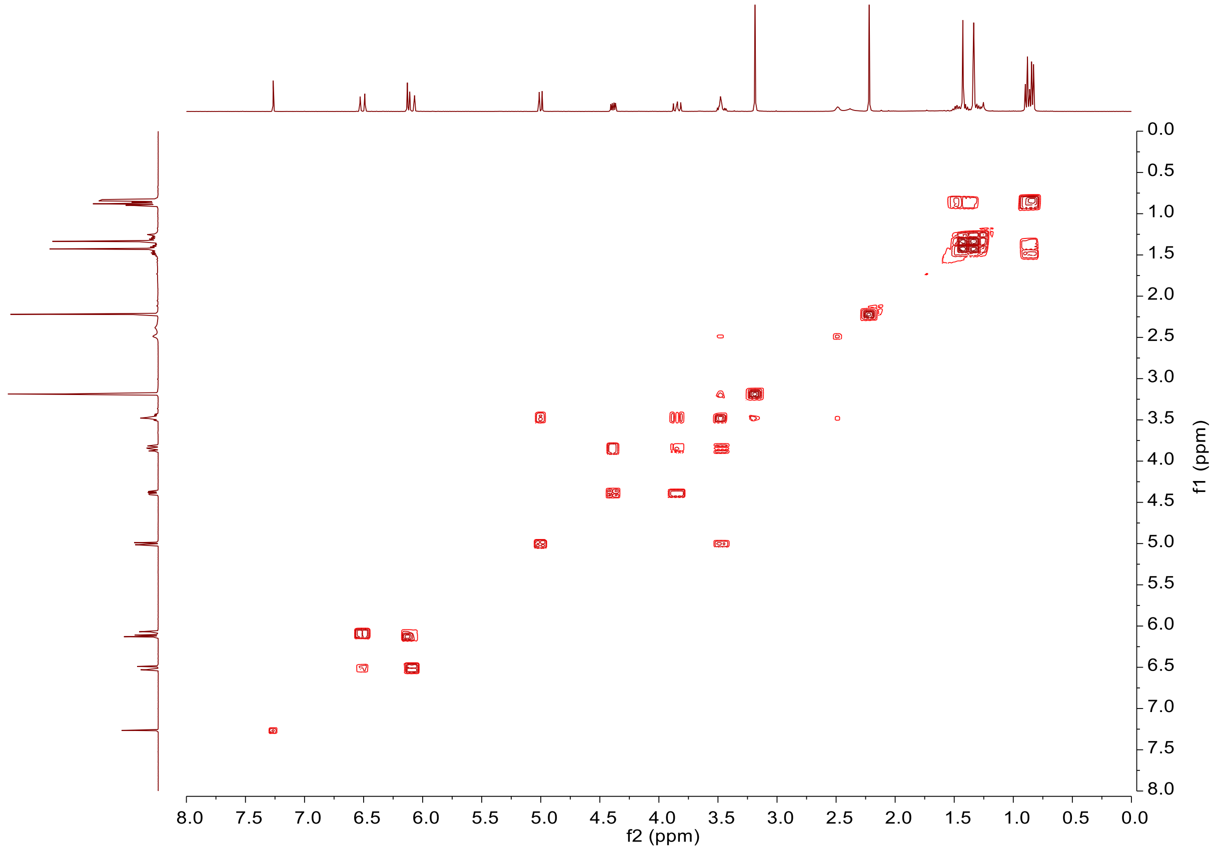

Figure S78. COSY spectrum of peniphilone H (**10**)

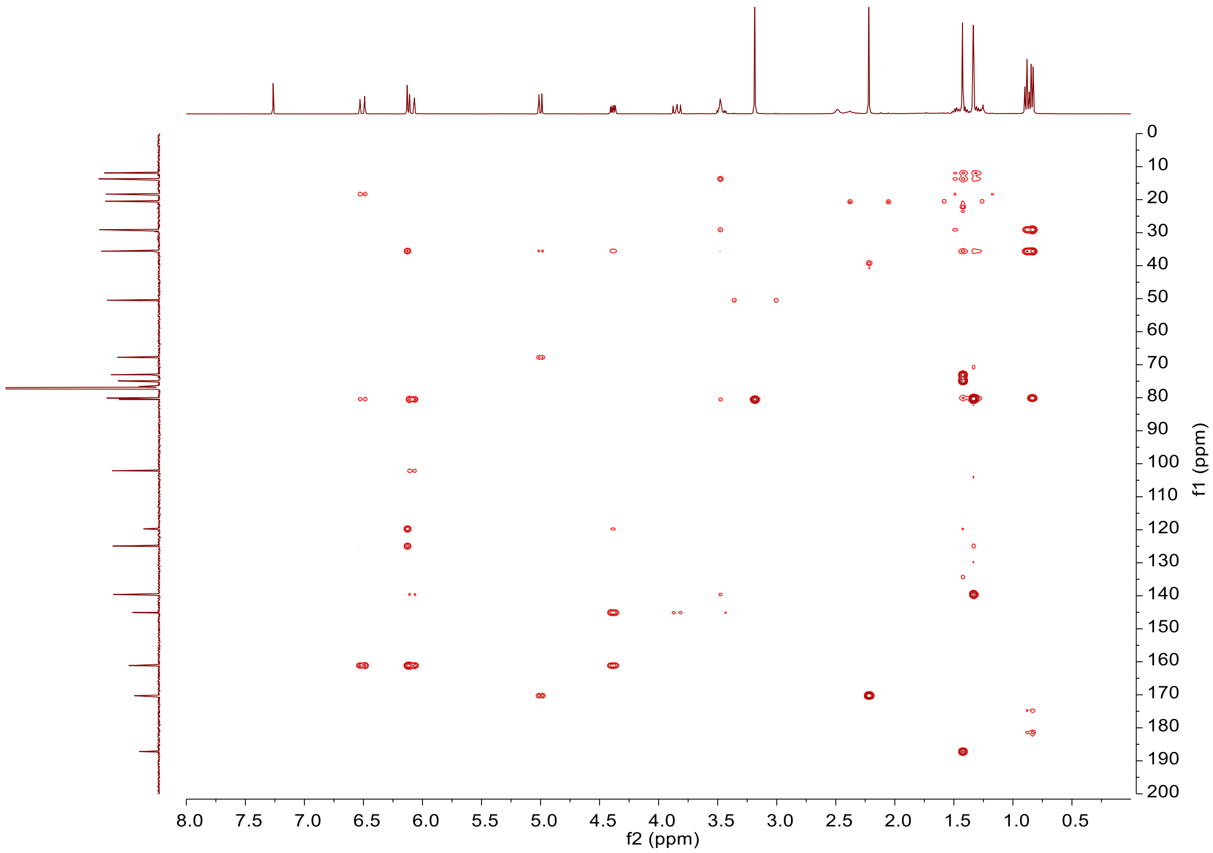

Figure S79. HMBC spectrum of peniphilone H (**10**)

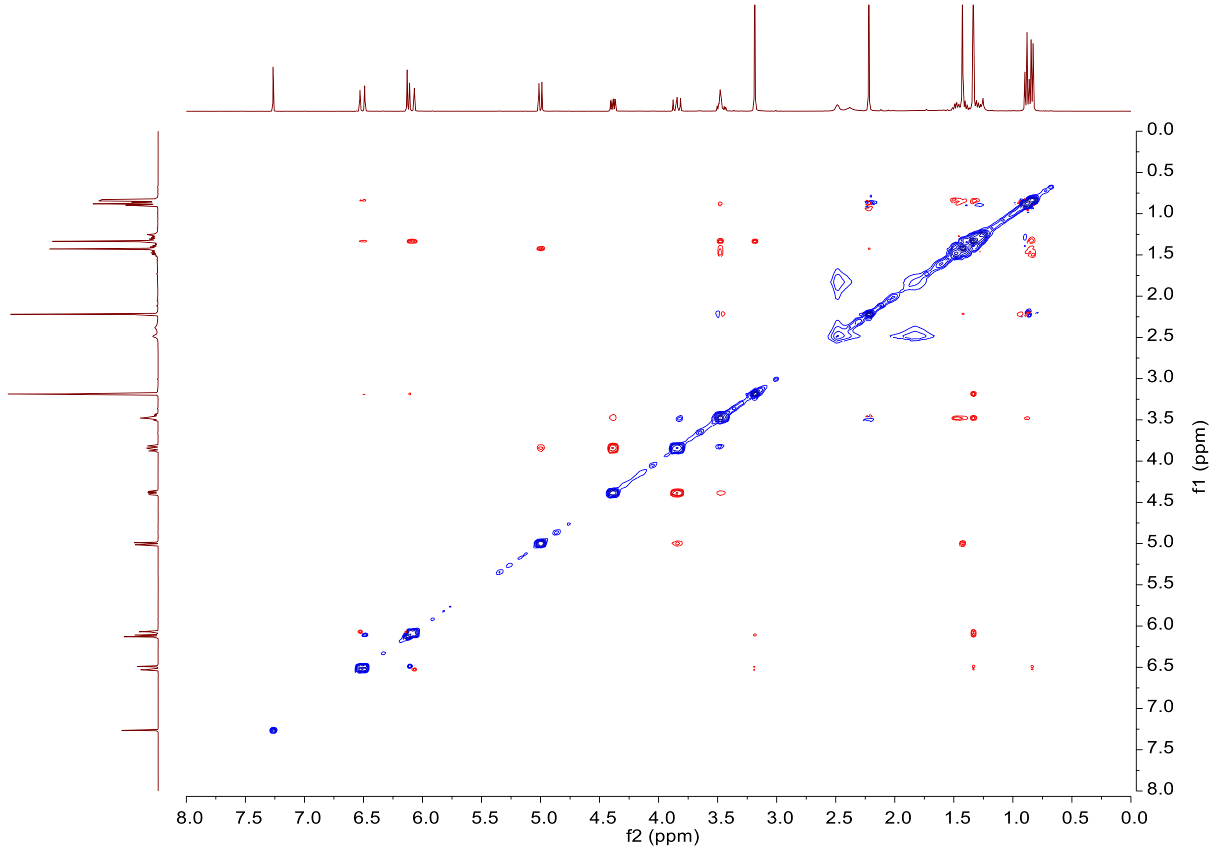

Figure S80. NOESY spectrum of peniphilone H (**10**)

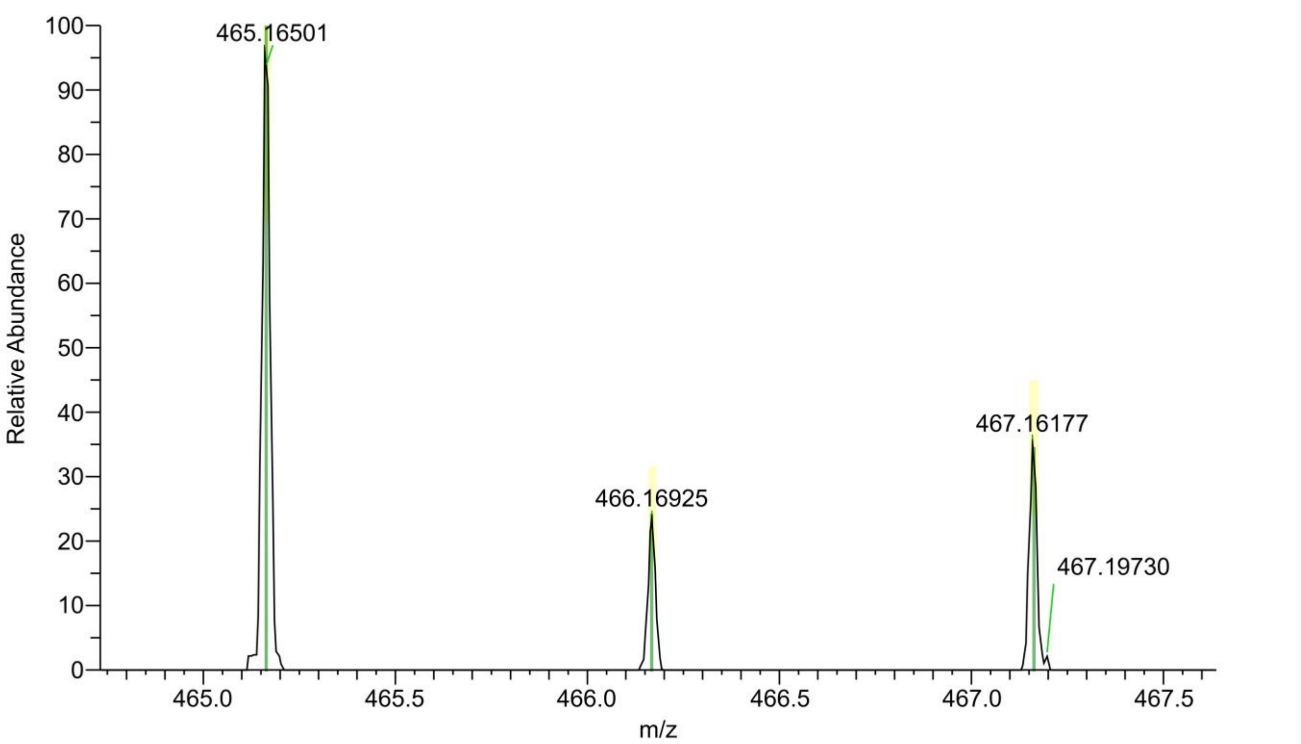

Peak Mass	Display F...	S Fit	RDB	Delta [pp...	Theo. ma...	Rank	Combine...	# Match...	# Missed...	MS Cov....	Pattern C...	MSMS M...
465.1650 1	$C_{22}H_{31}O_7$ $^{35}Cl^{23}Na$	49.75005 7753108 2	6.50	-0.09	465.1650 5	1	96.05	4	1	98.62	99.22	(Collecti on)

Figure S81. HRESIMS spectrum of peniphilone I (**11**)

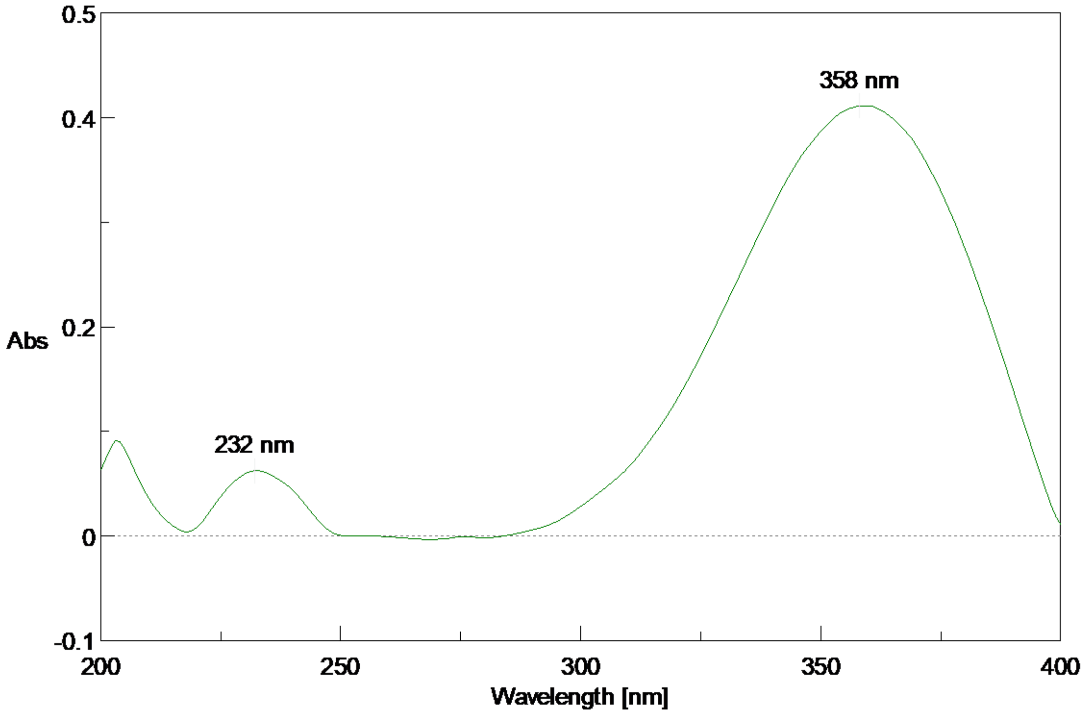

Figure S82. UV spectrum of peniphilone I (**11**)

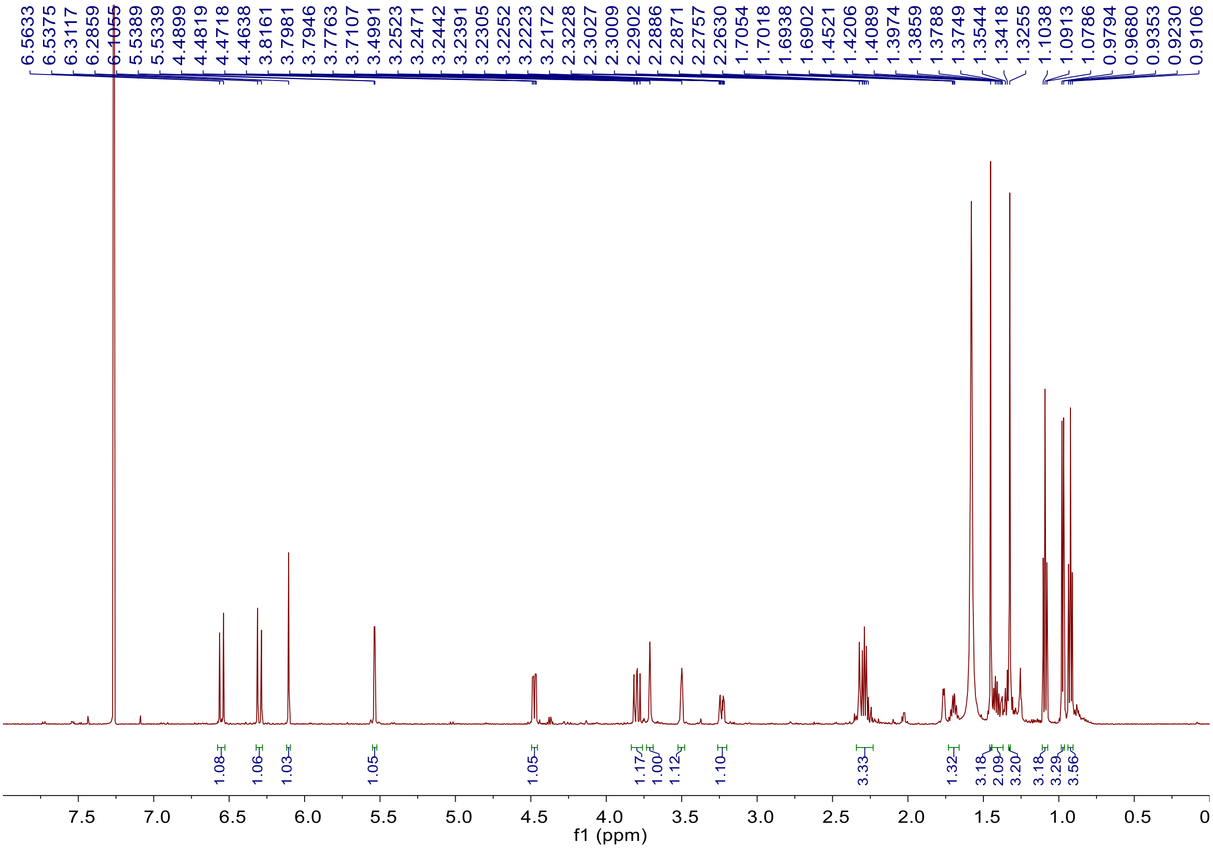

Figure S83. ^{1}H NMR spectrum (600 MHz, $CDCl_3$) of peniphilone I (**11**)

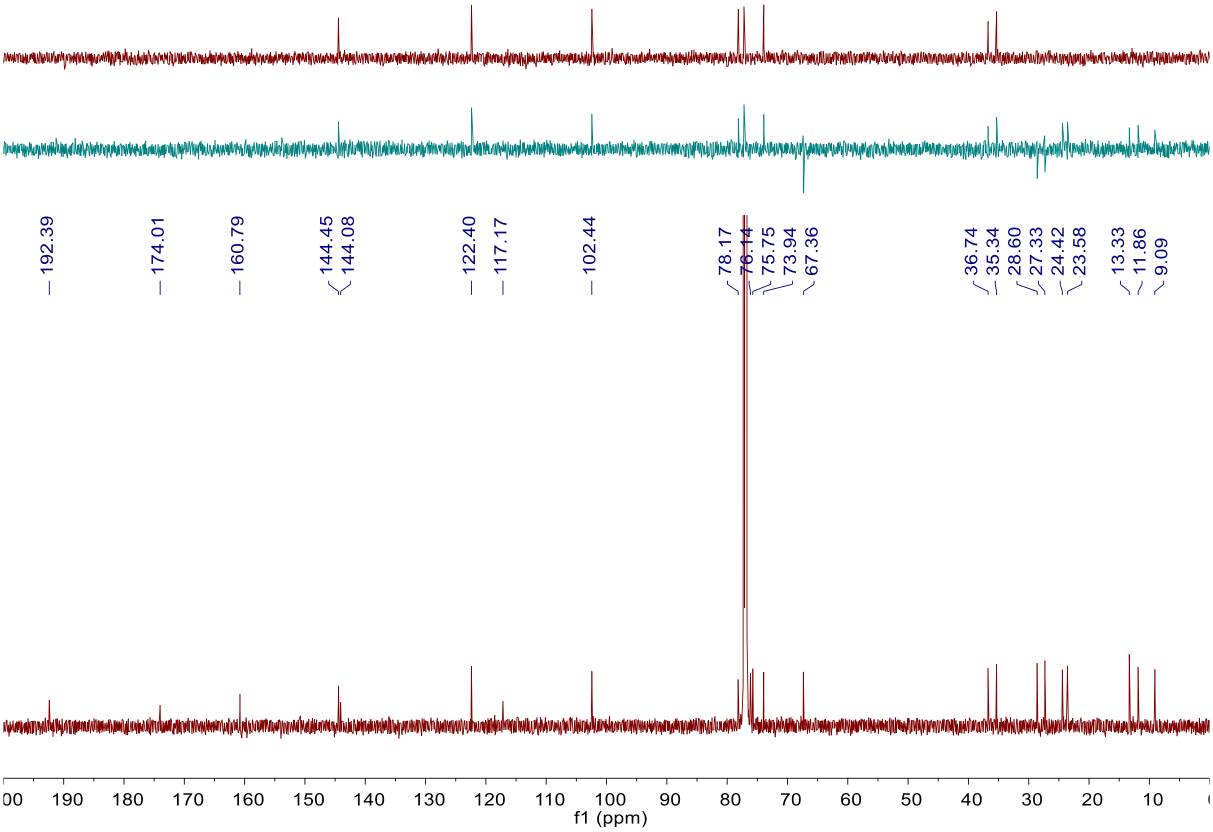

Figure S84. ^{13}C NMR spectrum (150 MHz, $CDCl_3$) of peniphilone I (**11**)

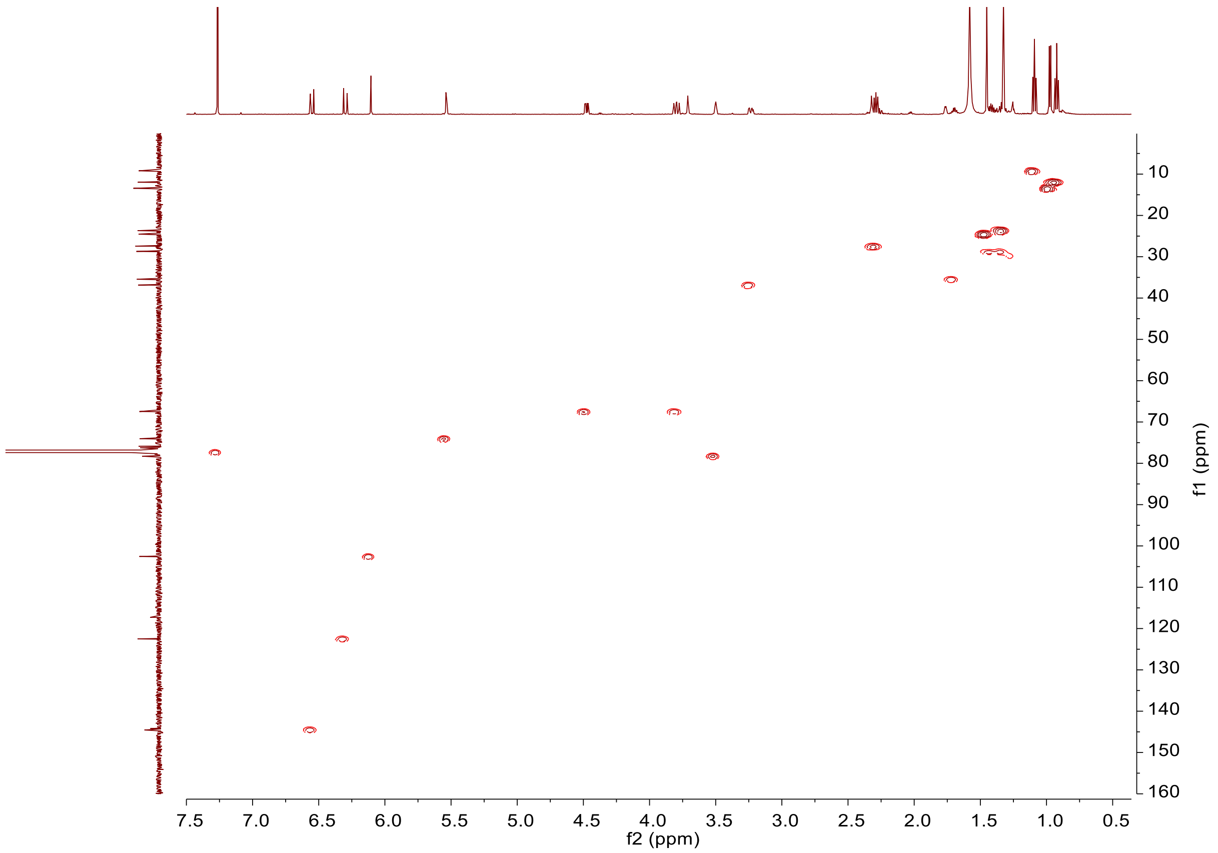

Figure S85. ^{1}H-^{13}C HSQC spectrum of peniphilone I (**11**)

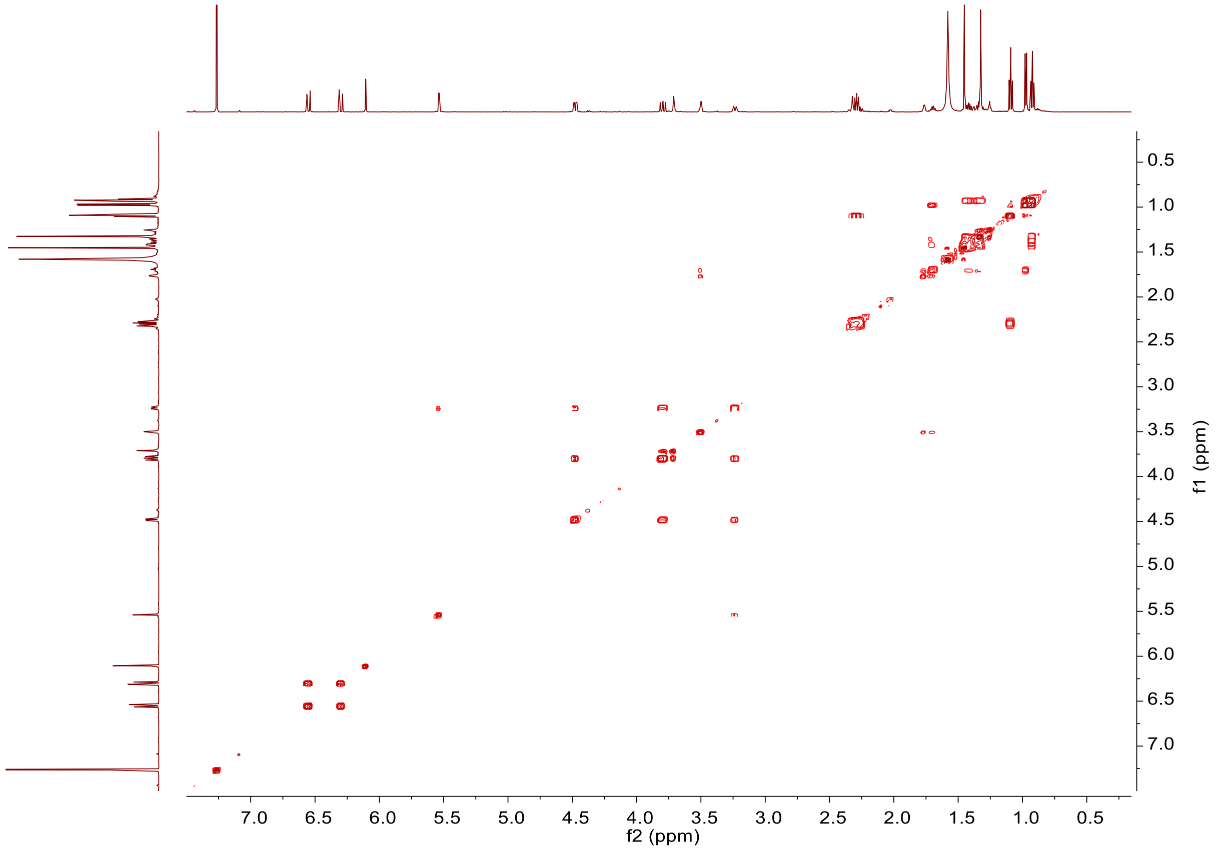

Figure S86. COSY spectrum of peniphilone I (**11**)

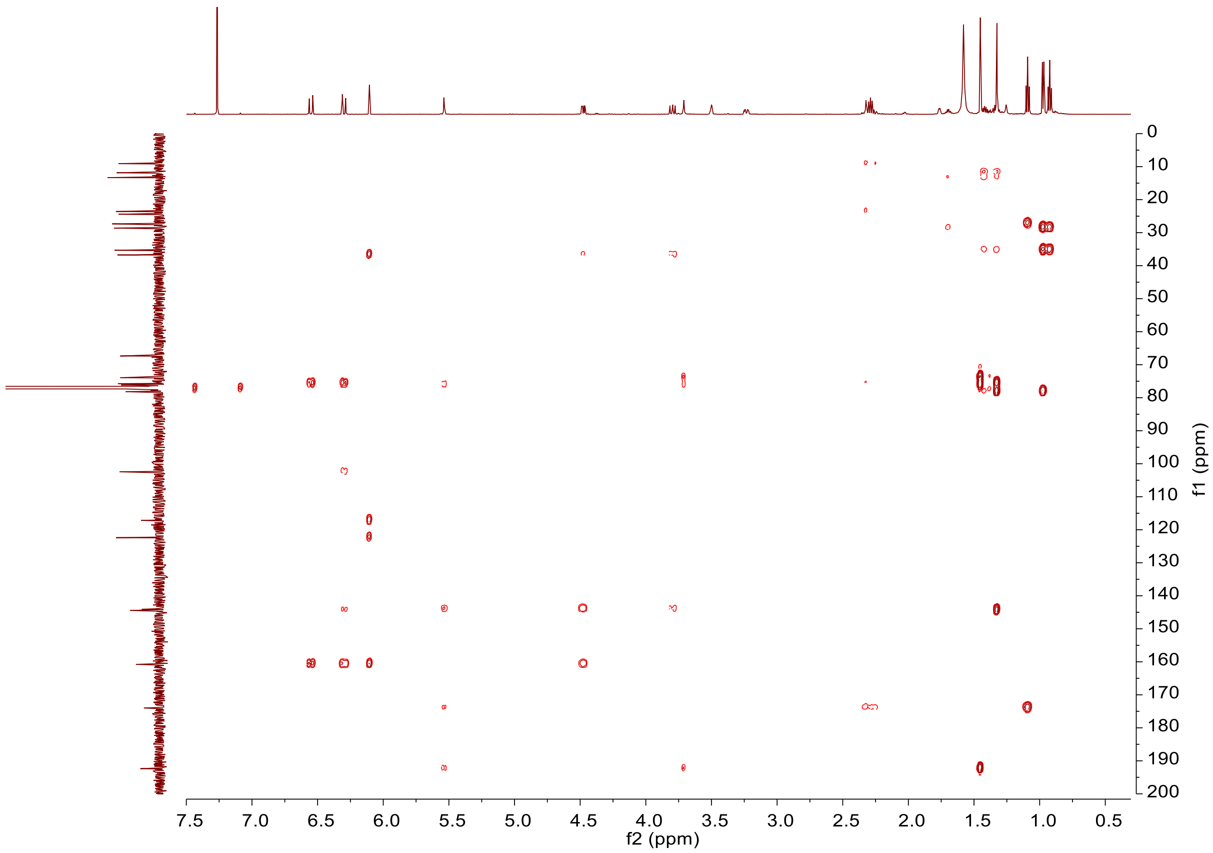

Figure S87. HMBC spectrum of peniphilone I (**11**)

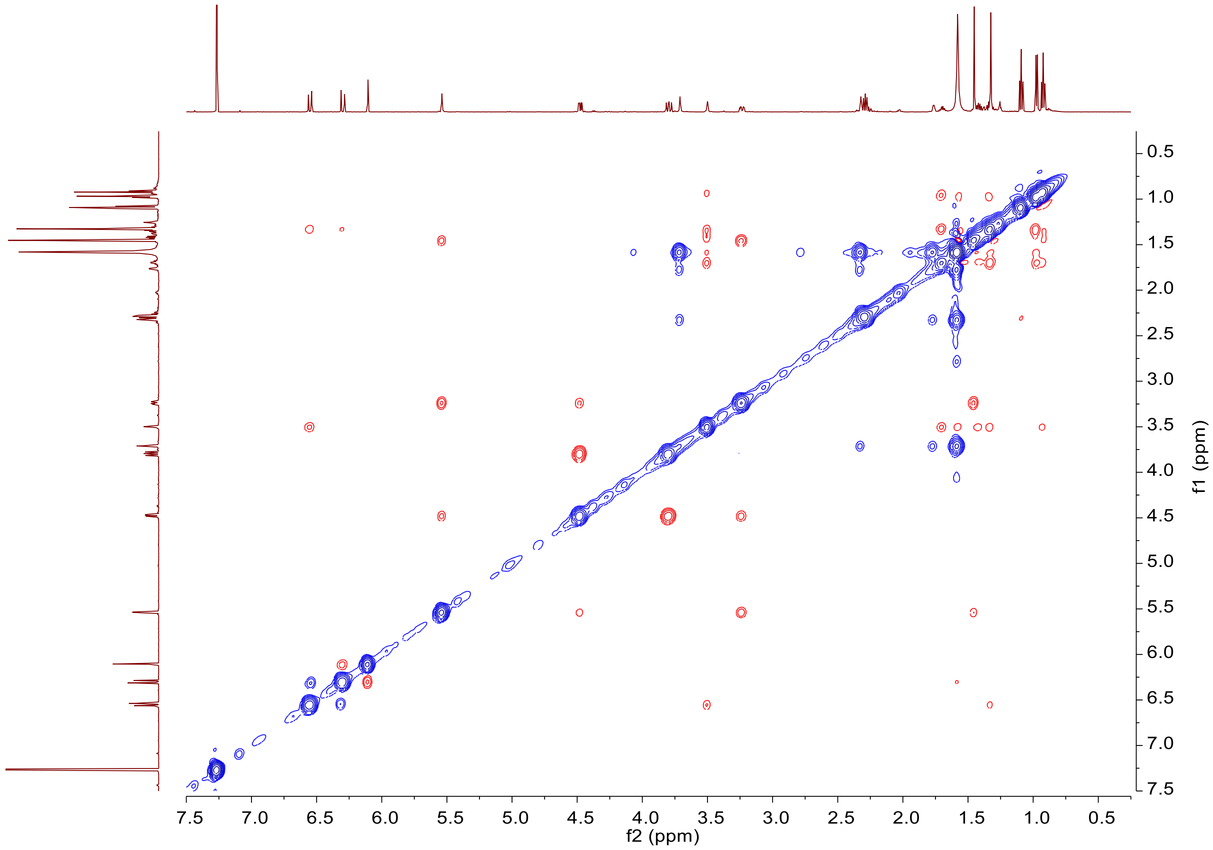

Figure S88. NOESY spectrum of peniphilone I (**11**)

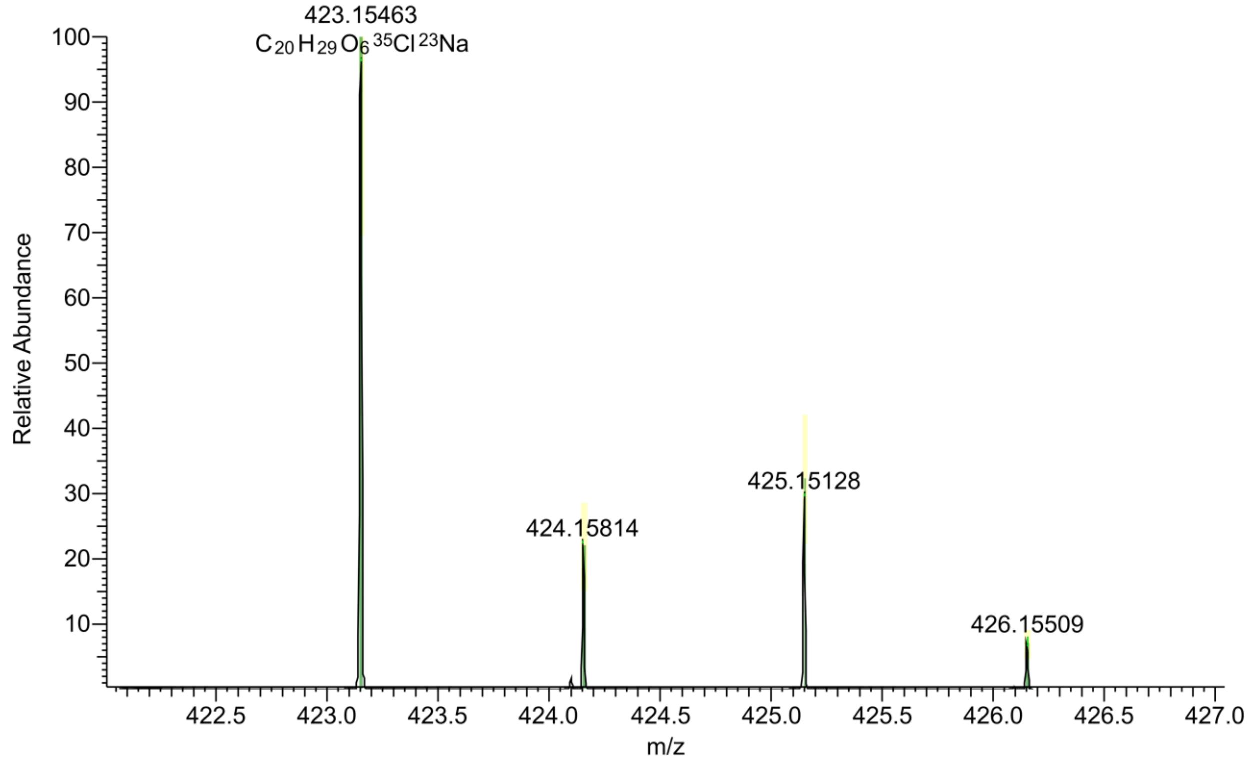

Peak Mass	Display Formula	S Fit	RDB	Delta [ppm]	Theo. mass	Pattern Cov. [%]	MSMS Matched...
423.15463	$C_{20}H_{29}O_6{}^{35}Cl^{23}Na$	54.4444868421464	5.50	0.34	423.15449	99.92	(Collection)

Figure S89. HRESIMS spectrum of peniphilone J (**12**)

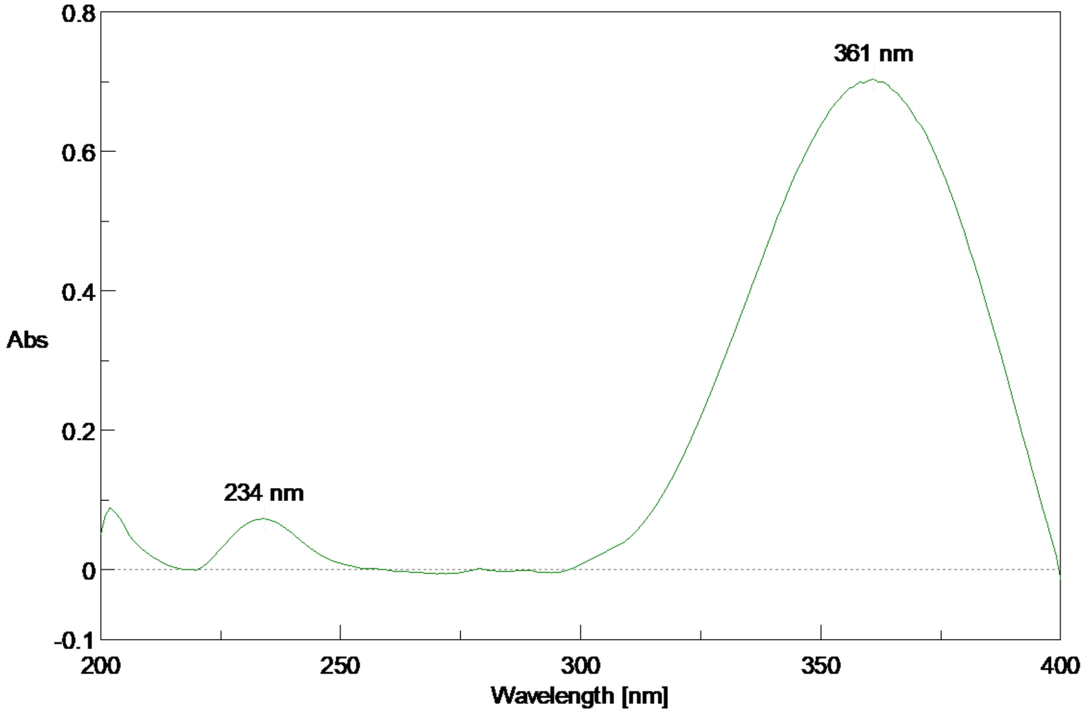

Figure S90. UV spectrum of peniphilone J (**12**)

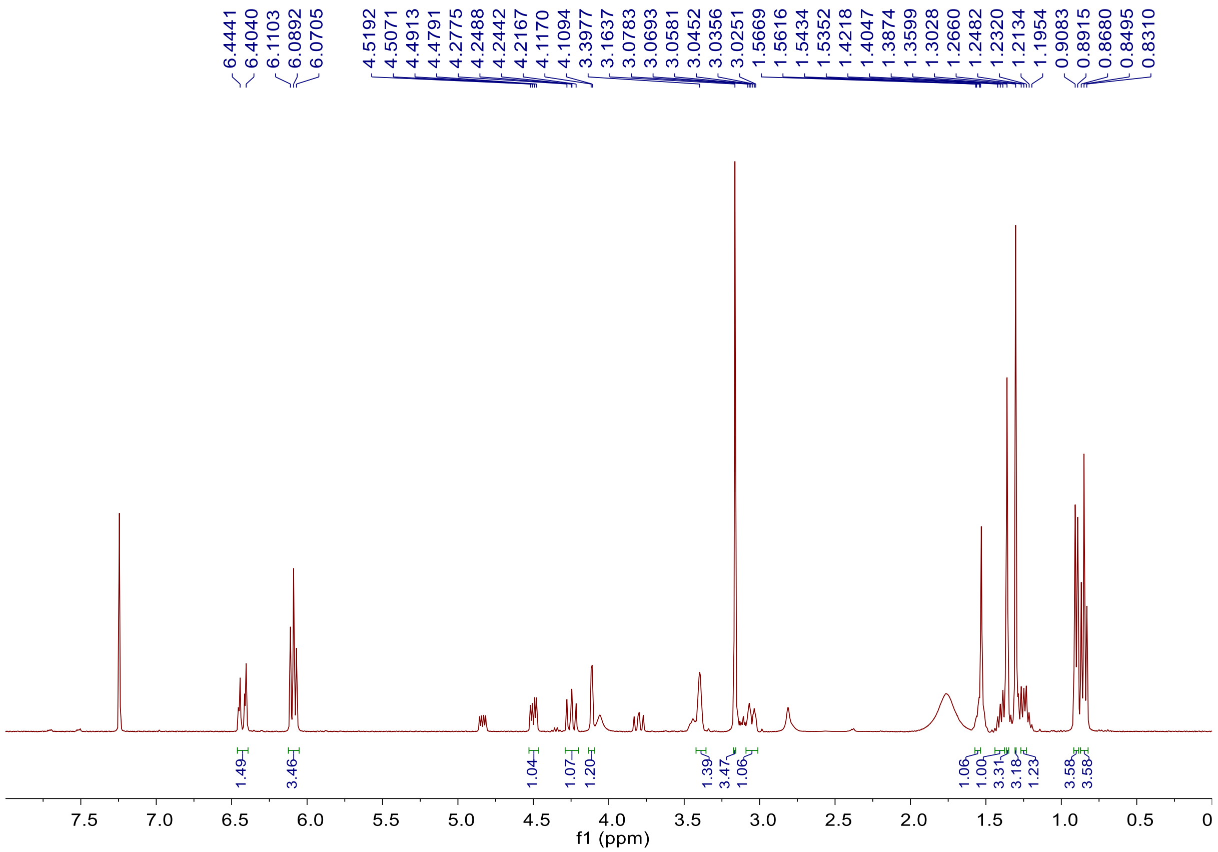

Figure S91. ^{1}H NMR spectrum (600 MHz, $CDCl_3$) of peniphilone J (**12**)

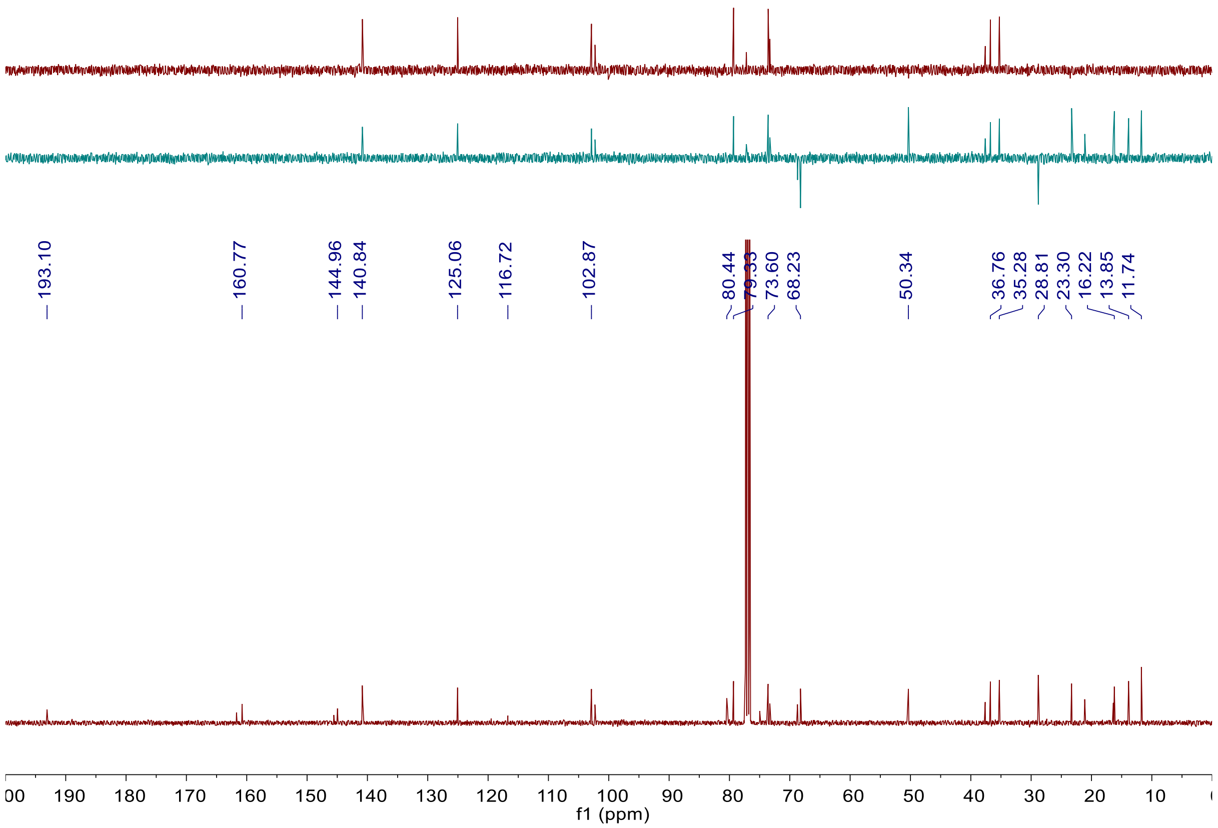

Figure S92. ^{13}C NMR spectrum (150 MHz, $CDCl_3$) of peniphilone J (**12**)

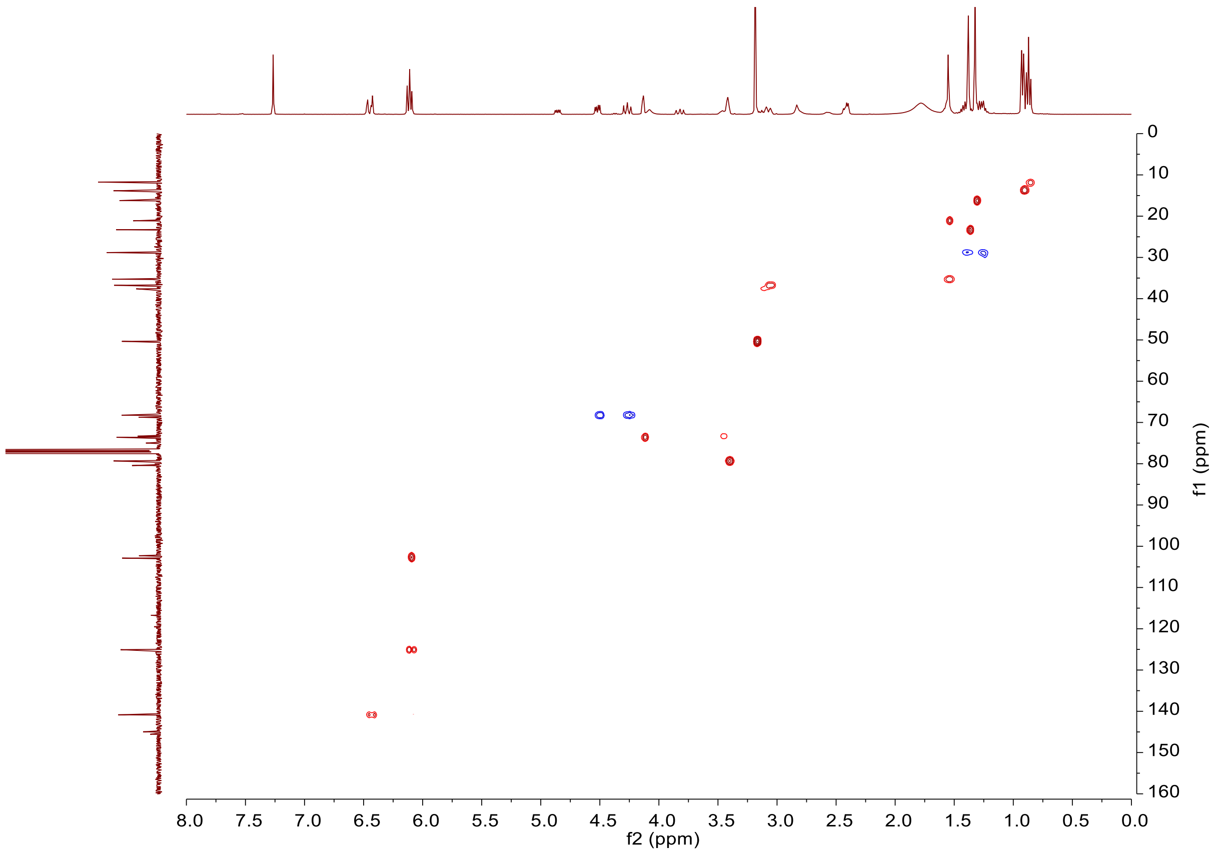

Figure S93. ^{1}H-^{13}C HSQC spectrum of peniphilone J (**12**)

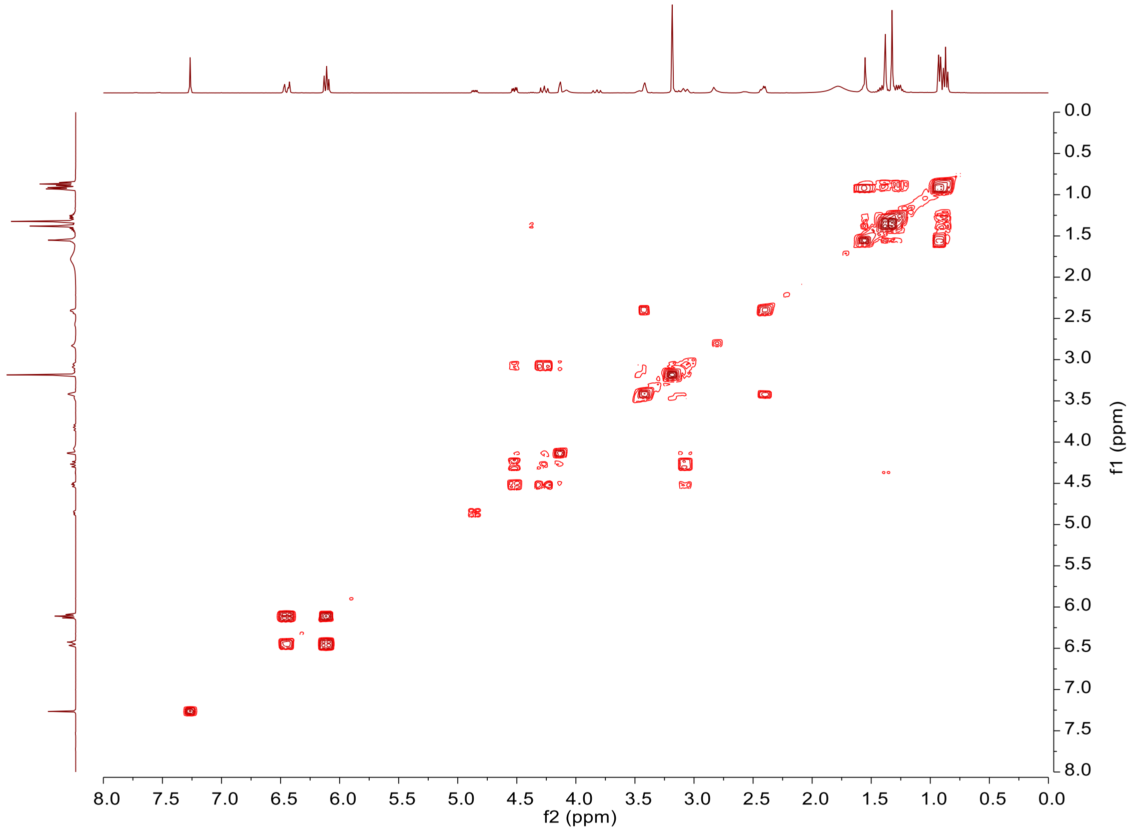

Figure S94. COSY spectrum of peniphilone J (**12**)

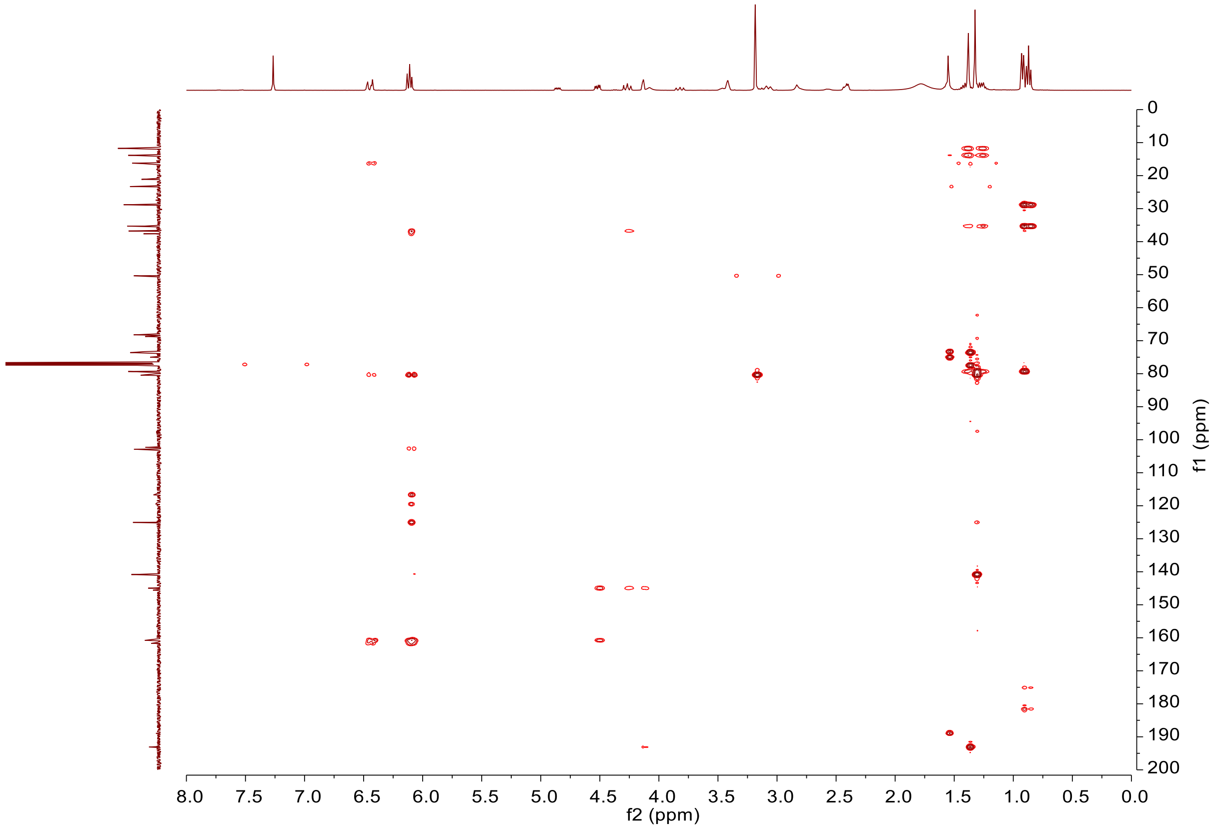

Figure S95. HMBC spectrum of peniphilone J (**12**)

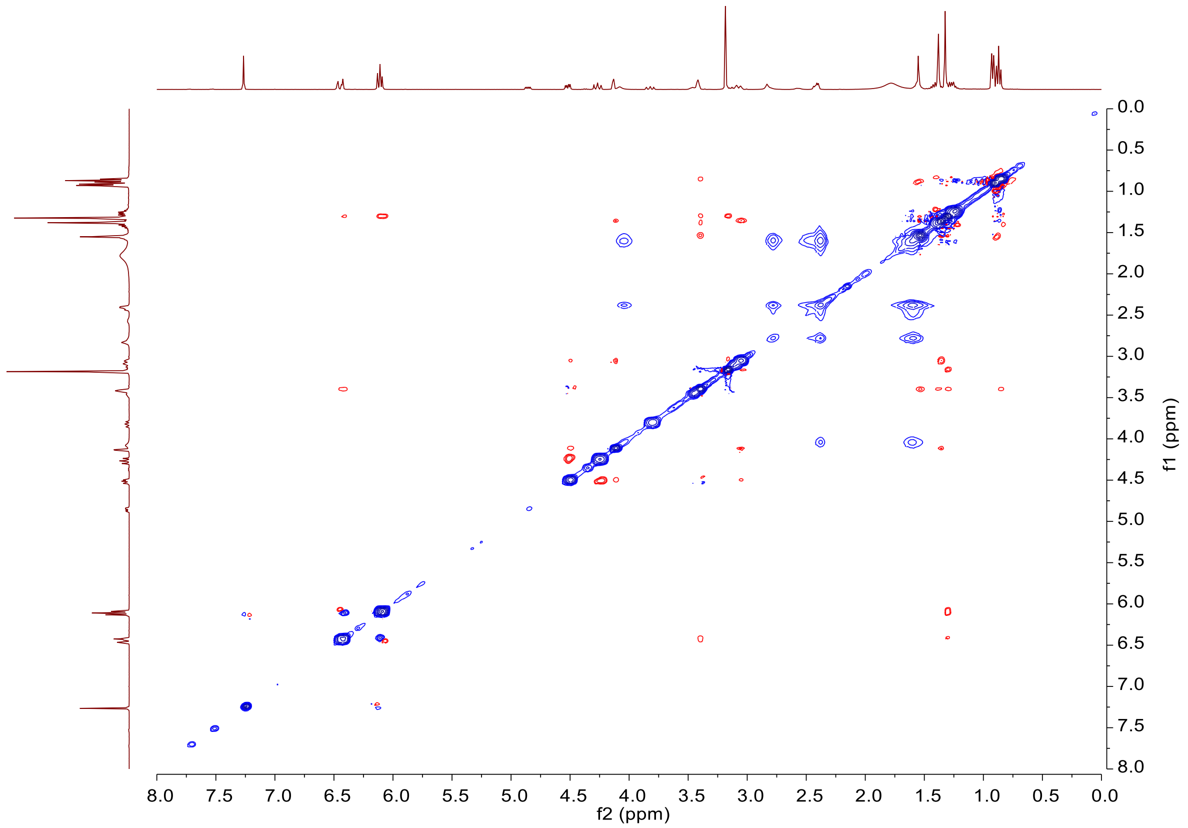

Figure S96. NOESY spectrum of peniphilone J (**12**)

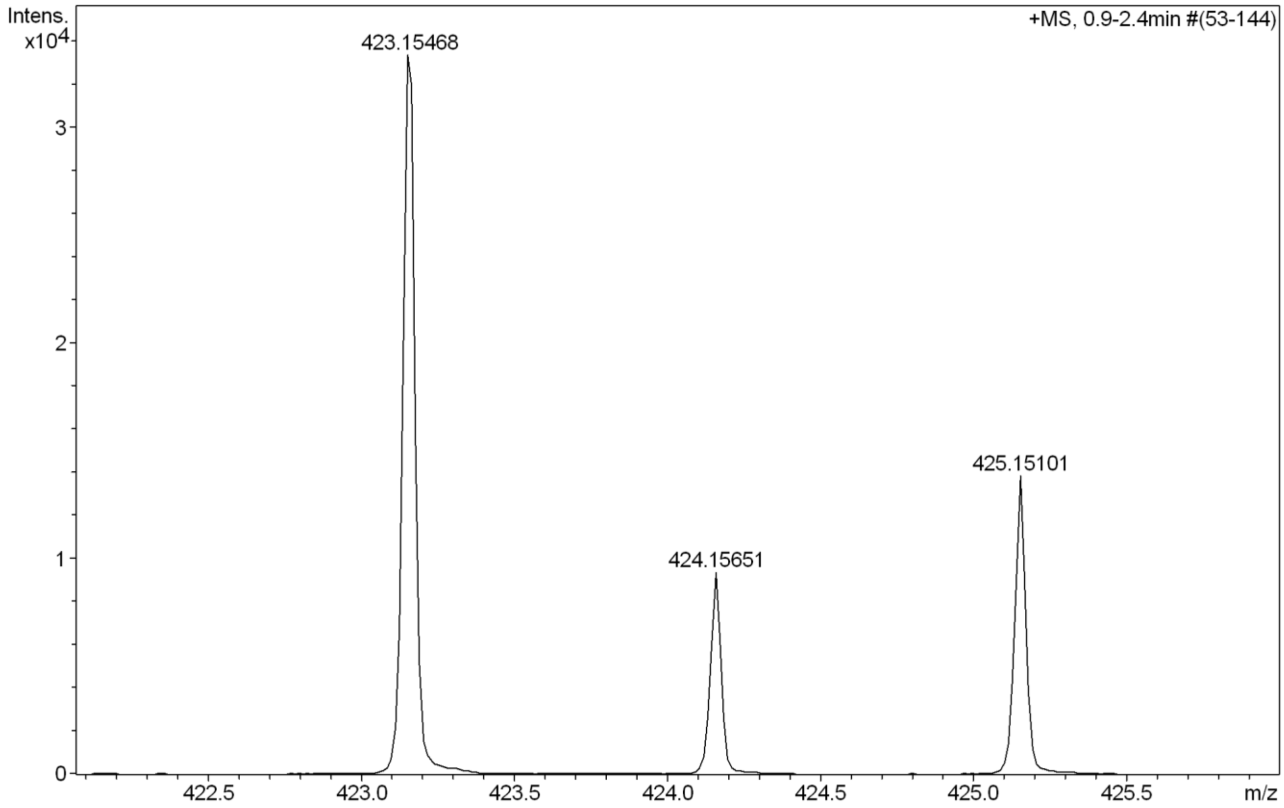

Meas. m/z	#	Formula	Score	m/z	err [mDa]	err [ppm]	mSigma	rdb	e¯ Conf	N-Rule
423.15468	1	C 20 H 29 Cl Na O 6	100.00	423.15449	-0.19	-0.45	40.1	5.5	even	ok

Figure S97. HRESIMS spectrum of peniphilone K (**13**)

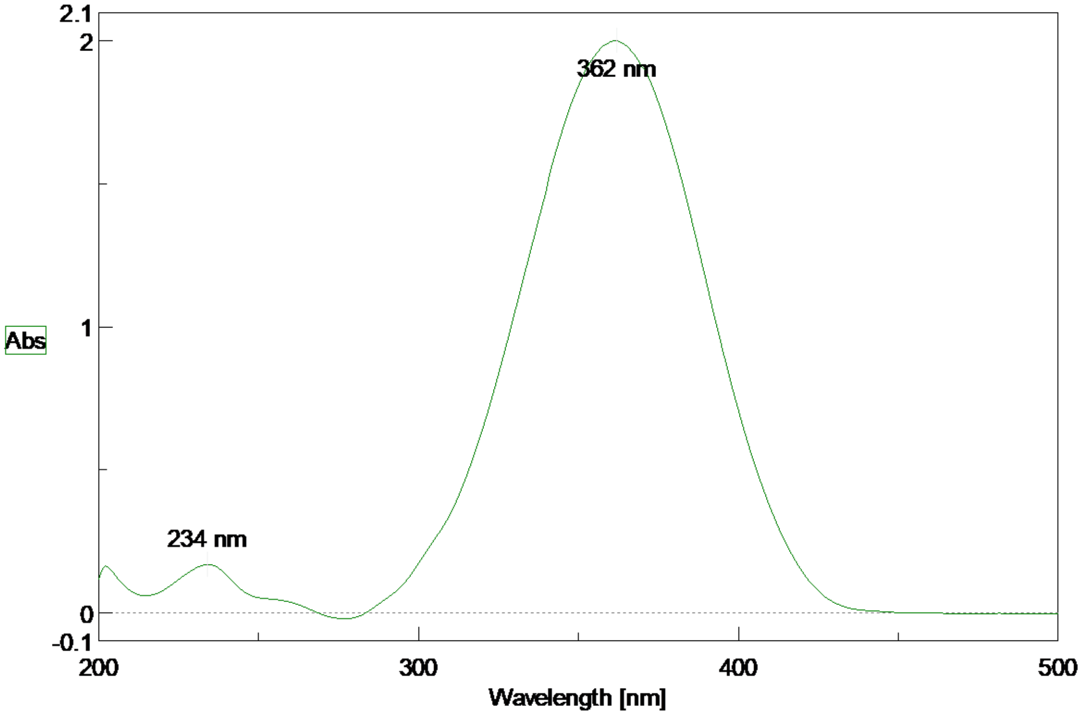

Figure S98. UV spectrum of peniphilone K (**13**)

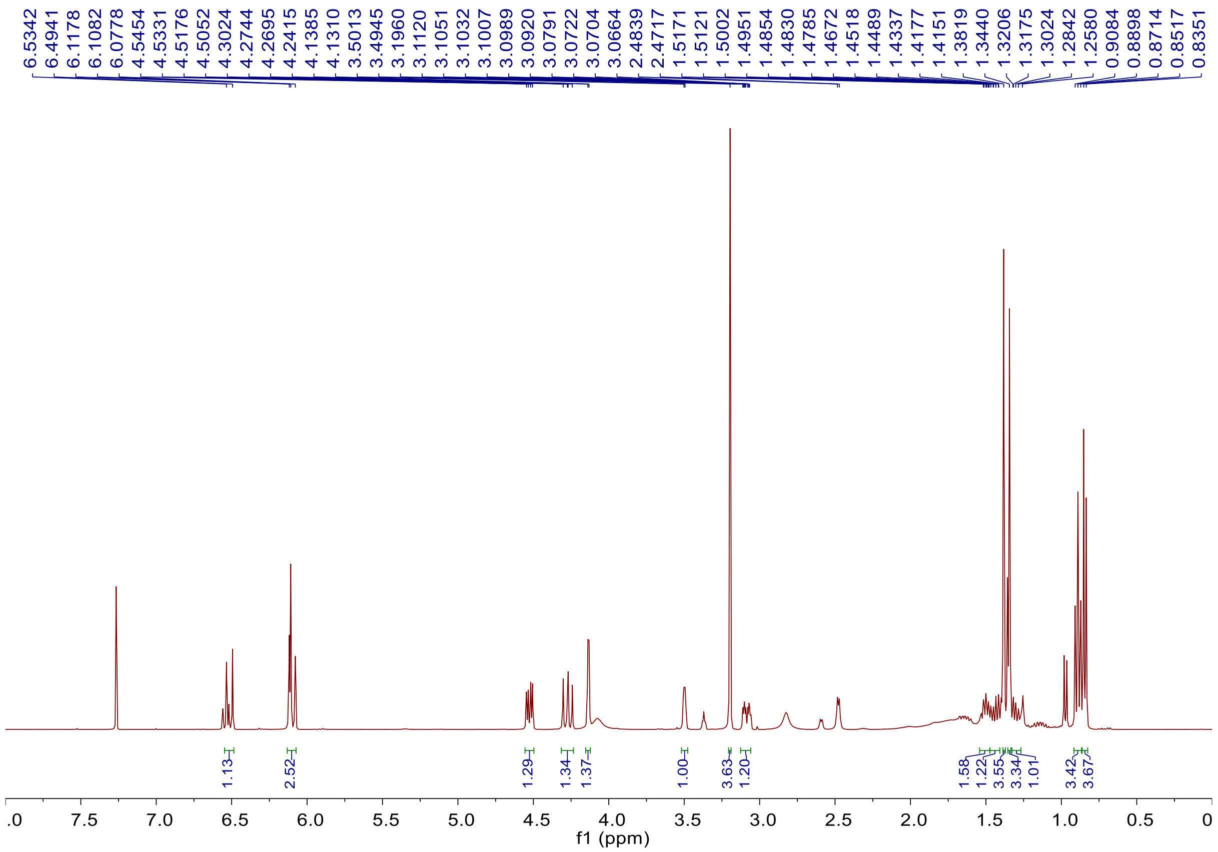

Figure S99. ^{1}H NMR spectrum (600 MHz, $CDCl_3$) of peniphilone K (**13**)

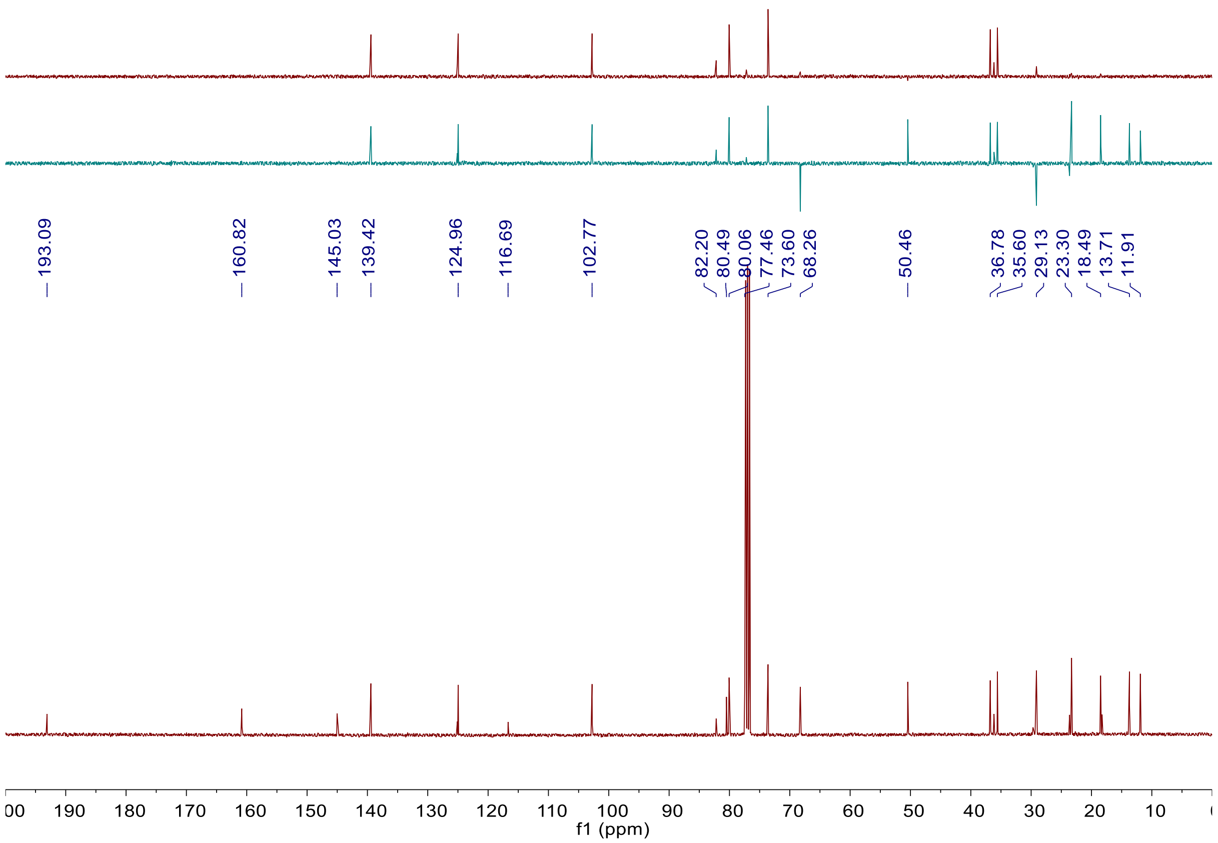

Figure S100. ^{13}C NMR spectrum (150 MHz, $CDCl_3$) of peniphilone K (**13**)

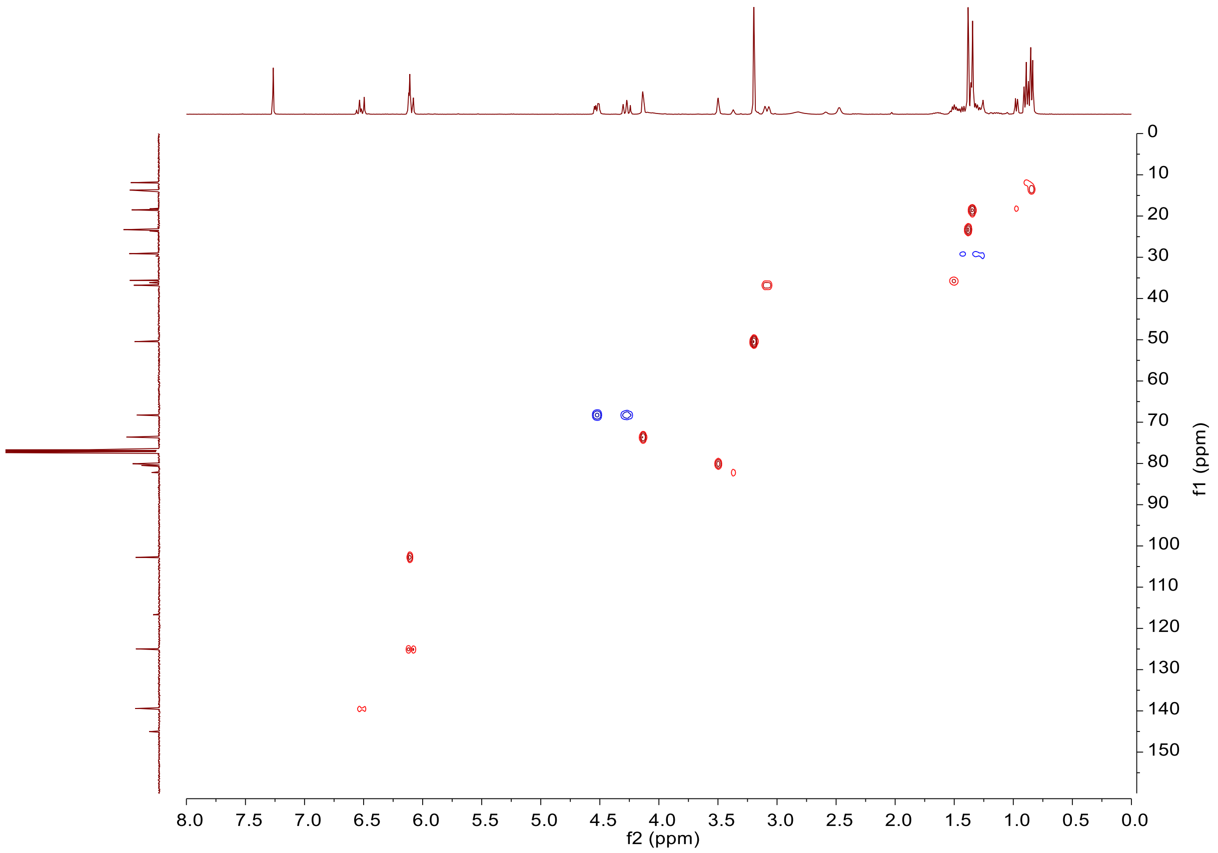

Figure S101. ^{1}H-^{13}C HSQC spectrum of peniphilone K (**13**)

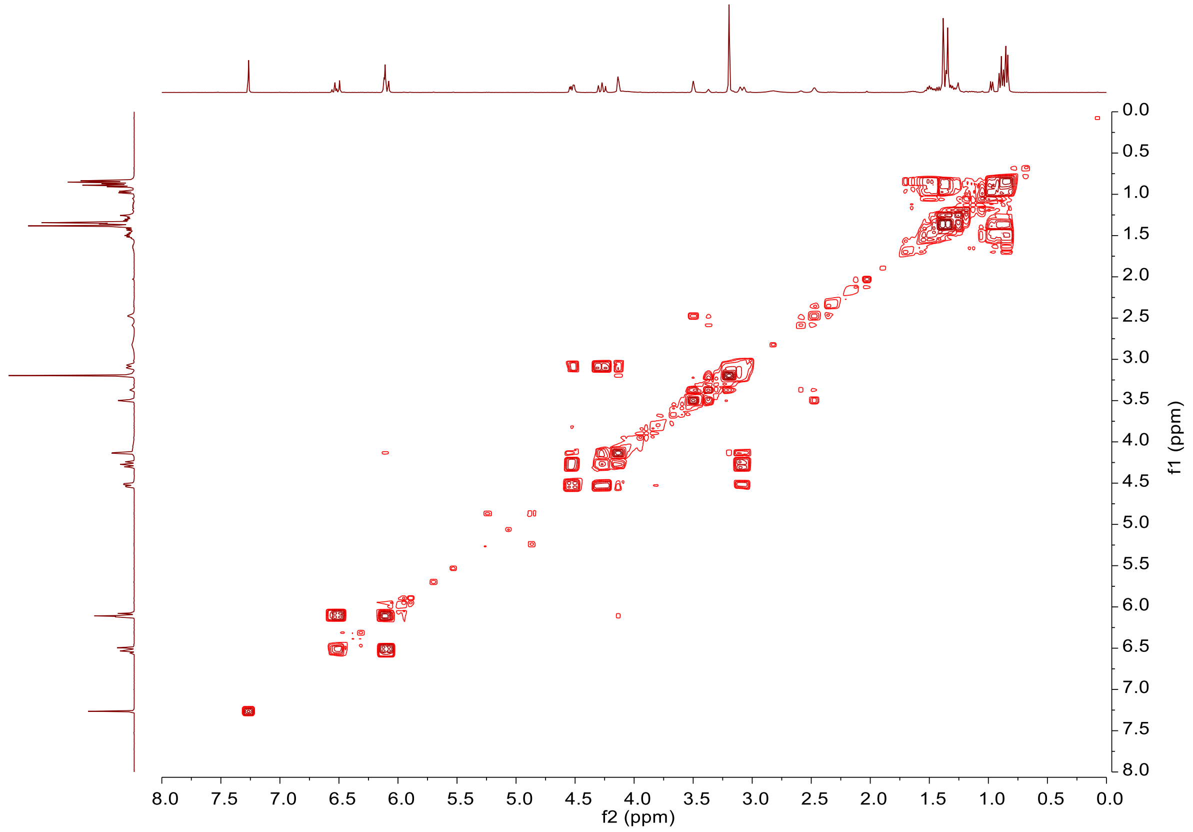

Figure S102. COSY spectrum of peniphilone K (**13**)

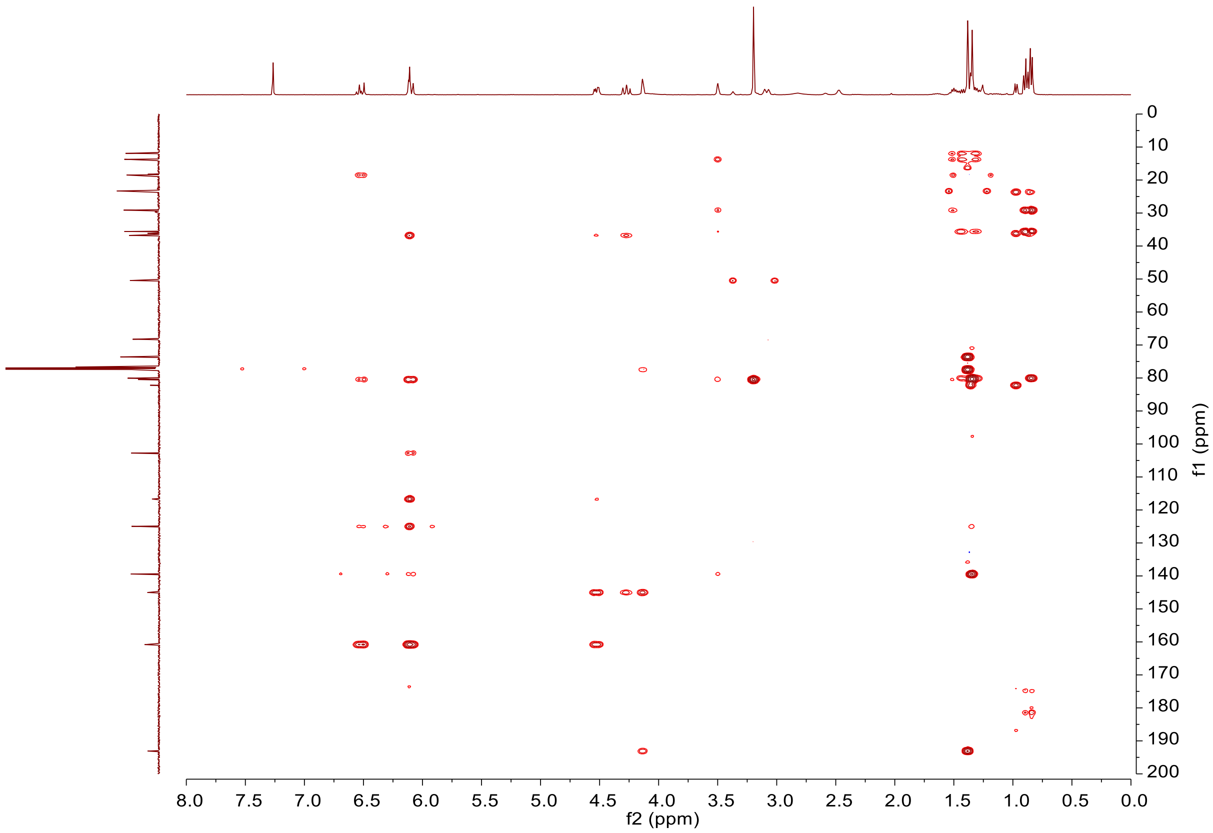

Figure S103. HMBC spectrum of peniphilone K (**13**)

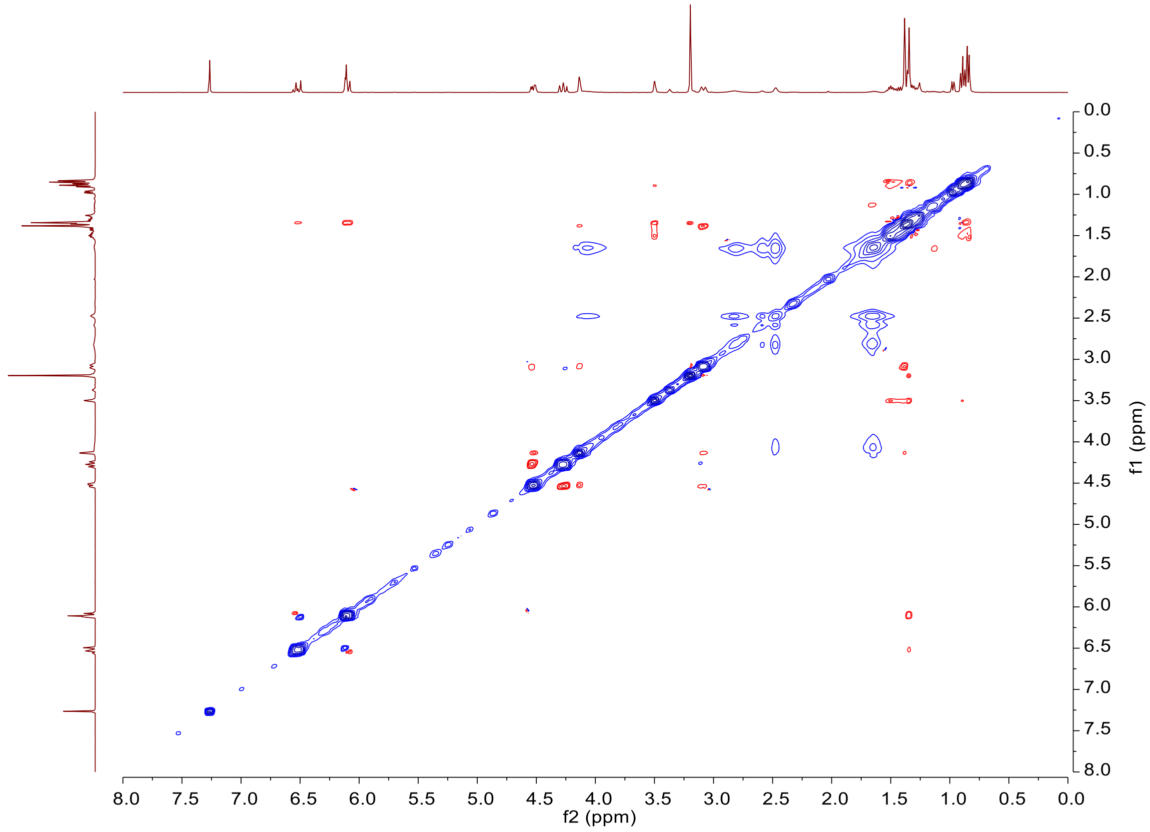

Figure S104. NOESY spectrum of peniphilone K (**13**)

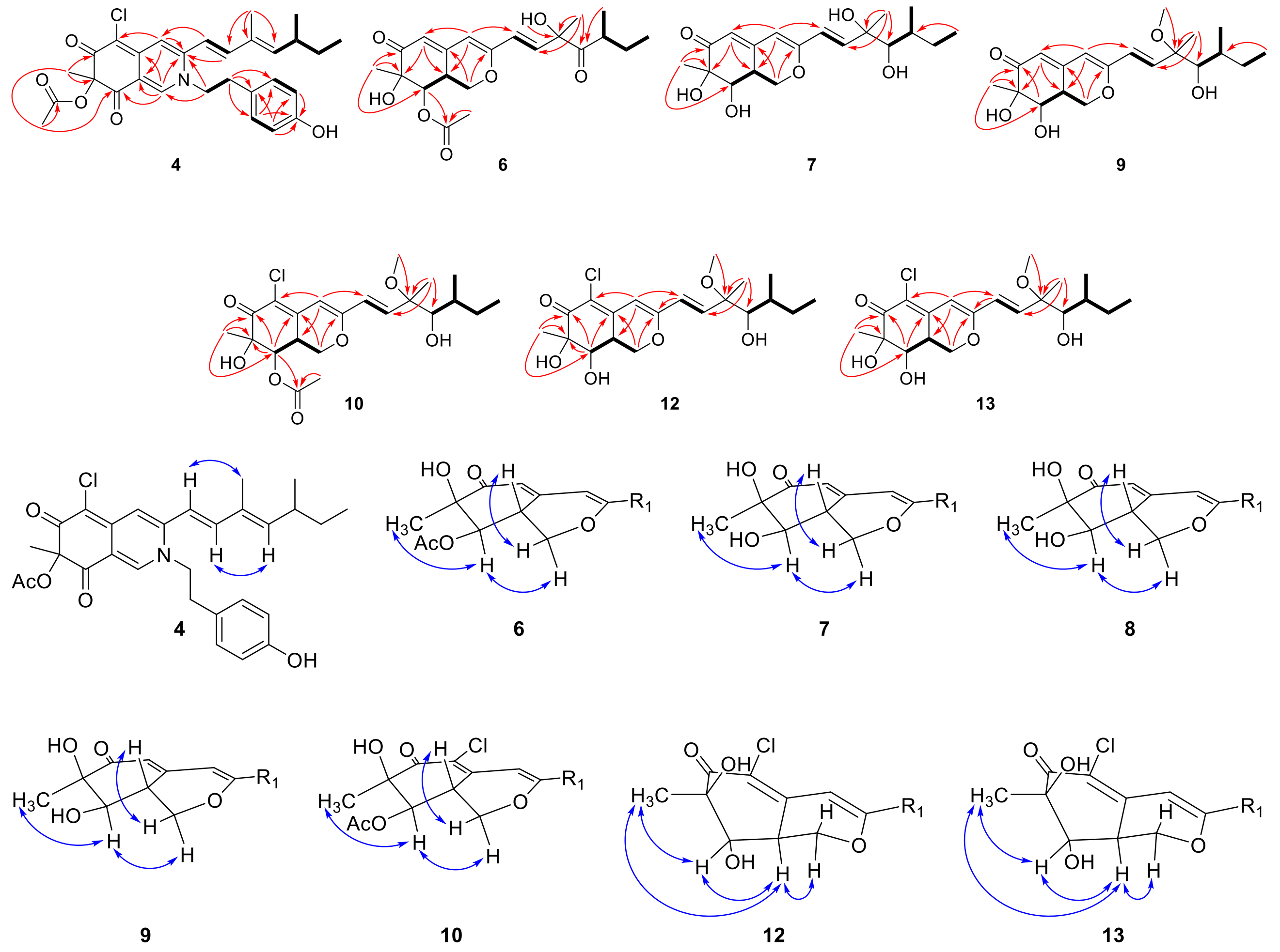

Figure S105. Key COSY (bold lines), HMBC (red arrows), and NOESY (blue double arrow) correlations of new compounds.
